# A Set of Global Metabolomic Biomarker Candidates to Predict the Risk of Dry Eye Disease

**DOI:** 10.3389/fcell.2020.00344

**Published:** 2020-06-08

**Authors:** Yaping Jiang, Chuanxi Yang, Yuxiang Zheng, Yining Liu, Yihui Chen

**Affiliations:** ^1^Department of Ophthalmology, Yangpu Hospital, Tongji University School of Medicine, Shanghai, China; ^2^Department of Cardiology, Jiangsu Province Hospital, Medical School of Southeast University, Nanjing, China; ^3^Yangpu Daqiao Community Health Service Center, Shanghai, China; ^4^Institute of Plant Physiology and Ecology, Shanghai Institutes for Biological Sciences, Shanghai, China

**Keywords:** metabolomic, dry eye disease, least absolute shrinkage and selection operator (LASSO) regression, tear profile, glucose metabolism, Glutathione metabolism, amino acid metabolism

## Abstract

**Purpose:**

We used ultraperformance liquid chromatography coupled with quadrupole/time-of-flight tandem mass spectrometry (UPLC-Q/TOF-MS/MS) to analyze the metabolic profile of reflex tears obtained from patients with dry eye disorders.

**Methods:**

We performed a cross-sectional study involving 113 subjects: 85 patients diagnosed with dry eye syndrome (dry eye group) and 28 healthy volunteers (control group). Reflex tears (20–30 μl) were collected from the tear meniscus of both eyes of each subject using a Schirmer I test strip. MS data were acquired with a standard workflow by UPLC-Q/TOF-MS/MS. Metabolites were quantitatively analyzed and matched with entries in the Metlin, Massbank, and HMDB databases. Least absolute shrinkage and selection operator (LASSO) regression was conducted to detect important metabolites. Multiple logistic regression was used to identify the significant metabolic biomarker candidates for dry eye syndrome. Open database sources, including the Kyoto Encyclopedia of Genes and Genomes and MetaboAnalyst, were used to identify metabolic pathways.

**Results:**

After the LASSO regression and multiple logistic regression analysis, 4 of 20 metabolic biomarker candidates were significantly correlated with Ocular Surface Disease Index score, 42 of 57 with fluorescein breakup time, and 26 of 57 with fluorescein staining. By focusing on the overlap of these three sets, 48 of 51 metabolites contributed to the incidence of dry eye and there were obvious changes in different age groups. Metabolic pathway analysis revealed that the main pathways were glucose metabolism, amino acid metabolism, and glutathione metabolism.

**Conclusion:**

Dry eye syndrome induces changes in the metabolic profile of tears, and the trend differs with age. This evidence reveals the relationship between changes in metabolites, symptoms of dry eye syndrome, and age.

## Introduction

Dry eye disease (DED) is one of the most common ocular surface diseases, with a prevalence of 20–50%, and is becoming a significant global health problem (Najafi et al., 2013; Stapleton et al., 2017). The Tear Film and Ocular Surface Dry Eye Workshop II (TFOS DEWS II) in 2017 provided a new definition: “Dry eye is a multifactorial disease of the ocular surface characterized by a loss of homeostasis of the tear film, and accompanied by ocular symptoms, in which tear film instability and hyperosmolarity, ocular surface inflammation and damage, and neurosensory abnormalities play etiological roles (Wolffsohn et al., 2017).” DED has a marked negative impact on the physical (Stapleton et al., 2017) and psychosomatic (Asiedu et al., 2018; Jonas et al., 2018) well-being of patients and exerts a substantial economic impact (Yu et al., 2011).

Despite its prevalence and impact, there is no “gold standard” diagnostic test for DED (Williamson et al., 2014; Valim et al., 2015). According to a report from the 2017 TFOS DEWS II, a diagnostic test battery for DED, including the screening 5-Item Dry Eye Questionnaire (DEQ-5) and Ocular Surface Disease Index (OSDI), confirms that a patient may have DED and triggers the diagnostic testing of non-invasive breakup time, osmolarity, and ocular surface staining with fluorescein and lissamine green (Wolffsohn et al., 2017). The DEQ-5 and OSDI questionnaires are used by patients to self-assess the symptoms, frequency, and severity of dry eye. Although the questionnaire score can roughly distinguish between dry eyes and non-dry eyes, a study conducted by Liu et al. (2019) showed that the perception of dry eye symptoms is not obvious due to lower corneal sensitivity. It is also affected by subjectivity, level of education, living environment, etc. Usually, the measurement of the tear film breakup time is the most frequent test of tear film stability in clinical practice. The measurement of non-invasive breakup time depends on the inspection equipment. Hence, the easy-to-operate fluorescein breakup time (FBUT) is most commonly used; however, it is affected by fluorescein, temperature, humidity, air circulation, etc. While osmolarity is considered the most relevant factor to the severity of dry eye, it is rarely used in outpatient clinics due to the high requirement for consumables and their instability. Ocular surface staining is a late feature of dry eye syndrome and a marker for the clinical diagnosis of severe dry eye syndrome, which has no significant value for the diagnosis of early dry eye. In summary, the above diagnostic methods are characterized by limitations for the diagnosis of dry eye. Therefore, the development of a diagnostic method with precise, stable, sensitive, specific, and convenient operation is urgently warranted.

Metabolomics is defined as the simultaneous qualitative and quantitative analysis of the metabolic response of living systems to pathophysiological stimuli or genetic modification. The main analytical techniques adopted in a global metabolomic analysis are nuclear magnetic resonance spectroscopy and mass spectrometry (MS) (Zhang et al., 2015). MS is usually combined with different separation techniques, such as liquid chromatography (LC) (Khamis et al., 2019), gas chromatography (Beale et al., 2018), and capillary electrophoresis (Ramautar et al., 2019), to enhance sensitivity. In the last 10 years, many studies have investigated the metabolite composition of the cornea, lens, and vitreous humor (Young and Wallace, 2009). Metabolomics has been used in clinical and animal studies of several eye diseases, including diabetic retinopathy (Chen et al., 2016), age-related macular degeneration (Brantley et al., 2012), glaucoma (Aribindi et al., 2013), keratoconus (Karamichos et al., 2015), and dry eye (Galbis-Estrada et al., 2014). With advances in technology, untargeted LC-MS metabolomic analysis of tear fluid in ocular diseases could be further conducted. Thus, investigations for the discovery of biomarker candidates for ocular diseases from tear fluid may also be useful for an accurate clinical diagnosis.

In the present study, we investigated alterations in the metabolic profile of tear fluid obtained from a clinical cohort of 113 subjects to detect metabolite aberrations in tears. Subsequently, all metabolites were separated by association with the clinical signs [OSDI, FBUT, corneal fluorescein staining (FL)] to identify the metabolites in tears that are pathologically relevant to DED. In addition, we constructed a more accurate metabolite model to assist us in the effective diagnosis of DED.

## Subjects and Methods

### Subjects and Groups

According to the diagnostic criteria and exclusion criteria for dry eye proposed in TFOS DEWS II, and considering the operability of the outpatient clinic, we used the following inclusion criterion: OSDI ≥ 13 or DEQ-5 ≥ 6. More strictly, FBUT < 10 s and ocular surface staining (+) were met. However, any of the following conditions were excluded: history of eye medication within 1 month; active inflammation of the eye or use of contact lens within 3 months; eye trauma or surgical history within 6 months; combined with hyperthyroidism, rheumatism, dry syndrome, and other diseases affecting tear secretion; presence of life-threatening primary diseases; or participation in other clinical trials. A total of 113 subjects were included in the final population. Tear samples (the tears of both eyes were treated as one sample) were divided into two groups: 28 and 85 samples were included in the control group (CG) and dry eye group (DEG), respectively. All subjects provided written informed consent. The study complied with the tenets of the Declaration of Helsinki for the protection of human subjects in medical research. Supplementary Table S1 lists the inclusion and exclusion criteria.

### Sample Collection

Firstly, each subject completed a form that included informed consent, basic information, past history, surgical history, medication history, family history, the OSDI questionnaire, and the DEQ-5 questionnaire. The OSDI questionnaire was used to assess the effects of ocular symptoms, visual function, and environmental factors in dry eye syndrome; a score ≥13 was considered meaningful. DEQ-5 was used to assess the duration of dry eye symptoms; a score ≥6 was considered meaningful. After completion of the questionnaire, each subject underwent systematic ophthalmologic examinations, including the Schirmer I test (SIT), tear meniscus height, FBUT, FL, corneal sensitivity, and meibomian gland function. After the SIT, both strips were placed in a single cryotube as one sample and stored in a −80°C refrigerator.

### Preparation of Tear Samples and Metabolomic Study

The SIT strip containing tears was removed from the −80°C refrigerator and immediately dissolved in 1 ml of 80% methanol. After ultrasonication, the supernatant was centrifuged (at 4,000 rpm), dried in a vacuum concentrator, and reconstituted with 10 μl of high-performance LC double-distilled water. One microliter of the sample was used for ultraperformance liquid chromatography coupled with quadrupole/time-of-flight mass spectrometry analysis. Liquid-chromatographic separation of processed tears was performed on a 100 × 2.1-mm Zorbax Eclipse Plus 1.8-μm C18 column using a 1290 infinity system, while MS was performed on a 6545 Quadrupole-Time of Flight system (all devices from Agilent Technologies, Santa Clara, CA, United States). Samples were randomly assigned to analyses. During analysis of each sample sequence, one quality control sample was run after every 20 injections. The gradient program of elution was: 1% B at 0–1 min, 15% B at 3 min, 70% B at 5 min, 85% at 9 min, 100% at 10–12 min, and subsequently return to the initial conditions with 2 min for equilibration. The sample volume injected was 2 μl, and the flow rate was 0.4 ml/min. The MS parameters were set as follows: fragmental voltage 135 V, nebulizer gas 35 psig, capillary voltage 4,000 V, drying gas 300°C flow 6 l/min, sheath gas 340°C flow 11 l/min. The data were collected with both centroid and profile stored in autoMSMS scan mode between a mass range MS of 100–3,200 m/z and MS/MS 30–3,200 m/z using the high-resolution mode (4 GHz). Reference masses at m/z 112.05087 and 922.009798 were introduced for online accurate mass calibration.

### Data Processing and Identification of Metabolites

The acquired MS data (.d) were exported to mzdata format using the MassHunter Workstation software (version B.07.00; Agilent Technologies). The program XCMS was applied for data pretreatment procedures, such as peak discrimination, filtering, and alignment. After peak alignment of the data in the time domain and automatic integration and extraction of the peak intensities, a list of the intensities of all the peaks detected was generated using the retention time and the mass-to-ratio data pairs as the parameters for each ion. MS/MS spectra of the selected putative identifications were retrieved and matched with entries in the Metlin, Massbank, and HMDB databases. Open database sources, including the Kyoto Encyclopedia of Genes and Genomes and MetaboAnalyst, were used to identify metabolic pathways.

### Statistical Analyses

Results are expressed as the mean ± standard deviation for continuous variables and as the number (percent) for categorical variables. Prior to the analysis, all metabolites were normalized to follow a normal distribution. Statistical analyses were performed using R 3.2.4. Firstly, unconditional logistic regression was performed to detect the association among metabolites and the four dry eye indexes (DEQ-5, OSDI, FBUT, and FL). Secondly, least absolute shrinkage and selection operator (LASSO) regression was conducted to detect important metabolites. Thirdly, the significant metabolic biomarker candidates were further confirmed by multiple logistic regression, with adjustment for covariates [i.e., age, sex, and hemoglobin A1c (HbA1c) levels]. Fourthly, the union set of metabolites associated with dry eye indexes was moved to the detection process of influence to dry eye. Two-sided *P* < 0.05 denoted statistically significant differences, and the *P*-values for each metabolite in all comparisons were corrected by false discovery rate correction. Finally, the expression of metabolites were converted into Log2(x + 1) values to normalize non-normal distributions. The normalized data were compared by unpaired Student’s *T*-test analysis.

## Results

### Demographic Data and Ocular Surface Parameters

A total of 113 subjects were enrolled in this study, with 85 patients in the DEG and 28 volunteers in the CG. The demographic data and results of ocular surface parameters are shown in Table 1. Briefly, there were 46.4% females in the CG and 41.2% in the DEG. The mean ages of subjects in the CG and DEG, with an unbalanced distribution, were 60.8 ± 11.2 and 55.4 ± 8.8 years, respectively (*P* < 0.0001). The mean levels of HbA1c in the CG and DEG were 4.86% ± 0.66% and 5.446% ± 0.16%, respectively. The results of the comparison showed that the OSDI and DEQ-5 scores of patients in the DEG were higher than those recorded in the CG (*P* < 0.0001 and *P* < 0.0001, respectively). The patients in the DEG showed lower FBUT values and SIT scores compared with those in the normal group (*P* < 0.0001 and *P* < 0.0001, respectively), strongly suggesting that tear film stability was altered in DEG patients. Additionally, the patients in the DEG showed lower corneal sensitivity and severe corneal injury compared with the subjects in the CG (*P* < 0.0001).

**TABLE 1:** Demographic and characteristics of study subjects in the control group and dry eye group.

**Feature**		**Normal (*n* = 28)**	**Dry eye (*n* = 85)**	***P* value**
Gender (Female, n(%))		13 46.4%	35 41.2%	0.78
Age (years)		60.8 ± 11.2	55.4 ± 8.8	<2.2 × 10^–16^
HbAlc (%)		4.86 ± 0.66	5.446 ± 0.16	0.02
FBUT (S)		12.46 ± 2.03	3.78 ± 1.66	<2.2 × 10^–16^
OSDI Scores		15.36 ± 15.34	24.99 ± 15.28	<2.2 × 10^–16^
DEQ-5		2.68 ± 2.37	4.95 ± 3.34	<2.2 × 10^–16^
FL (<5 sites)		28 100.0%	8 9.4%	<2.2 × 10^–16^
SIT (mm)		21.7 ± 1.46	7.754 ± 0.53	<0.0001
Corneal sensitivity				
	Normal	27 96.4%	15 17.6%	
	Moderate	1 3.6%	65 76.5%	
	Disappear	0	5 5.9%	
Meibomian gland dysfunction		6 21.4%	63 74.1%	
	Mild	6 100%	44 69.8%	
	Moderate	0	13 20.6%	
	Severe	0	6 9.6%	

*Non-parametric *t* tests; HbA1c, hemoglobin A1c; OSDI, Ocular Surface Disease Index; FBUT, fluorescein breakup time; DEQ-5, 5-Item Dry Eye Questionnaire; SIT, Schirmer I Test; FL, corneal fluorescein staining.*

### Association Among Metabolites and Dry Eye Indexes

**FIGURE 1:**
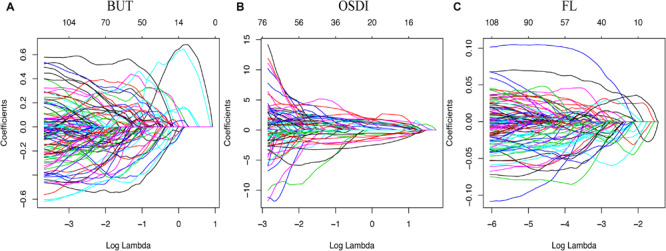
Model for least absolute shrinkage and selection operator (LASSO). Color lines represent the metabolites potentially associated with dry eye. The *x*-axis represents the alpha (cutoff), and the *y*-axis represents the shrink effect value.

**FIGURE 2:**
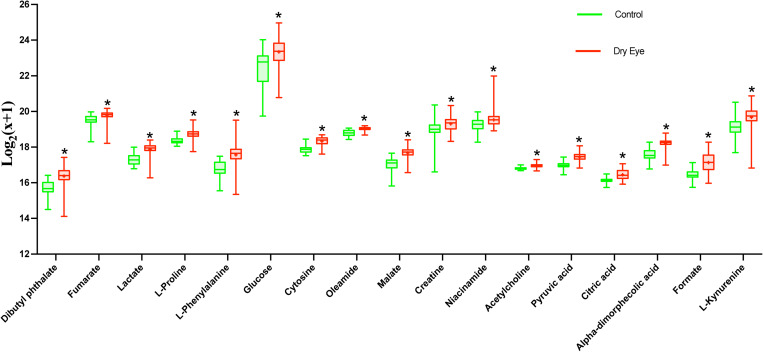
The 17 increasing metabolites presented by relative expression in the boxplot. *P < 0.05.

**FIGURE 3:**
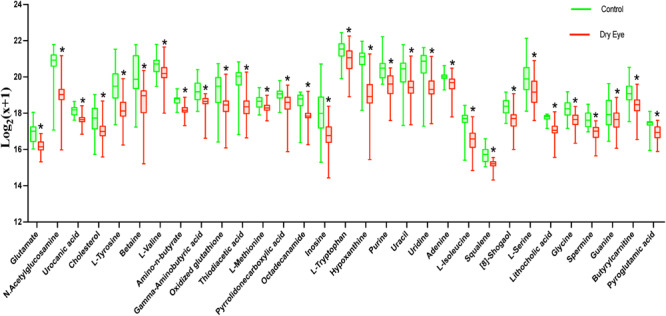
The 31 decreasing metabolites presented by relative expression in the boxplot. *P < 0.05.

The relationship among metabolites and dry eye indexes is demonstrated in Supplementary Table S2. The results showed that 37, 991, and 598 metabolic features exerted potential effects on the OSDI, FBUT, and FL, respectively. However, none of the metabolites were significantly associated with the DEQ-5 score, which was lower than the meaningful value in both groups. The LASSO regressions accurately identified the important metabolic biomarker candidates. As shown in Figures 1A–C, 20, 57, and 57 metabolic features were retained for FBUT, OSDI, and FL, respectively. After adjusting for age, sex, and HbA1c levels, 4 of 20 metabolic features remained significant for OSDI, 42 of 57 for FBUT, and 26 of 57 for FL (Tables 2–4). There is a potential mechanism by which metabolic features are related to OSDI, FBUT, or FL and may contribute to DED. Therefore, the biomarker candidate union set was further modified to confirm the relationship with DED. As shown in Table 5, 48 of 51 metabolic features contributed to the incidence of DED. Figures 2, 3 show the 17 increasing and 31 decreasing metabolites by fold change.

**TABLE 2:** The 4 most significant metabolites associated with OSDI among 20 significant metabolites adjusted for age, sex, and HbA1c levels.

**Metabolite**	**OR**	**CL**	**CU**	***t* value**	**P**	**FDR**
Thiodiacetic acid*	1.55247681	1.26158984	1.9104341	4.15516541	3.25E-05	0.0006501
Uridine*	1.40832563	1.14309313	1.73510018	3.21621402	0.00129894	0.01298939
Octadecanamide*	1.3405631	1.09814985	1.63648834	2.8800132	0.00397659	0.02651057
Phthalic anhydride*	0.81766755	0.67425718	0.99158043	−2.0459358	0.04076269	0.20381346
3-Acrylamidopropyl trimethylammonium	1.19362223	0.98364445	1.44842379	1.79294729	0.07298135	0.28723142
Triglyme	1.65924095	0.9305183	2.95865276	1.71595966	0.08616943	0.28723142
N-Heptane	1.19858444	0.95035917	1.51164392	1.5299583	0.12602705	0.36007729
1-Piperidinecarboxaldehyde	1.14944952	0.94898168	1.39226524	1.42445336	0.15431528	0.37131023
2-Methylbutyroylcarnitine	1.14444901	0.94508279	1.38587174	1.38161611	0.1670896	0.37131023
Palmitic amide	0.88257065	0.72651124	1.07215266	−1.2582488	0.20830179	0.41660359
Diglyme	0.92001999	0.75985836	1.11394022	−0.8542429	0.39297044	0.68896788
N-(3-Indolylac etyl)-L-isol eucine	1.08241214	0.89532492	1.30859312	0.81795877	0.41338073	0.68896788
N,N′-Dicyclohexylurea	0.94679681	0.77986533	1.14946027	−0.5524484	0.58064115	0.82684503
(S)-Desoxy-D2PM	1.05012842	0.86620605	1.27310321	0.49790162	0.61855338	0.82684503
Tuckolide	0.95474254	0.78708046	1.1581196	−0.4700638	0.63830943	0.82684503
Alanyl-Alanine	0.95900176	0.79513398	1.15664076	−0.4378762	0.66147602	0.82684503
Dihydroterrein	0.96411129	0.79540346	1.16860262	−0.3724069	0.70958995	0.83481171
Indoline	1.02916604	0.84773779	1.24942257	0.2905526	0.77139351	0.8571039
N-methyl corydaldine	1.00723376	0.82432556	1.23072714	0.07049554	0.94379925	0.98564298
(-)-Corey Lactone Diol	0.99823666	0.82365976	1.20981561	−0.0179948	0.98564298	0.98564298

OSDI, Ocular Surface Disease Index; HbA1c, hemoglobin A1c; OR, odds ratio; CL, lower of confidence interval; CU, upper of confidence interval. * *P* < 0.05.

**TABLE 3:** The 42 most significant metabolites associated with FBUT out of 57 significant metabolites adjusted of age, sex, and HbA1c level.

**Metabolite**	**OR**	**CL**	**CU**	***t* value**	**P**	**FDR**
Gamma-Aminobutyric acid*	2.31000409	1.72405625	3.09509559	5.60895326	2.04E-08	1.16E-06
L-Tyrosine*	2.85341036	1.92114399	4.23807415	5.19494456	2.05E-07	5.84E-06
Oxidized glutathione*	1.7939313	1.42065818	2.26528066	4.90996828	9.11E-07	1.73E-05
Cholesterol*	1.73958267	1.38364581	2.18708274	4.74020998	2.13E-06	3.04E-05
Glutamate*	1.61563596	1.31307235	1.98791755	4.53449486	5.77E-06	6.58E-05
fumarate*	0.64862127	0.52991355	0.79392111	–4.1976691	2.70E-05	0.00025619
Butyrylcarnitine*	1.70194948	1.32064315	2.19334955	4.10902868	3.97E-05	0.00032354
Lactate*	0.65144643	0.52930406	0.80177441	–4.04551	5.22E-05	0.00037199
Formate*	0.66830153	0.54536283	0.81895375	–3.8856717	0.00010205	0.00062014
acetylcholine*	0.65827145	0.53212825	0.81431741	–3.8524858	0.00011692	0.00062014
L-Isoleucine*	1.47248491	1.2089998	1.79339303	3.84678675	0.00011968	0.00062014
Oleamide*	0.66041693	0.53217629	0.81956023	–3.7664914	0.00016556	0.0007516
1-Stearoyl-2-Arachidonoyl PC*	1.4633179	1.19916256	1.78566222	3.74810872	0.00017817	0.0007516
Inosine*	2.65048162	1.59011429	4.41795465	3.73920617	0.0001846	0.0007516
Uracil*	1.46024988	1.19506913	1.7842731	3.70288298	0.00021316	0.00081002
Uridine*	1.47168572	1.19767109	1.80839204	3.67598012	0.00023694	0.00084409
Guanine*	1.50029386	1.20718859	1.86456506	3.65785151	0.00025434	0.00085278
L-Valine*	1.4420049	1.18277435	1.75805143	3.62023335	0.00029434	0.00090405
Dibutyl phthalate*	0.67430255	0.54455312	0.83496708	–3.6141379	0.00030135	0.00090405
L-Methionine*	1.47026597	1.18226511	1.8284241	3.46527672	0.00052969	0.00150961
Uracil*	1.42469938	1.15975359	1.75017206	3.37182114	0.00074673	0.00202684
Cytosine*	0.69994564	0.56654428	0.86475836	–3.306898	0.00094335	0.00244414
Uridine*	1.40832563	1.14309313	1.73510018	3.21621402	0.00129894	0.00321911
Squalene*	1.37339911	1.12939739	1.67011642	3.17930202	0.0014763	0.00350622
L-Kynurenine*	0.72719546	0.59496403	0.88881547	–3.111087	0.001864	0.00417575
malate*	0.72901762	0.59715129	0.89000341	–3.1046987	0.00190473	0.00417575
Spermine*	1.35525764	1.11347072	1.64954788	3.03203154	0.00242914	0.00512818
Citric acid*	0.73919799	0.60698117	0.90021518	–3.0055174	0.00265129	0.00539728
L-Serine*	1.42722391	1.12895219	1.80429969	2.97402045	0.00293925	0.00577716
Glucose*	0.73965682	0.60485591	0.90450007	–2.9378068	0.00330543	0.00628032
Pyroglutamic acid*	1.32121764	1.08651926	1.60661307	2.79159771	0.00524485	0.00964376
pyruvic acid*	0.75616671	0.61730273	0.92626852	–2.6998564	0.00693694	0.01231244
Adenine*	1.3194087	1.07818635	1.61459967	2.69079233	0.00712826	0.01231244
Glycocholic acid*	0.76903499	0.63133985	0.93676141	–2.6089881	0.00908104	0.0152241
L-Phenylalanine*	0.77952238	0.64158301	0.9471185	–2.506818	0.01218234	0.01983981
Alpha-dimorphecolic acid*	0.76942166	0.62336711	0.94969671	–2.4405653	0.0146643	0.02321847
Arginine*	0.78958675	0.64748007	0.96288251	–2.3336225	0.0196155	0.03021847
creatine*	0.79935316	0.6602648	0.96774124	–2.2962044	0.0216642	0.03249629
glycine*	1.24311063	1.02397819	1.50913765	2.19949186	0.02784297	0.04069357
Choline*	0.80593633	0.66141539	0.98203547	–2.13979	0.03237174	0.0455426
Niacinamide*	0.80940226	0.66658792	0.9828141	–2.1350282	0.03275871	0.0455426
4-Hydroxycitrulline*	1.24092841	1.01274466	1.52052476	2.08215659	0.03732817	0.05065966
PS(20:2(11Z,14Z)/16:0)	1.21081356	0.99481789	1.47370639	1.90817319	0.05636884	0.0745487
S-Glutathionyl-L-cysteine	0.82193434	0.67134384	1.0063041	–1.8991378	0.05754637	0.0745487
PS(22:4(7Z,10Z,13Z,16Z)/17:0)	1.2217118	0.96398133	1.5483492	1.65654564	0.09761137	0.12364107
Oleoyl Oxazolopyridine	0.85018063	0.69683413	1.03727281	–1.5993885	0.10973431	0.13597512
Triisobutyl phosphate	0.85487102	0.70210055	1.04088291	–1.5610858	0.11850352	0.14371703
2′-Hydroxy-a-naphthoflavone	0.84884908	0.68885126	1.04600922	–1.5378668	0.12408118	0.14529034
1-Oleoyl-2-acetyl-sn-glycerol	0.86144308	0.7120229	1.04221953	–1.5345325	0.12489871	0.14529034
1,2-Dierucoyl-SN-Glycero-3-Phosphoethanolamine	0.86269924	0.71131474	1.04630193	–1.5002282	0.13355529	0.15225303
S-Acetyldihydrolipoamide-E	0.86465346	0.71188448	1.05020635	–1.4661349	0.14261154	0.15938937
Tetrahydropteroyltri-L-glutamate	1.14762768	0.94841889	1.3886789	1.41556554	0.15690275	0.17047874
Streptidine 6-phosphate	0.87080284	0.71847067	1.05543292	–1.4100825	0.15851532	0.17047874
Succinoadenosine	1.1382523	0.93749381	1.38200198	1.30802614	0.19086444	0.20146802
PE(16:0/0:0)	0.88503301	0.71933451	1.08890011	–1.1547383	0.2481976	0.25722297
MG(8:2(9Z,12Z)/0:0/0:0)	0.92122998	0.75599324	1.12258236	–0.8134942	0.41593479	0.4233622
Tributyl phosphate	0.95282779	0.77633407	1.16944605	–0.4623322	0.64384309	0.64384309

OR, odds ratio; CL, lower of confidence interval; CU, upper of confidence interval; *p<0.05.

**TABLE 4:** The 26 most significant metabolites associated with FL among 57 significant metabolites adjusted for age, sex, and HbA1c levels.

**Metabolite**	**OR**	**CL**	**CU**	***t* value**	**P**	**FDR**
Betaine*	2.12966068	1.66438833	2.72499784	6.01077856	1.85E-09	1.05E-07
L-Tyrosine*	2.85341036	1.92114399	4.23807415	5.19494456	2.05E-07	5.84E-06
Oxidized glutathione*	1.7939313	1.42065818	2.26528066	4.90996828	9.11E-07	1.73E-05
N-Acetylglucosamine*	1.6785802	1.35712434	2.07617784	4.77550669	1.79E-06	2.55E-05
Lithocholic acid*	1.53009111	1.24630259	1.8784995	4.06364889	4.83E-05	0.00049599
Lactate*	0.65144643	0.52930406	0.80177441	−4.04551	5.22E-05	0.00049599
Pyrrolidonecarboxylic acid*	1.4633179	1.19916256	1.78566222	3.74810872	0.00017817	0.00145084
L-Methionine*	1.47026597	1.18226511	1.8284241	3.46527672	0.00052969	0.00377401
L-Tryptophan*	1.4145468	1.15893701	1.7265327	3.41056514	0.00064828	0.00378973
Urocanic acid*	1.40312876	1.15449382	1.70531039	3.40367209	0.00066487	0.00378973
Uracil*	1.42469938	1.15975359	1.75017206	3.37182114	0.00074673	0.00386942
[8]-Shogaol*	1.40526916	1.14462591	1.72526359	3.25052568	0.00115192	0.00547161
L-Proline*	0.72332176	0.59365193	0.88131504	−3.2134187	0.00131165	0.00575108
Purine*	1.39189424	1.12750237	1.71828426	3.07653288	0.00209423	0.00852652
Glucose*	0.73965682	0.60485591	0.90450007	−2.9378068	0.00330543	0.01256063
Pyruvic acid*	0.75616671	0.61730273	0.92626852	−2.6998564	0.00693694	0.02390062
Adenine*	1.3194087	1.07818635	1.61459967	2.69079233	0.00712826	0.02390062
Alpha-dimorphecolic acid*	0.76942166	0.62336711	0.94969671	−2.4405653	0.0146643	0.04643693
Phenylalanyl-Arginine*	1.26891943	1.04241067	1.54464699	2.37402965	0.01759514	0.050412
Dideoxymycobactin*	0.78981126	0.64990349	0.95983763	−2.3720766	0.01768842	0.050412
Glycerol tripropanoate*	1.25491863	1.03820859	1.51686354	2.34767749	0.01889087	0.05127522
Phenylpyruvic acid*	0.77780104	0.62924378	0.96143097	−2.3237225	0.02014037	0.05218187
Creatine*	0.79935316	0.6602648	0.96774124	−2.2962044	0.0216642	0.05368953
3-Formylsalicylic acid*	1.25266662	1.02631689	1.52893679	2.21546694	0.02672803	0.06347907
4-oxo-docosanoic acid*	1.23158951	1.01314323	1.49713553	2.09107893	0.03652099	0.08183483
3-Pyrimidin-2-yl-Propionic Acid*	1.24092841	1.01274466	1.52052476	2.08215659	0.03732817	0.08183483
Palmitoyl glucuronide	1.19088225	0.98170979	1.44462299	1.77268467	0.07628097	0.16103761
N-Acetylvanilalanine	0.84455515	0.6903238	1.0332447	−1.6421217	0.10056478	0.20472115
Myristoylcarnitine	0.85018063	0.69683413	1.03727281	−1.5993885	0.10973431	0.21568467
PG(P-18:0/0:0)	0.84884908	0.68885126	1.04600922	−1.5378668	0.12408118	0.23575425
3′-Keto-3′-deoxy-AMP	0.87080284	0.71847067	1.05543292	−1.4100825	0.15851532	0.29095774
2-glyceryl-PGF2α	1.14719977	0.94493723	1.39275634	1.38767768	0.16523521	0.29095774
Palmitoyl Ethanolamide	0.87101039	0.71559309	1.06018226	−1.3772039	0.16844922	0.29095774
Niacinamide	0.87638866	0.72270821	1.06274853	−1.3413271	0.17981427	0.30145333
N-palmitoyl leucine	0.878715	0.72528806	1.06459778	−1.3206278	0.1866255	0.30393295
1-Octen-3-yl glucoside	1.13228205	0.92938045	1.37948095	1.23308906	0.21754254	0.34444235
Inosine 2′-phosphate	0.89619591	0.73830719	1.08784949	−1.1084092	0.26768515	0.41237983
Prosopinine	0.91231546	0.74867246	1.11172715	−0.9098758	0.362888	0.52960861
Tetrandrine	0.90727914	0.73483704	1.12018775	−0.9047284	0.36560931	0.52960861
N-arachidonoyl tyrosine	1.09225223	0.90000599	1.32556332	0.89337729	0.37165516	0.52960861
3,7-Dimethyloctanal	0.92523277	0.76583693	1.11780413	−0.8055564	0.42049868	0.58459572
3-Amino-L-Tyrosine	0.92597687	0.76343194	1.1231298	−0.7809123	0.43485409	0.59015912
PS(12:0/18:3(6Z,9Z,12Z))	1.07789258	0.88891905	1.30703962	0.76269803	0.4456435	0.59073674
N-ethyl N-(2-hydroxy-ethyl) arachidonoyl amine	0.92694468	0.75277268	1.14141554	−0.7143992	0.47498035	0.61531545
Oleoyl Ethanolamide	0.94634878	0.78134734	1.14619448	−0.5641298	0.57266578	0.70474265
2-Methyl-1,3-oxathiane	0.94759109	0.78021615	1.15087193	−0.5428866	0.58720787	0.70474265
2,8-Dihydroxyadenine	1.05409689	0.87055921	1.27632932	0.53977667	0.58935106	0.70474265
3S-Aminodecanoic acid	1.05316396	0.86678598	1.27961728	0.52128152	0.60217067	0.70474265
DL-Glycerol 1-phosphate	1.05456176	0.86186592	1.29034051	0.51603294	0.6058314	0.70474265
Maprotiline glucuronide	0.96128301	0.79507038	1.16224306	−0.4076807	0.68350813	0.77919927
4-Methylene-L-glutamate	1.03562819	0.84849307	1.26403596	0.34428259	0.73063378	0.8165907
N,N-Dimethylaniline N-oxide	1.02864516	0.84885995	1.24650817	0.28815395	0.7732289	0.84757783
Cystamine	1.02490095	0.84205024	1.24745759	0.24531946	0.80620909	0.86705506
L-Homocysteic acid	1.02230671	0.83844272	1.24649065	0.21808909	0.8273597	0.8702951
PI(P-18:0/15:1(9Z))	0.9801715	0.80721801	1.19018179	−0.2022025	0.83975843	0.8702951
Propylthiouracil glucuronide	1.0084228	0.82428505	1.23369524	0.08153469	0.93501674	0.95171346
Butyl butyryllactate	0.99823666	0.82365976	1.20981561	−0.0179948	0.98564298	0.98564298

FL, corneal fluorescein staining; HbA1c, hemoglobin A1c; OR, odds ratio; CL, lower of confidence interval; CU, upper of confidence interval. * *P* < 0.05.

**TABLE 5:** The 48 most significant metabolites among 51 metabolites which contributed to the incidence of dry eye.

**Metabolite**	**OR**	**CL**	**CU**	**FDR**	**P**	**KEGG**	**HMDB**
Glutamate*	0.15892318	0.05881561	0.42941961	0.00474346	0.00028695	C00025	HMDB0000148
N-Acetylglucosamine*	0.33352376	0.18169142	0.61223639	0.00474346	0.00039529	C00140	HMDB0000215
Urocanic acid*	0.30038193	0.15246704	0.59179546	0.00474346	0.00050841	C00785	HMDB0000301
Dibutyl phthalate*	4.9575756	1.964163	12.512992	0.00474346	0.00070123	C14214	HMDB0033244
Cholesterol*	0.32553998	0.16974262	0.62433511	0.00474346	0.0007306	C00187	HMDB0000067
fumarate*	2.93700184	1.57053771	5.49237356	0.00474346	0.00074232	C00122	HMDB0000134
Lactate*	2.87485838	1.55392837	5.31865616	0.00474346	0.00076742	C00186	HMDB0000190
L-Tyrosine*	0.11616869	0.03287657	0.41047964	0.00474346	0.00082995	C00082	HMDB0000158
Betaine*	0.26844912	0.12382333	0.58199799	0.00474346	0.0008652	C00719	HMDB0000043
L-Valine*	0.34191908	0.18113828	0.6454111	0.00474346	0.00093009	C00183	HMDB0000883
Amino-n-butyrate*	0.23891563	0.09963151	0.57291791	0.00575326	0.00133553	NA	NA
Gamma-Aminobutyric acid*	0.23879924	0.09944765	0.57341804	0.00575326	0.00135371	C00334	HMDB0000112
Oxidized glutathione*	0.38068595	0.20887416	0.69382345	0.00632659	0.00161266	C00127	HMDB0003337
Thiodiacetic acid*	0.37739123	0.20461086	0.69607324	0.00653555	0.00180862	C01857	HMDB0031188
L-Methionine*	0.39076767	0.21502082	0.71016085	0.00653555	0.00204947	C00073	HMDB0000696
Pyrrolidonecarboxylic acid*	0.36061539	0.18740786	0.69390611	0.00653555	0.00225616	C02237	HMDB0000805
Octadecanamide*	0.39764946	0.21949705	0.72039732	0.00653555	0.00235239	C13846	HMDB0034146
L-Proline*	2.71415154	1.42470751	5.17061821	0.00653555	0.00239396	C00148	HMDB0000162
Inosine*	0.11144874	0.02697315	0.46048842	0.00653555	0.00243481	C00294	HMDB0000195
L-Tryptophan*	0.42621286	0.24230821	0.7496956	0.00784822	0.00307773	C00078	HMDB0000929
Hypoxanthine*	0.29993152	0.13456396	0.6685216	0.00785034	0.00323249	C00262	HMDB0000157
L-Phenylalanine*	3.67273631	1.51638767	8.89547722	0.00890948	0.0039459	C00079	HMDB0000159
Purine*	0.21222101	0.07380947	0.6101894	0.00890948	0.004018	C15587	HMDB0001366
Uracil*	0.41871956	0.22873078	0.76651719	0.01014436	0.00477382	C00106	HMDB0000300
Glucose*	2.83823315	1.3674437	5.89096821	0.01018904	0.00511095	C00031	HMDB0000122
Uridine*	0.41091757	0.220228	0.76672017	0.01018904	0.00519441	C00299	HMDB0000296
Cytosine*	2.74780932	1.32931549	5.67995794	0.01202255	0.00636488	C00380	HMDB0000630
Oleamide*	2.44685903	1.27149955	4.7087072	0.01344234	0.00738011	C19670	HMDB0002117
Adenine*	0.4549752	0.25202611	0.82135314	0.01578398	0.0089752	C00147	HMDB0000034
L-Isoleucine*	0.46790707	0.26221692	0.83494622	0.01712454	0.0101541	C00407	HMDB0000172
Squalene*	0.2605037	0.09280449	0.73123806	0.01712454	0.01063655	C00751	HMDB0000256
[8]-Shogaol*	0.46518125	0.25836679	0.83754416	0.01712454	0.01074481	C10494	HMDB0031463
malate*	2.83554082	1.26616827	6.35009732	0.01714406	0.01128604	C00149	HMDB0000156
creatine*	2.17111139	1.19063873	3.95898818	0.01714406	0.01142937	C00300	HMDB0000064
Niacinamide*	5.44745851	1.4046755	21.1257363	0.02073519	0.01423003	C00153	HMDB0001406
acetylcholine*	2.1406061	1.12609791	4.06909065	0.02830387	0.02021258	C01996	HMDB0000895
pyruvic acid*	2.33590317	1.13945145	4.78865828	0.02830387	0.02053418	C00022	HMDB0000243
L-Serine*	0.42525807	0.20369022	0.88784051	0.03060053	0.02280039	C00065	HMDB0000187
Lithocholic acid*	0.45386319	0.22816228	0.90283021	0.0318622	0.02436521	C03990	HMDB0000761
Citric acid*	2.05194986	1.08127092	3.89402706	0.03553974	0.0278743	C00158	HMDB0000094
glycine*	0.44191416	0.20966057	0.931449	0.03866868	0.03181944	C00037	HMDB0000123
Spermine*	0.34996191	0.1341625	0.91287309	0.03866868	0.0318448	C00750	HMDB0001256
Guanine*	0.59179132	0.36040424	0.97173375	0.04523848	0.03814225	C00242	HMDB0000132
Alpha-dimorphecolic acid*	2.04750337	1.03654999	4.0444456	0.04529542	0.0390784	C14767	HMDB0004670
Formate*	2.62276742	1.04426296	6.58733401	0.0455058	0.04015218	C00058	HMDB0000142
Butyrylcarnitine*	0.54961227	0.30885677	0.97803797	0.04589771	0.0417985	C02862	HMDB0002013
L-Kynurenine*	1.77187779	1.02008151	3.07774513	0.04589771	0.04229789	C00328	HMDB0000684
Pyroglutamic acid*	0.52951971	0.28364654	0.98852298	0.04877657	0.04590736	C01879	HMDB0000267
Arginine	3.00381699	0.99530218	9.06550465	0.05306126	0.05098043	C00062	HMDB0000517
Choline	1.73881637	0.90931083	3.32502625	0.09630046	0.09441221	C00114	HMDB0000097
Glycocholic acid	1.32778957	0.78435453	2.24774013	0.29114021	0.29114021	C01921	HMDB0000138

OR, odds ratio; CL, lower of confidence interval; CU, upper of confidence interval. * *P* < 0.05.

### Trends of Metabolites Related to Dry Eye in Different Age Groups

**FIGURE 4:**
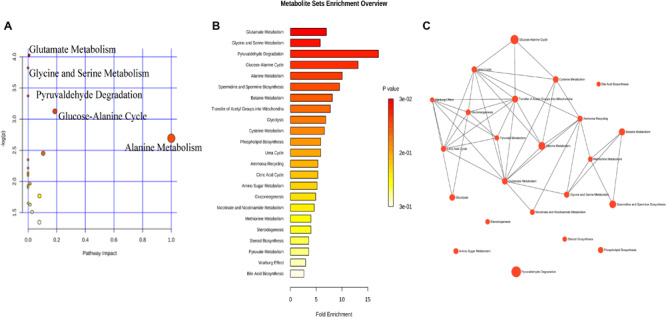
The metabolic pathways involved in dry eye disease at different ages. Six metabolites were subjected to analysis with Metaboanalyst to generate a topology map **(A)**, enrichment analysis network and table for the pathway-associated metabolite sets **(B,C)**.

We analyzed the relationship between metabolites and age. All subjects were grouped into two groups with the age nodes of 50 years (group A ≤ 50, group B > 50), 55 years (group C ≤ 55, group D > 55), and 60 years (group E ≤ 60, group F > 60). The increasing and decreasing metabolites are shown in Table 6. In group A, 18 of 48 metabolites did not show significance, and the remaining 30 metabolites showed the same trend in groups A and B. The 18 metabolites were subjected to enrichment and pathway analyses. Supplementary Figures S1A–C present the perturbed pathways. The 18 metabolites were related to the aminoacyl-tRNA biosynthesis, glycine, serine, and threonine metabolism, arginine and proline metabolism, valine, leucine, and isoleucine biosynthesis, pantothenate, and Coenzyme A biosynthesis. Comparison of groups C and D revealed that three metabolites (i.e., alpha-dimorphecolic acid, formate, and glycine), which were significant between the CG and DEG, did not show significance in each group. Of note, 39 metabolites showed the same trend in each group. Interestingly, acetylcholine, niacinamide, and pyruvic acid increased only in group D. The only decreasing metabolite in group C was cholesterol, while gamma-aminobutyric acid and L-methionine decreased in group D. In addition, an opposite trend was observed between groups C and D for L-serine. Enrichment and pathway analyses on the six metabolites are shown in Figures 4A–C. The data showed that the perturbed pathways and glutamate, glycine and serine, pyruvaldehyde, degradation, glucose–alanine cycle, and alanine metabolism were related. In the comparison of groups E and F, 10 of 48 metabolites did not show significance in group F, and the remaining 38 metabolites showed the same trend in each group. Enrichment and pathway analyses of the 10 metabolites showed that aminoacyl-tRNA biosynthesis, glycine, serine and threonine metabolism, glyoxylate and dicarboxylate metabolism, tryptophan metabolism, cysteine and methionine metabolism, and glutathione metabolism were involved (Supplementary Figures S2A–C).

**TABLE 6:** The increasing and decreasing metabolites in groups A–F.

**Group A **≦** 50 Control (*n* = 10), Dry Eye (*n* = 15)**		**Group B > 50 Control (*n* = 18), Dry Eye (*n* = 70)**		**Group C ≦ 5 Control (*n* = 16), Dry Eye (*n* = 42)**		**Group D > 55 Control (*n* = 12), Dry Eye (*n* = 43)**		**Group E ≦ 60 Control (*n* = 121), Dry Eye (*n* = 40)**		**Group F > 60 Control (*n* = 7), Dry Eye (*n* = 45)**	
**Increased**	**Decreased**	**Increased**	**Decreased**	**Increased**	**Decreased**	**Increased**	**Decreased**	**Increased**	**Decreased**	**Increased**	**Decreased**
Acetylcholine	Adenine	Acetylcholine	[8]-shogaol	Citric acid	[8]-shogaol	Acetylcholine	[8]-shogaol	Acetylcholine	[8]-shogaol	Acetylcholine	[8]-shogaol
Alpha-dimorphecolic acid	Amino-n-butyrate	Alpha-dimorphecolic acid	Adenine	Creatine	Adenine	Citric acid	Adenine	Alpha-dimorphecolic acid	Adenine	Alpha-dimorphecolic acid	Adenine
Citric acid	Cholesterol	Citric acid	Amino-n-butyrate	Cystosine	Amino-n-butyrate	Creatine	Amino-n-butyrate	Citric acid	Amino-n-butyrate	Citric acid	Amino-n-butyrate
Cystosine	Glutamate	Creatine	Betaine	Dibutyl Phthalate	Betaine	Cystosine	Betaine	Creatine	Betaine	Cystosine	Betaine
Dibutyl Phthalate	Glycine	Cystosine	Butyrylcamitine	Fumarate	Butyrylcamitine	Dibutyl Phthalate	Butyrylcamitine	Cystosine	Butyrylcamitine	Dibutyl Phthalate	Butyrylcamitine
Formate	Hypoxanthine	Dibutyl Phthalate	Cholesterol	Glucose	Gamma-Aminobutyric acid	Fumarate	Cholesterol	Dibutyl Phthalate	Cholesterol	Fumarate	Cholesterol
Fumarate	Inosine	Formate	Gamma-Aminobutyric acid	Lactate	Glutamate	Glucose	Glutamate	Formate	Gamma-Aminobutyric acid	Glucose	Gamma-Aminobutyric acid
Glucose	Lithocholic acid	Fumarate	Glutamate	L-Kynurenine	Guanine	Lactate	Guanine	Fumarate	Glutamate	Lactate	Glutamate
Lactate	L-Methionine	Glucose	Glycine	L-Phenyl alanine	Hypoxanthine	L-Kynurenine	Hypoxanthine	Glucose	Glycine	L-Phenyl alanine	Hypoxanthine
L-Phenyl alanine	L-tyrosine	Lactate	Guanine	L-Proline	Inosine	L-Phenyl alanine	Inosine	Lactate	Guanine	L-Proline	Inosine
Malate	Oxidized glutathione	L-Kynurenine	Hypoxanthine	Malate	L-Isoleucine	L-Proline	L-Isoleucine	L-Kynurenine	Hypoxanthine	Malate	L-Isoleucine
Oleamide	Purine	L-Phenyl alanine	Inosine	Oleamide	Lithocholic acid	L-Serine	Lithocholic acid	L-Phenyl alanine	Inosine	Niacinamide	Lithocholic acid
Pyruvic acid	Pyroglutamic acid	L-Proline	L-Isoleucine		L-Methionine	Malate	L-Tryptophan	L-Proline	L-Isoleucine	Oleamide	L-Tyrosine
	Pyrrolidonc carboxylic acid	Malate	Lithocholic acid		L-Serine	Niacinamide	L-Tyrosine	Malate	Lithocholic acid	Pyruvic acid	L-Valine
	Spermine	Niacinamide	L-Methionine		L-Tryptophan	Oleamide	L-Valine	Niacinamide	L-Methionine		N-Acetylglu cosamine
	Squalene	Oleamide	L-serine		L-Tyrosine	Pyruvic acid	N-Acetylglu cosamine	Oleamide	L-Serine		Octadecanamide
	Urocanic acid	Pyruvic acid	L-Tryptophan		L-Valine		Octadecanamide	Pyruvic acid	L-Tryptophan		Oxidized glutathione
			L-Tyrosine		N-Acetylglu cosamine		Oxidized glutathione		L-Tyrosine		Pyrrolidonc carboxylic acid
			L-Valine		Octadecanamide		Purine		L-Valine		Spermine
			N-Acetylglu cosamine		Oxidized glutathione		Pyroglutamic acid		N-Acetylglu cosamine		Squalene
			octadecanamide		Purine		Spermine		Octadecanamide		Thiodiacetic acid
			Oxidized glutathione		Pyroglutamic acid		Squalene		Oxidized glutathione		Uracil
			Purine		Pyrrolidonc carboxylic acid		Thiodiacetic acid		Purine		Uridine
			Pyroglutamic acid		Spermine		Uracil		Pyroglutamic acid		Urocanic acid
			Pyrrolidonc carboxylic acid		Squalene		Uridine		Pyrrolidonc carboxylic acid		
			Spermine		Thiodiacetic acid		Urocanic acid		Spermine		
			Squalene		Uracil				Squalene		
			Thiodiacetic acid		Uridine				Thiodiacetic acid		
			Uracil		Urocanic acid				Uracil		
			Uridine						Uridine		
			Urocanic acid						Urocanic acid		

*Group A age: ≤50 years, group B age: >50 years, group C age: ≤55 years, group D age: >55 years, group E age: ≤60 years, group F age: >60 years.*

### Metabolic Pathways Involved in Dry Eye Disease

**FIGURE 5:**
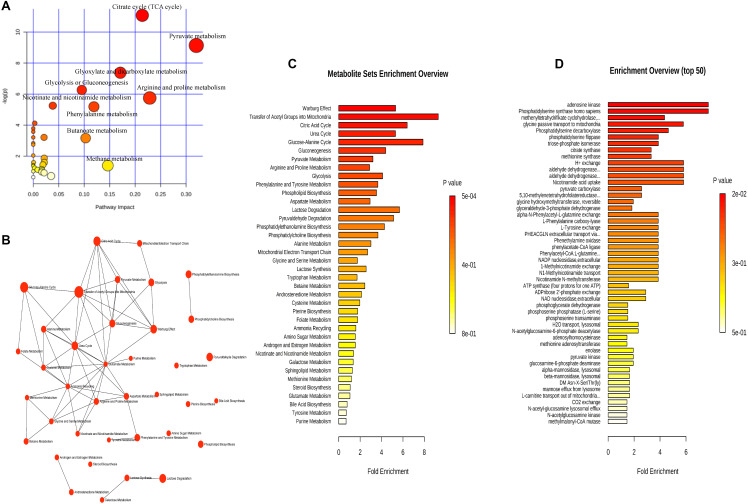
The increasing metabolic pathways involved in dry eye disease. The 17 increasing metabolites were subjected to analysis with Metaboanalyst (http://www.metaboanalyst.ca) to generate a topology map **(A)**, enrichment analysis network and table for the pathway-associated metabolite sets **(B,C)**, and enrichment analysis table for the predicted metabolite enzyme sets **(D)**.

**FIGURE 6:**
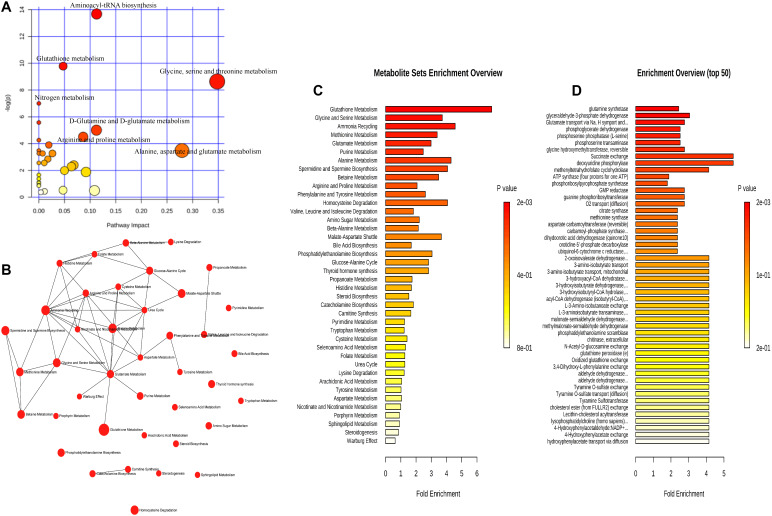
The decreasing metabolic pathways involved in dry eye disease. The 31 decreasing metabolites were subjected to analysis with Metaboanalyst to generate a topology map **(A)**, enrichment analysis network and table for the pathway-associated metabolite sets **(B,C)**, and enrichment analysis table for the predicted metabolite enzyme sets **(D)**.

**FIGURE 7:**
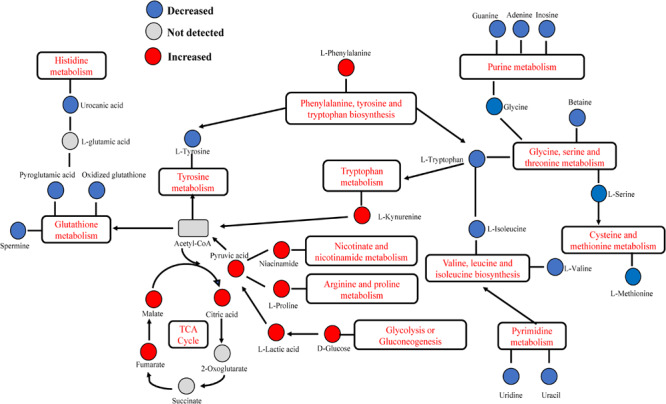
Dry eye disease-related altered metabolic pathway network of the significantly regulated metabolites. Several metabolic pathways of amino acids were activated: tryptophan, arginine-proline, and cysteine-methionine metabolism. Notably, phenylalanine-tyrosine-tryptophan biosynthesis, glycine-serine-threonine metabolism, and valine-leucine-isoleucine biosynthesis were decreased. The increased levels of malate, citric acid, fumarate, and lactic acid indicated significant elevation of the tricarboxylic acid (TCA) cycle and glycolysis or gluconeogenesis. Metabolites of adenine, guanine, and inosine were significantly inhibited, suggesting decreased purine metabolism. Histidine and glutathione metabolism was inhibited, indicated by a decrease in urocanic acid, pyroglutamic acid, oxidized glutathione, and spermine. Pyrimidine metabolism was inhibited as indicated by the levels of uridine and uracil.

For the interpretation of differences in metabolites that may be involved in the pathophysiological mechanism of DED, 17 increasing and 31 decreasing metabolites were subjected to enrichment and pathway analyses. Figures 5A–C, 6A–C present the perturbed pathways. In the DEG versus the CG, the increasing metabolites were related to the citrate cycle, nicotinate and nicotinamide, butanoate, arginine-proline, pyruvate, glycolysis or gluconeogenesis, glyoxylate-dicarboxylate, and phenylalanine; the decreasing metabolites were related to glutathione, glycine-serine-threonine, nitrogen, d-glutamine and d-glutamate, and alanine-aspartate-glutamate. Furthermore, we comprehensively analyzed the metabolites that have been associated with DED. As shown in Figure 7, malate, fumarate, niacinamide, L-lactic acid, pyruvic acid, D-glucose, L-proline, citric acid, L-kynurenine, and L-phenylalanine were significantly elevated along with their metabolic pathways. In contrast, spermine, oxidized glutathione, pyroglutamic acid, urocanic acid, L-valine, L-tyrosine, L-methionine, L-isoleucine, glycine, guanine, adenine, inosine, and uridine were decreased as a result of the inhibition of metabolic pathways. In addition, metabolic enzymes that may be involved in the changes in metabolites are shown in Figures 5D, 6D.

## Discussion

Dry eye disease is an ocular surface disorder in which the tear film is unstable due to multiple factors (Craig et al., 2017). Studying changes in the chemical composition of tears is the most direct approach to exploring the mechanism of dry eye development. In an early study conducted by Srinivasan et al. (2012) using the protein quantification method, statistically significant differences in the protein ratios were detected between the normal and dry eye groups. Lam et al. (2014) underscored the pathological relevance of structure-specific alterations in tear lipid components, particularly wax esters, to the pathogenesis of DED. In recent years, an increasing number of research studies have been devoted to identifying differential biomarker candidates. Perumal et al. (2016) compared tears between aqueous-deficient, evaporative dry eye patients and healthy subjects, showing that 13 major proteins were differentially expressed. A similar study performed by Huang et al. (2018) ascertained the differential expression profiles of 18 proteins (*P* < 0.05) with a fast proteomic method based on LC-quadrupole-orbitrap-MS analysis.

The global-omics platform has been widely used in the study of the tear proteome and lipidome (Lam et al., 2014). However, the analysis of tear metabolites is limited to the analysis of targeted compound classes, and the number of identified metabolites is also markedly lower than that of other biomolecules identified in tears. This observation may be attributed to challenges in instrument sensitivity, as the typical sample volume of tears is only 5–10 μl. Galbis-Estrada et al. (2014) suggested for the first time that the severity of dry eye exerts a significant effect on tear metabolism, suggesting that the tear metabolome may be utilized to identify biomarker candidates and surrogate endpoints for DED. In this study, we improved the utilization of tears by optimizing the collection of samples and using high-resolution MS. Previous research directly compared the statistically significant increase or decrease of chemicals between tears of patients with DED and healthy subjects (Galbis-Estrada et al., 2015). We used the LASSO algorithm, which refers to the variable selection method (minimizing the log partial to the sum of the absolute values of the parameters being bounded by a constant) to identify the significant metabolites associated with DED.

By focusing on the union of the sets of metabolites that were correlated with the OSDI, FBUT, and FL, we confirmed 48 significantly different metabolites between the DEG and CG. As previously reported, the major metabolites were amino acids, carbohydrates, and lipids (Butovich, 2008; Chen et al., 2011; Nakatsukasa et al., 2011; Rantamaki et al., 2011; Zhou and Beuerman, 2012). Firstly, increased levels of glucose (Taormina et al., 2007), lactate (Rucker et al., 2003), and creatine (Zhou and Beuerman, 2012) have been reported in patients with dry eye, suggesting that these patients may be in a state of high energy metabolism. However, glutathione, cholesterol, and N-acetylglucosamine were noticeably decreased in the DEG versus the CG. In addition, previous proteomics studies in patients with dry eye reported that the lower expression of lactoferrin, lipocalin, and lipophilin AC-1 (Baca et al., 2007) may render these patients more susceptible to infections because of the increased oxidative stress and reduced antimicrobial proteins (Saijyothi et al., 2012). Moreover, the levels of kynurenine increased as a result of the catabolism of tryptophan, which is involved in the inflammatory mechanisms of Sjögren’s syndrome with dry eye (de Oliveira et al., 2018).

Various adenine and uracil nucleotides have been shown to improve corneal barrier function and increase both tear fluid secretion and corneal epithelial resistance (Fujihara et al., 2001). Kim and Kang (2013) reported that pyrimidine nucleoside uridine exerts a protective effect on cultured human corneal epithelial cells in an animal model of dry eye and in patients. Our results showed that decreased levels of adenine, uridine, pyrimidine, and uracil may contribute to lower corneal sensitivity.

Ding and Sullivan (2012) clearly demonstrated that aging is a risk factor for dry eye, and the mechanism of age-related increase in the incidence of DED has also been elucidated. Interestingly, when we grouped all patients according to different age nodes, various changes were observed. The analysis revealed that the basal secretion of human tears decreased with the increase of age; we found that acetylcholine in tears was significantly increased in group D and can activate the muscarinic receptor to cause reflex secretion of tears (Mauduit et al., 1993), which may be a kind of negative feedback regulation. Wang et al. (2018) found that nicotinamide and pyruvate accumulated to higher levels in old mice, and cholesterol was significantly lower in the aged cornea, while 1-methylNAA may be a hallmark for the aging eye. Notably, 1-methylNAA is produced by N-methyltransferase, transferring methyl groups from S-adenosylmethionine to nicotinamide (Wang et al., 2018), which is consistent with the high expression of nicotinamide and pyruvic acid, and the low expression of cholesterol in group D. In addition, taurine is one of the most abundant amino acids in the retina (Lei et al., 2011), cornea, and lens. The levels of taurine in the rat cornea and lens decrease with age (Yanshole et al., 2014), and the main pathway of taurine biosynthesis is methionine and cysteine. Hence, we infer that the reduction in L-methionine may be related to taurine metabolism. Gao et al. (2018) revealed that serine is involved in lipid metabolism and thus affects mitochondrial metabolism. Dry eye is also closely related to lipid metabolism; however, the mechanism of change in the levels of serine remains unclear. It is well established that gamma-aminobutyric acid is an important inhibitory neurotransmitter in the central nervous system and plays an important role in tissue development and biological signal communication; however, its mechanism of action in the ocular surface remains unknown. Regarding the three metabolites not exhibiting any trend, we found that their *P*-values prior to grouping were close to 0.05. Therefore, we hypothesized that the test effect may be influenced by the change in sample size after grouping. In addition, 18 of 48 metabolites in group A, seven of 48 in group C, five of 48 in group D, and 10 of 48 in group F did not show significance, indicating that the expression of metabolites related to dry eye changed with age. Based on the number of metabolites which did not show significance in each group, we can infer that the metabolites related to the dry eye significantly increase from the age of 50 years. This is consistent with the findings of the TFOS DEWS II Epidemiology Report (Stapleton et al., 2017). Moreover, the number of meaningful metabolites related to dry eye was greater between the age of 50 and 60 years, which indirectly reflected the relationship between the symptoms of dry eye and age. At the age of 50–60 years, when the patients experience the most obvious symptoms of dry eye, and with increasing age, the sensitivity appears to be decreasing. This effect may be related to weakened corneal perception. The metabolic pathways associated with differential metabolites in different groups are partly different, which may be related to different endocrine levels at each age. However, the mechanism involved in this process is unclear.

A number of reports have been published in recent years on the components of tears. However, differences in the collection method, the instrument for the detection of metabolites, and the technique used for the metabolomic analysis contribute to the discrepancies observed between the reports. Our study had several limitations. Firstly, there was a significant difference in age between the CG and the DEG; however, the appropriate statistical procedure was performed to adjust for age. Secondly, our study did not analyze the different metabolites in the dry eye subgroups. Precise statistical analyses were essential to guarantee the consistency of our data.

In summary, metabolomics can be used to extract molecular information from human tears. This information can be useful for the accurate diagnosis of DED, prediction of prognosis, and development of personalized therapies. Our data demonstrated the different metabolomic profiles between individuals with and without dry eye and between different age groups. Furthermore, they partly illustrate the relationship between changes in metabolites, symptoms in dry eye, and age.

## Biosecurity Statement

All experimental procedures described in this article adhered to biosecurity and institutional safety standards.

## Data Availability Statement

The raw data supporting the conclusions of this article will be made available by the authors, without undue reservation, to any qualified researcher.

## Ethics Statement

This study was performed in accordance with the principles of the Declaration of Helsinki, and the study protocol was reviewed and approved by the Ethics Committee of Yangpu District Central Hospital. Informed consent was provided by all subjects enrolled in this study.

## Author Contributions

YC designed the work, performed the data analysis, and prepared the manuscript. CY and YJ performed the experiments and wrote the manuscript. YZ and YL analyzed and interpreted the data. All authors discussed the results and read and approved the final version of the manuscript for publication.

## Conflict of Interest

The authors declare that the research was conducted in the absence of any commercial or financial relationships that could be construed as a potential conflict of interest.

## Supplementary Material

FIGURE S1 The metabolic pathways involved in dry eye disease at different ages. Eighteen metabolites were subjected to analysis with Metaboanalyst to generate a topology map **(A)**, enrichment analysis network and table for the pathway-associated metabolite sets **(B,C)**.

FIGURE S2 The metabolic pathways involved in dry eye disease at different ages. Ten metabolites were subjected to analysis with Metaboanalyst to generate a topology map **(A)**, enrichment analysis network and table for the pathway-associated metabolite sets **(B,C)**.

TABLE S1 Inclusion and exclusion criteria.

TABLE S2 The relationship between metabolites and dry eye indexes. OSDI, Ocular Surface Disease Index; FBUT, fluorescein breakup time; DEQ-5, 5-Item Dry Eye Questionnaire; FL, corneal fluorescein staining; OR, odds ratio; CL, lower of confidence interval; CU, upper of confidence interval.
