# Supplementary figures and images for: A Set of Global Metabolomic Biomarker Candidates to Predict the Risk of Dry Eye Disease
Source: Front Cell Dev Biol. 2020 Jun 8;8:344. doi: 10.3389/fcell.2020.00344 (PMC7295093; doi:10.3389/fcell.2020.00344)

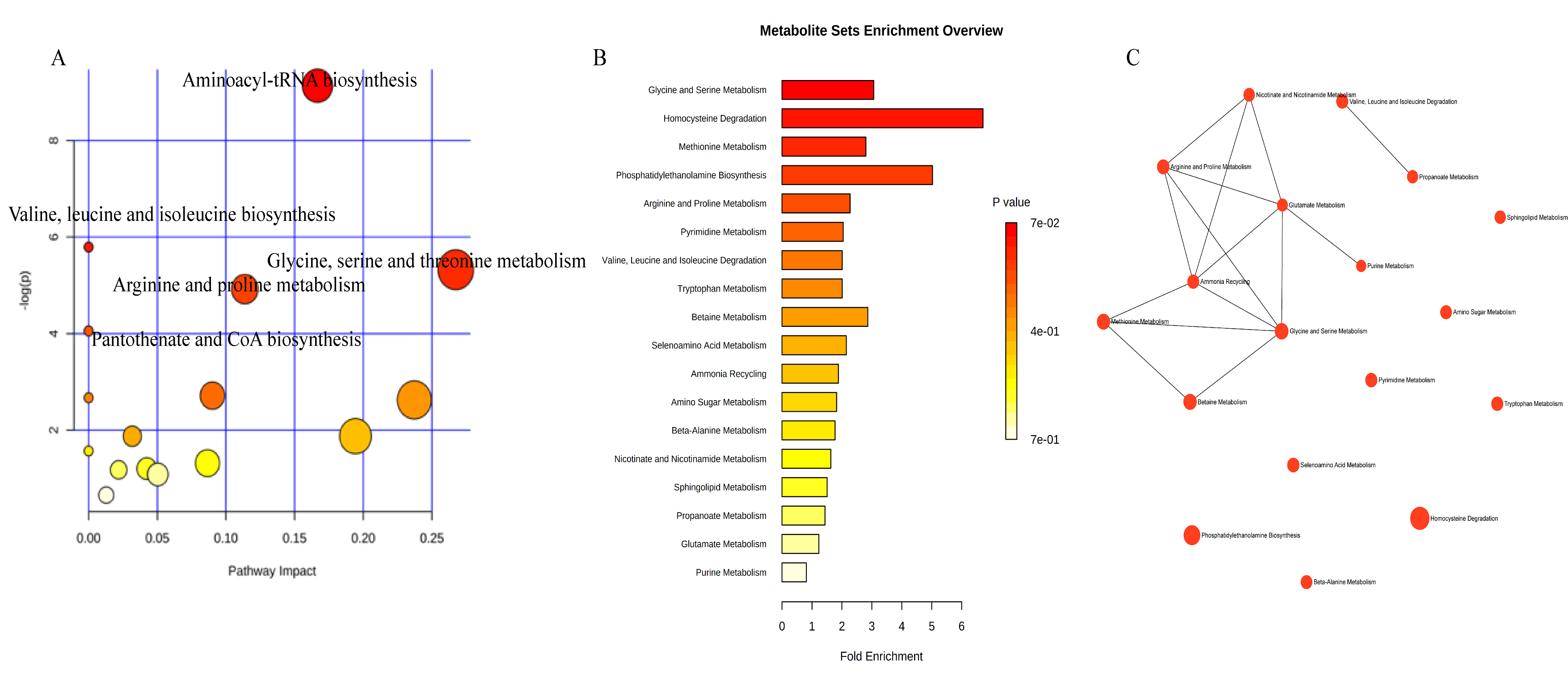

Supplement: FIGURE S1 — The metabolic pathways involved in dry eye disease at different ages. Eighteen metabolites were subjected to analysis with Metaboanalyst to generate a topology map (A), enrichment analysis network and table for the pathway-associated metabolite sets (B,C). [file Image_1.tif]

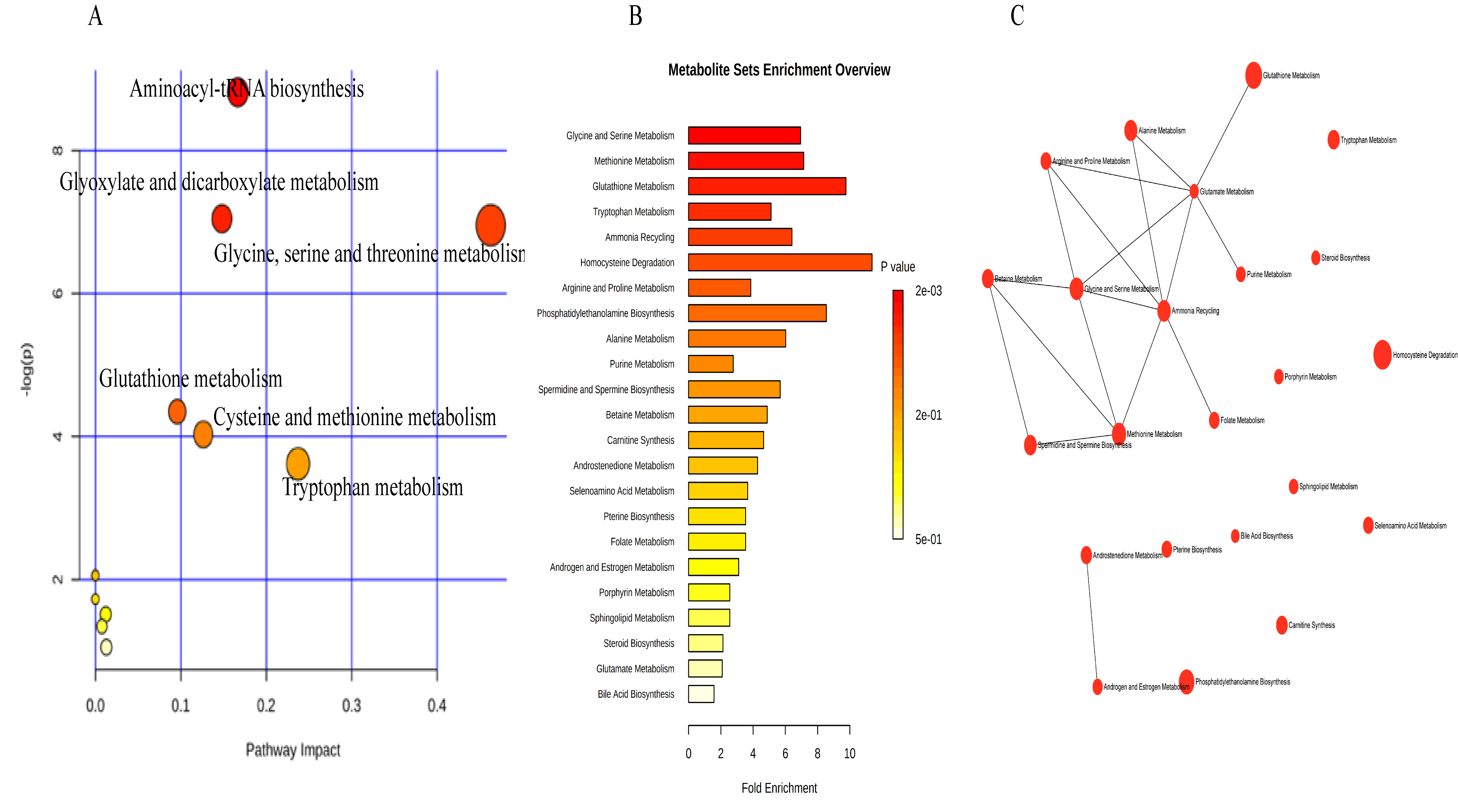

Supplement: FIGURE S2 — The metabolic pathways involved in dry eye disease at different ages. Ten metabolites were subjected to analysis with Metaboanalyst to generate a topology map (A), enrichment analysis network and table for the pathway-associated metabolite sets (B,C). [file Image_2.tif]
