# Supplementary material for: A Set of Global Metabolomic Biomarker Candidates to Predict the Risk of Dry Eye Disease
Source: Front Cell Dev Biol. 2020 Jun 8;8:344. doi: 10.3389/fcell.2020.00344 (PMC7295093; doi:10.3389/fcell.2020.00344)
Supplement: TABLE S1 — Inclusion and exclusion criteria. [file Table_1.docx]

Table1: Inclusion criteria and exclusion criteria

However, any of the following conditions will be excluded: There is a history of eye medication within 1 month active inflammation of the eye or contact lens wear within 3 months, eye trauma or surgical history within 6 months, combined with hyperthyroidism, rheumatism, dry syndrome and other diseases affecting tear secretion, suffering from life-threatening primary diseases or participating in other clinical trials.
