# Supplementary material for: A Set of Global Metabolomic Biomarker Candidates to Predict the Risk of Dry Eye Disease
Source: Front Cell Dev Biol. 2020 Jun 8;8:344. doi: 10.3389/fcell.2020.00344 (PMC7295093; doi:10.3389/fcell.2020.00344)
Supplement: TABLE S2 — The relationship between metabolites and dry eye indexes. OSDI, Ocular Surface Disease Index; FBUT, fluorescein breakup time; DEQ-5, 5-Item Dry Eye Questionnaire; FL, corneal fluorescein staining; OR, odds ratio; CL, lower of confidence interval; CU, upper of confidence interval. [file Table_2.docx]

| metabolites | FBUT | | | | |  | DEQ | | | | |  | OSDI | | | | |  | FL | | | | |
| --- | --- | --- | --- | --- | --- | --- | --- | --- | --- | --- | --- | --- | --- | --- | --- | --- | --- | --- | --- | --- | --- | --- | --- |
|  | OR | CL | CU | P | FDR |  | OR | CL | CU | P | FDR |  | OR | CL | CU | P | FDR |  | OR | CL | CU | P | FDR |
| metabolite1 | 165.2353184 | 0.721723 | 37829.9209 | 0.068091 | 0.168009563 |  | 0.911881 | 0.011847 | 70.19123 | 0.966872 | 0.984108828 |  | 1.07819E-09 | 1.21829E-18 | 0.954207216 | 0.051976 | 0.392904029 |  | 0.528598 | 0.287246 | 0.972741 | 0.042838 | 0.151259058 |
| metabolite2 | 3.21904E-06 | 3.6E-08 | 0.000287941 | 2.29E-07 | 1.57E-05 |  | 1.68867 | 0.031147 | 91.55246 | 0.797513 | 0.920823742 |  | 0.000404045 | 1.82442E-12 | 89482.0362 | 0.427139 | 0.774359849 |  | 2.912658 | 1.704813 | 4.97625 | 0.000158 | 0.003846653 |
| metabolite3 | 0.001501026 | 6.85E-06 | 0.328998884 | 0.019803 | 0.067484605 |  | 0.456818 | 0.005912 | 35.30067 | 0.72459 | 0.880180765 |  | 0.000184726 | 1.5089E-13 | 226149.3097 | 0.422422 | 0.77189627 |  | 1.302468 | 0.700464 | 2.421855 | 0.40549 | 0.614831664 |
| metabolite4 | 775.1460592 | 2.276716 | 263911.4641 | 0.027312 | 0.08633446 |  | 75.28983 | 0.738848 | 7672.153 | 0.069676 | 0.291180836 |  | 2.63219E+11 | 65.8132689 | 1.05274E+21 | 0.02155 | 0.283005659 |  | 0.759364 | 0.388804 | 1.483097 | 0.42197 | 0.625122608 |
| metabolite5 | 0.440497065 | 0.001236 | 157.0518662 | 0.785015 | 0.875040525 |  | 38.95763 | 0.400102 | 3793.272 | 0.119761 | 0.372764958 |  | 1767789.697 | 0.000415054 | 7.52934E+15 | 0.206162 | 0.597241307 |  | 1.761671 | 0.916267 | 3.387097 | 0.092346 | 0.250890714 |
| metabolite6 | 8.40843E-06 | 8.15E-08 | 0.000867818 | 2.78E-06 | 8.74E-05 |  | 0.857172 | 0.015197 | 48.34651 | 0.940423 | 0.97690349 |  | 10.05130383 | 3.58557E-08 | 2817650791 | 0.816553 | 0.946906762 |  | 2.814929 | 1.634232 | 4.848654 | 0.000303 | 0.005616989 |
| metabolite7 | 0.056199913 | 0.000252 | 12.52745102 | 0.298936 | 0.470748957 |  | 0.096025 | 0.001361 | 6.773076 | 0.282902 | 0.570570429 |  | 5.36396E-07 | 6.93484E-16 | 414.8922196 | 0.169527 | 0.568636202 |  | 0.731032 | 0.397518 | 1.34436 | 0.315667 | 0.527891308 |
| metabolite8 | 3.38047E-06 | 2.3E-08 | 0.000497579 | 2.71E-06 | 8.66E-05 |  | 84.13221 | 1.184143 | 5977.512 | 0.043958 | 0.236415195 |  | 147416.3251 | 0.000132039 | 1.64584E+14 | 0.265283 | 0.650304541 |  | 2.492533 | 1.371403 | 4.530194 | 0.003374 | 0.028447123 |
| metabolite9 | 7.320796811 | 0.02534 | 2115.002663 | 0.492496 | 0.650747709 |  | 4.724855 | 0.054526 | 409.4269 | 0.496585 | 0.736835733 |  | 123.6535298 | 5.38824E-08 | 2.8377E+11 | 0.662182 | 0.897173851 |  | 1.075939 | 0.567649 | 2.039367 | 0.822897 | 0.911301022 |
| metabolite10 | 0.384576646 | 0.001139 | 129.8383886 | 0.748273 | 0.852314445 |  | 5.394194 | 0.055547 | 523.8298 | 0.471883 | 0.718413961 |  | 10026.59104 | 2.62762E-06 | 3.82599E+13 | 0.414842 | 0.769732538 |  | 1.249412 | 0.649142 | 2.404756 | 0.506445 | 0.692234584 |
| metabolite11 | 4.84829E-06 | 3.02E-08 | 0.000779239 | 6.88E-06 | 0.000160628 |  | 548.9077 | 8.041632 | 37467.48 | 0.004147 | 0.1046835 |  | 40253924.62 | 0.033875488 | 4.78334E+16 | 0.10332 | 0.49665678 |  | 3.555373 | 1.988841 | 6.355799 | 3.99E-05 | 0.001825815 |
| metabolite12 | 1.107648518 | 0.004593 | 267.0938947 | 0.970924 | 0.984976957 |  | 0.002377 | 3.67E-05 | 0.153956 | 0.005378 | 0.113768772 |  | 1.44474E-10 | 1.97744E-19 | 0.105554452 | 0.03168 | 0.323868345 |  | 0.616636 | 0.334616 | 1.136344 | 0.123945 | 0.29948526 |
| metabolite13 | 0.119637156 | 0.000464 | 30.84025643 | 0.455104 | 0.620004423 |  | 0.112807 | 0.001435 | 8.865142 | 0.329223 | 0.605724591 |  | 7.37598E-05 | 5.20064E-14 | 104612.1976 | 0.378084 | 0.737889126 |  | 0.822466 | 0.439831 | 1.537977 | 0.541771 | 0.713960117 |
| metabolite14 | 0.003837235 | 1.28E-05 | 1.150875857 | 0.058492 | 0.150842888 |  | 2.381397 | 0.024872 | 228.0101 | 0.709998 | 0.873029036 |  | 7.365233759 | 2.01472E-09 | 26925146680 | 0.859254 | 0.958572817 |  | 1.762578 | 0.925345 | 3.357322 | 0.087493 | 0.243027857 |
| metabolite15 | 0.002428583 | 9.23E-06 | 0.638691168 | 0.036433 | 0.10662558 |  | 7.389378 | 0.085438 | 639.0927 | 0.381332 | 0.653135786 |  | 28083.07653 | 1.28029E-05 | 6.16002E+13 | 0.352643 | 0.719834155 |  | 1.74574 | 0.928142 | 3.28356 | 0.086653 | 0.241237713 |
| metabolite16 | 2.261340693 | 0.009648 | 530.0117513 | 0.770014 | 0.866770268 |  | 7.569192 | 0.104549 | 547.9985 | 0.356225 | 0.629200512 |  | 0.000129308 | 1.35966E-13 | 122976.4813 | 0.397787 | 0.757408315 |  | 1.184783 | 0.64112 | 2.189467 | 0.589468 | 0.755880139 |
| metabolite17 | 2.660911375 | 0.009414 | 752.1210557 | 0.734612 | 0.844007535 |  | 0.009233 | 0.000118 | 0.722988 | 0.037477 | 0.220921127 |  | 2.89097E-07 | 1.67157E-16 | 499.9913382 | 0.16811 | 0.568636202 |  | 0.504567 | 0.270552 | 0.940994 | 0.033623 | 0.131481225 |
| metabolite18 | 0.019454552 | 3.35E-05 | 11.28113372 | 0.227485 | 0.390371454 |  | 0.068485 | 0.000453 | 10.35663 | 0.297338 | 0.579217354 |  | 4572901712 | 0.176296019 | 1.18615E+20 | 0.07174 | 0.447917707 |  | 1.355476 | 0.660294 | 2.782573 | 0.408959 | 0.61692996 |
| metabolite19 | 0.000414982 | 2.07E-06 | 0.083206805 | 0.004782 | 0.022295326 |  | 64.61904 | 0.915167 | 4562.686 | 0.057528 | 0.264422304 |  | 2833.693147 | 2.55927E-06 | 3.13755E+12 | 0.455941 | 0.791392334 |  | 2.948435 | 1.641953 | 5.294468 | 0.000445 | 0.007208601 |
| metabolite20 | 1.591906625 | 0.005881 | 430.898612 | 0.871051 | 0.930364047 |  | 0.523673 | 0.00637 | 43.05314 | 0.774227 | 0.908230447 |  | 0.008831957 | 5.14583E-12 | 15158564.76 | 0.663727 | 0.898220689 |  | 1.122543 | 0.597499 | 2.10896 | 0.72006 | 0.846417908 |
| metabolite21 | 564075.4079 | 3642.397 | 87354854.82 | 1.15E-06 | 4.66E-05 |  | 0.586732 | 0.007074 | 48.66742 | 0.813456 | 0.926554541 |  | 1.40808E-05 | 8.56503E-15 | 23148.81185 | 0.304423 | 0.688849398 |  | 0.213232 | 0.121435 | 0.374421 | 4.19E-07 | 8.65E-05 |
| metabolite22 | 729.2421443 | 3.917212 | 135758.327 | 0.014953 | 0.054456043 |  | 0.125175 | 0.001859 | 8.429208 | 0.335393 | 0.611358189 |  | 545755.6726 | 0.000878083 | 3.39204E+14 | 0.203657 | 0.597241307 |  | 0.422176 | 0.235656 | 0.756325 | 0.004515 | 0.034261821 |
| metabolite23 | 1.08321E-05 | 6.35E-08 | 0.001846629 | 2.91E-05 | 0.000482247 |  | 31.95185 | 0.420047 | 2430.494 | 0.11984 | 0.372764958 |  | 0.720488917 | 4.803E-10 | 1080792329 | 0.975794 | 0.991698663 |  | 2.42022 | 1.32232 | 4.429688 | 0.004975 | 0.036665306 |
| metabolite24 | 5.23065E-05 | 6.1E-07 | 0.004485296 | 3.15E-05 | 0.000508551 |  | 4.054807 | 0.092342 | 178.0499 | 0.469696 | 0.717917212 |  | 639.459753 | 7.57315E-06 | 53994568168 | 0.48926 | 0.805878285 |  | 1.789594 | 1.05191 | 3.044602 | 0.033997 | 0.131606193 |
| metabolite25 | 2543.654827 | 14.04251 | 460756.6142 | 0.003807 | 0.018688892 |  | 3.053071 | 0.043673 | 213.4309 | 0.607518 | 0.811847126 |  | 0.008305189 | 1.04195E-11 | 6619907.39 | 0.647752 | 0.889606808 |  | 0.496637 | 0.274157 | 0.899664 | 0.022807 | 0.099805557 |
| metabolite26 | 0.000789858 | 3.1E-06 | 0.2010154 | 0.012889 | 0.048460849 |  | 322.9318 | 4.147243 | 25145.61 | 0.010585 | 0.135235336 |  | 2219593571 | 1.286675114 | 3.82894E+18 | 0.049811 | 0.387346738 |  | 2.284462 | 1.225265 | 4.259295 | 0.010614 | 0.059411473 |
| metabolite27 | 0.196898736 | 0.000538 | 72.05766373 | 0.590539 | 0.732180031 |  | 986.7935 | 11.25339 | 86530.48 | 0.003132 | 0.096298132 |  | 4152.861666 | 7.75929E-07 | 2.22266E+13 | 0.467545 | 0.795534682 |  | 2.059895 | 1.073192 | 3.95378 | 0.031957 | 0.127108339 |
| metabolite28 | 0.634857611 | 0.002092 | 192.6793017 | 0.876463 | 0.932230925 |  | 616.7949 | 8.045166 | 47287.52 | 0.004476 | 0.107162727 |  | 150347.4902 | 6.23943E-05 | 3.62283E+14 | 0.281794 | 0.665728265 |  | 1.659781 | 0.877895 | 3.138044 | 0.121785 | 0.297136655 |
| metabolite29 | 3.17815E-05 | 1.65E-07 | 0.006106383 | 0.000191 | 0.001937643 |  | 19.01368 | 0.239205 | 1511.338 | 0.1898 | 0.465845944 |  | 32565.11043 | 2.04639E-05 | 5.18222E+13 | 0.338527 | 0.713403042 |  | 3.565329 | 1.986701 | 6.398331 | 4.29E-05 | 0.001881693 |
| metabolite30 | 0.001395541 | 5.68E-06 | 0.342911016 | 0.021011 | 0.070883528 |  | 20.70454 | 0.253173 | 1693.219 | 0.180195 | 0.45552071 |  | 0.000104805 | 5.57969E-14 | 196858.5971 | 0.402107 | 0.760948731 |  | 2.660379 | 1.447733 | 4.888759 | 0.002086 | 0.020813109 |
| metabolite31 | 0.182705798 | 0.000697 | 47.89707012 | 0.550874 | 0.702506744 |  | 0.003035 | 4.3E-05 | 0.214471 | 0.008756 | 0.126652263 |  | 3.22759E-11 | 3.27199E-20 | 0.031837898 | 0.024138 | 0.294657193 |  | 0.71641 | 0.383381 | 1.338731 | 0.298069 | 0.511658131 |
| metabolite32 | 1245.756058 | 3.513481 | 441700.9974 | 0.019041 | 0.065367715 |  | 4.041636 | 0.03559 | 458.966 | 0.56413 | 0.787805088 |  | 2.027299198 | 2.37838E-10 | 17280440040 | 0.951806 | 0.986747222 |  | 0.61486 | 0.314035 | 1.203854 | 0.158763 | 0.345610269 |
| metabolite33 | 3219105.911 | 19678.19 | 526605610.4 | 7.56E-08 | 6.68E-06 |  | 0.614997 | 0.006345 | 59.61401 | 0.835366 | 0.93545489 |  | 240.9653845 | 6.39961E-08 | 9.0731E+11 | 0.626834 | 0.879305472 |  | 0.28791 | 0.156115 | 0.530969 | 0.00012 | 0.003137511 |
| metabolite34 | 7.44572E-05 | 2.59E-07 | 0.021429608 | 0.001342 | 0.008631692 |  | 28.73551 | 0.280542 | 2943.338 | 0.157876 | 0.424548994 |  | 0.000222263 | 3.82692E-14 | 1290876.433 | 0.46491 | 0.792880411 |  | 3.477388 | 1.858213 | 6.507447 | 0.000166 | 0.003901274 |
| metabolite35 | 0.00207908 | 1.43E-05 | 0.301903062 | 0.016635 | 0.05930012 |  | 449.9219 | 9.486253 | 21339.27 | 0.002433 | 0.089390474 |  | 23.0783698 | 8.65447E-08 | 6154177249 | 0.751763 | 0.935229984 |  | 2.160243 | 1.237214 | 3.771902 | 0.007829 | 0.049021219 |
| metabolite36 | 0.000782487 | 4.27E-06 | 0.14341022 | 0.008238 | 0.033968221 |  | 37.6226 | 0.575029 | 2461.544 | 0.091818 | 0.328579908 |  | 1.34291E+13 | 39521.83659 | 4.56309E+21 | 0.003175 | 0.117813005 |  | 2.214131 | 1.230452 | 3.984205 | 0.009176 | 0.054137506 |
| metabolite37 | 0.00011266 | 5.09E-07 | 0.02492807 | 0.0013 | 0.008462449 |  | 329.7239 | 4.37272 | 24862.75 | 0.009779 | 0.13042689 |  | 391617.0145 | 0.000206717 | 7.41904E+14 | 0.239901 | 0.630505297 |  | 2.632327 | 1.428333 | 4.851213 | 0.00243 | 0.023240612 |
| metabolite38 | 6.26428093 | 0.017007 | 2307.349951 | 0.54402 | 0.69567761 |  | 11.9119 | 0.115281 | 1230.852 | 0.297367 | 0.579217354 |  | 1503.303826 | 2.68545E-07 | 8.41543E+12 | 0.524271 | 0.824725981 |  | 1.197921 | 0.615535 | 2.33133 | 0.596081 | 0.760941521 |
| metabolite39 | 0.000102621 | 6.92E-07 | 0.015226361 | 0.000476 | 0.003859334 |  | 5.367341 | 0.084713 | 340.07 | 0.428989 | 0.691262593 |  | 984.9066354 | 1.96491E-06 | 4.93682E+11 | 0.501478 | 0.813012498 |  | 2.020052 | 1.130349 | 3.610042 | 0.019332 | 0.088069497 |
| metabolite40 | 300.3016705 | 0.998773 | 90291.88298 | 0.052552 | 0.140202132 |  | 0.550432 | 0.005708 | 53.0819 | 0.798326 | 0.920845809 |  | 1.801244646 | 4.7803E-10 | 6787189183 | 0.958376 | 0.9871034 |  | 0.608418 | 0.318519 | 1.162168 | 0.135203 | 0.312998899 |
| metabolite41 | 704392.6308 | 4842.501 | 102461303 | 5.95E-07 | 3.07E-05 |  | 0.280323 | 0.0035 | 22.45192 | 0.570703 | 0.790253764 |  | 11.30383042 | 7.20509E-09 | 17734214319 | 0.822788 | 0.948840121 |  | 0.332155 | 0.183482 | 0.601296 | 0.000416 | 0.006949231 |
| metabolite42 | 1018.723747 | 3.082145 | 336712.9196 | 0.021053 | 0.070912031 |  | 19.69343 | 0.189037 | 2051.615 | 0.211298 | 0.489772916 |  | 613.1717676 | 9.93228E-08 | 3.78543E+12 | 0.57793 | 0.857194036 |  | 0.656196 | 0.337562 | 1.275597 | 0.21676 | 0.413572088 |
| metabolite43 | 2.46755E-05 | 1.21E-07 | 0.005026834 | 0.000159 | 0.001676389 |  | 145.6434 | 1.845097 | 11496.42 | 0.027432 | 0.19379821 |  | 34114546.45 | 0.01909886 | 6.09357E+16 | 0.113369 | 0.513062936 |  | 2.527323 | 1.366255 | 4.675086 | 0.003825 | 0.03063876 |
| metabolite44 | 8.1623E-05 | 4.37E-07 | 0.015256658 | 0.000612 | 0.004689462 |  | 14.16931 | 0.189303 | 1060.573 | 0.231127 | 0.512158638 |  | 205708.9693 | 0.000184411 | 2.29466E+14 | 0.25219 | 0.64285589 |  | 1.903247 | 1.03446 | 3.501679 | 0.040877 | 0.146989084 |
| metabolite45 | 0.008754779 | 2.92E-05 | 2.627317593 | 0.106342 | 0.233050076 |  | 0.024386 | 0.000273 | 2.178047 | 0.107992 | 0.354968411 |  | 0.120728791 | 3.63069E-11 | 401451373.4 | 0.850436 | 0.955623187 |  | 0.702456 | 0.367886 | 1.341297 | 0.286849 | 0.499763282 |
| metabolite46 | 1.23564E-06 | 1.09E-08 | 0.000140482 | 1.36E-07 | 1.04E-05 |  | 37.51059 | 0.578151 | 2433.698 | 0.091438 | 0.328579908 |  | 892443.8429 | 0.001457744 | 5.46362E+14 | 0.187122 | 0.586045046 |  | 4.773898 | 2.809729 | 8.11114 | 6.96E-08 | 3.69E-05 |
| metabolite47 | 3.196055691 | 0.01205 | 847.7096498 | 0.683999 | 0.806840056 |  | 0.011416 | 0.000152 | 0.856917 | 0.044739 | 0.237074035 |  | 0.000481559 | 3.08007E-13 | 752903.6834 | 0.480928 | 0.800476896 |  | 0.412705 | 0.224885 | 0.757389 | 0.005106 | 0.037427713 |
| metabolite48 | 0.000267258 | 1.55E-06 | 0.046196628 | 0.002236 | 0.012748126 |  | 0.059427 | 0.000891 | 3.962147 | 0.190395 | 0.466888136 |  | 2.62997E-08 | 4.61547E-17 | 14.9859778 | 0.092533 | 0.485016082 |  | 2.122059 | 1.177451 | 3.82448 | 0.01375 | 0.070773455 |
| metabolite49 | 11.87273563 | 0.045644 | 3088.265438 | 0.385069 | 0.554302037 |  | 1.001025 | 0.012363 | 81.05133 | 0.999636 | 0.999753507 |  | 0.01934278 | 1.21608E-11 | 30766311.72 | 0.715818 | 0.920092212 |  | 0.891513 | 0.475676 | 1.670877 | 0.720801 | 0.846684943 |
| metabolite50 | 6817.583127 | 31.4133 | 1479610.309 | 0.001704 | 0.010298851 |  | 0.057009 | 0.000702 | 4.630742 | 0.20433 | 0.48289214 |  | 7.45566E-08 | 4.85814E-17 | 114.4200211 | 0.131158 | 0.530263915 |  | 0.338993 | 0.185895 | 0.618179 | 0.000608 | 0.008988394 |
| metabolite51 | 7.819234244 | 0.033576 | 1820.947202 | 0.461137 | 0.625696571 |  | 1.246145 | 0.016869 | 92.05783 | 0.920327 | 0.9726671 |  | 0.000253477 | 2.59087E-13 | 247989.1493 | 0.434727 | 0.779380463 |  | 0.994467 | 0.537404 | 1.840264 | 0.985935 | 0.992083499 |
| metabolite52 | 5638.467613 | 30.9786 | 1026267.216 | 0.001513 | 0.009412724 |  | 0.098488 | 0.001381 | 7.023599 | 0.289349 | 0.573923383 |  | 0.000118604 | 1.30995E-13 | 107385.48 | 0.392141 | 0.751670347 |  | 0.467534 | 0.257368 | 0.849323 | 0.014022 | 0.071775342 |
| metabolite53 | 8.241959204 | 0.036215 | 1875.752117 | 0.447859 | 0.614243311 |  | 0.18423 | 0.002567 | 13.22221 | 0.4395 | 0.696406905 |  | 2.47337E-05 | 2.8552E-14 | 21425.99424 | 0.314578 | 0.697572488 |  | 0.754798 | 0.409827 | 1.390146 | 0.368582 | 0.583535522 |
| metabolite54 | 14.74590382 | 0.059334 | 3664.702118 | 0.341019 | 0.514438912 |  | 0.198931 | 0.002566 | 15.42336 | 0.468468 | 0.717917212 |  | 0.00246097 | 1.84547E-12 | 3281742.733 | 0.576352 | 0.85695946 |  | 0.72896 | 0.39173 | 1.356501 | 0.320586 | 0.533496176 |
| metabolite55 | 202368.6804 | 1182.876 | 34621617.77 | 8.96E-06 | 0.000197817 |  | 0.010541 | 0.000137 | 0.812896 | 0.04238 | 0.235275011 |  | 4.26239E-07 | 2.68761E-16 | 675.9887504 | 0.177497 | 0.575285157 |  | 0.355783 | 0.194598 | 0.650476 | 0.00108 | 0.013237413 |
| metabolite56 | 536104.2921 | 3240.132 | 88702497.33 | 1.67E-06 | 6.04E-05 |  | 1.185908 | 0.013673 | 102.8579 | 0.940441 | 0.97690349 |  | 0.005786853 | 2.63195E-12 | 12723520.46 | 0.639674 | 0.884587563 |  | 0.400536 | 0.216483 | 0.741071 | 0.004309 | 0.033117315 |
| metabolite57 | 128402337.6 | 656429.3 | 25116428962 | 2.83E-10 | 1.17E-07 |  | 0.043019 | 0.000308 | 6.0103 | 0.214527 | 0.493557558 |  | 9.59356E-06 | 3.99336E-16 | 230473.6183 | 0.345459 | 0.715799853 |  | 0.218307 | 0.113651 | 0.419336 | 1.27E-05 | 0.000739244 |
| metabolite58 | 363.5510183 | 1.130053 | 116958.4993 | 0.047778 | 0.130763111 |  | 0.492552 | 0.004825 | 50.28596 | 0.764702 | 0.902319648 |  | 2.18261E-10 | 6.46009E-20 | 0.73741925 | 0.049362 | 0.387279385 |  | 0.469379 | 0.245811 | 0.896287 | 0.023805 | 0.103443 |
| metabolite59 | 6.895381902 | 0.02401 | 1980.259462 | 0.505127 | 0.660276743 |  | 6.479085 | 0.07548 | 556.154 | 0.412522 | 0.679773848 |  | 26434.22721 | 1.26051E-05 | 5.54353E+13 | 0.354481 | 0.720810472 |  | 1.227199 | 0.64858 | 2.32202 | 0.530474 | 0.708331897 |
| metabolite60 | 0.000149172 | 5.86E-07 | 0.037958269 | 0.002323 | 0.013020884 |  | 66.40751 | 0.751683 | 5866.78 | 0.06916 | 0.290456173 |  | 1.75803E+12 | 977.4645349 | 3.16193E+21 | 0.010788 | 0.212626714 |  | 3.965434 | 2.180475 | 7.211581 | 1.59E-05 | 0.000853607 |
| metabolite61 | 28108.88432 | 145.2399 | 5440029.113 | 0.000226 | 0.00217138 |  | 9.929815 | 0.123292 | 799.7376 | 0.307506 | 0.587691064 |  | 0.023520859 | 1.37104E-11 | 40351047.3 | 0.730253 | 0.925034149 |  | 0.527559 | 0.283959 | 0.980136 | 0.045426 | 0.157252627 |
| metabolite62 | 0.356993126 | 0.00148 | 86.09684491 | 0.713546 | 0.828525915 |  | 0.173399 | 0.00233 | 12.90625 | 0.427251 | 0.689909047 |  | 0.001171863 | 1.06866E-12 | 1285029.958 | 0.526406 | 0.824919067 |  | 0.959313 | 0.516978 | 1.780117 | 0.895467 | 0.950808708 |
| metabolite63 | 0.355947113 | 0.001105 | 114.6833884 | 0.726571 | 0.838538656 |  | 89.04004 | 1.016379 | 7800.369 | 0.051665 | 0.254388663 |  | 5622.738329 | 1.74338E-06 | 1.81344E+13 | 0.441179 | 0.782677331 |  | 1.534311 | 0.804205 | 2.927251 | 0.196684 | 0.394046135 |
| metabolite64 | 12.04906763 | 0.039232 | 3700.577953 | 0.396165 | 0.565232162 |  | 0.189717 | 0.002078 | 17.31929 | 0.471973 | 0.718413961 |  | 5.676089627 | 1.88119E-09 | 17126371343 | 0.87639 | 0.965088312 |  | 0.761408 | 0.399389 | 1.451573 | 0.409435 | 0.617226213 |
| metabolite65 | 79.65013006 | 0.255371 | 24842.81948 | 0.137985 | 0.280889663 |  | 0.155446 | 0.001636 | 14.77138 | 0.424769 | 0.688648815 |  | 3.633660773 | 9.79125E-10 | 13484993932 | 0.908836 | 0.971397225 |  | 0.656448 | 0.34317 | 1.255718 | 0.206063 | 0.403747645 |
| metabolite66 | 0.208585943 | 0.000656 | 66.30249156 | 0.59496 | 0.735720172 |  | 11.88687 | 0.129517 | 1090.96 | 0.285346 | 0.572365249 |  | 22285151.18 | 0.008471882 | 5.86207E+16 | 0.129139 | 0.530263915 |  | 1.225591 | 0.640635 | 2.344662 | 0.540069 | 0.713461462 |
| metabolite67 | 1.68144E-05 | 9.88E-08 | 0.002862754 | 5.53E-05 | 0.000771753 |  | 470.6087 | 7.050643 | 31411.68 | 0.004898 | 0.110032926 |  | 519519513.6 | 0.546321054 | 4.94033E+17 | 0.059673 | 0.415087442 |  | 4.294587 | 2.451321 | 7.523893 | 1.45E-06 | 0.000167857 |
| metabolite68 | 2.296043739 | 0.008507 | 619.6701634 | 0.771579 | 0.867104274 |  | 19.29064 | 0.242761 | 1532.902 | 0.187621 | 0.462941033 |  | 2125.443201 | 1.28273E-06 | 3.52179E+12 | 0.480797 | 0.800476896 |  | 1.336959 | 0.713135 | 2.50648 | 0.367087 | 0.581913276 |
| metabolite69 | 0.01703305 | 6.36E-05 | 4.562409563 | 0.156144 | 0.305133943 |  | 129.5794 | 1.673664 | 10032.37 | 0.030461 | 0.202218637 |  | 5334662089 | 3.947092924 | 7.21002E+18 | 0.039086 | 0.354640645 |  | 1.631606 | 0.869932 | 3.060167 | 0.129918 | 0.30669642 |
| metabolite70 | 0.953351532 | 0.005207 | 174.538888 | 0.985694 | 0.992378901 |  | 44.54496 | 0.782873 | 2534.58 | 0.068251 | 0.288471947 |  | 248192.4992 | 0.000720008 | 8.55539E+13 | 0.218137 | 0.609109294 |  | 1.607735 | 0.900024 | 2.871936 | 0.111524 | 0.281406417 |
| metabolite71 | 0.453805309 | 0.002145 | 96.01877356 | 0.772966 | 0.867133448 |  | 11.59874 | 0.175089 | 768.3559 | 0.25444 | 0.538734937 |  | 0.113826196 | 1.65829E-10 | 78130930.76 | 0.834574 | 0.953010974 |  | 1.923475 | 1.065197 | 3.473308 | 0.032179 | 0.127855993 |
| metabolite72 | 6863.319957 | 12.49179 | 3770890.717 | 0.00707 | 0.030544967 |  | 13.7169 | 0.082753 | 2273.667 | 0.317417 | 0.596153365 |  | 10859027145 | 0.276819518 | 4.25976E+20 | 0.065992 | 0.43267573 |  | 0.591462 | 0.285653 | 1.224657 | 0.160091 | 0.347628057 |
| metabolite73 | 0.176199738 | 0.000738 | 42.04324683 | 0.535519 | 0.687888774 |  | 0.496585 | 0.006627 | 37.21347 | 0.751205 | 0.895049124 |  | 514.3717883 | 4.74578E-07 | 5.57502E+11 | 0.557613 | 0.84564791 |  | 0.90285 | 0.486916 | 1.674085 | 0.746242 | 0.861215423 |
| metabolite74 | 3.173883656 | 0.007395 | 1362.196746 | 0.709538 | 0.826440128 |  | 193.0922 | 1.80072 | 20705.39 | 0.029401 | 0.200006549 |  | 2.51636E+12 | 459.2398952 | 1.37881E+22 | 0.014039 | 0.247990281 |  | 1.028756 | 0.519509 | 2.03719 | 0.935326 | 0.967936596 |
| metabolite75 | 288.0575849 | 0.468228 | 177215.4715 | 0.086695 | 0.202221697 |  | 5.379793 | 0.032312 | 895.7201 | 0.520405 | 0.755565108 |  | 0.019543969 | 3.61422E-13 | 1056843673 | 0.755561 | 0.937628791 |  | 0.575118 | 0.278327 | 1.18839 | 0.138043 | 0.318183779 |
| metabolite76 | 0.016542213 | 4.67E-05 | 5.863165967 | 0.173612 | 0.32675075 |  | 323.989 | 3.472364 | 30229.8 | 0.013959 | 0.149726748 |  | 70684.14969 | 1.32852E-05 | 3.76076E+14 | 0.330571 | 0.705434241 |  | 2.197505 | 1.146585 | 4.211661 | 0.019407 | 0.088259713 |
| metabolite77 | 24097.81873 | 92.51505 | 6276868.866 | 0.000556 | 0.004365827 |  | 33.07054 | 0.340224 | 3214.532 | 0.136898 | 0.399707348 |  | 0.000455959 | 9.82715E-14 | 2115551.344 | 0.499534 | 0.812201491 |  | 0.528164 | 0.275582 | 1.012248 | 0.057001 | 0.182827993 |
| metabolite78 | 44.89350847 | 0.24952 | 8077.218629 | 0.153816 | 0.303517081 |  | 1.446414 | 0.023354 | 89.581 | 0.861142 | 0.947996663 |  | 6.954706398 | 1.57392E-08 | 3073085537 | 0.848909 | 0.955623187 |  | 0.5264 | 0.295293 | 0.938382 | 0.031704 | 0.126917244 |
| metabolite79 | 0.440281903 | 0.002375 | 81.63491864 | 0.75876 | 0.859511446 |  | 1.595524 | 0.026091 | 97.56844 | 0.82424 | 0.93112774 |  | 6.79247E-06 | 1.8407E-14 | 2506.527242 | 0.239594 | 0.630174307 |  | 0.884928 | 0.49148 | 1.593346 | 0.684471 | 0.82309527 |
| metabolite80 | 4.918386055 | 0.025042 | 965.9865431 | 0.555512 | 0.705994725 |  | 3.432293 | 0.053673 | 219.4879 | 0.56221 | 0.78648406 |  | 112.5576317 | 2.15713E-07 | 58731943767 | 0.645542 | 0.888249197 |  | 0.852829 | 0.470408 | 1.54614 | 0.601012 | 0.76355936 |
| metabolite81 | 0.326561095 | 0.003756 | 28.39093982 | 0.624223 | 0.761753392 |  | 0.07322 | 0.002242 | 2.3914 | 0.144442 | 0.409178104 |  | 4.91154E-05 | 2.28785E-12 | 1054.403863 | 0.251852 | 0.64285589 |  | 0.987616 | 0.596925 | 1.634017 | 0.961399 | 0.981510624 |
| metabolite82 | 113.7552395 | 0.750382 | 17244.896 | 0.067279 | 0.166447169 |  | 0.043224 | 0.000815 | 2.293711 | 0.123916 | 0.378479122 |  | 0.00838679 | 3.31984E-11 | 2118721.931 | 0.629091 | 0.879305472 |  | 0.607729 | 0.344822 | 1.071087 | 0.087767 | 0.243608612 |
| metabolite83 | 2.261993936 | 0.009012 | 567.7425361 | 0.772707 | 0.867104274 |  | 0.564419 | 0.007275 | 43.78786 | 0.797168 | 0.920823742 |  | 1.913760915 | 1.45144E-09 | 2523340431 | 0.951802 | 0.986747222 |  | 0.671257 | 0.361754 | 1.245558 | 0.20895 | 0.40640187 |
| metabolite84 | 22.11601 | 0.069567 | 7030.89061 | 0.2945 | 0.46596689 |  | 0.17764 | 0.00188 | 16.78281 | 0.458064 | 0.709676915 |  | 7.167515148 | 2.00698E-09 | 25597280373 | 0.861006 | 0.959517929 |  | 0.690237 | 0.360846 | 1.320306 | 0.265002 | 0.473355199 |
| metabolite85 | 5.565671162 | 0.017098 | 1811.740741 | 0.562042 | 0.711128398 |  | 1.536807 | 0.016047 | 147.1796 | 0.853864 | 0.944356957 |  | 224749.4747 | 6.97191E-05 | 7.24512E+14 | 0.272339 | 0.656510118 |  | 0.769747 | 0.401504 | 1.475727 | 0.432327 | 0.634387873 |
| metabolite86 | 0.285657762 | 0.001896 | 43.03740271 | 0.625321 | 0.762340658 |  | 25.72742 | 0.517195 | 1279.789 | 0.1061 | 0.353481876 |  | 5945.305996 | 3.31426E-05 | 1.0665E+12 | 0.372064 | 0.736141064 |  | 1.747025 | 1.00202 | 3.045944 | 0.051665 | 0.171647229 |
| metabolite87 | 1.216597854 | 0.004796 | 308.6298793 | 0.944786 | 0.973603329 |  | 82.09057 | 1.134481 | 5940.038 | 0.046029 | 0.241601529 |  | 9473770.37 | 0.008602165 | 1.04337E+16 | 0.133302 | 0.532435499 |  | 1.442633 | 0.776165 | 2.681376 | 0.249034 | 0.453688606 |
| metabolite88 | 4.26965E-05 | 1.86E-07 | 0.009813054 | 0.000435 | 0.003603222 |  | 154.3073 | 1.838271 | 12952.8 | 0.027803 | 0.194420599 |  | 896.3005703 | 3.02123E-07 | 2.65903E+12 | 0.542501 | 0.834668554 |  | 3.546156 | 1.939543 | 6.483599 | 7.55E-05 | 0.002437744 |
| metabolite89 | 378749.1092 | 2146.362 | 66834448.2 | 3.78E-06 | 0.000101152 |  | 21.7925 | 0.254415 | 1866.684 | 0.177482 | 0.452669541 |  | 1336.334755 | 5.51865E-07 | 3.23592E+12 | 0.515176 | 0.819395994 |  | 0.428478 | 0.229992 | 0.798259 | 0.008729 | 0.052672715 |
| metabolite90 | 43.71441777 | 0.293309 | 6515.147893 | 0.141811 | 0.285236732 |  | 0.257061 | 0.004846 | 13.63633 | 0.503952 | 0.743356023 |  | 0.000206649 | 1.01304E-12 | 42154.14112 | 0.386651 | 0.744694442 |  | 1.010865 | 0.572126 | 1.786053 | 0.970384 | 0.984337009 |
| metabolite91 | 7.63297E-06 | 9.12E-08 | 0.000639144 | 8.57E-07 | 3.83E-05 |  | 5.59913 | 0.11595 | 270.3781 | 0.385737 | 0.657589432 |  | 9.395414548 | 6.6546E-08 | 1326509130 | 0.815429 | 0.946906762 |  | 3.568077 | 2.156186 | 5.904487 | 2.67E-06 | 0.000241561 |
| metabolite92 | 0.059532778 | 0.000178 | 19.89706804 | 0.343446 | 0.516630458 |  | 171.3766 | 1.914595 | 15340.03 | 0.026866 | 0.193061777 |  | 90874377203 | 35.35129175 | 2.33603E+20 | 0.024364 | 0.296445853 |  | 1.724374 | 0.900704 | 3.301269 | 0.102926 | 0.267290785 |
| metabolite93 | 0.021333822 | 9.84E-05 | 4.623886408 | 0.1637 | 0.313949599 |  | 0.717231 | 0.010005 | 51.41694 | 0.87909 | 0.954279989 |  | 0.047084102 | 5.28858E-11 | 41918873.27 | 0.771863 | 0.940047404 |  | 1.266881 | 0.688629 | 2.330701 | 0.448525 | 0.646217836 |
| metabolite94 | 4.657986862 | 0.047706 | 454.8048155 | 0.511741 | 0.666809236 |  | 0.559784 | 0.015099 | 20.75389 | 0.753537 | 0.895699576 |  | 7.06311E-05 | 2.06411E-12 | 2416.910077 | 0.282544 | 0.665728265 |  | 1.209546 | 0.722105 | 2.026022 | 0.471278 | 0.665998256 |
| metabolite95 | 3.441064021 | 0.017069 | 693.7069032 | 0.648947 | 0.781896839 |  | 0.138327 | 0.002146 | 8.917036 | 0.354058 | 0.627941989 |  | 2.148130019 | 3.70565E-09 | 1245252073 | 0.94093 | 0.981943104 |  | 0.673152 | 0.371764 | 1.218872 | 0.194052 | 0.390948272 |
| metabolite96 | 2.405406374 | 0.024149 | 239.5969117 | 0.709204 | 0.826440128 |  | 13.38684 | 0.368258 | 486.6349 | 0.159841 | 0.427931877 |  | 267.8385187 | 6.99217E-06 | 10259680625 | 0.531609 | 0.82751665 |  | 1.021112 | 0.607925 | 1.715128 | 0.937206 | 0.968524039 |
| metabolite97 | 0.015605832 | 7.28E-05 | 3.343796924 | 0.131558 | 0.270928255 |  | 12.06127 | 0.172923 | 841.2647 | 0.252736 | 0.537479982 |  | 5.136299589 | 5.81126E-09 | 4539730994 | 0.876559 | 0.965088312 |  | 1.486528 | 0.810662 | 2.725879 | 0.202691 | 0.400040889 |
| metabolite98 | 211002.962 | 782.8085 | 56875018.91 | 3.78E-05 | 0.000587279 |  | 0.890087 | 0.007632 | 103.8066 | 0.961839 | 0.981849859 |  | 26232.77472 | 3.02691E-06 | 2.27347E+14 | 0.385357 | 0.743660185 |  | 0.446274 | 0.229737 | 0.866906 | 0.01894 | 0.087095279 |
| metabolite99 | 2993881.731 | 18172.06 | 493247624.8 | 8.90E-08 | 7.50E-06 |  | 0.558339 | 0.005763 | 54.09089 | 0.803235 | 0.922563913 |  | 34034.35551 | 9.62499E-06 | 1.20347E+14 | 0.354258 | 0.720810472 |  | 0.34043 | 0.182596 | 0.634694 | 0.000967 | 0.012336399 |
| metabolite100 | 0.040554723 | 0.000184 | 8.946954626 | 0.246877 | 0.412684897 |  | 5.248559 | 0.073818 | 373.1802 | 0.447629 | 0.70031648 |  | 0.081775068 | 9.05266E-11 | 73869542.96 | 0.81234 | 0.946496748 |  | 1.727419 | 0.945152 | 3.157138 | 0.078364 | 0.225083899 |
| metabolite101 | 0.576930863 | 0.002084 | 159.6878748 | 0.848315 | 0.916477001 |  | 16.37023 | 0.201365 | 1330.843 | 0.215465 | 0.49540891 |  | 47564.11667 | 2.74417E-05 | 8.24419E+13 | 0.323223 | 0.703078286 |  | 1.407218 | 0.749238 | 2.643033 | 0.290417 | 0.503523289 |
| metabolite102 | 0.17799242 | 0.000886 | 35.7681722 | 0.524833 | 0.676739442 |  | 1.979735 | 0.030238 | 129.6147 | 0.74948 | 0.894781821 |  | 40.60963662 | 7.04346E-08 | 23413807105 | 0.719616 | 0.920542269 |  | 1.012784 | 0.556702 | 1.842513 | 0.966888 | 0.983904973 |
| metabolite103 | 1.509305718 | 0.008552 | 266.3630158 | 0.876345 | 0.932230925 |  | 16.20029 | 0.284802 | 921.5171 | 0.1795 | 0.455229213 |  | 4.077174869 | 1.18719E-08 | 1400223726 | 0.888795 | 0.967390372 |  | 0.797469 | 0.445943 | 1.426094 | 0.447004 | 0.644706691 |
| metabolite104 | 2.853251168 | 0.014188 | 573.8119788 | 0.699164 | 0.818743542 |  | 4.144601 | 0.063984 | 268.4708 | 0.505435 | 0.743723621 |  | 0.029372839 | 5.19364E-11 | 16611930.17 | 0.732185 | 0.925814843 |  | 0.981747 | 0.539971 | 1.784963 | 0.951949 | 0.976688483 |
| metabolite105 | 213.3038247 | 0.843166 | 53961.50104 | 0.060084 | 0.153880749 |  | 0.283769 | 0.003411 | 23.60927 | 0.577698 | 0.79199206 |  | 1.84203E-05 | 1.07198E-14 | 31652.26543 | 0.317148 | 0.700308709 |  | 0.583083 | 0.312002 | 1.089691 | 0.093686 | 0.253588887 |
| metabolite106 | 0.429111757 | 0.00144 | 127.8639937 | 0.77154 | 0.867104274 |  | 59.60867 | 0.715271 | 4967.62 | 0.072767 | 0.297124077 |  | 32990.72117 | 1.42084E-05 | 7.66017E+13 | 0.346423 | 0.716996282 |  | 1.546986 | 0.818326 | 2.924466 | 0.182043 | 0.376988254 |
| metabolite107 | 315.9936315 | 1.610629 | 61995.65663 | 0.034797 | 0.10281162 |  | 11.33076 | 0.166956 | 768.9809 | 0.261697 | 0.547748746 |  | 90672.3718 | 0.000131135 | 6.26949E+13 | 0.27406 | 0.658701838 |  | 0.804523 | 0.439156 | 1.473868 | 0.482792 | 0.674056562 |
| metabolite108 | 0.032822256 | 0.000124 | 8.687963527 | 0.232533 | 0.395476628 |  | 128.4498 | 1.694755 | 9735.536 | 0.029959 | 0.200797686 |  | 1973694.027 | 0.001275064 | 3.05512E+15 | 0.18212 | 0.580004487 |  | 1.945751 | 1.046501 | 3.617719 | 0.037668 | 0.139369213 |
| metabolite109 | 1.59526E-05 | 1.08E-07 | 0.002357115 | 3.23E-05 | 0.000519494 |  | 98.37193 | 1.524619 | 6347.182 | 0.033053 | 0.210580718 |  | 174487142.9 | 0.289710524 | 1.0509E+17 | 0.068456 | 0.438759642 |  | 2.961511 | 1.668213 | 5.25745 | 0.000328 | 0.005796663 |
| metabolite110 | 12.83574379 | 0.055423 | 2972.727714 | 0.360239 | 0.53218466 |  | 1.112088 | 0.015031 | 82.27857 | 0.961499 | 0.981849859 |  | 4.700150564 | 4.51627E-09 | 4891514258 | 0.884116 | 0.966407332 |  | 1.0133 | 0.547477 | 1.875471 | 0.966524 | 0.983904973 |
| metabolite111 | 3003.824341 | 7.394804 | 1220175.797 | 0.010223 | 0.040316732 |  | 0.149139 | 0.001155 | 19.25217 | 0.444511 | 0.699260511 |  | 2.76043E-07 | 2.00789E-17 | 3795.030807 | 0.207437 | 0.599065696 |  | 0.479124 | 0.241875 | 0.949084 | 0.037119 | 0.138701319 |
| metabolite112 | 0.031431875 | 0.000128 | 7.727843721 | 0.22058 | 0.383764944 |  | 0.191394 | 0.002462 | 14.8792 | 0.458202 | 0.709676915 |  | 6130.210915 | 4.6781E-06 | 8.03306E+12 | 0.41727 | 0.770009271 |  | 1.586768 | 0.855008 | 2.944807 | 0.146164 | 0.329534576 |
| metabolite113 | 1260516.537 | 5823.173 | 272858456.2 | 1.30E-06 | 5.06E-05 |  | 0.623696 | 0.005632 | 69.06553 | 0.844521 | 0.938792681 |  | 6.141256424 | 8.40473E-10 | 44873582375 | 0.875819 | 0.965088312 |  | 0.376061 | 0.196634 | 0.719214 | 0.003804 | 0.03063876 |
| metabolite114 | 2.014720618 | 0.004411 | 920.2656862 | 0.823026 | 0.900162473 |  | 5.173148 | 0.041988 | 637.3617 | 0.504785 | 0.743356023 |  | 47.1393841 | 3.72526E-09 | 5.96501E+11 | 0.746046 | 0.933438158 |  | 1.09753 | 0.550623 | 2.187653 | 0.791931 | 0.892455404 |
| metabolite115 | 12728448.08 | 59363.84 | 2729159480 | 2.86E-08 | 3.54E-06 |  | 0.935569 | 0.007258 | 120.5942 | 0.978616 | 0.990088079 |  | 8.592607575 | 5.67829E-10 | 1.30027E+11 | 0.857596 | 0.957853167 |  | 0.313816 | 0.16207 | 0.607639 | 0.000827 | 0.011407084 |
| metabolite116 | 5.410805346 | 0.022319 | 1311.756282 | 0.547938 | 0.699397338 |  | 70.37483 | 0.996042 | 4972.296 | 0.052719 | 0.255720229 |  | 282.2229409 | 2.4417E-07 | 3.26206E+11 | 0.597184 | 0.868644224 |  | 1.31944 | 0.711678 | 2.446219 | 0.380699 | 0.594352089 |
| metabolite117 | 0.000149054 | 5.01E-07 | 0.044363737 | 0.003024 | 0.015652659 |  | 494.0916 | 5.367648 | 45481.1 | 0.008288 | 0.126652263 |  | 66829533.26 | 0.014271118 | 3.12953E+17 | 0.115596 | 0.516670981 |  | 3.453392 | 1.845612 | 6.461769 | 0.000179 | 0.00406166 |
| metabolite118 | 53698.25878 | 175.6021 | 16420659.99 | 0.000303 | 0.002732572 |  | 1.70635 | 0.014333 | 203.1418 | 0.826952 | 0.931900416 |  | 705.1404638 | 7.00363E-08 | 7.0995E+12 | 0.577902 | 0.857194036 |  | 0.485398 | 0.248261 | 0.949045 | 0.036852 | 0.138667711 |
| metabolite119 | 2159.210057 | 4.993761 | 933602.5491 | 0.014666 | 0.053672897 |  | 0.212254 | 0.001578 | 28.54159 | 0.536647 | 0.767731839 |  | 0.19557792 | 1.01022E-11 | 3786393605 | 0.892835 | 0.968237896 |  | 0.462444 | 0.232529 | 0.91969 | 0.029976 | 0.122109507 |
| metabolite120 | 5.841350984 | 0.013783 | 2475.551865 | 0.568573 | 0.715372941 |  | 18.50842 | 0.161822 | 2116.905 | 0.230066 | 0.51154783 |  | 9339112.263 | 0.001145431 | 7.61451E+16 | 0.170851 | 0.568636202 |  | 1.066057 | 0.538849 | 2.109087 | 0.854539 | 0.928878912 |
| metabolite121 | 0.039651484 | 0.000228 | 6.894283313 | 0.222644 | 0.386090472 |  | 1.073908 | 0.017989 | 64.11057 | 0.972798 | 0.987432496 |  | 50.26831422 | 1.37589E-07 | 18365557833 | 0.697708 | 0.907533437 |  | 1.342654 | 0.749961 | 2.403752 | 0.323533 | 0.537434711 |
| metabolite122 | 23.53051884 | 0.097952 | 5652.606958 | 0.261212 | 0.428161236 |  | 0.360893 | 0.004719 | 27.60105 | 0.645996 | 0.834420454 |  | 4.35372E-05 | 3.79191E-14 | 49987.77543 | 0.34749 | 0.717869012 |  | 0.73759 | 0.39742 | 1.368928 | 0.336798 | 0.549387235 |
| metabolite123 | 5145.48585 | 26.74935 | 989781.9217 | 0.001881 | 0.011130877 |  | 0.000723 | 1.18E-05 | 0.044144 | 0.000801 | 0.07423496 |  | 6.22946E-14 | 1.16962E-22 | 3.31783E-05 | 0.003694 | 0.127024611 |  | 0.371529 | 0.205775 | 0.670799 | 0.001367 | 0.015705921 |
| metabolite124 | 10.4779299 | 0.05212 | 2106.426184 | 0.387149 | 0.556106434 |  | 2.766746 | 0.042071 | 181.9537 | 0.63466 | 0.828542651 |  | 1.609206601 | 2.66956E-09 | 970028488.8 | 0.963296 | 0.988990812 |  | 0.523339 | 0.290921 | 0.941439 | 0.03281 | 0.129118001 |
| metabolite125 | 9.484600475 | 0.028484 | 3158.162587 | 0.449356 | 0.615109479 |  | 705.4822 | 8.489258 | 58627.64 | 0.004389 | 0.105752273 |  | 46796211.53 | 0.014748372 | 1.48483E+17 | 0.11644 | 0.518895872 |  | 1.308306 | 0.680262 | 2.516184 | 0.422331 | 0.625280207 |
| metabolite126 | 351.7884573 | 1.941473 | 63742.8991 | 0.029151 | 0.090676284 |  | 0.039475 | 0.000629 | 2.479311 | 0.128826 | 0.3859835 |  | 0.062137224 | 1.07232E-10 | 36006271.16 | 0.787748 | 0.942605472 |  | 0.45253 | 0.25338 | 0.808207 | 0.008492 | 0.052004978 |
| metabolite127 | 9.33457E-05 | 4.54E-07 | 0.019198698 | 0.000893 | 0.006347293 |  | 20.85457 | 0.263156 | 1652.685 | 0.176086 | 0.450616586 |  | 61464.53184 | 3.92106E-05 | 9.63487E+13 | 0.309609 | 0.693815357 |  | 2.38485 | 1.296332 | 4.387388 | 0.006126 | 0.041706949 |
| metabolite128 | 15.65760981 | 0.065265 | 3756.386047 | 0.327318 | 0.501056247 |  | 3.350903 | 0.044196 | 254.06 | 0.585083 | 0.7970792 |  | 0.396997267 | 3.29213E-10 | 478737550.9 | 0.931151 | 0.977587293 |  | 0.871864 | 0.469269 | 1.619853 | 0.665232 | 0.810198673 |
| metabolite129 | 0.001232774 | 3.81E-06 | 0.399253042 | 0.025055 | 0.080851925 |  | 330.8638 | 3.568894 | 30673.61 | 0.013496 | 0.149502273 |  | 214.8143739 | 3.84778E-08 | 1.19927E+12 | 0.640021 | 0.884587563 |  | 2.045378 | 1.064991 | 3.928267 | 0.033798 | 0.131606193 |
| metabolite130 | 2.30222E-05 | 1.12E-07 | 0.004741984 | 0.000149 | 0.001629 |  | 104.0747 | 1.286321 | 8420.565 | 0.040551 | 0.228353453 |  | 241502.75 | 0.000113395 | 5.1434E+14 | 0.260484 | 0.647529078 |  | 3.572048 | 1.970047 | 6.47676 | 5.55E-05 | 0.00206643 |
| metabolite131 | 733.2184743 | 1.369685 | 392505.8601 | 0.041919 | 0.119295292 |  | 0.032084 | 0.000216 | 4.762443 | 0.180338 | 0.455572273 |  | 1112.69797 | 3.15564E-08 | 3.92344E+13 | 0.572463 | 0.853863319 |  | 0.431691 | 0.213528 | 0.872751 | 0.021127 | 0.093894345 |
| metabolite132 | 0.003976065 | 1.73E-05 | 0.913996053 | 0.04878 | 0.13290961 |  | 8.968711 | 0.117096 | 686.94 | 0.32381 | 0.599929612 |  | 334.4613759 | 2.54464E-07 | 4.39609E+11 | 0.588501 | 0.865026277 |  | 2.176883 | 1.187186 | 3.991642 | 0.013347 | 0.069176316 |
| metabolite133 | 18041025.39 | 145192.6 | 2241701714 | 5.77E-10 | 2.14E-07 |  | 0.013367 | 0.000157 | 1.139926 | 0.059728 | 0.266303606 |  | 2.22201E-05 | 8.31823E-15 | 59355.27137 | 0.335396 | 0.710418618 |  | 0.206045 | 0.115877 | 0.366377 | 4.20E-07 | 8.65E-05 |
| metabolite134 | 0.980091387 | 0.003767 | 255.0059315 | 0.994358 | 0.99677543 |  | 0.859303 | 0.010772 | 68.55093 | 0.946014 | 0.977627498 |  | 6.99788E-07 | 5.50216E-16 | 890.0203107 | 0.187874 | 0.586870392 |  | 1.013585 | 0.541757 | 1.896338 | 0.9664 | 0.983904973 |
| metabolite135 | 5.98722E-05 | 4.3E-07 | 0.008328113 | 0.00019 | 0.00193489 |  | 1.742617 | 0.027811 | 109.1914 | 0.792975 | 0.919028507 |  | 537.3706041 | 1.18171E-06 | 2.44364E+11 | 0.537778 | 0.831192316 |  | 2.279412 | 1.286647 | 4.038185 | 0.005627 | 0.03984845 |
| metabolite136 | 123.4092735 | 0.627647 | 24265.00343 | 0.076644 | 0.183501072 |  | 37.80123 | 0.587832 | 2430.851 | 0.09008 | 0.326006724 |  | 16.70954316 | 2.44028E-08 | 11441682868 | 0.78667 | 0.942360455 |  | 0.774707 | 0.424527 | 1.413741 | 0.407313 | 0.615949425 |
| metabolite137 | 66930.28774 | 378.2818 | 11842131.66 | 5.25E-05 | 0.00074125 |  | 0.12006 | 0.001518 | 9.498611 | 0.343902 | 0.618322999 |  | 0.168084224 | 1.0737E-10 | 263129568 | 0.869171 | 0.962305681 |  | 0.321461 | 0.17798 | 0.580613 | 0.000271 | 0.005202601 |
| metabolite138 | 321322.4249 | 2152.291 | 47971252.84 | 2.51E-06 | 8.17E-05 |  | 0.019843 | 0.00027 | 1.456843 | 0.07645 | 0.30475391 |  | 0.000275829 | 2.15737E-13 | 352659.315 | 0.445263 | 0.784097818 |  | 0.247028 | 0.140195 | 0.43527 | 4.26E-06 | 0.000336994 |
| metabolite139 | 0.000224424 | 1.24E-06 | 0.040585169 | 0.001981 | 0.011649241 |  | 0.172663 | 0.002435 | 12.2434 | 0.420905 | 0.685629403 |  | 0.028760889 | 3.22153E-11 | 25676855.29 | 0.736388 | 0.92772069 |  | 1.814034 | 0.994332 | 3.309478 | 0.054736 | 0.178808358 |
| metabolite140 | 726.277534 | 3.343811 | 157747.8723 | 0.018073 | 0.062682393 |  | 0.060603 | 0.000811 | 4.530379 | 0.205458 | 0.484097203 |  | 9.25772E-06 | 8.11311E-15 | 10563.81497 | 0.278404 | 0.662280984 |  | 0.441994 | 0.241899 | 0.807605 | 0.009102 | 0.054036995 |
| metabolite141 | 178.5046034 | 0.478154 | 66639.36033 | 0.088985 | 0.206259663 |  | 1.983182 | 0.017622 | 223.1908 | 0.776843 | 0.909927034 |  | 0.015752522 | 2.00646E-12 | 123671799.5 | 0.721716 | 0.922275831 |  | 0.681827 | 0.348155 | 1.335288 | 0.266486 | 0.47476173 |
| metabolite142 | 0.003330574 | 2.03E-05 | 0.546926723 | 0.030478 | 0.093474932 |  | 0.48261 | 0.007993 | 29.14021 | 0.728331 | 0.883101408 |  | 0.000692514 | 1.83458E-12 | 261409.1993 | 0.471795 | 0.798982501 |  | 1.443655 | 0.805951 | 2.585938 | 0.219582 | 0.416989176 |
| metabolite143 | 0.052954989 | 0.000234 | 11.96322961 | 0.2903 | 0.462181116 |  | 42.72559 | 0.620224 | 2943.254 | 0.084843 | 0.318034052 |  | 0.269462382 | 2.77111E-10 | 262024449.6 | 0.901387 | 0.969275008 |  | 1.508307 | 0.820491 | 2.77272 | 0.188533 | 0.383267648 |
| metabolite144 | 0.001769368 | 7.84E-06 | 0.399473206 | 0.0238 | 0.077748229 |  | 0.229538 | 0.002937 | 17.94146 | 0.509493 | 0.747914811 |  | 4.78366E-08 | 4.27031E-17 | 53.58719634 | 0.115698 | 0.516670981 |  | 2.265104 | 1.235763 | 4.151846 | 0.00936 | 0.054787206 |
| metabolite145 | 0.567948845 | 0.001563 | 206.354418 | 0.851154 | 0.917673295 |  | 2.554859 | 0.024681 | 264.4635 | 0.692683 | 0.865071815 |  | 2.927948779 | 5.47372E-10 | 15661894721 | 0.925278 | 0.976872592 |  | 1.246243 | 0.642263 | 2.418202 | 0.51647 | 0.698222914 |
| metabolite146 | 5.40332E-05 | 3.43E-07 | 0.008516533 | 0.000232 | 0.002210662 |  | 1.519185 | 0.021977 | 105.0137 | 0.846926 | 0.940012856 |  | 43.76344098 | 5.87694E-08 | 32589028445 | 0.717626 | 0.920092212 |  | 1.845873 | 1.017908 | 3.347305 | 0.045956 | 0.157762563 |
| metabolite147 | 32196.81279 | 156.5086 | 6623499.873 | 0.000221 | 0.002134392 |  | 1.284181 | 0.01483 | 111.1998 | 0.912696 | 0.968825497 |  | 0.043774969 | 1.9786E-11 | 96848611.82 | 0.776183 | 0.940047404 |  | 0.429697 | 0.231491 | 0.797609 | 0.008565 | 0.052193328 |
| metabolite148 | 28307.67187 | 117.6461 | 6811314.598 | 0.000382 | 0.003267411 |  | 2.807431 | 0.029156 | 270.3318 | 0.658644 | 0.84458436 |  | 0.009840445 | 2.63993E-12 | 36680679.42 | 0.681877 | 0.903557902 |  | 0.383078 | 0.204212 | 0.718612 | 0.003441 | 0.028699648 |
| metabolite149 | 0.001455486 | 5.51E-06 | 0.384462906 | 0.023555 | 0.077356152 |  | 61.8942 | 0.738731 | 5185.772 | 0.070543 | 0.292171646 |  | 224.7792783 | 8.80818E-08 | 5.73623E+11 | 0.625095 | 0.879305472 |  | 2.884852 | 1.564479 | 5.319581 | 0.000957 | 0.012294172 |
| metabolite150 | 0.004256738 | 2.19E-05 | 0.828410602 | 0.044748 | 0.124889502 |  | 0.368574 | 0.005402 | 25.14813 | 0.644084 | 0.833376947 |  | 63.5997626 | 8.9707E-08 | 45090468975 | 0.69038 | 0.907533437 |  | 1.114612 | 0.609082 | 2.039728 | 0.72556 | 0.849619451 |
| metabolite151 | 29.02096354 | 0.100455 | 8384.043403 | 0.246492 | 0.412226696 |  | 75.10566 | 0.907072 | 6218.759 | 0.057845 | 0.264422304 |  | 596521.7779 | 0.000269993 | 1.31796E+15 | 0.228291 | 0.62110655 |  | 1.106945 | 0.582612 | 2.103163 | 0.756936 | 0.867423249 |
| metabolite152 | 0.006960142 | 3.59E-05 | 1.350293212 | 0.067227 | 0.166429483 |  | 0.285796 | 0.004264 | 19.15551 | 0.56055 | 0.785873824 |  | 9.144424668 | 1.37524E-08 | 6080434409 | 0.831311 | 0.952506813 |  | 0.990698 | 0.542386 | 1.809567 | 0.975799 | 0.986163292 |
| metabolite153 | 0.738835829 | 0.001628 | 335.339352 | 0.922924 | 0.959644668 |  | 50.35405 | 0.430377 | 5891.415 | 0.109584 | 0.355528671 |  | 14.74656446 | 1.19224E-09 | 1.82398E+11 | 0.820866 | 0.948096664 |  | 1.368886 | 0.688897 | 2.72007 | 0.372048 | 0.587518605 |
| metabolite154 | 432582.8664 | 657.6097 | 284557733.2 | 0.000154 | 0.001643043 |  | 0.063882 | 0.000281 | 14.54272 | 0.322727 | 0.599929612 |  | 6.29867E-07 | 2.71683E-18 | 146027.7772 | 0.287228 | 0.670028181 |  | 0.288796 | 0.137123 | 0.608239 | 0.001441 | 0.016199737 |
| metabolite155 | 12097.67068 | 74.9875 | 1951707.128 | 0.000438 | 0.003622594 |  | 0.102972 | 0.001526 | 6.949939 | 0.292424 | 0.575085256 |  | 4.80342E-05 | 7.04637E-14 | 32744.23463 | 0.340055 | 0.713874057 |  | 0.309724 | 0.176049 | 0.544898 | 8.95E-05 | 0.002574089 |
| metabolite156 | 49.3805268 | 0.173178 | 14080.5206 | 0.179108 | 0.333011153 |  | 57.35239 | 0.687258 | 4786.118 | 0.07556 | 0.302959698 |  | 3995.60305 | 1.66612E-06 | 9.58203E+12 | 0.453296 | 0.789385467 |  | 0.889834 | 0.468449 | 1.690268 | 0.722097 | 0.847202474 |
| metabolite157 | 4929.951957 | 23.37194 | 1039897.53 | 0.002347 | 0.013134583 |  | 1.427357 | 0.017633 | 115.5413 | 0.874172 | 0.953453472 |  | 0.01362851 | 8.57836E-12 | 21651713.36 | 0.691837 | 0.907533437 |  | 0.531943 | 0.286868 | 0.98639 | 0.04756 | 0.161626934 |
| metabolite158 | 49.56083937 | 0.182064 | 13491.2487 | 0.175166 | 0.328635539 |  | 11.04244 | 0.131659 | 926.1468 | 0.290182 | 0.573923383 |  | 167.3487503 | 8.03166E-08 | 3.4869E+11 | 0.640925 | 0.884847869 |  | 0.814649 | 0.431427 | 1.538274 | 0.528626 | 0.706996813 |
| metabolite159 | 411264.3547 | 2414.469 | 70052004.03 | 2.88E-06 | 8.84E-05 |  | 0.167712 | 0.00195 | 14.42702 | 0.433769 | 0.694237021 |  | 2.62549E-07 | 1.37961E-16 | 499.6498123 | 0.167313 | 0.568636202 |  | 0.197877 | 0.112642 | 0.347607 | 1.34E-07 | 5.52E-05 |
| metabolite160 | 6206960.796 | 46516.35 | 828232714.3 | 7.29E-09 | 1.59E-06 |  | 0.368796 | 0.004182 | 32.52055 | 0.663342 | 0.84717161 |  | 8.09945E-05 | 3.51098E-14 | 186845.3959 | 0.393567 | 0.753237018 |  | 0.219156 | 0.123224 | 0.389772 | 1.06E-06 | 0.00012656 |
| metabolite161 | 70160.79984 | 410.4884 | 11991903.78 | 4.40E-05 | 0.00065856 |  | 0.004302 | 6.16E-05 | 0.300616 | 0.013345 | 0.148274315 |  | 2.94302E-09 | 2.87928E-18 | 3.008171188 | 0.066114 | 0.432685867 |  | 0.247023 | 0.140009 | 0.435832 | 4.46E-06 | 0.000337742 |
| metabolite162 | 0.107340696 | 0.00057 | 20.2079193 | 0.405442 | 0.574387393 |  | 0.291879 | 0.004689 | 18.1683 | 0.56024 | 0.785873824 |  | 3.02356E-08 | 8.40913E-17 | 10.87140245 | 0.087745 | 0.475490674 |  | 0.77188 | 0.427927 | 1.392288 | 0.391453 | 0.604959788 |
| metabolite163 | 517837.6741 | 2960.617 | 90574326.79 | 2.22E-06 | 7.47E-05 |  | 0.091327 | 0.001037 | 8.04345 | 0.297144 | 0.579217354 |  | 3.46898E-09 | 1.74942E-18 | 6.87874086 | 0.077247 | 0.45805738 |  | 0.209328 | 0.117879 | 0.371722 | 5.04E-07 | 8.91E-05 |
| metabolite164 | 256659.7874 | 1110.425 | 59323463.19 | 1.78E-05 | 0.000323126 |  | 0.014306 | 0.000145 | 1.410204 | 0.072496 | 0.297124077 |  | 7.065E-10 | 1.72966E-19 | 2.885782782 | 0.064662 | 0.426996029 |  | 0.195086 | 0.107824 | 0.352969 | 3.79E-07 | 8.65E-05 |
| metabolite165 | 7278504.245 | 50356.5 | 1052031501 | 8.72E-09 | 1.80E-06 |  | 0.060239 | 0.000657 | 5.527302 | 0.225623 | 0.506832586 |  | 1.16548E-05 | 3.78879E-15 | 35851.43756 | 0.31035 | 0.694400821 |  | 0.1782 | 0.101167 | 0.313889 | 2.88E-08 | 2.59E-05 |
| metabolite166 | 30155.41261 | 153.0104 | 5943054.179 | 0.000216 | 0.002111186 |  | 0.474171 | 0.005682 | 39.56949 | 0.741592 | 0.890859072 |  | 0.003387323 | 1.84653E-12 | 6213808.995 | 0.602266 | 0.872650335 |  | 0.367962 | 0.200886 | 0.673994 | 0.00159 | 0.017147509 |
| metabolite167 | 601453.2896 | 4234.221 | 85433908.18 | 7.00E-07 | 3.42E-05 |  | 0.029723 | 0.000398 | 2.219631 | 0.112959 | 0.361352629 |  | 9.31028E-08 | 8.35853E-17 | 103.7041564 | 0.130533 | 0.530263915 |  | 0.214577 | 0.123249 | 0.37358 | 3.20E-07 | 8.65E-05 |
| metabolite168 | 21929796.26 | 147738.9 | 3255174770 | 1.29E-09 | 3.68E-07 |  | 0.032418 | 0.000323 | 3.248751 | 0.147457 | 0.412408021 |  | 5.55183E-06 | 1.11573E-15 | 27625.7052 | 0.290409 | 0.673147458 |  | 0.280873 | 0.150794 | 0.523162 | 0.000114 | 0.00305052 |
| metabolite169 | 23332480.26 | 226935.9 | 2398936083 | 8.53E-11 | 6.33E-08 |  | 0.218975 | 0.002674 | 17.92938 | 0.500604 | 0.741149898 |  | 0.004978617 | 2.86789E-12 | 8642809.594 | 0.626148 | 0.879305472 |  | 0.181839 | 0.105329 | 0.313925 | 1.45E-08 | 2.59E-05 |
| metabolite170 | 15987.3462 | 67.02862 | 3813225.678 | 0.000753 | 0.005546763 |  | 19.47143 | 0.215378 | 1760.33 | 0.199073 | 0.477435637 |  | 36856067.26 | 0.014537694 | 9.34378E+16 | 0.117636 | 0.519645256 |  | 0.561011 | 0.295765 | 1.064134 | 0.079526 | 0.227365653 |
| metabolite171 | 307574.6487 | 1713.928 | 55196120.74 | 5.59E-06 | 0.000139245 |  | 0.174221 | 0.001985 | 15.29104 | 0.445653 | 0.699418725 |  | 5.64737E-05 | 2.42307E-14 | 131621.1687 | 0.376 | 0.736490425 |  | 0.287015 | 0.157788 | 0.52208 | 8.22E-05 | 0.002563907 |
| metabolite172 | 1315003.894 | 7877.295 | 219521465.2 | 3.90E-07 | 2.19E-05 |  | 0.641024 | 0.006932 | 59.27642 | 0.847675 | 0.940012856 |  | 2.052625851 | 6.69079E-10 | 6297122325 | 0.948669 | 0.986298878 |  | 0.386047 | 0.207053 | 0.719777 | 0.00339 | 0.028461643 |
| metabolite173 | 178285.3465 | 1021.235 | 31124723.2 | 1.17E-05 | 0.000233636 |  | 0.068569 | 0.000837 | 5.619252 | 0.235756 | 0.515872016 |  | 1.89105E-08 | 1.25093E-17 | 28.58723772 | 0.101959 | 0.49665678 |  | 0.218385 | 0.123795 | 0.38525 | 7.28E-07 | 0.000107901 |
| metabolite174 | 78.52982415 | 0.533604 | 11557.12479 | 0.089436 | 0.206918822 |  | 7.424656 | 0.140869 | 391.3256 | 0.323796 | 0.599929612 |  | 0.000319447 | 1.53324E-12 | 66556.35487 | 0.411931 | 0.765870595 |  | 0.743068 | 0.421506 | 1.309945 | 0.306822 | 0.520153696 |
| metabolite175 | 763.0507512 | 3.694138 | 157613.6078 | 0.016249 | 0.058068109 |  | 0.039447 | 0.000553 | 2.811491 | 0.140347 | 0.402493523 |  | 7.0535E-06 | 7.43405E-15 | 6692.436822 | 0.263119 | 0.64884445 |  | 0.345874 | 0.192914 | 0.620115 | 0.000539 | 0.00827437 |
| metabolite176 | 1661.619728 | 6.805523 | 405697.0078 | 0.009388 | 0.037810894 |  | 0.184603 | 0.002151 | 15.84445 | 0.458591 | 0.709967803 |  | 5.12974E-10 | 3.30138E-19 | 0.797067667 | 0.050064 | 0.387346738 |  | 0.358482 | 0.194922 | 0.659289 | 0.0013 | 0.015026011 |
| metabolite177 | 1806748.954 | 9836.687 | 331853789.3 | 3.56E-07 | 2.10E-05 |  | 0.069678 | 0.000708 | 6.857212 | 0.257688 | 0.542416607 |  | 0.000389351 | 8.69682E-14 | 1743097.574 | 0.490094 | 0.806374128 |  | 0.329055 | 0.175768 | 0.616022 | 0.000731 | 0.010360534 |
| metabolite178 | 23.95876821 | 0.123833 | 4635.453774 | 0.239566 | 0.40373801 |  | 1.529555 | 0.023604 | 99.11526 | 0.842091 | 0.938160513 |  | 0.622998723 | 1.12893E-09 | 343801690.3 | 0.963331 | 0.988990812 |  | 0.648512 | 0.359011 | 1.171462 | 0.153968 | 0.33849225 |
| metabolite179 | 3577.502322 | 15.32505 | 835137.5558 | 0.003981 | 0.019283204 |  | 0.012621 | 0.000158 | 1.010896 | 0.053079 | 0.255757554 |  | 1.20033E-07 | 6.71849E-17 | 214.4504702 | 0.145443 | 0.546460871 |  | 0.255603 | 0.142394 | 0.45882 | 1.27E-05 | 0.000739244 |
| metabolite180 | 173382.8934 | 1165.282 | 25797725.34 | 6.75E-06 | 0.000158503 |  | 1.239306 | 0.016528 | 92.92812 | 0.922581 | 0.972917864 |  | 1.02637581 | 9.22001E-10 | 1142566198 | 0.99805 | 0.999851958 |  | 0.315537 | 0.176826 | 0.56306 | 0.000163 | 0.003873072 |
| metabolite181 | 471.5460245 | 2.669426 | 83297.18034 | 0.021508 | 0.071841748 |  | 1.703096 | 0.026269 | 110.4153 | 0.80293 | 0.922563913 |  | 4.9023E-07 | 1.06099E-15 | 226.5101962 | 0.15631 | 0.560993738 |  | 0.573004 | 0.318311 | 1.031487 | 0.066014 | 0.201049447 |
| metabolite182 | 35709.5645 | 282.5766 | 4512663.378 | 4.54E-05 | 0.000671193 |  | 2.476675 | 0.040841 | 150.1887 | 0.665837 | 0.848467311 |  | 0.016362549 | 4.08937E-11 | 6547045.169 | 0.684812 | 0.903557902 |  | 0.405658 | 0.230973 | 0.71246 | 0.002166 | 0.02143789 |
| metabolite183 | 0.029969116 | 5.27E-05 | 17.04559939 | 0.280815 | 0.451518939 |  | 79.09844 | 0.557358 | 11225.39 | 0.086628 | 0.321084001 |  | 36713.10661 | 1.19494E-06 | 1.12797E+15 | 0.395431 | 0.755202238 |  | 2.039254 | 1.006644 | 4.131107 | 0.050363 | 0.168717728 |
| metabolite184 | 35087.19464 | 234.1395 | 5258023.651 | 8.06E-05 | 0.001024018 |  | 0.003387 | 5.63E-05 | 0.203943 | 0.00757 | 0.125141666 |  | 1.17113E-13 | 3.41201E-22 | 4.01972E-05 | 0.003657 | 0.127024611 |  | 0.227547 | 0.132717 | 0.390136 | 4.15E-07 | 8.65E-05 |
| metabolite185 | 0.712374914 | 0.004715 | 107.6268927 | 0.894848 | 0.943611806 |  | 2.762328 | 0.053345 | 143.0404 | 0.614872 | 0.817552529 |  | 0.008737392 | 4.68078E-11 | 1630967.316 | 0.626631 | 0.879305472 |  | 1.16252 | 0.661022 | 2.044488 | 0.602148 | 0.764217798 |
| metabolite186 | 619.4529895 | 3.155391 | 121608.3937 | 0.018697 | 0.06441966 |  | 2.482061 | 0.035071 | 175.6627 | 0.676523 | 0.853357187 |  | 8.104426893 | 9.51061E-09 | 6906152332 | 0.842284 | 0.95448538 |  | 0.545786 | 0.29975 | 0.993772 | 0.050129 | 0.168351763 |
| metabolite187 | 92132.44485 | 666.5351 | 12735094.38 | 1.40E-05 | 0.000272604 |  | 0.004042 | 6.69E-05 | 0.244085 | 0.009642 | 0.13042689 |  | 8.94235E-08 | 1.55552E-16 | 51.40756939 | 0.117607 | 0.519645256 |  | 0.227626 | 0.13287 | 0.389956 | 4.03E-07 | 8.65E-05 |
| metabolite188 | 69.86936928 | 0.365046 | 13372.92464 | 0.116018 | 0.248066585 |  | 20.15908 | 0.318894 | 1274.369 | 0.158476 | 0.425238556 |  | 188.5307734 | 3.30071E-07 | 1.07686E+11 | 0.611559 | 0.875638024 |  | 0.542972 | 0.301701 | 0.977188 | 0.044032 | 0.154154495 |
| metabolite189 | 57707.13108 | 408.5124 | 8151803.622 | 3.15E-05 | 0.000508551 |  | 0.003211 | 5.44E-05 | 0.189676 | 0.006785 | 0.122236677 |  | 3.96442E-09 | 8.00095E-18 | 1.964343159 | 0.060841 | 0.417148502 |  | 0.251648 | 0.145805 | 0.434324 | 2.61E-06 | 0.000241561 |
| metabolite190 | 1453.613328 | 5.902327 | 357992.9944 | 0.010827 | 0.042029023 |  | 0.632436 | 0.00728 | 54.94546 | 0.840952 | 0.938160513 |  | 0.544073256 | 2.39536E-10 | 1235789787 | 0.955939 | 0.9871034 |  | 0.503831 | 0.269444 | 0.942109 | 0.03399 | 0.131606193 |
| metabolite191 | 8762.673426 | 33.01943 | 2325432.389 | 0.001861 | 0.011049245 |  | 0.08753 | 0.000907 | 8.44421 | 0.298369 | 0.579650755 |  | 0.122115448 | 2.93173E-11 | 508647331.6 | 0.852729 | 0.956407596 |  | 0.27179 | 0.147608 | 0.500444 | 5.78E-05 | 0.002082797 |
| metabolite192 | 940.0834329 | 3.894624 | 226917.0932 | 0.016028 | 0.057522384 |  | 1.111384 | 0.013175 | 93.75278 | 0.962859 | 0.981849859 |  | 1.04145E-05 | 5.90075E-15 | 18380.94624 | 0.293218 | 0.676990344 |  | 0.397766 | 0.215955 | 0.732645 | 0.003781 | 0.03063876 |
| metabolite193 | 7.696817739 | 0.063845 | 927.8829311 | 0.405678 | 0.574387393 |  | 2.109689 | 0.048014 | 92.69702 | 0.699638 | 0.869072177 |  | 8.39263E-06 | 1.11868E-13 | 629.6388606 | 0.209109 | 0.602019678 |  | 0.761646 | 0.444248 | 1.305814 | 0.324376 | 0.537872071 |
| metabolite194 | 42596.99163 | 183.1823 | 9905454.738 | 0.000209 | 0.002076971 |  | 0.800854 | 0.008329 | 77.00623 | 0.924225 | 0.97342373 |  | 1.95886E-05 | 5.81447E-15 | 65993.09111 | 0.334885 | 0.710418618 |  | 0.306693 | 0.165804 | 0.5673 | 0.000267 | 0.005181725 |
| metabolite195 | 10857.46594 | 79.68758 | 1479334.223 | 0.00033 | 0.002909079 |  | 0.309717 | 0.005151 | 18.62405 | 0.57607 | 0.791336134 |  | 1.27983E-08 | 4.34161E-17 | 3.772731871 | 0.070455 | 0.446172776 |  | 0.341169 | 0.19651 | 0.592317 | 0.00022 | 0.004532241 |
| metabolite196 | 7952.84979 | 58.30268 | 1084818.406 | 0.000509 | 0.004063341 |  | 1.604246 | 0.026925 | 95.58358 | 0.821112 | 0.928972215 |  | 17.40024839 | 4.75634E-08 | 6365585981 | 0.776983 | 0.940047404 |  | 0.390048 | 0.223253 | 0.681457 | 0.00127 | 0.014776786 |
| metabolite197 | 377530.6166 | 2407.546 | 59201097 | 2.36E-06 | 7.82E-05 |  | 0.449153 | 0.005513 | 36.59062 | 0.722126 | 0.878337862 |  | 0.18042153 | 1.07681E-10 | 302300009.2 | 0.874722 | 0.965088312 |  | 0.337399 | 0.185824 | 0.612612 | 0.000528 | 0.008171057 |
| metabolite198 | 214.9613914 | 0.973912 | 47446.19108 | 0.05365 | 0.14221014 |  | 0.130082 | 0.001756 | 9.6378 | 0.355146 | 0.628491529 |  | 1.42908E-06 | 1.46065E-15 | 1398.202306 | 0.205239 | 0.597241307 |  | 0.636046 | 0.344741 | 1.173504 | 0.150436 | 0.33409124 |
| metabolite199 | 41764.48638 | 345.0557 | 5055046.395 | 3.06E-05 | 0.000500063 |  | 0.074566 | 0.00129 | 4.310006 | 0.212404 | 0.490298887 |  | 6.70009E-10 | 2.73558E-18 | 0.164101328 | 0.034266 | 0.336462533 |  | 0.327998 | 0.189921 | 0.566461 | 0.000115 | 0.00305052 |
| metabolite200 | 167.9959227 | 0.545673 | 51720.795 | 0.082399 | 0.19457315 |  | 35.90839 | 0.389038 | 3314.36 | 0.12373 | 0.378275375 |  | 17.10937305 | 4.48984E-09 | 65198452747 | 0.801288 | 0.9460629 |  | 0.76451 | 0.398172 | 1.467898 | 0.421523 | 0.625020256 |
| metabolite201 | 5914.270873 | 35.24094 | 992555.7002 | 0.001207 | 0.008013947 |  | 0.005397 | 8.79E-05 | 0.33118 | 0.014404 | 0.149726748 |  | 3.47049E-08 | 6.11778E-17 | 19.68735464 | 0.097692 | 0.492678215 |  | 0.257394 | 0.148522 | 0.446071 | 4.27E-06 | 0.000336994 |
| metabolite202 | 32268.50195 | 193.4894 | 5381464.742 | 0.000125 | 0.001450706 |  | 0.010721 | 0.000157 | 0.731666 | 0.037547 | 0.220921127 |  | 4.0629E-08 | 4.9033E-17 | 33.66538921 | 0.107134 | 0.506823236 |  | 0.315204 | 0.176946 | 0.56149 | 0.000154 | 0.003809609 |
| metabolite203 | 5778.074936 | 32.42617 | 1029605.241 | 0.001407 | 0.00900185 |  | 0.191914 | 0.002702 | 13.62869 | 0.449481 | 0.702031003 |  | 8.69259E-06 | 1.07997E-14 | 6996.605735 | 0.267768 | 0.653630337 |  | 0.381044 | 0.212405 | 0.683575 | 0.001599 | 0.017197377 |
| metabolite204 | 10.46545776 | 0.029417 | 3723.277212 | 0.435028 | 0.602385306 |  | 2.716235 | 0.026371 | 279.771 | 0.673422 | 0.851700797 |  | 0.0381128 | 7.33655E-12 | 197993128.5 | 0.775219 | 0.940047404 |  | 0.504175 | 0.262897 | 0.966888 | 0.041605 | 0.148490198 |
| metabolite205 | 48.65617992 | 0.169759 | 13945.78529 | 0.181142 | 0.335852162 |  | 0.880064 | 0.009859 | 78.56027 | 0.955641 | 0.980733594 |  | 0.43516481 | 1.68751E-10 | 1122177465 | 0.940149 | 0.981519724 |  | 0.646735 | 0.341915 | 1.223308 | 0.182922 | 0.377980424 |
| metabolite206 | 0.02255426 | 8.22E-05 | 6.192160784 | 0.188366 | 0.344178367 |  | 0.262726 | 0.00307 | 22.48322 | 0.557191 | 0.783826463 |  | 3062457.927 | 0.001685574 | 5.56407E+15 | 0.172532 | 0.571154682 |  | 1.287754 | 0.681936 | 2.43177 | 0.437215 | 0.637923084 |
| metabolite207 | 47553.93402 | 273.1697 | 8278285.636 | 8.16E-05 | 0.001033713 |  | 0.101248 | 0.001323 | 7.747385 | 0.302982 | 0.583677462 |  | 5.28187E-11 | 6.20543E-20 | 0.044957536 | 0.02605 | 0.300224208 |  | 0.266804 | 0.15044 | 0.473175 | 1.56E-05 | 0.00084871 |
| metabolite208 | 134.5433809 | 0.265516 | 68176.47815 | 0.125757 | 0.262772889 |  | 1.951017 | 0.013752 | 276.7945 | 0.79198 | 0.919002513 |  | 414.05059 | 1.74951E-08 | 9.79917E+12 | 0.62197 | 0.879305472 |  | 0.621921 | 0.307767 | 1.256752 | 0.188464 | 0.383267648 |
| metabolite209 | 7.492861642 | 0.033781 | 1661.979466 | 0.466475 | 0.629945116 |  | 2.60654 | 0.036806 | 184.5932 | 0.660239 | 0.845752706 |  | 3.64184E-08 | 5.43107E-17 | 24.4205775 | 0.101395 | 0.49665678 |  | 1.051535 | 0.571438 | 1.93499 | 0.871991 | 0.939047556 |
| metabolite210 | 727.2125536 | 3.489398 | 151555.7024 | 0.0172 | 0.060444102 |  | 0.073021 | 0.001004 | 5.308869 | 0.23399 | 0.515235933 |  | 2.50585E-11 | 3.78022E-20 | 0.016610813 | 0.020258 | 0.279023995 |  | 0.456629 | 0.250701 | 0.831705 | 0.011736 | 0.063765457 |
| metabolite211 | 565459.7921 | 4401.315 | 72647561.57 | 4.85E-07 | 2.61E-05 |  | 0.989867 | 0.013596 | 72.06744 | 0.996294 | 0.999037854 |  | 0.010519884 | 1.10955E-11 | 9974105.537 | 0.666672 | 0.899970769 |  | 0.333311 | 0.18694 | 0.594287 | 0.00031 | 0.005676228 |
| metabolite212 | 32979.0311 | 143.786 | 7564135.794 | 0.000281 | 0.002550684 |  | 2.198064 | 0.02344 | 206.123 | 0.734541 | 0.887227321 |  | 80.31928924 | 2.46441E-08 | 2.61774E+11 | 0.695476 | 0.907533437 |  | 0.433846 | 0.23082 | 0.815447 | 0.010776 | 0.060040448 |
| metabolite213 | 20021.78867 | 126.4605 | 3169937.466 | 0.00021 | 0.002076971 |  | 0.558304 | 0.008024 | 38.84732 | 0.78822 | 0.916953836 |  | 4.25787E-07 | 6.54842E-16 | 276.8520368 | 0.159327 | 0.563107638 |  | 0.331386 | 0.187176 | 0.586703 | 0.000246 | 0.004941425 |
| metabolite214 | 545452.4881 | 3752.188 | 79291990.09 | 9.20E-07 | 3.92E-05 |  | 0.148039 | 0.001896 | 11.56148 | 0.392109 | 0.664133233 |  | 0.00111354 | 7.96993E-13 | 1555812.899 | 0.52807 | 0.824919067 |  | 0.398075 | 0.218136 | 0.726443 | 0.00332 | 0.028065041 |
| metabolite215 | 2700881.603 | 19466.54 | 374733385.6 | 4.31E-08 | 4.70E-06 |  | 0.367377 | 0.004312 | 31.30243 | 0.65968 | 0.845620877 |  | 0.110751479 | 5.29565E-11 | 231621866.7 | 0.841094 | 0.954234322 |  | 0.290911 | 0.160697 | 0.526641 | 8.58E-05 | 0.0025692 |
| metabolite216 | 10.35241081 | 0.026877 | 3987.561446 | 0.443274 | 0.610763127 |  | 0.796328 | 0.007239 | 87.60003 | 0.924515 | 0.97342373 |  | 1.06813E-05 | 1.6692E-15 | 68350.46131 | 0.322552 | 0.702459439 |  | 0.720295 | 0.368715 | 1.407117 | 0.338983 | 0.550772699 |
| metabolite217 | 4643.527504 | 21.33721 | 1010551.359 | 0.002655 | 0.014322114 |  | 0.914141 | 0.011052 | 75.61266 | 0.968285 | 0.98473715 |  | 2.31063E-05 | 1.41962E-14 | 37608.53377 | 0.326039 | 0.704745603 |  | 0.451587 | 0.24438 | 0.834483 | 0.012551 | 0.066545486 |
| metabolite218 | 0.027097995 | 0.000122 | 6.04160645 | 0.193579 | 0.350254087 |  | 0.714987 | 0.0098 | 52.16624 | 0.878455 | 0.954279989 |  | 0.564380357 | 5.77901E-10 | 551175857.6 | 0.956902 | 0.9871034 |  | 1.219995 | 0.661164 | 2.251163 | 0.525952 | 0.705643323 |
| metabolite219 | 9.394067562 | 0.045882 | 1923.381215 | 0.41113 | 0.579454942 |  | 1.098683 | 0.01642 | 73.51618 | 0.965076 | 0.98282039 |  | 4.36525E-08 | 8.69848E-17 | 21.90662981 | 0.10014 | 0.493288222 |  | 0.689048 | 0.37919 | 1.252108 | 0.224219 | 0.421517679 |
| metabolite220 | 19168434.53 | 167642.8 | 2191736586 | 2.84E-10 | 1.17E-07 |  | 0.546562 | 0.006279 | 47.57307 | 0.791422 | 0.918950642 |  | 0.017395212 | 7.66637E-12 | 39470209.54 | 0.713112 | 0.920092212 |  | 0.233521 | 0.130881 | 0.416655 | 2.98E-06 | 0.00025598 |
| metabolite221 | 0.346026861 | 0.001409 | 84.96471787 | 0.706191 | 0.824370679 |  | 0.067712 | 0.000912 | 5.027699 | 0.223112 | 0.505166221 |  | 0.227450822 | 1.86745E-10 | 277029889.7 | 0.889911 | 0.967390372 |  | 0.974245 | 0.523937 | 1.811579 | 0.934439 | 0.967936596 |
| metabolite222 | 0.008342169 | 5.61E-05 | 1.240996762 | 0.063365 | 0.159249077 |  | 2.729655 | 0.050164 | 148.5335 | 0.623367 | 0.822537502 |  | 382206.6363 | 0.00183395 | 7.96543E+13 | 0.191142 | 0.591107063 |  | 1.541715 | 0.874824 | 2.716984 | 0.137124 | 0.316854688 |
| metabolite223 | 3.10913E-05 | 1.95E-07 | 0.004958487 | 0.00011 | 0.001297079 |  | 4.899723 | 0.068972 | 348.0737 | 0.466553 | 0.716333142 |  | 821516.3994 | 0.001066759 | 6.32654E+14 | 0.194756 | 0.593481771 |  | 2.268467 | 1.25496 | 4.100484 | 0.007755 | 0.048806967 |
| metabolite224 | 1.643174176 | 0.00429 | 629.3404285 | 0.870305 | 0.930213274 |  | 28.12712 | 0.270825 | 2921.204 | 0.161758 | 0.430111247 |  | 26998.56623 | 4.47433E-06 | 1.62912E+14 | 0.376448 | 0.736490425 |  | 1.333519 | 0.68379 | 2.600613 | 0.400151 | 0.61210182 |
| metabolite225 | 0.043153449 | 0.000172 | 10.82374914 | 0.267244 | 0.435548387 |  | 8.476023 | 0.108676 | 661.0732 | 0.338379 | 0.613446617 |  | 0.00925312 | 6.41984E-12 | 13336813.03 | 0.664248 | 0.898332447 |  | 1.616797 | 0.870299 | 3.003604 | 0.131257 | 0.308440007 |
| metabolite226 | 1.97663E-05 | 1.3E-07 | 0.003001404 | 4.89E-05 | 0.00070836 |  | 1.31445 | 0.018538 | 93.20067 | 0.90015 | 0.962668145 |  | 0.000717858 | 8.82265E-13 | 584087.5761 | 0.490653 | 0.80640431 |  | 2.381428 | 1.322939 | 4.286818 | 0.004592 | 0.034708056 |
| metabolite227 | 0.590027678 | 0.002093 | 166.3359116 | 0.854902 | 0.919577252 |  | 0.5104 | 0.006012 | 43.32816 | 0.76717 | 0.904373702 |  | 6.595902049 | 3.24066E-09 | 13425038524 | 0.863355 | 0.960982454 |  | 0.814697 | 0.431987 | 1.536462 | 0.527941 | 0.706996813 |
| metabolite228 | 7.72134E-06 | 5.9E-08 | 0.001010372 | 6.55E-06 | 0.000155839 |  | 4.443105 | 0.066726 | 295.8565 | 0.487753 | 0.730742678 |  | 361.5235115 | 5.68126E-07 | 2.30053E+11 | 0.570149 | 0.853748966 |  | 2.245958 | 1.254043 | 4.022452 | 0.00755 | 0.048146473 |
| metabolite229 | 2.186521831 | 0.011065 | 432.080156 | 0.772313 | 0.867104274 |  | 0.051906 | 0.000837 | 3.220056 | 0.162895 | 0.43086535 |  | 0.141688799 | 2.67428E-10 | 75069474.18 | 0.849137 | 0.955623187 |  | 0.702548 | 0.388647 | 1.269981 | 0.245001 | 0.448735763 |
| metabolite230 | 44053.39792 | 147.9699 | 13115515.52 | 0.000362 | 0.00312665 |  | 2.759061 | 0.023935 | 318.0518 | 0.676017 | 0.853009073 |  | 758082.3169 | 9.65688E-05 | 5.95108E+15 | 0.246651 | 0.639636914 |  | 0.490719 | 0.251963 | 0.955714 | 0.038611 | 0.141305445 |
| metabolite231 | 18064.83248 | 39.5961 | 8241674.493 | 0.002182 | 0.012494514 |  | 0.099158 | 0.00066 | 14.8998 | 0.368118 | 0.642560828 |  | 8.31716E-07 | 2.75157E-17 | 25140.29873 | 0.257961 | 0.647052464 |  | 0.442565 | 0.219011 | 0.894313 | 0.025062 | 0.107397606 |
| metabolite232 | 0.00107414 | 2.4E-06 | 0.481226844 | 0.030259 | 0.093186179 |  | 14.06404 | 0.106234 | 1861.899 | 0.291203 | 0.574315439 |  | 1721751752 | 0.123427852 | 2.40175E+19 | 0.077082 | 0.45805738 |  | 1.868776 | 0.934796 | 3.735922 | 0.079604 | 0.227413537 |
| metabolite233 | 487892.6891 | 2187.388 | 108823544.2 | 6.19E-06 | 0.000148153 |  | 2.55383 | 0.024004 | 271.7018 | 0.694519 | 0.866048876 |  | 33.06848259 | 5.46328E-09 | 2.00159E+11 | 0.76136 | 0.938107312 |  | 0.367166 | 0.193305 | 0.697397 | 0.002766 | 0.025387543 |
| metabolite234 | 0.001637077 | 9.22E-06 | 0.290793914 | 0.016817 | 0.059609832 |  | 0.350982 | 0.005364 | 22.9665 | 0.62452 | 0.823083204 |  | 0.198902733 | 3.38332E-10 | 116933326 | 0.87572 | 0.965088312 |  | 1.136148 | 0.62462 | 2.06659 | 0.676619 | 0.817653672 |
| metabolite235 | 0.653344275 | 0.00274 | 155.8115227 | 0.879149 | 0.934159679 |  | 1.408292 | 0.018905 | 104.9069 | 0.876573 | 0.954228173 |  | 6.072500113 | 5.63837E-09 | 6540059792 | 0.865326 | 0.962020828 |  | 0.985269 | 0.531781 | 1.825479 | 0.962463 | 0.98204616 |
| metabolite236 | 0.103664631 | 0.000148 | 72.68010211 | 0.4992 | 0.656794916 |  | 12.37112 | 0.071794 | 2131.727 | 0.340431 | 0.616004697 |  | 13661.75674 | 2.13196E-07 | 8.75455E+14 | 0.454811 | 0.790540474 |  | 1.716236 | 0.824789 | 3.571175 | 0.151343 | 0.335139305 |
| metabolite237 | 58602.92378 | 57.97983 | 59232711.17 | 0.002376 | 0.013259948 |  | 73.11349 | 0.263969 | 20250.77 | 0.137541 | 0.400953333 |  | 1.27161E+18 | 4881598.946 | 3.31245E+29 | 0.00239 | 0.100075469 |  | 0.382837 | 0.173286 | 0.845793 | 0.019308 | 0.088069497 |
| metabolite238 | 0.001331938 | 7.19E-06 | 0.246808667 | 0.014441 | 0.05302088 |  | 0.147378 | 0.002188 | 9.925199 | 0.374608 | 0.648098075 |  | 0.063679742 | 8.99955E-11 | 45059024.24 | 0.791591 | 0.94456376 |  | 1.369315 | 0.750362 | 2.498827 | 0.307982 | 0.520914323 |
| metabolite239 | 461.9110616 | 0.638941 | 333930.2065 | 0.070442 | 0.172092597 |  | 15.45375 | 0.082185 | 2905.858 | 0.307714 | 0.587691064 |  | 252.5298297 | 2.438E-09 | 2.61572E+13 | 0.669876 | 0.901579219 |  | 0.568918 | 0.270015 | 1.1987 | 0.140815 | 0.322372139 |
| metabolite240 | 3.447290994 | 0.008407 | 1413.632304 | 0.68759 | 0.808762873 |  | 6.370653 | 0.056316 | 720.6646 | 0.444385 | 0.699260511 |  | 286167.5443 | 3.7547E-05 | 2.18105E+15 | 0.281481 | 0.665728265 |  | 1.03279 | 0.524198 | 2.034831 | 0.925873 | 0.964603095 |
| metabolite241 | 0.229850567 | 0.000582 | 90.81791756 | 0.63077 | 0.766717206 |  | 3.794549 | 0.034283 | 419.9941 | 0.579782 | 0.793059732 |  | 3.93119E-05 | 5.65954E-15 | 273065.1479 | 0.382187 | 0.741063249 |  | 1.129147 | 0.575591 | 2.215069 | 0.724527 | 0.848711841 |
| metabolite242 | 3488.138459 | 18.75138 | 648864.6459 | 0.002781 | 0.014762948 |  | 0.217465 | 0.003023 | 15.64171 | 0.485764 | 0.728940358 |  | 0.000294357 | 3.26762E-13 | 265164.9008 | 0.441227 | 0.782677331 |  | 0.544271 | 0.297981 | 0.994129 | 0.050274 | 0.168686895 |
| metabolite243 | 0.904275836 | 0.002003 | 408.2141329 | 0.974319 | 0.986419272 |  | 22.00579 | 0.184994 | 2617.683 | 0.207487 | 0.485794701 |  | 4632079801 | 0.552089372 | 3.88636E+19 | 0.058838 | 0.412757962 |  | 0.892756 | 0.4486 | 1.776668 | 0.747232 | 0.861976791 |
| metabolite244 | 0.000458005 | 2.19E-06 | 0.095847408 | 0.005687 | 0.025520901 |  | 0.048361 | 0.000644 | 3.63389 | 0.172056 | 0.443346793 |  | 0.005070406 | 3.87065E-12 | 6642049.573 | 0.622731 | 0.879305472 |  | 1.609764 | 0.868765 | 2.982787 | 0.133139 | 0.310369395 |
| metabolite245 | 3.897908845 | 0.018846 | 806.1991363 | 0.617997 | 0.755397197 |  | 0.002255 | 3.94E-05 | 0.129132 | 0.003862 | 0.104558924 |  | 2.60864E-05 | 4.47104E-14 | 15220.21077 | 0.307663 | 0.691419261 |  | 0.769232 | 0.422469 | 1.400617 | 0.392693 | 0.60568737 |
| metabolite246 | 0.034584692 | 0.000169 | 7.062935146 | 0.217708 | 0.380732682 |  | 0.140771 | 0.002107 | 9.403295 | 0.362389 | 0.635338624 |  | 0.001151637 | 1.74241E-12 | 761169.1385 | 0.515088 | 0.819395994 |  | 1.047624 | 0.573096 | 1.915065 | 0.880117 | 0.940701625 |
| metabolite247 | 145.7613376 | 0.394177 | 53900.58231 | 0.101481 | 0.226320594 |  | 21.32968 | 0.198225 | 2295.149 | 0.202512 | 0.480821359 |  | 28593833174 | 5.949250939 | 1.3743E+20 | 0.036512 | 0.342359087 |  | 0.786649 | 0.401456 | 1.541431 | 0.485884 | 0.67528941 |
| metabolite248 | 7.51043E-05 | 3.83E-07 | 0.014744088 | 0.000616 | 0.004709696 |  | 0.869597 | 0.010848 | 69.70543 | 0.950302 | 0.978289252 |  | 14.71242301 | 9.64011E-09 | 22453633877 | 0.803658 | 0.9460629 |  | 2.083407 | 1.129677 | 3.842323 | 0.020516 | 0.091952043 |
| metabolite249 | 3831.530238 | 20.41406 | 719142.8656 | 0.002535 | 0.01393901 |  | 0.155198 | 0.002145 | 11.23033 | 0.395584 | 0.665161247 |  | 4.63737E-07 | 5.53083E-16 | 388.8246488 | 0.166955 | 0.568636202 |  | 0.381518 | 0.211963 | 0.686703 | 0.001717 | 0.018210016 |
| metabolite250 | 485622.8835 | 1827.075 | 129074947 | 1.14E-05 | 0.000230395 |  | 1.92304 | 0.015909 | 232.4483 | 0.789731 | 0.917849231 |  | 33.75573366 | 3.03703E-09 | 3.75185E+11 | 0.766118 | 0.939544636 |  | 0.300938 | 0.157308 | 0.575707 | 0.000433 | 0.007043202 |
| metabolite251 | 1.14337E-05 | 8.03E-08 | 0.001628279 | 1.70E-05 | 0.000312755 |  | 89.07403 | 1.386505 | 5722.433 | 0.036766 | 0.2199105 |  | 15037858.86 | 0.024091526 | 9.38659E+15 | 0.112573 | 0.513062936 |  | 2.235212 | 1.240782 | 4.026632 | 0.008521 | 0.052010027 |
| metabolite252 | 58430629.98 | 268303.1 | 12724929145 | 2.25E-09 | 5.56E-07 |  | 1.50012 | 0.010283 | 218.8423 | 0.873547 | 0.953451165 |  | 85.13372205 | 3.12157E-09 | 2.32183E+12 | 0.717664 | 0.920092212 |  | 0.256603 | 0.131777 | 0.499671 | 0.000114 | 0.00305052 |
| metabolite253 | 4.099005685 | 0.020789 | 808.2121663 | 0.601822 | 0.741487567 |  | 3.103772 | 0.048407 | 199.0079 | 0.594724 | 0.80372156 |  | 2213.472808 | 4.34457E-06 | 1.12772E+12 | 0.453052 | 0.789332094 |  | 0.789784 | 0.435914 | 1.430919 | 0.438049 | 0.637923084 |
| metabolite254 | 0.014610018 | 5.28E-05 | 4.042306427 | 0.143553 | 0.287184383 |  | 0.294735 | 0.003391 | 25.61846 | 0.592837 | 0.801756256 |  | 15.10459587 | 6.51412E-09 | 35023734323 | 0.805543 | 0.9460629 |  | 1.251832 | 0.661301 | 2.369697 | 0.491727 | 0.680895718 |
| metabolite255 | 7.99664E-05 | 4.56E-07 | 0.01402104 | 0.000513 | 0.004086954 |  | 2547.554 | 44.7628 | 144987.1 | 0.000234 | 0.070911374 |  | 9.40528E+11 | 1778.03401 | 4.97511E+20 | 0.008244 | 0.188291096 |  | 4.176256 | 2.399549 | 7.268495 | 1.70E-06 | 0.000186553 |
| metabolite256 | 39.40824231 | 0.151515 | 10249.8557 | 0.198042 | 0.355383897 |  | 1.361167 | 0.016519 | 112.1619 | 0.891286 | 0.958865771 |  | 12.0645235 | 6.90373E-09 | 21083213038 | 0.819018 | 0.947573924 |  | 0.939962 | 0.500114 | 1.766656 | 0.847843 | 0.925219154 |
| metabolite257 | 0.007858514 | 3.54E-05 | 1.744998236 | 0.081499 | 0.192884945 |  | 0.153853 | 0.002089 | 11.33039 | 0.395318 | 0.665096037 |  | 0.065251716 | 6.01682E-11 | 70764678.82 | 0.79754 | 0.9460629 |  | 1.051176 | 0.567213 | 1.948067 | 0.874302 | 0.939114565 |
| metabolite258 | 0.112961836 | 0.000549 | 23.25127929 | 0.424064 | 0.592473505 |  | 7.647155 | 0.115841 | 504.8192 | 0.343341 | 0.618214159 |  | 0.001831959 | 2.90181E-12 | 1156546.812 | 0.543369 | 0.835657199 |  | 1.050133 | 0.575341 | 1.916741 | 0.873689 | 0.939114565 |
| metabolite259 | 0.080340522 | 0.000439 | 14.70793941 | 0.344883 | 0.517746159 |  | 1.796294 | 0.029249 | 110.3184 | 0.780913 | 0.91188411 |  | 0.000107436 | 2.70318E-13 | 42700.06338 | 0.367634 | 0.733489635 |  | 1.124925 | 0.624321 | 2.026932 | 0.695919 | 0.830938413 |
| metabolite260 | 2.83169E-05 | 1.58E-07 | 0.005070579 | 0.000134 | 0.001526763 |  | 0.01449 | 0.000198 | 1.059363 | 0.055701 | 0.260334523 |  | 4.17682E-05 | 3.26515E-14 | 53430.42988 | 0.347992 | 0.717869012 |  | 2.056568 | 1.117874 | 3.783497 | 0.022267 | 0.097906824 |
| metabolite261 | 0.043657355 | 0.00017 | 11.20266791 | 0.270967 | 0.440841705 |  | 0.299778 | 0.00373 | 24.09416 | 0.591465 | 0.801077424 |  | 0.356743416 | 2.23419E-10 | 569629453.6 | 0.924221 | 0.976740842 |  | 1.041958 | 0.555903 | 1.952998 | 0.898203 | 0.951826138 |
| metabolite262 | 0.012112616 | 5.55E-05 | 2.645063403 | 0.111106 | 0.240135646 |  | 0.439095 | 0.006032 | 31.96267 | 0.707459 | 0.871639843 |  | 0.782312716 | 8.01029E-10 | 764034096.3 | 0.981496 | 0.992234575 |  | 1.022176 | 0.553351 | 1.888213 | 0.94428 | 0.972314639 |
| metabolite263 | 0.002395378 | 1.2E-05 | 0.479883295 | 0.027656 | 0.087048676 |  | 0.025552 | 0.000379 | 1.72306 | 0.090663 | 0.327151227 |  | 0.015782595 | 1.84332E-11 | 13513122.75 | 0.693337 | 0.907533437 |  | 1.544574 | 0.843576 | 2.82809 | 0.161695 | 0.350086614 |
| metabolite264 | 0.148970494 | 0.000573 | 38.722318 | 0.503522 | 0.659572935 |  | 1.109576 | 0.013796 | 89.24135 | 0.963034 | 0.981849859 |  | 31.68299925 | 2.05079E-08 | 48947495252 | 0.749474 | 0.934575459 |  | 1.167777 | 0.623859 | 2.185914 | 0.628703 | 0.783187743 |
| metabolite265 | 0.236749453 | 0.000949 | 59.04678066 | 0.609908 | 0.74846808 |  | 0.668909 | 0.00863 | 51.84992 | 0.856572 | 0.946297778 |  | 6.097394078 | 4.66921E-09 | 7962416990 | 0.866251 | 0.962114722 |  | 0.63551 | 0.343012 | 1.177431 | 0.152441 | 0.336805369 |
| metabolite266 | 10280.58713 | 33.091 | 3193934.352 | 0.002065 | 0.011973223 |  | 11.52234 | 0.105295 | 1260.881 | 0.309783 | 0.587997129 |  | 234122617.3 | 0.04060512 | 1.34991E+18 | 0.095653 | 0.491996639 |  | 0.568258 | 0.291792 | 1.106669 | 0.099343 | 0.261157657 |
| metabolite267 | 0.003566857 | 9.08E-06 | 1.40069013 | 0.067057 | 0.166198656 |  | 2.251131 | 0.019034 | 266.2425 | 0.739603 | 0.890272914 |  | 594.7633231 | 6.04819E-08 | 5.84874E+12 | 0.587418 | 0.86487248 |  | 1.286738 | 0.650921 | 2.543621 | 0.46992 | 0.665227499 |
| metabolite268 | 0.000417105 | 4.41E-07 | 0.394083461 | 0.028006 | 0.087734885 |  | 0.029814 | 0.000125 | 7.115537 | 0.211202 | 0.489772916 |  | 4.34392E-10 | 1.64565E-21 | 114.6636683 | 0.110985 | 0.512820569 |  | 1.702014 | 0.77826 | 3.722218 | 0.185567 | 0.380884233 |
| metabolite269 | 18.67988606 | 0.053829 | 6482.386746 | 0.328765 | 0.502076647 |  | 17.0151 | 0.171742 | 1685.746 | 0.229362 | 0.511407568 |  | 27.87136165 | 5.699E-09 | 1.36307E+11 | 0.770578 | 0.940047404 |  | 0.749769 | 0.387692 | 1.45 | 0.393941 | 0.606214557 |
| metabolite270 | 63029.09086 | 52.96642 | 75003483.53 | 0.002787 | 0.014774249 |  | 39.7505 | 0.124491 | 12692.52 | 0.213282 | 0.4913029 |  | 3.11737E+16 | 52480.84712 | 1.85172E+28 | 0.007045 | 0.177844242 |  | 0.430721 | 0.19052 | 0.973759 | 0.045383 | 0.157252627 |
| metabolite271 | 0.000433355 | 2.37E-06 | 0.079091287 | 0.004303 | 0.020515633 |  | 3.020866 | 0.043127 | 211.5987 | 0.611097 | 0.814869082 |  | 0.000352658 | 4.53589E-13 | 274185.3944 | 0.448179 | 0.785636346 |  | 1.952953 | 1.076472 | 3.543078 | 0.029699 | 0.121380454 |
| metabolite272 | 2.076349065 | 0.004387 | 982.6821547 | 0.816594 | 0.895502195 |  | 2.834398 | 0.022239 | 361.2427 | 0.674405 | 0.852133236 |  | 706.4365271 | 4.97884E-08 | 1.00235E+13 | 0.583383 | 0.862523742 |  | 0.748256 | 0.374597 | 1.494638 | 0.413098 | 0.618713835 |
| metabolite273 | 0.08246022 | 0.000275 | 24.73213259 | 0.39299 | 0.561998321 |  | 21.14834 | 0.242048 | 1847.787 | 0.183634 | 0.457170747 |  | 9.97113E+12 | 7444.87617 | 1.33546E+22 | 0.006179 | 0.168601937 |  | 1.233887 | 0.64842 | 2.347976 | 0.523326 | 0.703390434 |
| metabolite274 | 1.82019294 | 0.008592 | 385.6087151 | 0.826909 | 0.9020159 |  | 1.081239 | 0.015919 | 73.43957 | 0.971115 | 0.986533912 |  | 4.363238514 | 6.33343E-09 | 3005930136 | 0.887426 | 0.966893207 |  | 1.637095 | 0.90169 | 2.972288 | 0.108089 | 0.275871002 |
| metabolite275 | 6968.927335 | 24.0574 | 2018752.96 | 0.002779 | 0.014762948 |  | 35.90294 | 0.360783 | 3572.844 | 0.129943 | 0.387325551 |  | 1424.445123 | 2.7013E-07 | 7.51137E+12 | 0.526225 | 0.824919067 |  | 0.571558 | 0.296382 | 1.102221 | 0.097832 | 0.258830584 |
| metabolite276 | 0.001362128 | 1.59E-06 | 1.164604381 | 0.057968 | 0.149909345 |  | 0.042926 | 0.000199 | 9.236613 | 0.253116 | 0.537946712 |  | 1.437E-10 | 9.6546E-22 | 21.38847544 | 0.087012 | 0.475490674 |  | 2.238408 | 1.048675 | 4.777904 | 0.039561 | 0.143589072 |
| metabolite277 | 0.017926168 | 3.3E-05 | 9.745279735 | 0.213393 | 0.375130465 |  | 0.59246 | 0.004018 | 87.35675 | 0.837582 | 0.937071771 |  | 0.112079301 | 3.85456E-12 | 3258941431 | 0.859016 | 0.958572817 |  | 1.174133 | 0.575066 | 2.397272 | 0.660219 | 0.80605611 |
| metabolite278 | 0.090991434 | 0.000429 | 19.30660094 | 0.382418 | 0.551443738 |  | 4.675713 | 0.068483 | 319.2351 | 0.475639 | 0.721626069 |  | 44.30783264 | 6.0475E-08 | 32462726520 | 0.716527 | 0.920092212 |  | 1.4519 | 0.795576 | 2.649671 | 0.226987 | 0.4247845 |
| metabolite279 | 0.000757196 | 4.39E-06 | 0.130715946 | 0.00728 | 0.031160219 |  | 4.043447 | 0.0617 | 264.9819 | 0.514017 | 0.751824235 |  | 1.967731771 | 3.26068E-09 | 1187470877 | 0.9478 | 0.986298878 |  | 2.858154 | 1.621674 | 5.037416 | 0.000427 | 0.007039017 |
| metabolite280 | 1.876217406 | 0.009137 | 385.2812827 | 0.817255 | 0.89596214 |  | 0.016614 | 0.000269 | 1.026927 | 0.054016 | 0.257328813 |  | 1.33611E-07 | 2.70165E-16 | 66.07766356 | 0.124062 | 0.527043366 |  | 0.702721 | 0.387083 | 1.275739 | 0.24871 | 0.453544911 |
| metabolite281 | 0.001556055 | 6.89E-06 | 0.351187335 | 0.021153 | 0.0709759 |  | 0.298267 | 0.003791 | 23.46686 | 0.5881 | 0.799135378 |  | 7.06382E-05 | 5.2792E-14 | 94517.19802 | 0.374611 | 0.736141064 |  | 1.104617 | 0.591261 | 2.06369 | 0.755602 | 0.86674608 |
| metabolite282 | 0.004391984 | 1.83E-05 | 1.053709149 | 0.054758 | 0.143914965 |  | 220.7211 | 3.080909 | 15812.8 | 0.014785 | 0.152336933 |  | 6.32319E+11 | 745.293898 | 5.36469E+20 | 0.010869 | 0.212626714 |  | 2.516116 | 1.375686 | 4.60195 | 0.003381 | 0.028447123 |
| metabolite283 | 4.591792974 | 0.019042 | 1107.249057 | 0.587091 | 0.730370228 |  | 1.666257 | 0.022069 | 125.805 | 0.817407 | 0.927644858 |  | 8.624113325 | 7.49202E-09 | 9927276573 | 0.839972 | 0.954234322 |  | 0.919282 | 0.495266 | 1.706315 | 0.790189 | 0.891033439 |
| metabolite284 | 127.9042805 | 0.389311 | 42021.73312 | 0.103647 | 0.229249448 |  | 0.280677 | 0.002788 | 28.25841 | 0.590304 | 0.800466136 |  | 26.22486354 | 5.572E-09 | 1.23428E+11 | 0.774283 | 0.940047404 |  | 0.586014 | 0.304993 | 1.125968 | 0.111574 | 0.281406417 |
| metabolite285 | 0.023973096 | 0.000115 | 4.998893598 | 0.173652 | 0.32675075 |  | 0.067603 | 0.001003 | 4.556387 | 0.21245 | 0.490298887 |  | 0.046379893 | 6.09203E-11 | 35309954.46 | 0.769066 | 0.940047404 |  | 1.185047 | 0.646646 | 2.171724 | 0.583855 | 0.751278668 |
| metabolite286 | 0.448951721 | 0.002105 | 95.72949032 | 0.770286 | 0.866770268 |  | 0.033739 | 0.000518 | 2.198548 | 0.114605 | 0.364939136 |  | 8.29304E-09 | 1.57091E-17 | 4.377990673 | 0.072085 | 0.447917707 |  | 0.749099 | 0.410352 | 1.367482 | 0.348861 | 0.560442601 |
| metabolite287 | 0.00035274 | 2.27E-06 | 0.054788825 | 0.002544 | 0.013966866 |  | 0.172434 | 0.002781 | 10.69266 | 0.405664 | 0.67386668 |  | 0.690608282 | 1.45901E-09 | 326891836.5 | 0.97109 | 0.990751288 |  | 1.67989 | 0.93651 | 3.013347 | 0.084635 | 0.236863845 |
| metabolite288 | 122.8667114 | 0.62986 | 23967.5799 | 0.076474 | 0.183211436 |  | 3.675972 | 0.054854 | 246.342 | 0.545219 | 0.773434982 |  | 0.000719097 | 1.12421E-12 | 459967.4261 | 0.485636 | 0.802577618 |  | 0.721483 | 0.396189 | 1.313863 | 0.288095 | 0.501229113 |
| metabolite289 | 3.241075494 | 0.012213 | 860.0927263 | 0.680434 | 0.803657107 |  | 0.017003 | 0.000223 | 1.294403 | 0.067959 | 0.287567984 |  | 4.41487E-09 | 3.64038E-18 | 5.354123701 | 0.074137 | 0.451538889 |  | 0.83576 | 0.445896 | 1.566498 | 0.576795 | 0.745554827 |
| metabolite290 | 0.045374831 | 8.75E-05 | 23.5392262 | 0.334317 | 0.507900927 |  | 0.120587 | 0.000873 | 16.65027 | 0.401945 | 0.670390102 |  | 928.0177515 | 4.20661E-08 | 2.04729E+13 | 0.575033 | 0.856319102 |  | 1.150651 | 0.567604 | 2.332609 | 0.697862 | 0.831848922 |
| metabolite291 | 0.125863922 | 0.000589 | 26.9126902 | 0.450566 | 0.616083905 |  | 1.20743 | 0.017474 | 83.43044 | 0.930649 | 0.974227887 |  | 0.555801152 | 7.41071E-10 | 416849258.3 | 0.955178 | 0.9871034 |  | 1.763877 | 0.971314 | 3.203149 | 0.06491 | 0.198746033 |
| metabolite292 | 22.01894151 | 0.088073 | 5504.894235 | 0.274775 | 0.445024204 |  | 0.010849 | 0.000149 | 0.79131 | 0.041065 | 0.230897809 |  | 1.14298E-06 | 9.2337E-16 | 1414.828126 | 0.20292 | 0.597241307 |  | 0.577306 | 0.311523 | 1.069845 | 0.083666 | 0.234874892 |
| metabolite293 | 79331.57306 | 294.7097 | 21354906.69 | 0.000137 | 0.001544492 |  | 0.185525 | 0.001695 | 20.30378 | 0.483408 | 0.727464944 |  | 9.01125E-11 | 1.87572E-20 | 0.432915119 | 0.044376 | 0.375945688 |  | 0.427913 | 0.222419 | 0.823265 | 0.012384 | 0.065920012 |
| metabolite294 | 0.000151227 | 7.51E-07 | 0.030465485 | 0.001529 | 0.009444014 |  | 8.575183 | 0.110263 | 666.8968 | 0.335452 | 0.611358189 |  | 0.123910411 | 8.58304E-11 | 178885146 | 0.846482 | 0.955623187 |  | 2.069481 | 1.12379 | 3.810991 | 0.021361 | 0.094595828 |
| metabolite295 | 0.004002835 | 2.66E-05 | 0.602740818 | 0.033092 | 0.099115867 |  | 0.263813 | 0.004722 | 14.7385 | 0.517548 | 0.753564307 |  | 0.00612144 | 2.2458E-11 | 1668538.908 | 0.608114 | 0.874215598 |  | 1.613054 | 0.912538 | 2.851326 | 0.10278 | 0.267192118 |
| metabolite296 | 2.388600438 | 0.008536 | 668.4225901 | 0.762535 | 0.862471218 |  | 1.289418 | 0.015237 | 109.1161 | 0.910824 | 0.967945632 |  | 12.78513745 | 6.43937E-09 | 25384427329 | 0.815962 | 0.946906762 |  | 0.968622 | 0.513373 | 1.827578 | 0.921773 | 0.962095745 |
| metabolite297 | 22.79483525 | 0.072363 | 7180.495296 | 0.28907 | 0.460996091 |  | 0.034589 | 0.000381 | 3.144021 | 0.146527 | 0.411005424 |  | 6.002820757 | 1.73557E-09 | 20761989813 | 0.873225 | 0.964446262 |  | 0.614958 | 0.322657 | 1.172059 | 0.142365 | 0.32414565 |
| metabolite298 | 1.790142588 | 0.006255 | 512.3079519 | 0.840471 | 0.911278495 |  | 24.86431 | 0.300777 | 2055.453 | 0.156492 | 0.422358153 |  | 2517453.053 | 0.001387472 | 4.56771E+15 | 0.178159 | 0.575285157 |  | 1.441196 | 0.764779 | 2.71588 | 0.260706 | 0.467832873 |
| metabolite299 | 2.095035573 | 0.005605 | 783.1274118 | 0.807138 | 0.889621162 |  | 0.960195 | 0.009036 | 102.028 | 0.986417 | 0.994456263 |  | 0.167560494 | 2.8094E-11 | 999376378.1 | 0.876667 | 0.965088312 |  | 1.072302 | 0.550188 | 2.089886 | 0.837918 | 0.919530925 |
| metabolite300 | 2.056592607 | 0.0062 | 682.2147545 | 0.808079 | 0.889803214 |  | 10.46876 | 0.11053 | 991.5437 | 0.314013 | 0.592125811 |  | 44847.83314 | 1.29248E-05 | 1.55618E+14 | 0.341315 | 0.713874057 |  | 1.322029 | 0.688846 | 2.537229 | 0.403084 | 0.613011721 |
| metabolite301 | 0.015876844 | 9.02E-05 | 2.793487265 | 0.119132 | 0.252916465 |  | 4.389664 | 0.07224 | 266.7395 | 0.481701 | 0.726556062 |  | 1.724398144 | 4.09092E-09 | 726865712.5 | 0.95721 | 0.9871034 |  | 1.104676 | 0.613263 | 1.989862 | 0.740855 | 0.859698164 |
| metabolite302 | 0.008958164 | 4.99E-05 | 1.607535286 | 0.077693 | 0.18529455 |  | 151.6914 | 2.674567 | 8603.367 | 0.016376 | 0.15744195 |  | 2678884.033 | 0.006699489 | 1.07119E+15 | 0.145843 | 0.546460871 |  | 1.402137 | 0.777603 | 2.528268 | 0.263556 | 0.47158482 |
| metabolite303 | 0.052572128 | 0.000252 | 10.94744799 | 0.281857 | 0.452997681 |  | 0.016317 | 0.000256 | 1.041377 | 0.054809 | 0.25844294 |  | 0.028409212 | 4.01035E-11 | 20125005.21 | 0.732622 | 0.925814843 |  | 0.868135 | 0.474571 | 1.588082 | 0.647186 | 0.797379217 |
| metabolite304 | 0.000287899 | 1.57E-06 | 0.052901703 | 0.002733 | 0.014591755 |  | 4.99501 | 0.07018 | 355.5161 | 0.46138 | 0.712136233 |  | 15.43150475 | 1.70703E-08 | 13950057583 | 0.795287 | 0.945629989 |  | 1.975186 | 1.085793 | 3.593098 | 0.027785 | 0.115208465 |
| metabolite305 | 1.321492584 | 0.007025 | 248.5877858 | 0.917097 | 0.956132096 |  | 0.075615 | 0.001258 | 4.544481 | 0.219227 | 0.500219786 |  | 4.16431E-05 | 1.03912E-13 | 16688.67091 | 0.320449 | 0.700344699 |  | 0.906566 | 0.502712 | 1.634857 | 0.744995 | 0.861001512 |
| metabolite306 | 0.03956743 | 0.000162 | 9.692968082 | 0.252318 | 0.419001144 |  | 0.771844 | 0.009889 | 60.24275 | 0.907475 | 0.965785701 |  | 15.2281846 | 1.13389E-08 | 20451533933 | 0.800011 | 0.9460629 |  | 0.58721 | 0.317338 | 1.086589 | 0.09278 | 0.25162578 |
| metabolite307 | 0.002334078 | 1E-05 | 0.542662436 | 0.031379 | 0.095303955 |  | 23.78634 | 0.309557 | 1827.743 | 0.155342 | 0.421465998 |  | 0.033582821 | 2.23511E-11 | 50458560.7 | 0.753509 | 0.936460506 |  | 1.607815 | 0.86448 | 2.990317 | 0.136453 | 0.315498589 |
| metabolite308 | 16.79497756 | 0.05224 | 5399.488283 | 0.340249 | 0.513695564 |  | 41.2989 | 0.453377 | 3761.991 | 0.108853 | 0.355281522 |  | 98873.59035 | 2.9908E-05 | 3.26868E+14 | 0.305962 | 0.68936977 |  | 0.890312 | 0.46358 | 1.709856 | 0.727792 | 0.851190927 |
| metabolite309 | 46503.48418 | 248.098 | 8716611.559 | 0.000104 | 0.001242075 |  | 0.012633 | 0.000165 | 0.965126 | 0.050631 | 0.253251612 |  | 52.19923639 | 3.01596E-08 | 90344658889 | 0.716236 | 0.920092212 |  | 0.294544 | 0.163493 | 0.53064 | 8.84E-05 | 0.002574089 |
| metabolite310 | 0.064459594 | 0.000158 | 26.31843574 | 0.37334 | 0.543496624 |  | 0.477757 | 0.004137 | 55.17349 | 0.761054 | 0.900309113 |  | 8226.429891 | 9.69707E-07 | 6.97882E+13 | 0.441223 | 0.782677331 |  | 1.132821 | 0.57435 | 2.234323 | 0.719627 | 0.846417908 |
| metabolite311 | 0.013935728 | 6.05E-05 | 3.208484424 | 0.126431 | 0.263434492 |  | 8.013919 | 0.107531 | 597.2488 | 0.346114 | 0.62049668 |  | 206781.1461 | 0.000199463 | 2.14368E+14 | 0.25033 | 0.641381034 |  | 1.414195 | 0.763957 | 2.617878 | 0.272394 | 0.482308416 |
| metabolite312 | 0.413671299 | 0.001859 | 92.03857955 | 0.749501 | 0.852666193 |  | 1.746396 | 0.024743 | 123.2625 | 0.797868 | 0.920823742 |  | 948.1002698 | 1.18037E-06 | 7.61537E+11 | 0.513681 | 0.819395994 |  | 1.034703 | 0.562738 | 1.902501 | 0.91278 | 0.958092754 |
| metabolite313 | 0.002971001 | 9.89E-06 | 0.892333738 | 0.048037 | 0.13136742 |  | 10.09719 | 0.106463 | 957.6387 | 0.321627 | 0.599401439 |  | 483091.6606 | 0.000145078 | 1.60863E+15 | 0.24453 | 0.637028366 |  | 1.591143 | 0.832031 | 3.042841 | 0.163083 | 0.352065957 |
| metabolite314 | 0.131406741 | 0.000319 | 54.16825534 | 0.510248 | 0.665096936 |  | 6.918741 | 0.060623 | 789.6168 | 0.425268 | 0.68885575 |  | 392300.1508 | 4.93453E-05 | 3.11883E+15 | 0.270523 | 0.655253129 |  | 1.180944 | 0.598948 | 2.328464 | 0.632062 | 0.784791292 |
| metabolite315 | 2.451584994 | 0.007786 | 771.9600715 | 0.760517 | 0.860713711 |  | 1.386701 | 0.014934 | 128.7626 | 0.887795 | 0.95849259 |  | 1.372157333 | 4.39011E-10 | 4288766535 | 0.977423 | 0.991698663 |  | 0.872904 | 0.456742 | 1.668254 | 0.681624 | 0.821001495 |
| metabolite316 | 45.99017249 | 0.168359 | 12563.00319 | 0.18378 | 0.338297821 |  | 0.265606 | 0.003114 | 22.65819 | 0.560129 | 0.785873824 |  | 1.655403627 | 7.73052E-10 | 3544859600 | 0.963407 | 0.988990812 |  | 0.692091 | 0.367387 | 1.303775 | 0.25714 | 0.463226315 |
| metabolite317 | 69.3553172 | 0.302062 | 15924.42681 | 0.129259 | 0.267978166 |  | 0.10986 | 0.001482 | 8.146197 | 0.316957 | 0.595860722 |  | 88.17232149 | 7.74118E-08 | 1.00429E+11 | 0.674565 | 0.902621002 |  | 0.555063 | 0.301898 | 1.020528 | 0.060747 | 0.189759212 |
| metabolite318 | 4.524529209 | 0.010536 | 1943.022436 | 0.626495 | 0.763270314 |  | 99.13836 | 0.900001 | 10920.44 | 0.057927 | 0.264422304 |  | 1.614538008 | 1.5648E-10 | 16658619211 | 0.967591 | 0.989787236 |  | 0.838505 | 0.423602 | 1.659788 | 0.614152 | 0.772874325 |
| metabolite319 | 0.00436371 | 1.52E-05 | 1.250229706 | 0.062368 | 0.157985471 |  | 0.556793 | 0.006036 | 51.36095 | 0.800222 | 0.921663861 |  | 0.413346342 | 1.36008E-10 | 1256218526 | 0.936933 | 0.980253548 |  | 2.025583 | 1.074581 | 3.818222 | 0.031187 | 0.125565587 |
| metabolite320 | 31.53403912 | 0.118818 | 8369.069312 | 0.22811 | 0.391001382 |  | 0.059932 | 0.000741 | 4.846538 | 0.211824 | 0.490285108 |  | 20.79656032 | 1.12623E-08 | 38402151544 | 0.780933 | 0.94146208 |  | 0.676203 | 0.360639 | 1.26789 | 0.225073 | 0.422482367 |
| metabolite321 | 0.047332956 | 0.000234 | 9.573758888 | 0.262555 | 0.429856163 |  | 13.28622 | 0.203831 | 866.0295 | 0.227427 | 0.509702039 |  | 153.1536246 | 2.42078E-07 | 96894473218 | 0.627485 | 0.879305472 |  | 1.059828 | 0.580844 | 1.933799 | 0.850142 | 0.925726864 |
| metabolite322 | 10575.3829 | 40.2238 | 2780411.637 | 0.001481 | 0.009334108 |  | 0.002257 | 2.63E-05 | 0.193369 | 0.008398 | 0.126652263 |  | 4.20329E-08 | 1.25495E-17 | 140.7831028 | 0.131887 | 0.530263915 |  | 0.38473 | 0.204398 | 0.72416 | 0.00376 | 0.03059961 |
| metabolite323 | 0.000911383 | 5.02E-06 | 0.16551183 | 0.009544 | 0.038236224 |  | 6.399723 | 0.09516 | 430.3943 | 0.38917 | 0.660059856 |  | 1.591394162 | 2.25976E-09 | 1120707473 | 0.964428 | 0.989435728 |  | 1.591641 | 0.875436 | 2.893783 | 0.130397 | 0.307588089 |
| metabolite324 | 0.35500938 | 0.001535 | 82.10486135 | 0.709951 | 0.826440128 |  | 0.364471 | 0.005019 | 26.46472 | 0.645233 | 0.833774355 |  | 0.252209068 | 2.60027E-10 | 244625952.7 | 0.896426 | 0.96870562 |  | 0.670379 | 0.364606 | 1.232584 | 0.200764 | 0.398412495 |
| metabolite325 | 114.1540294 | 0.254685 | 51165.7171 | 0.131125 | 0.270364382 |  | 0.403933 | 0.003148 | 51.83045 | 0.71506 | 0.874551186 |  | 0.000424899 | 2.94896E-14 | 6122132.486 | 0.516689 | 0.820468337 |  | 0.711665 | 0.356262 | 1.421612 | 0.337386 | 0.550028652 |
| metabolite326 | 1.114945044 | 0.004655 | 267.0578106 | 0.96902 | 0.984976957 |  | 57.51813 | 0.822251 | 4023.508 | 0.064161 | 0.278407555 |  | 1206.894953 | 1.14859E-06 | 1.26816E+12 | 0.504555 | 0.816435137 |  | 0.838351 | 0.452682 | 1.552599 | 0.576074 | 0.744931712 |
| metabolite327 | 5.540419798 | 0.016713 | 1836.721607 | 0.564303 | 0.712627998 |  | 0.857267 | 0.008819 | 83.33402 | 0.947535 | 0.977627498 |  | 289.4114224 | 7.62342E-08 | 1.09871E+12 | 0.615511 | 0.877473841 |  | 1.003822 | 0.521579 | 1.931936 | 0.99091 | 0.993652937 |
| metabolite328 | 0.150958388 | 0.000756 | 30.13039466 | 0.485571 | 0.644698499 |  | 0.770103 | 0.011788 | 50.31053 | 0.902719 | 0.96430326 |  | 1.12806E-06 | 2.31488E-15 | 549.7119975 | 0.182394 | 0.580004487 |  | 1.250635 | 0.688812 | 2.270704 | 0.463916 | 0.659108358 |
| metabolite329 | 3.983154927 | 0.012061 | 1315.413065 | 0.641373 | 0.776553038 |  | 0.125735 | 0.001322 | 11.96104 | 0.374211 | 0.647713885 |  | 0.00070856 | 1.94692E-13 | 2578721.431 | 0.519826 | 0.822282435 |  | 0.938704 | 0.488174 | 1.805025 | 0.849953 | 0.925726864 |
| metabolite330 | 3.275952616 | 0.017751 | 604.5800632 | 0.656665 | 0.786754015 |  | 67.43873 | 1.19008 | 3821.579 | 0.043272 | 0.236415195 |  | 2174373.718 | 0.006327442 | 7.47206E+14 | 0.148455 | 0.548173861 |  | 0.98723 | 0.548197 | 1.77787 | 0.965922 | 0.983904973 |
| metabolite331 | 2.865442635 | 0.009131 | 899.2267189 | 0.72034 | 0.833694452 |  | 0.040615 | 0.000456 | 3.620163 | 0.164775 | 0.433105382 |  | 0.024884806 | 8.12341E-12 | 76230754.67 | 0.740948 | 0.929662886 |  | 0.867947 | 0.454294 | 1.658248 | 0.668916 | 0.812552731 |
| metabolite332 | 3.120418357 | 0.007687 | 1266.650767 | 0.711081 | 0.826440128 |  | 4.55739 | 0.040466 | 513.2686 | 0.530448 | 0.764725841 |  | 223681.0765 | 3.04462E-05 | 1.64333E+15 | 0.290198 | 0.673147458 |  | 0.878501 | 0.446613 | 1.72804 | 0.70816 | 0.837715271 |
| metabolite333 | 0.026820377 | 6.89E-05 | 10.43995052 | 0.236913 | 0.401120207 |  | 14.30329 | 0.130047 | 1573.153 | 0.269656 | 0.556559039 |  | 332733907.2 | 0.055784421 | 1.98464E+18 | 0.090306 | 0.479980149 |  | 1.553786 | 0.794221 | 3.039772 | 0.200735 | 0.398412495 |
| metabolite334 | 13.23460771 | 0.048624 | 3602.24129 | 0.368509 | 0.53946196 |  | 0.014124 | 0.000181 | 1.104804 | 0.058042 | 0.264422304 |  | 3.26574E-07 | 2.03365E-16 | 524.4285976 | 0.170069 | 0.568636202 |  | 0.500163 | 0.268903 | 0.930312 | 0.030755 | 0.124191864 |
| metabolite335 | 0.008690993 | 6.25E-06 | 12.08422542 | 0.201414 | 0.359869383 |  | 0.127311 | 0.000414 | 39.14755 | 0.482153 | 0.726556062 |  | 0.061066531 | 5.72726E-14 | 65111802777 | 0.843517 | 0.955230753 |  | 1.749619 | 0.774729 | 3.951272 | 0.181079 | 0.375619751 |
| metabolite336 | 988.530017 | 4.146572 | 235662.5418 | 0.015063 | 0.054747659 |  | 2.444988 | 0.029303 | 204.0062 | 0.692803 | 0.865071815 |  | 100703.0863 | 5.93278E-05 | 1.70933E+14 | 0.290348 | 0.673147458 |  | 0.774162 | 0.411694 | 1.455756 | 0.428615 | 0.630936456 |
| metabolite337 | 25.00128348 | 0.165647 | 3773.473842 | 0.21118 | 0.372653236 |  | 1.470836 | 0.027542 | 78.54774 | 0.849571 | 0.941402931 |  | 0.122272123 | 5.6581E-10 | 26423142.51 | 0.830452 | 0.952506813 |  | 0.655834 | 0.373246 | 1.152372 | 0.145244 | 0.327868785 |
| metabolite338 | 68.92483414 | 0.441813 | 10752.58382 | 0.103223 | 0.228829455 |  | 0.060474 | 0.001118 | 3.270808 | 0.170987 | 0.442862885 |  | 1.90782E-06 | 8.22349E-15 | 442.6058494 | 0.182966 | 0.580954027 |  | 0.60705 | 0.343936 | 1.071448 | 0.087868 | 0.243705985 |
| metabolite339 | 0.105871362 | 0.000428 | 26.17724687 | 0.426163 | 0.594098834 |  | 26.28672 | 0.353511 | 1954.655 | 0.139849 | 0.402308456 |  | 0.311289665 | 2.37419E-10 | 408144057.5 | 0.913436 | 0.974349572 |  | 1.292326 | 0.694752 | 2.403889 | 0.419758 | 0.624902667 |
| metabolite340 | 0.090595325 | 0.000124 | 66.10678619 | 0.476771 | 0.638966051 |  | 36.56148 | 0.209928 | 6367.615 | 0.174367 | 0.446876732 |  | 36950685.56 | 0.000570562 | 2.393E+18 | 0.172847 | 0.571179693 |  | 1.836188 | 0.879881 | 3.831867 | 0.108273 | 0.276049051 |
| metabolite341 | 0.011444832 | 9.73E-06 | 13.45704137 | 0.217842 | 0.380787436 |  | 39.69574 | 0.152259 | 10349.14 | 0.197352 | 0.476003291 |  | 600409550.5 | 0.001403666 | 2.56821E+20 | 0.141898 | 0.542758378 |  | 0.854716 | 0.383553 | 1.904663 | 0.701715 | 0.834101676 |
| metabolite342 | 0.129896566 | 0.000466 | 36.22624191 | 0.478916 | 0.639626783 |  | 5.0416 | 0.059839 | 424.7682 | 0.476033 | 0.721800861 |  | 38195.05413 | 2.03862E-05 | 7.15613E+13 | 0.334892 | 0.710418618 |  | 1.148433 | 0.60849 | 2.167492 | 0.670158 | 0.813529 |
| metabolite343 | 43.55861321 | 0.053176 | 35680.71376 | 0.272539 | 0.442816247 |  | 0.124257 | 0.000622 | 24.82021 | 0.441971 | 0.698062827 |  | 2175.889461 | 1.68293E-08 | 2.81325E+14 | 0.557235 | 0.845421283 |  | 0.668155 | 0.313716 | 1.423044 | 0.298119 | 0.511658131 |
| metabolite344 | 2.066626972 | 0.005985 | 713.5605977 | 0.808107 | 0.889803214 |  | 2.322196 | 0.023327 | 231.177 | 0.720328 | 0.877877645 |  | 146.2570842 | 3.37238E-08 | 6.34304E+11 | 0.660548 | 0.895940229 |  | 0.995401 | 0.515255 | 1.922974 | 0.989077 | 0.993652937 |
| metabolite345 | 15.50501857 | 0.019077 | 12601.86969 | 0.424359 | 0.592473505 |  | 0.424885 | 0.002144 | 84.19697 | 0.751697 | 0.895049124 |  | 0.067237787 | 5.5239E-13 | 8184289872 | 0.836164 | 0.953010974 |  | 0.719014 | 0.338138 | 1.528908 | 0.393285 | 0.605845296 |
| metabolite346 | 0.018077457 | 7.6E-05 | 4.298642859 | 0.153361 | 0.303048035 |  | 0.283326 | 0.003686 | 21.77709 | 0.57031 | 0.790253764 |  | 0.984012121 | 7.61555E-10 | 1271450601 | 0.998801 | 0.999851958 |  | 1.183193 | 0.635716 | 2.202158 | 0.596661 | 0.761261199 |
| metabolite347 | 0.002322576 | 1.22E-05 | 0.443236017 | 0.025538 | 0.082265385 |  | 19.1869 | 0.289528 | 1271.506 | 0.170145 | 0.441852546 |  | 420.7830207 | 5.95742E-07 | 2.97207E+11 | 0.562275 | 0.848472323 |  | 1.72747 | 0.951463 | 3.136382 | 0.075136 | 0.219206096 |
| metabolite348 | 0.032833457 | 7.53E-05 | 14.31798831 | 0.272974 | 0.443328227 |  | 13.78893 | 0.114938 | 1654.244 | 0.285032 | 0.572067351 |  | 1811.193353 | 1.56053E-07 | 2.10212E+13 | 0.527088 | 0.824919067 |  | 1.28752 | 0.647903 | 2.558572 | 0.472253 | 0.666868939 |
| metabolite349 | 40.03594391 | 0.086614 | 18506.0726 | 0.241079 | 0.405577437 |  | 0.020913 | 0.000171 | 2.562642 | 0.117776 | 0.370080485 |  | 1.02678E-07 | 8.07723E-18 | 1305.247338 | 0.177971 | 0.575285157 |  | 0.505466 | 0.255107 | 1.001526 | 0.053022 | 0.174746347 |
| metabolite350 | 245.200451 | 1.091909 | 55062.49803 | 0.048845 | 0.132989244 |  | 1.989956 | 0.02602 | 152.1849 | 0.756402 | 0.897380672 |  | 0.001400914 | 1.1762E-12 | 1668564.079 | 0.538992 | 0.832169354 |  | 0.7956 | 0.428334 | 1.477771 | 0.470712 | 0.665959817 |
| metabolite351 | 6078.476682 | 29.34312 | 1259166.759 | 0.001782 | 0.010632024 |  | 4.957508 | 0.062135 | 395.5384 | 0.475192 | 0.721241882 |  | 38.21533203 | 2.45192E-08 | 59561848602 | 0.736489 | 0.92772069 |  | 0.562008 | 0.302731 | 1.043344 | 0.070603 | 0.210296582 |
| metabolite352 | 0.341947953 | 0.001195 | 97.84190756 | 0.710724 | 0.826440128 |  | 1.200368 | 0.013924 | 103.4845 | 0.936131 | 0.975459713 |  | 272.2893932 | 1.28004E-07 | 5.79213E+11 | 0.609906 | 0.874215598 |  | 1.717958 | 0.91539 | 3.224176 | 0.094844 | 0.254494682 |
| metabolite353 | 0.058715121 | 0.000178 | 19.34136919 | 0.339894 | 0.513368609 |  | 10.83806 | 0.113113 | 1038.459 | 0.308178 | 0.587691064 |  | 4416.757024 | 1.15923E-06 | 1.68281E+13 | 0.457431 | 0.792496109 |  | 1.296228 | 0.674039 | 2.492746 | 0.438418 | 0.637923084 |
| metabolite354 | 189.5254503 | 0.456029 | 78766.71379 | 0.091035 | 0.209443693 |  | 0.320246 | 0.002622 | 39.11493 | 0.643231 | 0.833081041 |  | 33.37945223 | 2.8131E-09 | 3.96072E+11 | 0.767479 | 0.940047404 |  | 0.666727 | 0.336468 | 1.321149 | 0.247805 | 0.452560798 |
| metabolite355 | 206.5088072 | 0.844201 | 50516.25835 | 0.060075 | 0.153880749 |  | 0.043832 | 0.000559 | 3.436802 | 0.162734 | 0.43086535 |  | 0.034523318 | 2.09618E-11 | 56858543.02 | 0.756473 | 0.937628791 |  | 0.440742 | 0.239299 | 0.81176 | 0.00977 | 0.056123804 |
| metabolite356 | 3.762334741 | 0.009557 | 1481.132218 | 0.664682 | 0.793896579 |  | 4.486654 | 0.040762 | 493.8465 | 0.532701 | 0.765926082 |  | 432111.9129 | 6.66212E-05 | 2.80272E+15 | 0.262703 | 0.648628965 |  | 0.970125 | 0.494622 | 1.902752 | 0.929837 | 0.966583064 |
| metabolite357 | 2.671025148 | 0.010285 | 693.6646706 | 0.729724 | 0.839623847 |  | 16.12088 | 0.208163 | 1248.461 | 0.212919 | 0.491076651 |  | 0.058471738 | 3.9065E-11 | 87519284.57 | 0.792726 | 0.944703928 |  | 1.184098 | 0.633311 | 2.2139 | 0.597675 | 0.76192816 |
| metabolite358 | 7815.219166 | 41.89087 | 1458018.213 | 0.001069 | 0.007291593 |  | 4.59553 | 0.061588 | 342.9054 | 0.489657 | 0.73260055 |  | 3869.865438 | 3.60477E-06 | 4.15445E+12 | 0.43784 | 0.782677331 |  | 0.406123 | 0.223995 | 0.736338 | 0.003671 | 0.030171356 |
| metabolite359 | 0.608612858 | 0.001905 | 194.4873575 | 0.866285 | 0.927609805 |  | 5.156173 | 0.055505 | 478.9857 | 0.479543 | 0.725472534 |  | 15275425.82 | 0.005778154 | 4.03829E+16 | 0.137906 | 0.537530978 |  | 1.119894 | 0.585028 | 2.143765 | 0.733151 | 0.854498762 |
| metabolite360 | 0.205514393 | 0.000998 | 42.33006301 | 0.561691 | 0.710925703 |  | 0.216815 | 0.003277 | 14.3444 | 0.476266 | 0.721800861 |  | 0.042905589 | 6.79456E-11 | 27093573.37 | 0.761268 | 0.938107312 |  | 0.979109 | 0.536799 | 1.785871 | 0.945232 | 0.972755138 |
| metabolite361 | 0.455210163 | 0.002172 | 95.41929886 | 0.773445 | 0.867408369 |  | 6.579642 | 0.099053 | 437.0576 | 0.380755 | 0.652948304 |  | 36.86388622 | 5.6046E-08 | 24246960009 | 0.728342 | 0.924067425 |  | 0.811401 | 0.444834 | 1.480039 | 0.496971 | 0.68533928 |
| metabolite362 | 0.067014029 | 0.000368 | 12.19872982 | 0.310916 | 0.484183577 |  | 31.55981 | 0.540736 | 1841.976 | 0.098998 | 0.342386296 |  | 909.0684082 | 2.23573E-06 | 3.69635E+11 | 0.501987 | 0.813127466 |  | 1.366171 | 0.760294 | 2.45487 | 0.298994 | 0.512382264 |
| metabolite363 | 1.555021142 | 0.006393 | 378.254028 | 0.875137 | 0.931621453 |  | 1.690702 | 0.022359 | 127.8444 | 0.812361 | 0.926554541 |  | 0.005673943 | 4.98227E-12 | 6461635.981 | 0.627851 | 0.879305472 |  | 0.920095 | 0.49559 | 1.708217 | 0.792417 | 0.892475506 |
| metabolite364 | 18.07648903 | 0.047346 | 6901.534371 | 0.341986 | 0.515232931 |  | 2.93591 | 0.026805 | 321.5619 | 0.653944 | 0.840590574 |  | 524.4705394 | 7.65003E-08 | 3.59567E+12 | 0.588939 | 0.865026277 |  | 0.801875 | 0.409868 | 1.568807 | 0.520353 | 0.700918259 |
| metabolite365 | 3.770537988 | 0.013068 | 1087.924054 | 0.64698 | 0.780540757 |  | 0.108235 | 0.001269 | 9.228717 | 0.329095 | 0.605724591 |  | 0.183733789 | 8.11958E-11 | 415761528.3 | 0.877757 | 0.96528138 |  | 0.623244 | 0.331072 | 1.173262 | 0.145767 | 0.328839434 |
| metabolite366 | 0.037388822 | 0.000214 | 6.529582545 | 0.214782 | 0.37671285 |  | 5.608818 | 0.094718 | 332.1307 | 0.409381 | 0.676533319 |  | 0.006333716 | 1.71211E-11 | 2343074.07 | 0.616044 | 0.877473841 |  | 1.654391 | 0.928094 | 2.949066 | 0.090622 | 0.248372808 |
| metabolite367 | 937.2947647 | 5.685452 | 154520.9459 | 0.009825 | 0.039037603 |  | 0.049504 | 0.000816 | 3.002591 | 0.154082 | 0.421307304 |  | 0.026535716 | 5.59221E-11 | 12591530.86 | 0.722469 | 0.922880405 |  | 0.412079 | 0.23322 | 0.728109 | 0.00284 | 0.025594573 |
| metabolite368 | 221.5828118 | 0.878895 | 55864.41715 | 0.058163 | 0.150205068 |  | 0.094557 | 0.001156 | 7.732454 | 0.296146 | 0.578621836 |  | 0.847143992 | 4.51255E-10 | 1590349416 | 0.987879 | 0.994828538 |  | 0.697105 | 0.371452 | 1.308258 | 0.263686 | 0.47158482 |
| metabolite369 | 0.025880529 | 9.29E-05 | 7.211808411 | 0.205968 | 0.366592577 |  | 25.31328 | 0.303147 | 2113.7 | 0.155148 | 0.421465998 |  | 0.377168166 | 1.65984E-10 | 857043179.6 | 0.929474 | 0.977539147 |  | 1.498852 | 0.794839 | 2.826432 | 0.213747 | 0.410835897 |
| metabolite370 | 12.30240542 | 0.031129 | 4862.074101 | 0.41245 | 0.580432576 |  | 1.726106 | 0.015362 | 193.9471 | 0.821158 | 0.928972215 |  | 37806.87871 | 5.22825E-06 | 2.73392E+14 | 0.364786 | 0.731310396 |  | 0.717411 | 0.3661 | 1.405842 | 0.335361 | 0.548370135 |
| metabolite371 | 1.422117043 | 0.005658 | 357.4375275 | 0.900842 | 0.947032206 |  | 0.511253 | 0.006595 | 39.63424 | 0.76303 | 0.902026947 |  | 68.06317711 | 5.24269E-08 | 88363000059 | 0.694188 | 0.907533437 |  | 0.867372 | 0.465656 | 1.615645 | 0.654776 | 0.802732911 |
| metabolite372 | 0.501084976 | 0.001267 | 198.1873408 | 0.821254 | 0.899019035 |  | 0.061693 | 0.000572 | 6.65918 | 0.246026 | 0.530183568 |  | 8.97315E-05 | 1.295E-14 | 621758.6288 | 0.421929 | 0.77189627 |  | 1.023782 | 0.521917 | 2.008231 | 0.945611 | 0.972875566 |
| metabolite373 | 0.000701086 | 3.38E-06 | 0.145393464 | 0.008761 | 0.035649454 |  | 3.452848 | 0.045593 | 261.4897 | 0.575728 | 0.791336134 |  | 120170.0289 | 0.000112014 | 1.2892E+14 | 0.272617 | 0.656510118 |  | 1.480135 | 0.799779 | 2.739255 | 0.214437 | 0.410835897 |
| metabolite374 | 0.003540569 | 1.73E-05 | 0.726675869 | 0.040059 | 0.114617802 |  | 0.365199 | 0.005111 | 26.09713 | 0.644652 | 0.833555093 |  | 1.096861086 | 1.21959E-09 | 986483619.9 | 0.993003 | 0.997033213 |  | 1.367372 | 0.744087 | 2.512753 | 0.315717 | 0.527891308 |
| metabolite375 | 1.403404059 | 0.005662 | 347.8564986 | 0.904313 | 0.948800188 |  | 0.839874 | 0.010935 | 64.50632 | 0.937346 | 0.975459713 |  | 42645.40058 | 3.74996E-05 | 4.84973E+13 | 0.318491 | 0.700344699 |  | 0.94058 | 0.505521 | 1.750059 | 0.84702 | 0.925206827 |
| metabolite376 | 2.667197807 | 0.011856 | 600.0508555 | 0.723244 | 0.836384485 |  | 0.07303 | 0.001053 | 5.06562 | 0.228898 | 0.511111286 |  | 6.25909E-05 | 7.75299E-14 | 50530.38647 | 0.356983 | 0.722729289 |  | 0.530913 | 0.291683 | 0.966353 | 0.040582 | 0.146355407 |
| metabolite377 | 0.026984742 | 8.14E-05 | 8.941109723 | 0.225009 | 0.387475589 |  | 0.055293 | 0.000574 | 5.330561 | 0.216834 | 0.497631469 |  | 1.619E-08 | 4.78422E-18 | 54.78789203 | 0.111911 | 0.512820569 |  | 1.75183 | 0.914795 | 3.35475 | 0.093576 | 0.253475612 |
| metabolite378 | 0.001455507 | 4.43E-06 | 0.478343241 | 0.029199 | 0.090676284 |  | 0.09397 | 0.000906 | 9.746539 | 0.320184 | 0.598590984 |  | 1387.559023 | 2.45595E-07 | 7.83942E+12 | 0.528985 | 0.825510246 |  | 0.872841 | 0.448222 | 1.699719 | 0.68995 | 0.827806035 |
| metabolite379 | 0.025380271 | 0.000105 | 6.139525126 | 0.192248 | 0.348695513 |  | 34.58558 | 0.466877 | 2562.049 | 0.109535 | 0.355528671 |  | 3455.602387 | 2.73167E-06 | 4.37139E+12 | 0.447695 | 0.785367543 |  | 1.622206 | 0.875745 | 3.004931 | 0.126857 | 0.303882925 |
| metabolite380 | 11.66172422 | 0.037158 | 3659.975347 | 0.404144 | 0.573528315 |  | 3.535644 | 0.037921 | 329.6534 | 0.5863 | 0.797565486 |  | 78870.97391 | 2.66182E-05 | 2.33699E+14 | 0.313108 | 0.696192245 |  | 0.852564 | 0.445558 | 1.631359 | 0.630914 | 0.784633083 |
| metabolite381 | 1.164733799 | 0.003414 | 397.322067 | 0.959221 | 0.980990323 |  | 34.4631 | 0.365938 | 3245.649 | 0.129732 | 0.387005594 |  | 41334781804 | 15.7756815 | 1.08304E+20 | 0.029207 | 0.315078184 |  | 1.616036 | 0.842922 | 3.098235 | 0.151161 | 0.335139305 |
| metabolite382 | 68519.56575 | 90.3786 | 51947375.07 | 0.001337 | 0.008616416 |  | 0.000771 | 3.83E-06 | 0.155294 | 0.009274 | 0.129767333 |  | 1.60821E-08 | 6.8583E-20 | 3771.115917 | 0.181856 | 0.579919061 |  | 0.289582 | 0.137095 | 0.611675 | 0.001536 | 0.016710577 |
| metabolite383 | 6679.320346 | 10.59347 | 4211397.081 | 0.008541 | 0.034866707 |  | 0.005698 | 3.31E-05 | 0.981289 | 0.051656 | 0.254388663 |  | 1.40816E-09 | 1.98675E-20 | 99.80704326 | 0.11269 | 0.513062936 |  | 0.413331 | 0.198995 | 0.858527 | 0.019564 | 0.088429298 |
| metabolite384 | 113.2018798 | 0.483269 | 26516.612 | 0.092161 | 0.211206194 |  | 0.018929 | 0.00026 | 1.379847 | 0.072555 | 0.297124077 |  | 0.000210199 | 1.69516E-13 | 260645.9947 | 0.429689 | 0.776705215 |  | 0.363896 | 0.201021 | 0.658738 | 0.001147 | 0.013861255 |
| metabolite385 | 2433.579584 | 3.942714 | 1502089.632 | 0.019092 | 0.065481775 |  | 0.010914 | 6.53E-05 | 1.823177 | 0.086407 | 0.320778781 |  | 0.001164939 | 1.62828E-14 | 83344809.44 | 0.597354 | 0.868644224 |  | 0.43273 | 0.209497 | 0.893833 | 0.025565 | 0.108175527 |
| metabolite386 | 0.000988013 | 2.38E-06 | 0.410903149 | 0.026481 | 0.084499343 |  | 12.77119 | 0.101748 | 1603.007 | 0.303793 | 0.583677462 |  | 0.212759733 | 1.42706E-11 | 3172017894 | 0.897206 | 0.96870562 |  | 2.930639 | 1.506932 | 5.699425 | 0.001981 | 0.020247661 |
| metabolite387 | 0.02625427 | 3.73E-05 | 18.47839506 | 0.278901 | 0.44961036 |  | 0.458238 | 0.002558 | 82.10334 | 0.768698 | 0.905024889 |  | 656435.8838 | 9.8976E-06 | 4.35366E+16 | 0.294354 | 0.678233572 |  | 1.354364 | 0.645987 | 2.839536 | 0.423641 | 0.625849351 |
| metabolite388 | 10.5790216 | 0.040194 | 2784.408781 | 0.408536 | 0.5773327 |  | 7.3761 | 0.091814 | 592.5751 | 0.373831 | 0.647357589 |  | 455807.2604 | 0.000312915 | 6.63952E+14 | 0.228701 | 0.621260608 |  | 0.79604 | 0.424709 | 1.492032 | 0.478177 | 0.670386936 |
| metabolite389 | 0.437008385 | 0.002186 | 87.37682521 | 0.759992 | 0.860381573 |  | 4.804806 | 0.074733 | 308.9154 | 0.461516 | 0.712136233 |  | 0.004039576 | 7.45377E-12 | 2189250.147 | 0.592228 | 0.866282943 |  | 1.331377 | 0.7346 | 2.412967 | 0.347532 | 0.559382136 |
| metabolite390 | 0.003483552 | 1.75E-05 | 0.691530913 | 0.038297 | 0.110858779 |  | 38.151 | 0.575773 | 2527.903 | 0.091557 | 0.328579908 |  | 99932.324 | 0.000140438 | 7.11094E+13 | 0.270686 | 0.655253129 |  | 1.759515 | 0.967104 | 3.201199 | 0.066907 | 0.203026447 |
| metabolite391 | 4.69803E-05 | 2.57E-07 | 0.008581737 | 0.000282 | 0.002551679 |  | 0.625177 | 0.008053 | 48.53474 | 0.832848 | 0.934576634 |  | 0.14577032 | 1.10715E-10 | 191924723.8 | 0.857678 | 0.957853167 |  | 1.815415 | 0.983753 | 3.35016 | 0.059027 | 0.186424894 |
| metabolite392 | 0.005135983 | 1.57E-05 | 1.682225772 | 0.077159 | 0.184495801 |  | 16.52457 | 0.166773 | 1637.319 | 0.234184 | 0.515235933 |  | 4.740333308 | 9.65185E-10 | 23281300850 | 0.891532 | 0.967390372 |  | 2.429194 | 1.279975 | 4.610231 | 0.007685 | 0.048598438 |
| metabolite393 | 0.002217978 | 6.15E-06 | 0.800131796 | 0.044313 | 0.12411014 |  | 1354.509 | 14.6362 | 125353.3 | 0.002293 | 0.088493313 |  | 84654.23347 | 1.18968E-05 | 6.02376E+14 | 0.329067 | 0.705196155 |  | 2.498912 | 1.299789 | 4.804289 | 0.007035 | 0.046045488 |
| metabolite394 | 9.21729E-05 | 2.44E-07 | 0.034789771 | 0.002696 | 0.014490012 |  | 23.58021 | 0.188166 | 2954.978 | 0.202422 | 0.480821359 |  | 1.88163532 | 1.19588E-10 | 29606266137 | 0.95801 | 0.9871034 |  | 3.078534 | 1.584871 | 5.979903 | 0.001221 | 0.014375745 |
| metabolite395 | 555.4320556 | 1.959924 | 157406.5144 | 0.030352 | 0.093248571 |  | 0.6397 | 0.00682 | 60.00465 | 0.84745 | 0.940012856 |  | 2028.294403 | 6.45806E-07 | 6.3703E+12 | 0.496328 | 0.810140132 |  | 0.505809 | 0.267455 | 0.956581 | 0.038306 | 0.140547174 |
| metabolite396 | 0.040775577 | 0.000116 | 14.32074876 | 0.286965 | 0.459020421 |  | 565.3134 | 6.362517 | 50228.43 | 0.006605 | 0.121537565 |  | 31.4826919 | 6.04231E-09 | 1.64037E+11 | 0.763082 | 0.938107312 |  | 2.201386 | 1.152468 | 4.204979 | 0.018544 | 0.086388888 |
| metabolite397 | 0.320850564 | 0.000797 | 129.0905011 | 0.710962 | 0.826440128 |  | 230.8794 | 2.28447 | 23333.78 | 0.022691 | 0.178026796 |  | 26841.93047 | 3.64443E-06 | 1.97696E+14 | 0.380905 | 0.740848834 |  | 1.398329 | 0.713343 | 2.741072 | 0.331021 | 0.543788165 |
| metabolite398 | 842.378868 | 2.595719 | 273374.0626 | 0.024315 | 0.079082473 |  | 0.089147 | 0.000863 | 9.205274 | 0.309107 | 0.587952672 |  | 0.482593432 | 8.34681E-11 | 2790244422 | 0.949459 | 0.986298878 |  | 0.577426 | 0.298871 | 1.115602 | 0.105002 | 0.270787859 |
| metabolite399 | 2.78970066 | 0.014378 | 541.2804252 | 0.703409 | 0.822675704 |  | 21.09003 | 0.345329 | 1288.017 | 0.148989 | 0.414467103 |  | 93.7579331 | 1.91418E-07 | 45923414633 | 0.657346 | 0.89355714 |  | 1.183397 | 0.654057 | 2.141143 | 0.578913 | 0.747771146 |
| metabolite400 | 0.022574143 | 8.16E-05 | 6.24182387 | 0.189023 | 0.344733488 |  | 29.43628 | 0.355199 | 2439.462 | 0.136265 | 0.399116999 |  | 6744253.716 | 0.003684996 | 1.23433E+16 | 0.151263 | 0.555778725 |  | 1.605925 | 0.853476 | 3.021753 | 0.144729 | 0.327293641 |
| metabolite401 | 0.128459632 | 0.000456 | 36.21765502 | 0.477378 | 0.639086946 |  | 84.44566 | 1.062442 | 6711.961 | 0.049371 | 0.249369471 |  | 25055199.25 | 0.014827584 | 4.23375E+16 | 0.118905 | 0.520349615 |  | 1.569779 | 0.834894 | 2.951516 | 0.164353 | 0.353982836 |
| metabolite402 | 3.675873292 | 0.013618 | 992.1999778 | 0.64944 | 0.782236369 |  | 0.600453 | 0.007289 | 49.46478 | 0.821128 | 0.928972215 |  | 226967.6552 | 0.000146017 | 3.52797E+14 | 0.25587 | 0.645470675 |  | 0.846768 | 0.450799 | 1.590544 | 0.606091 | 0.767908542 |
| metabolite403 | 139.4522688 | 0.392976 | 49486.29348 | 0.102134 | 0.227093771 |  | 0.284172 | 0.002653 | 30.44366 | 0.598831 | 0.80650665 |  | 1.662648587 | 2.59928E-10 | 10635263167 | 0.964877 | 0.989435728 |  | 0.627052 | 0.322869 | 1.217812 | 0.170932 | 0.362474101 |
| metabolite404 | 6.12852E-05 | 3.73E-07 | 0.010059724 | 0.000306 | 0.00275247 |  | 4.932578 | 0.070348 | 345.8564 | 0.463316 | 0.713133835 |  | 17953.32321 | 2.3016E-05 | 1.40042E+13 | 0.350435 | 0.717869012 |  | 2.158576 | 1.193695 | 3.903386 | 0.012275 | 0.065730417 |
| metabolite405 | 0.011245451 | 2.56E-05 | 4.942998282 | 0.151176 | 0.29920824 |  | 29.10584 | 0.240428 | 3523.506 | 0.171118 | 0.442862885 |  | 0.292886071 | 2.14678E-11 | 3995851365 | 0.918041 | 0.975057731 |  | 1.988513 | 1.007413 | 3.925088 | 0.050032 | 0.168177103 |
| metabolite406 | 62.93195214 | 0.259701 | 15249.93681 | 0.142055 | 0.285572067 |  | 48.20754 | 0.650373 | 3573.285 | 0.080455 | 0.310368915 |  | 969477.8271 | 0.000807218 | 1.16435E+15 | 0.198933 | 0.597241307 |  | 0.814453 | 0.436679 | 1.519041 | 0.520023 | 0.700765454 |
| metabolite407 | 0.039041085 | 0.000183 | 8.310253341 | 0.238239 | 0.402598494 |  | 2.366787 | 0.033935 | 165.0699 | 0.69154 | 0.864657959 |  | 5.709339812 | 7.19378E-09 | 4531214200 | 0.867968 | 0.962305681 |  | 1.314138 | 0.717255 | 2.407733 | 0.378458 | 0.592348165 |
| metabolite408 | 31.35905456 | 0.096297 | 10212.08648 | 0.245633 | 0.411425082 |  | 2.58817 | 0.026529 | 252.506 | 0.684855 | 0.859380301 |  | 0.008951674 | 2.26259E-12 | 35416316.02 | 0.676557 | 0.903131158 |  | 1.116602 | 0.579789 | 2.150439 | 0.742144 | 0.86020302 |
| metabolite409 | 4898.881716 | 22.2592 | 1078162.789 | 0.00255 | 0.013979423 |  | 0.026972 | 0.00034 | 2.142144 | 0.108351 | 0.355281522 |  | 4.23147E-07 | 2.68135E-16 | 667.7723369 | 0.177183 | 0.575285157 |  | 0.404757 | 0.219845 | 0.745199 | 0.004441 | 0.033908527 |
| metabolite410 | 449989.42 | 2486.995 | 81419748.4 | 3.18E-06 | 9.22E-05 |  | 1.401444 | 0.015334 | 128.087 | 0.883787 | 0.957198024 |  | 12.24137585 | 4.24772E-09 | 35278080980 | 0.822088 | 0.948494237 |  | 0.320316 | 0.174004 | 0.589655 | 0.000392 | 0.006603083 |
| metabolite411 | 0.309500268 | 0.00122 | 78.53939383 | 0.678801 | 0.8024736 |  | 282.6467 | 4.09364 | 19515.44 | 0.010237 | 0.133418413 |  | 1.31651E+11 | 163.8848105 | 1.05757E+20 | 0.015955 | 0.259782535 |  | 1.628655 | 0.878343 | 3.01991 | 0.12441 | 0.300379824 |
| metabolite412 | 0.026085515 | 0.00014 | 4.862137905 | 0.174362 | 0.32779064 |  | 24.34305 | 0.400029 | 1481.354 | 0.130626 | 0.388736613 |  | 18.71202636 | 3.77755E-08 | 9268974283 | 0.77483 | 0.940047404 |  | 1.678518 | 0.93424 | 3.015737 | 0.085969 | 0.239693797 |
| metabolite413 | 3.739486885 | 0.017134 | 816.1466335 | 0.632168 | 0.767662253 |  | 27.2339 | 0.408159 | 1817.147 | 0.12595 | 0.381081608 |  | 19128541.82 | 0.030906209 | 1.18391E+16 | 0.107347 | 0.506823236 |  | 1.077828 | 0.587335 | 1.977941 | 0.809255 | 0.901935501 |
| metabolite414 | 1.179851322 | 0.004498 | 309.5060193 | 0.953692 | 0.978206842 |  | 5.033909 | 0.063336 | 400.0932 | 0.470605 | 0.717917212 |  | 1494278.077 | 0.001139432 | 1.95963E+15 | 0.187136 | 0.586045046 |  | 1.553111 | 0.833819 | 2.892899 | 0.168124 | 0.35898076 |
| metabolite415 | 0.06690777 | 0.000334 | 13.38673908 | 0.319304 | 0.492285398 |  | 0.315715 | 0.004802 | 20.75539 | 0.590373 | 0.800466136 |  | 848.5452217 | 1.4573E-06 | 4.94085E+11 | 0.513893 | 0.819395994 |  | 1.034187 | 0.567859 | 1.883466 | 0.912685 | 0.958092754 |
| metabolite416 | 0.012694938 | 5.56E-05 | 2.90091596 | 0.117945 | 0.250827358 |  | 31.97354 | 0.444223 | 2301.336 | 0.11511 | 0.365729735 |  | 14172.79482 | 1.32677E-05 | 1.51396E+13 | 0.36942 | 0.734917241 |  | 2.08619 | 1.14115 | 3.81386 | 0.018583 | 0.086388888 |
| metabolite417 | 0.069025511 | 0.00021 | 22.66138078 | 0.36778 | 0.538819789 |  | 38.30338 | 0.413476 | 3548.327 | 0.117463 | 0.369746079 |  | 12971082.3 | 0.004070626 | 4.13325E+16 | 0.145202 | 0.546460871 |  | 1.062935 | 0.552171 | 2.04616 | 0.855403 | 0.929001775 |
| metabolite418 | 0.195896664 | 0.000749 | 51.22668909 | 0.567129 | 0.714640581 |  | 163.2551 | 2.245921 | 11866.95 | 0.021614 | 0.174688709 |  | 1538846854 | 1.410273939 | 1.67914E+18 | 0.048784 | 0.385659329 |  | 1.654886 | 0.889435 | 3.079085 | 0.114649 | 0.286361676 |
| metabolite419 | 1.7308927 | 0.007602 | 394.1298585 | 0.843324 | 0.913478275 |  | 10.43649 | 0.148463 | 733.6507 | 0.282081 | 0.569223569 |  | 147176288.7 | 0.218749609 | 9.90213E+16 | 0.072464 | 0.447917707 |  | 1.13155 | 0.614159 | 2.084813 | 0.692574 | 0.829613313 |
| metabolite420 | 6215.73411 | 21.27971 | 1815596.102 | 0.003179 | 0.016248283 |  | 1.312275 | 0.012552 | 137.1965 | 0.909003 | 0.967118379 |  | 0.003625454 | 6.71023E-13 | 19587874.43 | 0.624039 | 0.879305472 |  | 0.472606 | 0.246631 | 0.905631 | 0.025854 | 0.109029127 |
| metabolite421 | 12.291199 | 0.059186 | 2552.526192 | 0.358756 | 0.530838656 |  | 63.22235 | 0.999982 | 3997.139 | 0.052503 | 0.255694971 |  | 1580268.413 | 0.002732053 | 9.14055E+14 | 0.16835 | 0.568636202 |  | 1.079155 | 0.590378 | 1.972594 | 0.804946 | 0.900287383 |
| metabolite422 | 0.140329924 | 0.000702 | 28.0617275 | 0.469079 | 0.632081146 |  | 11.55988 | 0.180952 | 738.4885 | 0.250986 | 0.534588226 |  | 327005.9872 | 0.000648977 | 1.64771E+14 | 0.216844 | 0.607206891 |  | 1.774834 | 0.985213 | 3.197315 | 0.058663 | 0.185826725 |
| metabolite423 | 0.230153861 | 0.001144 | 46.30229184 | 0.588338 | 0.730697246 |  | 7.906454 | 0.122836 | 508.9064 | 0.332608 | 0.608034064 |  | 3202.228865 | 5.83925E-06 | 1.75609E+12 | 0.433425 | 0.779282769 |  | 1.681756 | 0.931881 | 3.035051 | 0.087169 | 0.242492193 |
| metabolite424 | 0.244084775 | 0.000685 | 86.99958738 | 0.639 | 0.774691533 |  | 195.4166 | 2.1139 | 18065.03 | 0.024267 | 0.185877348 |  | 12814.50823 | 2.7083E-06 | 6.06327E+13 | 0.407109 | 0.762519149 |  | 1.560018 | 0.808427 | 3.010363 | 0.18759 | 0.382588343 |
| metabolite425 | 0.001684802 | 5.49E-06 | 0.516827439 | 0.030921 | 0.094364527 |  | 247.1726 | 2.774952 | 22016.35 | 0.017799 | 0.162567222 |  | 26754766356 | 9.4482342 | 7.5762E+19 | 0.032745 | 0.32957416 |  | 2.352332 | 1.241349 | 4.457625 | 0.009943 | 0.056678428 |
| metabolite426 | 0.109690942 | 0.00036 | 33.42738018 | 0.450431 | 0.616083905 |  | 49.22247 | 0.570934 | 4243.663 | 0.089409 | 0.324973836 |  | 6531137.567 | 0.002747924 | 1.55229E+16 | 0.157068 | 0.561539131 |  | 1.314491 | 0.690426 | 2.502641 | 0.406986 | 0.615949425 |
| metabolite427 | 0.86528468 | 0.002538 | 294.9627685 | 0.961299 | 0.982077156 |  | 42.2815 | 0.451767 | 3957.181 | 0.108742 | 0.355281522 |  | 2954744.069 | 0.000839685 | 1.03974E+16 | 0.186744 | 0.5858046 |  | 1.578035 | 0.822681 | 3.026926 | 0.172622 | 0.364598211 |
| metabolite428 | 13.75770126 | 0.074119 | 2553.660801 | 0.327422 | 0.501056247 |  | 2.266545 | 0.036509 | 140.7114 | 0.698412 | 0.868760796 |  | 0.000163012 | 3.84172E-13 | 69169.55951 | 0.391374 | 0.750857464 |  | 1.051634 | 0.582436 | 1.898807 | 0.867675 | 0.937004424 |
| metabolite429 | 216.0427443 | 0.836148 | 55820.80069 | 0.060446 | 0.154320867 |  | 5.493601 | 0.065269 | 462.3877 | 0.452894 | 0.703511684 |  | 310.4530469 | 1.55432E-07 | 6.20087E+11 | 0.600513 | 0.871267487 |  | 0.956222 | 0.506404 | 1.805594 | 0.890466 | 0.947939724 |
| metabolite430 | 4177.491675 | 17.27847 | 1010010.675 | 0.003567 | 0.017758447 |  | 0.329186 | 0.003708 | 29.22132 | 0.628302 | 0.825647652 |  | 0.003204345 | 1.28063E-12 | 8017818.939 | 0.603979 | 0.873215145 |  | 0.612769 | 0.324439 | 1.157337 | 0.133984 | 0.310760289 |
| metabolite431 | 22.04982441 | 0.062778 | 7744.649193 | 0.303214 | 0.474779723 |  | 0.255426 | 0.00249 | 26.20609 | 0.564661 | 0.787805088 |  | 81.5025527 | 1.58467E-08 | 4.19182E+11 | 0.700436 | 0.909170666 |  | 0.640083 | 0.331425 | 1.236195 | 0.18671 | 0.382029269 |
| metabolite432 | 45.03307323 | 0.164696 | 12313.42426 | 0.186256 | 0.342007182 |  | 2.617526 | 0.030573 | 224.1004 | 0.672513 | 0.851700797 |  | 1.573380735 | 7.33422E-10 | 3375311425 | 0.967096 | 0.989787236 |  | 0.832822 | 0.440841 | 1.573337 | 0.574133 | 0.743407531 |
| metabolite433 | 49.56627863 | 0.21459 | 11448.88709 | 0.162596 | 0.313124187 |  | 58.17626 | 0.824112 | 4106.819 | 0.063988 | 0.278056389 |  | 3377.160122 | 3.11227E-06 | 3.6646E+12 | 0.445644 | 0.784097818 |  | 1.024319 | 0.551886 | 1.90117 | 0.939436 | 0.969748787 |
| metabolite434 | 0.130629525 | 0.000437 | 39.08017327 | 0.485538 | 0.644698499 |  | 8.442737 | 0.095533 | 746.1261 | 0.35285 | 0.627092503 |  | 688385.8684 | 0.000297418 | 1.5933E+15 | 0.224346 | 0.617181067 |  | 1.188458 | 0.624929 | 2.26015 | 0.599606 | 0.763326752 |
| metabolite435 | 0.005422353 | 3.24E-05 | 0.907388339 | 0.04825 | 0.131755069 |  | 19.66771 | 0.337231 | 1147.042 | 0.153809 | 0.421028033 |  | 297391.9447 | 0.000861892 | 1.02614E+14 | 0.211578 | 0.603798949 |  | 1.944531 | 1.095504 | 3.451564 | 0.02504 | 0.107397606 |
| metabolite436 | 0.141961225 | 0.000815 | 24.72930895 | 0.459952 | 0.624317225 |  | 62.17434 | 1.13841 | 3395.655 | 0.045423 | 0.239096444 |  | 576632.4229 | 0.001964335 | 1.69271E+14 | 0.185109 | 0.58220839 |  | 1.578432 | 0.886894 | 2.809183 | 0.123534 | 0.29923996 |
| metabolite437 | 1.341545204 | 0.007054 | 255.1310161 | 0.912817 | 0.954249005 |  | 184.9666 | 3.33073 | 10271.82 | 0.012232 | 0.142361591 |  | 28854228.44 | 0.081789061 | 1.01794E+16 | 0.089935 | 0.479980149 |  | 1.684013 | 0.939905 | 3.017222 | 0.082588 | 0.23308125 |
| metabolite438 | 3.601411847 | 0.009681 | 1339.820414 | 0.67217 | 0.798239357 |  | 0.095406 | 0.000918 | 9.919565 | 0.323536 | 0.599929612 |  | 8.844505821 | 1.49352E-09 | 52376489474 | 0.849759 | 0.955623187 |  | 0.695948 | 0.358324 | 1.35169 | 0.286835 | 0.499763282 |
| metabolite439 | 0.31367762 | 0.001183 | 83.19331126 | 0.684645 | 0.80702296 |  | 44.77461 | 0.583456 | 3436.018 | 0.088823 | 0.324630307 |  | 2299530.401 | 0.001672296 | 3.16202E+15 | 0.175185 | 0.573796732 |  | 1.578331 | 0.846251 | 2.943725 | 0.154083 | 0.338544557 |
| metabolite440 | 0.104293798 | 0.000607 | 17.9091325 | 0.391085 | 0.560353928 |  | 0.188241 | 0.003267 | 10.84463 | 0.42112 | 0.685629403 |  | 16.07402624 | 4.90064E-08 | 5272254881 | 0.781836 | 0.94146208 |  | 1.402904 | 0.786945 | 2.500987 | 0.25355 | 0.459435643 |
| metabolite441 | 0.096232762 | 0.000588 | 15.74047414 | 0.369982 | 0.540355917 |  | 9.721934 | 0.176975 | 534.0628 | 0.268223 | 0.555007395 |  | 2.258282213 | 8.18683E-09 | 622931722.6 | 0.934675 | 0.978919523 |  | 1.360783 | 0.766956 | 2.414389 | 0.29461 | 0.508510176 |
| metabolite442 | 2.674553753 | 0.010295 | 694.7956439 | 0.72939 | 0.839623847 |  | 0.018186 | 0.000243 | 1.362633 | 0.07153 | 0.294639472 |  | 4.22857E-09 | 3.80901E-18 | 4.69435142 | 0.072305 | 0.447917707 |  | 0.84842 | 0.45374 | 1.58641 | 0.607723 | 0.76840165 |
| metabolite443 | 18.31478384 | 0.076672 | 4374.880612 | 0.30025 | 0.47233039 |  | 138.7894 | 2.010271 | 9582.037 | 0.024323 | 0.185877348 |  | 111750.4629 | 0.000104195 | 1.19854E+14 | 0.275584 | 0.660614131 |  | 1.227371 | 0.661157 | 2.278488 | 0.517619 | 0.699011669 |
| metabolite444 | 9.21461422 | 0.028613 | 2967.511654 | 0.452587 | 0.617709038 |  | 1.593816 | 0.016707 | 152.0464 | 0.841503 | 0.938160513 |  | 0.241035717 | 6.7661E-11 | 858665819.5 | 0.899331 | 0.968957237 |  | 1.043444 | 0.543596 | 2.002912 | 0.898518 | 0.951826138 |
| metabolite445 | 154.6633603 | 0.634779 | 37683.61708 | 0.074909 | 0.180593149 |  | 31.76986 | 0.413032 | 2443.697 | 0.121389 | 0.375081938 |  | 369.2209594 | 2.40325E-07 | 5.67248E+11 | 0.584965 | 0.863553746 |  | 1.030935 | 0.550188 | 1.931755 | 0.924413 | 0.963893458 |
| metabolite446 | 0.865689932 | 0.002535 | 295.5911942 | 0.961435 | 0.982077156 |  | 1.189453 | 0.012037 | 117.5384 | 0.941118 | 0.97690349 |  | 66891265 | 0.020481878 | 2.18459E+17 | 0.109775 | 0.512129781 |  | 0.861612 | 0.446898 | 1.661174 | 0.657394 | 0.804083379 |
| metabolite447 | 47.61228438 | 0.173254 | 13084.43952 | 0.180336 | 0.335079079 |  | 0.2821 | 0.003287 | 24.20881 | 0.578575 | 0.79199206 |  | 1.45357374 | 6.61499E-10 | 3194074599 | 0.972874 | 0.991305907 |  | 0.553689 | 0.295421 | 1.037743 | 0.06779 | 0.203864842 |
| metabolite448 | 0.827237802 | 0.003487 | 196.2560782 | 0.945931 | 0.973744706 |  | 10.00182 | 0.137715 | 726.4043 | 0.294525 | 0.577076976 |  | 66993.64128 | 7.02713E-05 | 6.38688E+13 | 0.294431 | 0.678233572 |  | 1.320898 | 0.714938 | 2.440451 | 0.376135 | 0.590621951 |
| metabolite449 | 1.500476353 | 0.006363 | 353.8452703 | 0.884514 | 0.936826477 |  | 9.197238 | 0.127004 | 666.0365 | 0.31205 | 0.590825895 |  | 61.73606438 | 6.05207E-08 | 62975790666 | 0.697604 | 0.907533437 |  | 1.207234 | 0.653057 | 2.231677 | 0.549214 | 0.72120747 |
| metabolite450 | 6.157973682 | 0.030362 | 1248.940165 | 0.503826 | 0.659738065 |  | 81.46672 | 1.335471 | 4969.653 | 0.038181 | 0.222433593 |  | 885603.0178 | 0.001715168 | 4.57269E+14 | 0.18368 | 0.5811057 |  | 1.182033 | 0.649504 | 2.151181 | 0.585193 | 0.752216635 |
| metabolite451 | 1.458320165 | 0.005508 | 386.1153297 | 0.894789 | 0.943611806 |  | 0.821381 | 0.010153 | 66.45149 | 0.930205 | 0.974037858 |  | 459214.6492 | 0.000328696 | 6.4156E+14 | 0.227522 | 0.620300106 |  | 0.920494 | 0.4911 | 1.725329 | 0.796536 | 0.894656479 |
| metabolite452 | 350.2894799 | 1.751258 | 70065.4903 | 0.03235 | 0.097603489 |  | 0.853349 | 0.012057 | 60.3945 | 0.941958 | 0.97690349 |  | 1.351138487 | 1.6048E-09 | 1137575277 | 0.977154 | 0.991698663 |  | 0.676621 | 0.369499 | 1.239016 | 0.208292 | 0.406186691 |
| metabolite453 | 366.7744256 | 1.64922 | 81567.94726 | 0.034425 | 0.102038897 |  | 3.029239 | 0.039576 | 231.8621 | 0.617519 | 0.819310952 |  | 0.084304971 | 6.73789E-11 | 105482935 | 0.817413 | 0.946906762 |  | 0.748001 | 0.402846 | 1.388885 | 0.359784 | 0.57302879 |
| metabolite454 | 171.2783502 | 0.807563 | 36326.91707 | 0.062482 | 0.158141155 |  | 23.73348 | 0.340429 | 1654.612 | 0.146455 | 0.411005424 |  | 622918.9805 | 0.000759528 | 5.10881E+14 | 0.205293 | 0.597241307 |  | 0.926872 | 0.502207 | 1.710631 | 0.808541 | 0.901862093 |
| metabolite455 | 2.539757046 | 0.009366 | 688.7348773 | 0.744992 | 0.84980371 |  | 5.103706 | 0.062436 | 417.1916 | 0.469681 | 0.717917212 |  | 8407.903311 | 5.06613E-06 | 1.3954E+13 | 0.405896 | 0.762519149 |  | 0.867043 | 0.461391 | 1.629341 | 0.658441 | 0.804696336 |
| metabolite456 | 106.8432834 | 0.451244 | 25297.80244 | 0.096805 | 0.218448888 |  | 3.523372 | 0.045348 | 273.7518 | 0.571794 | 0.790542575 |  | 0.21495666 | 1.58256E-10 | 291973189.1 | 0.886327 | 0.966803451 |  | 0.851329 | 0.456675 | 1.587041 | 0.613497 | 0.772805844 |
| metabolite457 | 190.0292367 | 0.779228 | 46342.16132 | 0.063972 | 0.160404273 |  | 2.3726 | 0.029331 | 191.9188 | 0.700625 | 0.869142035 |  | 0.395015394 | 2.43064E-10 | 641958957.6 | 0.931751 | 0.977587293 |  | 0.978778 | 0.521907 | 1.835589 | 0.946814 | 0.973843434 |
| metabolite458 | 14.8047986 | 0.070449 | 3111.21138 | 0.325442 | 0.499055334 |  | 26.91315 | 0.40968 | 1768.006 | 0.125904 | 0.381081608 |  | 63339.03789 | 9.6463E-05 | 4.15893E+13 | 0.288124 | 0.67078217 |  | 0.992853 | 0.542162 | 1.818198 | 0.981504 | 0.990041471 |
| metabolite459 | 0.852548828 | 0.002513 | 289.2334661 | 0.957302 | 0.980554024 |  | 14.19578 | 0.148265 | 1359.188 | 0.256792 | 0.5411434 |  | 120736.8919 | 3.26825E-05 | 4.46031E+14 | 0.300108 | 0.684940846 |  | 1.085376 | 0.563137 | 2.091924 | 0.807124 | 0.901365747 |
| metabolite460 | 50.94905372 | 0.197494 | 13143.71793 | 0.168079 | 0.319376231 |  | 1.555462 | 0.018915 | 127.9129 | 0.844686 | 0.938792681 |  | 553.9189863 | 3.28362E-07 | 9.34416E+11 | 0.561239 | 0.848373622 |  | 0.671522 | 0.358897 | 1.256466 | 0.215472 | 0.411961353 |
| metabolite461 | 8.98956993 | 0.034151 | 2366.29562 | 0.441552 | 0.609371137 |  | 4.083003 | 0.050509 | 330.0582 | 0.531451 | 0.764725841 |  | 10948.93118 | 7.08019E-06 | 1.69316E+13 | 0.390786 | 0.750322822 |  | 1.216964 | 0.649203 | 2.281258 | 0.541472 | 0.713960117 |
| metabolite462 | 0.327037618 | 0.001162 | 92.06582755 | 0.698463 | 0.818439276 |  | 183.8708 | 2.403741 | 14064.94 | 0.02021 | 0.167410842 |  | 92760947.99 | 0.059375218 | 1.44919E+17 | 0.092206 | 0.484875813 |  | 1.394554 | 0.740707 | 2.625572 | 0.305146 | 0.51867642 |
| metabolite463 | 49.3258973 | 0.207232 | 11740.68527 | 0.165416 | 0.316422029 |  | 15.7604 | 0.210347 | 1180.858 | 0.213164 | 0.4913029 |  | 7659852.403 | 0.007353652 | 7.9788E+15 | 0.137417 | 0.536231553 |  | 0.884628 | 0.475225 | 1.64673 | 0.699737 | 0.832910141 |
| metabolite464 | 0.33943042 | 0.000659 | 174.7286553 | 0.735114 | 0.84432316 |  | 0.00773 | 6.14E-05 | 0.973016 | 0.051203 | 0.254368744 |  | 4.33368E-06 | 2.38755E-16 | 78661.54359 | 0.307757 | 0.691419261 |  | 1.406508 | 0.6979 | 2.834597 | 0.342141 | 0.554932217 |
| metabolite465 | 58.7739492 | 0.299166 | 11546.70314 | 0.133357 | 0.27311593 |  | 1.54355 | 0.023149 | 102.9239 | 0.839836 | 0.938160513 |  | 0.073044582 | 1.15942E-10 | 46018630.31 | 0.800637 | 0.9460629 |  | 0.786038 | 0.431726 | 1.431129 | 0.432678 | 0.634400237 |
| metabolite466 | 0.061325852 | 7.42E-05 | 50.70868818 | 0.417109 | 0.585657715 |  | 25.38119 | 0.130375 | 4941.176 | 0.231739 | 0.512810891 |  | 22.4106285 | 1.72297E-10 | 2.91495E+12 | 0.8122 | 0.946496748 |  | 1.505797 | 0.707667 | 3.204086 | 0.29033 | 0.503523289 |
| metabolite467 | 12.95698783 | 0.04415 | 3802.588624 | 0.378787 | 0.548451113 |  | 6.631657 | 0.07546 | 582.8106 | 0.409208 | 0.676533319 |  | 3241.784246 | 1.33581E-06 | 7.86725E+12 | 0.46498 | 0.792880411 |  | 0.843015 | 0.443867 | 1.601097 | 0.602854 | 0.764589917 |
| metabolite468 | 0.002236642 | 1.07E-05 | 0.468531467 | 0.027215 | 0.086099073 |  | 10.98978 | 0.152228 | 793.3859 | 0.274648 | 0.563632158 |  | 0.004286509 | 4.2424E-12 | 4331070.996 | 0.607286 | 0.874215598 |  | 1.924119 | 1.052512 | 3.517522 | 0.035696 | 0.135865714 |
| metabolite469 | 0.007445564 | 2.31E-05 | 2.402593373 | 0.099216 | 0.222667813 |  | 9.499483 | 0.09683 | 931.9453 | 0.338069 | 0.613183543 |  | 5561.267976 | 1.3214E-06 | 2.34052E+13 | 0.447247 | 0.785120072 |  | 1.737089 | 0.906225 | 3.329723 | 0.099055 | 0.261157657 |
| metabolite470 | 32.56836761 | 0.091974 | 11532.59698 | 0.247254 | 0.413128722 |  | 0.095432 | 0.000931 | 9.778114 | 0.322071 | 0.599401439 |  | 104.8667077 | 1.92477E-08 | 5.71341E+11 | 0.684958 | 0.903557902 |  | 0.780625 | 0.402029 | 1.515749 | 0.466003 | 0.660809251 |
| metabolite471 | 0.096222155 | 0.000394 | 23.47867774 | 0.405675 | 0.574387393 |  | 34.49369 | 0.471764 | 2522.053 | 0.108733 | 0.355281522 |  | 58938.30287 | 5.18975E-05 | 6.69343E+13 | 0.30406 | 0.688448386 |  | 1.158577 | 0.622914 | 2.154874 | 0.642905 | 0.794214079 |
| metabolite472 | 18.82745492 | 0.08706 | 4071.576472 | 0.286907 | 0.459020421 |  | 108.8984 | 1.691095 | 7012.541 | 0.029356 | 0.200006549 |  | 24236.90973 | 3.20033E-05 | 1.83553E+13 | 0.335237 | 0.710418618 |  | 0.988572 | 0.537831 | 1.817068 | 0.970545 | 0.984337009 |
| metabolite473 | 0.011565539 | 2.49E-05 | 5.371266364 | 0.157413 | 0.306323117 |  | 1593.506 | 14.74651 | 172194.1 | 0.002559 | 0.089719453 |  | 3.5472E+12 | 394.3015784 | 3.19111E+22 | 0.014988 | 0.253980523 |  | 2.177633 | 1.100138 | 4.310446 | 0.02749 | 0.114240546 |
| metabolite474 | 26.47364333 | 0.07614 | 9204.848952 | 0.274842 | 0.445024204 |  | 0.851254 | 0.008284 | 87.47899 | 0.945798 | 0.977627498 |  | 0.00212102 | 4.29058E-13 | 10485125.3 | 0.58991 | 0.865026277 |  | 0.794437 | 0.410082 | 1.539033 | 0.496613 | 0.685104404 |
| metabolite475 | 0.010105602 | 3.75E-05 | 2.722326147 | 0.110405 | 0.23906754 |  | 1479.731 | 21.19398 | 103312.6 | 0.001036 | 0.075024912 |  | 27785.8921 | 1.38004E-05 | 5.59444E+13 | 0.351227 | 0.718921174 |  | 2.865539 | 1.561921 | 5.257191 | 0.000936 | 0.012274952 |
| metabolite476 | 20.54920974 | 0.07043 | 5995.632127 | 0.298833 | 0.470748957 |  | 0.01404 | 0.000169 | 1.167532 | 0.061187 | 0.270639361 |  | 5.78611E-06 | 2.52304E-15 | 13269.33665 | 0.275141 | 0.660445997 |  | 0.770348 | 0.405941 | 1.461877 | 0.426427 | 0.628962904 |
| metabolite477 | 0.019174168 | 4.27E-05 | 8.60934987 | 0.207073 | 0.367679196 |  | 254.9227 | 2.243842 | 28961.74 | 0.023631 | 0.183844693 |  | 5.609627459 | 3.98658E-10 | 78934534257 | 0.885253 | 0.966756678 |  | 2.016601 | 1.021163 | 3.982402 | 0.045758 | 0.157595802 |
| metabolite478 | 39.41796367 | 0.18859 | 8238.915352 | 0.180408 | 0.335079079 |  | 44.01154 | 0.671762 | 2883.486 | 0.07888 | 0.308874138 |  | 159274.3928 | 0.00023352 | 1.08635E+14 | 0.250888 | 0.641493072 |  | 0.924779 | 0.504242 | 1.696045 | 0.800955 | 0.89693963 |
| metabolite479 | 0.041636324 | 6.36E-05 | 27.27184523 | 0.338743 | 0.512542019 |  | 7.661136 | 0.046083 | 1273.65 | 0.436781 | 0.695686251 |  | 246755523.7 | 0.005786736 | 1.0522E+19 | 0.124608 | 0.527043366 |  | 1.377309 | 0.663048 | 2.860998 | 0.392571 | 0.60568737 |
| metabolite480 | 0.060718077 | 0.000174 | 21.2478952 | 0.350597 | 0.522498898 |  | 88.73929 | 0.932964 | 8440.481 | 0.056141 | 0.261731894 |  | 1007130738 | 0.278858652 | 3.63737E+18 | 0.067518 | 0.436748477 |  | 1.521164 | 0.787937 | 2.936709 | 0.21398 | 0.410835897 |
| metabolite481 | 16.38154765 | 0.039177 | 6849.875987 | 0.365851 | 0.536607347 |  | 1.399689 | 0.011869 | 165.0668 | 0.890362 | 0.95849259 |  | 11805.69698 | 1.2734E-06 | 1.09451E+14 | 0.42498 | 0.773088432 |  | 0.642512 | 0.32635 | 1.264966 | 0.203238 | 0.4005389 |
| metabolite482 | 1.593697259 | 0.007921 | 320.6624789 | 0.863584 | 0.925574404 |  | 35.39983 | 0.572528 | 2188.799 | 0.092884 | 0.330166375 |  | 9299.665725 | 1.76393E-05 | 4.9029E+12 | 0.374434 | 0.736141064 |  | 1.124116 | 0.61868 | 2.042471 | 0.70171 | 0.834101676 |
| metabolite483 | 10.77769457 | 0.05637 | 2060.653325 | 0.376978 | 0.546613589 |  | 79.86093 | 1.36305 | 4679.042 | 0.037181 | 0.220921127 |  | 12421977.83 | 0.031363443 | 4.91992E+15 | 0.108666 | 0.511030336 |  | 0.996285 | 0.55016 | 1.804175 | 0.990222 | 0.993652937 |
| metabolite484 | 0.149056264 | 0.000468 | 47.42549672 | 0.518707 | 0.672481345 |  | 344.3708 | 4.167147 | 28458.61 | 0.010775 | 0.136475362 |  | 39059575.16 | 0.01482345 | 1.02921E+17 | 0.117075 | 0.519645256 |  | 1.362636 | 0.712947 | 2.604372 | 0.351184 | 0.562406492 |
| metabolite485 | 19942.41356 | 81.43503 | 4883646.237 | 0.000611 | 0.004689462 |  | 1.380474 | 0.014334 | 132.9514 | 0.890208 | 0.95849259 |  | 2.0478E-07 | 6.62332E-17 | 633.1387941 | 0.169927 | 0.568636202 |  | 0.637466 | 0.333443 | 1.21869 | 0.176014 | 0.36903245 |
| metabolite486 | 8758.626133 | 29.63748 | 2588395.873 | 0.00225 | 0.01276997 |  | 0.279471 | 0.002628 | 29.7233 | 0.593427 | 0.802260772 |  | 3.15644E-06 | 5.77648E-16 | 17247.74147 | 0.270593 | 0.655253129 |  | 0.537957 | 0.27849 | 1.039165 | 0.067611 | 0.203654718 |
| metabolite487 | 47.33019972 | 0.198928 | 11261.10227 | 0.169872 | 0.322287716 |  | 5.621467 | 0.073734 | 428.5775 | 0.436551 | 0.695686251 |  | 7240815681 | 8.720830908 | 6.01197E+18 | 0.032397 | 0.327586101 |  | 0.972033 | 0.522055 | 1.809863 | 0.928895 | 0.966583064 |
| metabolite488 | 3727.255974 | 18.68187 | 743632.0085 | 0.00292 | 0.015218843 |  | 10.39101 | 0.138361 | 780.3706 | 0.290368 | 0.573923383 |  | 174.0347002 | 1.42843E-07 | 2.12038E+11 | 0.629797 | 0.879629292 |  | 0.719706 | 0.387977 | 1.335069 | 0.299062 | 0.512382264 |
| metabolite489 | 150.2838098 | 0.589192 | 38332.5566 | 0.078989 | 0.187902892 |  | 16.24376 | 0.200528 | 1315.827 | 0.216359 | 0.497157176 |  | 21.53852302 | 1.1604E-08 | 39978268331 | 0.778522 | 0.940154348 |  | 0.932582 | 0.495277 | 1.756007 | 0.829248 | 0.915602351 |
| metabolite490 | 7.551613296 | 0.025638 | 2224.288962 | 0.48727 | 0.646268737 |  | 100.5127 | 1.229078 | 8219.818 | 0.042543 | 0.235634656 |  | 699.2588013 | 2.8723E-07 | 1.70234E+12 | 0.553723 | 0.843207133 |  | 1.211935 | 0.638527 | 2.300275 | 0.557776 | 0.727815953 |
| metabolite491 | 6213607.39 | 32751.1 | 1178858636 | 5.17E-08 | 5.26E-06 |  | 1.966527 | 0.017498 | 221.0133 | 0.779457 | 0.911331607 |  | 0.00702696 | 9.06219E-13 | 54488127.62 | 0.67039 | 0.901709343 |  | 0.321366 | 0.169142 | 0.610588 | 0.000751 | 0.010595707 |
| metabolite492 | 35384.69678 | 166.8252 | 7505322.957 | 0.000211 | 0.002076971 |  | 2.496776 | 0.02814 | 221.5335 | 0.690061 | 0.864005738 |  | 0.812394904 | 3.19289E-10 | 2067046952 | 0.985032 | 0.994410456 |  | 0.624844 | 0.330759 | 1.180407 | 0.15017 | 0.33409124 |
| metabolite493 | 357.4645528 | 1.141539 | 111937.43 | 0.047381 | 0.130051307 |  | 4.703083 | 0.047398 | 466.6671 | 0.51059 | 0.748932249 |  | 0.26300221 | 5.86857E-11 | 1178654735 | 0.906444 | 0.970375657 |  | 0.813761 | 0.421517 | 1.571009 | 0.540431 | 0.713461462 |
| metabolite494 | 2082.06843 | 2.104031 | 2060334.732 | 0.032031 | 0.096815929 |  | 9.713617 | 0.038554 | 2447.32 | 0.422011 | 0.68573328 |  | 18.1285569 | 4.37764E-11 | 7.50734E+12 | 0.832257 | 0.952506813 |  | 0.698024 | 0.316664 | 1.538657 | 0.374609 | 0.590056098 |
| metabolite495 | 13.40717337 | 0.010557 | 17027.41763 | 0.478025 | 0.639303846 |  | 1.299659 | 0.004616 | 365.928 | 0.927594 | 0.974037858 |  | 384.207725 | 5.9628E-10 | 2.47561E+14 | 0.668777 | 0.900846102 |  | 1.045215 | 0.466459 | 2.342061 | 0.914641 | 0.958407831 |
| metabolite496 | 47.66074441 | 0.180904 | 12556.61729 | 0.176974 | 0.330356671 |  | 0.058153 | 0.000718 | 4.707021 | 0.207102 | 0.485504903 |  | 1.38671E-06 | 8.57632E-16 | 2242.18438 | 0.215083 | 0.605810552 |  | 0.708163 | 0.377245 | 1.329362 | 0.28517 | 0.497539178 |
| metabolite497 | 8956.320269 | 36.00857 | 2227683.011 | 0.001612 | 0.009836635 |  | 1.343066 | 0.014286 | 126.2693 | 0.89898 | 0.962526365 |  | 0.052295776 | 1.58719E-11 | 172307770.6 | 0.792342 | 0.944703928 |  | 0.682764 | 0.357835 | 1.302741 | 0.24948 | 0.454279737 |
| metabolite498 | 2.335984288 | 0.002777 | 1965.253253 | 0.805433 | 0.888250662 |  | 27.10741 | 0.139538 | 5266.02 | 0.222256 | 0.50384446 |  | 1.78157E+11 | 2.158308524 | 1.47059E+22 | 0.045793 | 0.3810281 |  | 1.162303 | 0.544486 | 2.481144 | 0.698211 | 0.831848922 |
| metabolite499 | 427.1887415 | 0.880915 | 207159.8906 | 0.057448 | 0.149292994 |  | 0.072435 | 0.000526 | 9.979745 | 0.298494 | 0.579650755 |  | 0.001822875 | 7.97627E-14 | 41659465.98 | 0.605289 | 0.874215598 |  | 0.657657 | 0.325366 | 1.329312 | 0.245641 | 0.449493496 |
| metabolite500 | 2014748.362 | 6744.278 | 601874817.1 | 2.24E-06 | 7.47E-05 |  | 25.73002 | 0.186033 | 3558.701 | 0.199285 | 0.477435637 |  | 42846851339 | 2.602127287 | 7.0552E+20 | 0.043757 | 0.374151151 |  | 0.308809 | 0.157083 | 0.607086 | 0.000915 | 0.012180675 |
| metabolite501 | 1.129851985 | 0.00386 | 330.7008007 | 0.966467 | 0.984236643 |  | 7.38951 | 0.085737 | 636.8913 | 0.380958 | 0.652995742 |  | 1.28474E+12 | 1030.698593 | 1.60139E+21 | 0.010323 | 0.209346746 |  | 0.843204 | 0.445098 | 1.597386 | 0.601888 | 0.764149034 |
| metabolite502 | 0.65991606 | 0.002971 | 146.5615212 | 0.880426 | 0.935105681 |  | 3.862018 | 0.055219 | 270.1117 | 0.534247 | 0.766366251 |  | 74.14562416 | 9.14141E-08 | 60139260632 | 0.681558 | 0.903557902 |  | 0.898718 | 0.48913 | 1.651289 | 0.731459 | 0.853062678 |
| metabolite503 | 3.1325E-05 | 9.7E-08 | 0.010118588 | 0.000631 | 0.004796696 |  | 41.80817 | 0.363137 | 4813.395 | 0.126 | 0.381081608 |  | 148.8077463 | 1.34957E-08 | 1.6408E+12 | 0.672361 | 0.902331541 |  | 3.089619 | 1.607691 | 5.93755 | 0.000986 | 0.012532525 |
| metabolite504 | 0.345539911 | 0.001244 | 95.95861128 | 0.711961 | 0.827203038 |  | 4.667784 | 0.055945 | 389.4596 | 0.496301 | 0.736708633 |  | 391.4728839 | 2.0693E-07 | 7.40592E+11 | 0.584942 | 0.863553746 |  | 1.296203 | 0.688742 | 2.439437 | 0.423017 | 0.625673592 |
| metabolite505 | 0.000940841 | 2.48E-06 | 0.356409121 | 0.023283 | 0.076599628 |  | 12.31034 | 0.105189 | 1440.682 | 0.303764 | 0.583677462 |  | 11.13085429 | 1.051E-09 | 1.17884E+11 | 0.838252 | 0.954234322 |  | 2.700302 | 1.396924 | 5.219776 | 0.00383 | 0.03063876 |
| metabolite506 | 0.045275362 | 0.000149 | 13.71867513 | 0.290685 | 0.462181116 |  | 0.069296 | 0.000774 | 6.20489 | 0.246909 | 0.530560369 |  | 0.12037616 | 4.04394E-11 | 358324740.5 | 0.84948 | 0.955623187 |  | 0.913466 | 0.478477 | 1.743907 | 0.784333 | 0.887079912 |
| metabolite507 | 0.422105012 | 0.000808 | 220.4602506 | 0.787566 | 0.876095853 |  | 125.6807 | 0.986913 | 16005.09 | 0.053136 | 0.255757554 |  | 467187066.6 | 0.029301946 | 7.44878E+18 | 0.098637 | 0.492678215 |  | 1.549148 | 0.768946 | 3.120975 | 0.223242 | 0.42124391 |
| metabolite508 | 889.062393 | 3.056628 | 258596.0844 | 0.020749 | 0.070127589 |  | 24.74026 | 0.264754 | 2311.886 | 0.168545 | 0.439205618 |  | 18.76362661 | 4.86334E-09 | 72393420894 | 0.795085 | 0.945629989 |  | 0.962825 | 0.500331 | 1.852841 | 0.909895 | 0.957528306 |
| metabolite509 | 1.496896556 | 0.004419 | 507.0676547 | 0.892282 | 0.94284104 |  | 28.58727 | 0.303648 | 2691.377 | 0.150998 | 0.418173158 |  | 91603.83131 | 2.47762E-05 | 3.38682E+14 | 0.311622 | 0.695402541 |  | 1.034315 | 0.536633 | 1.993556 | 0.919908 | 0.961357068 |
| metabolite510 | 0.068450102 | 4.61E-05 | 101.6952118 | 0.473255 | 0.635861417 |  | 0.353153 | 0.001111 | 112.2255 | 0.723939 | 0.879678186 |  | 28696654.34 | 2.87676E-05 | 2.86259E+19 | 0.225723 | 0.618474536 |  | 1.272231 | 0.55844 | 2.898382 | 0.567708 | 0.738171789 |
| metabolite511 | 5.709288974 | 0.016985 | 1919.073339 | 0.558438 | 0.70849287 |  | 3.434626 | 0.03514 | 335.7062 | 0.598706 | 0.80650665 |  | 533137.2384 | 0.000148747 | 1.91087E+15 | 0.242584 | 0.635756125 |  | 1.016331 | 0.527243 | 1.959113 | 0.961502 | 0.981510624 |
| metabolite512 | 27.70232604 | 0.082177 | 9338.554725 | 0.265763 | 0.433897202 |  | 0.679678 | 0.006775 | 68.19051 | 0.869852 | 0.950816083 |  | 1626.491026 | 3.735E-07 | 7.08292E+12 | 0.515119 | 0.819395994 |  | 0.674175 | 0.350125 | 1.298144 | 0.240751 | 0.444712137 |
| metabolite513 | 0.014950259 | 5.68E-05 | 3.934764921 | 0.142181 | 0.285671239 |  | 1.781102 | 0.02122 | 149.494 | 0.798896 | 0.921001331 |  | 2980.814082 | 1.62815E-06 | 5.45727E+12 | 0.463781 | 0.792880411 |  | 1.365555 | 0.726395 | 2.567116 | 0.335435 | 0.548370135 |
| metabolite514 | 11.0852438 | 0.054706 | 2246.242543 | 0.376614 | 0.546583647 |  | 2.340615 | 0.0353 | 155.1966 | 0.691835 | 0.864735355 |  | 1.60579E-05 | 2.85964E-14 | 9017.064446 | 0.285151 | 0.668895894 |  | 0.975346 | 0.535079 | 1.777868 | 0.935195 | 0.967936596 |
| metabolite515 | 0.043036905 | 0.000147 | 12.575143 | 0.27984 | 0.450610465 |  | 299.7408 | 3.802227 | 23629.45 | 0.011831 | 0.141172602 |  | 14622.77233 | 6.02196E-06 | 3.55076E+13 | 0.386279 | 0.744665851 |  | 1.70722 | 0.904584 | 3.222032 | 0.101658 | 0.265110815 |
| metabolite516 | 0.007951562 | 3.37E-05 | 1.878464551 | 0.085715 | 0.200870984 |  | 598.7054 | 9.016077 | 39756.56 | 0.003465 | 0.103039496 |  | 12057264.48 | 0.010928732 | 1.33023E+16 | 0.127664 | 0.530263915 |  | 2.328121 | 1.272973 | 4.257865 | 0.007088 | 0.046188927 |
| metabolite517 | 0.01230846 | 2.91E-05 | 5.200713101 | 0.156815 | 0.305640099 |  | 123.068 | 1.096812 | 13808.88 | 0.048121 | 0.246314899 |  | 131705759.4 | 0.014672292 | 1.18226E+18 | 0.112677 | 0.513062936 |  | 1.522337 | 0.769088 | 3.01332 | 0.230252 | 0.42981097 |
| metabolite518 | 0.174194934 | 0.000799 | 37.98685807 | 0.526024 | 0.678039179 |  | 346.7271 | 5.710245 | 21053.33 | 0.006177 | 0.118151077 |  | 382040157.8 | 0.669083727 | 2.18141E+17 | 0.057305 | 0.408172365 |  | 1.772482 | 0.974464 | 3.224021 | 0.063386 | 0.195207369 |
| metabolite519 | 1.161215419 | 0.00501 | 269.1646103 | 0.957196 | 0.980554024 |  | 129.5811 | 1.959127 | 8570.792 | 0.024861 | 0.186382274 |  | 370969481.4 | 0.532419496 | 2.58477E+17 | 0.060117 | 0.415543963 |  | 1.183457 | 0.641344 | 2.183804 | 0.591039 | 0.75737034 |
| metabolite520 | 6.050176349 | 0.013627 | 2686.140147 | 0.563904 | 0.712627998 |  | 12.55501 | 0.104967 | 1501.699 | 0.302205 | 0.583000257 |  | 2423920.271 | 0.000240858 | 2.43936E+16 | 0.213557 | 0.604329586 |  | 0.818295 | 0.411814 | 1.625991 | 0.568203 | 0.738305651 |
| metabolite521 | 8652.032294 | 41.27755 | 1813520.059 | 0.001202 | 0.007994592 |  | 0.045726 | 0.000575 | 3.636658 | 0.169822 | 0.441632035 |  | 1.341936023 | 7.58045E-10 | 2375573014 | 0.978452 | 0.991698663 |  | 0.422564 | 0.229447 | 0.778221 | 0.006672 | 0.04436987 |
| metabolite522 | 8.763550195 | 0.041155 | 1866.098469 | 0.429134 | 0.597567016 |  | 35.07668 | 0.535865 | 2296.049 | 0.098221 | 0.340971204 |  | 974.4373218 | 1.36655E-06 | 6.94834E+11 | 0.509546 | 0.817874416 |  | 0.847241 | 0.462754 | 1.551183 | 0.592184 | 0.75831492 |
| metabolite523 | 0.0150568 | 4.15E-05 | 5.461758934 | 0.165683 | 0.316769901 |  | 247.3329 | 2.567336 | 23827.64 | 0.019788 | 0.165250229 |  | 186.8790931 | 2.9601E-08 | 1.17982E+12 | 0.6505 | 0.890878737 |  | 2.787017 | 1.466721 | 5.295801 | 0.002238 | 0.021910817 |
| metabolite524 | 3.36292756 | 0.009774 | 1157.086789 | 0.684806 | 0.80702296 |  | 344.9113 | 3.938151 | 30208.04 | 0.01179 | 0.141133863 |  | 150.3380899 | 3.47653E-08 | 6.50118E+11 | 0.658752 | 0.894812883 |  | 1.258416 | 0.652359 | 2.427515 | 0.494333 | 0.68364345 |
| metabolite525 | 0.021994393 | 3.85E-05 | 12.5809079 | 0.241193 | 0.405577437 |  | 12.71092 | 0.084921 | 1902.571 | 0.321923 | 0.599401439 |  | 69972593726 | 3.151840091 | 1.55343E+21 | 0.042281 | 0.369611019 |  | 1.495481 | 0.73105 | 3.059251 | 0.272801 | 0.482308416 |
| metabolite526 | 3.658418656 | 0.013515 | 990.3209096 | 0.650801 | 0.782860318 |  | 188.7434 | 2.546714 | 13988.25 | 0.018748 | 0.164863662 |  | 842691875.8 | 0.669666701 | 1.06042E+18 | 0.057107 | 0.407641201 |  | 1.294656 | 0.689764 | 2.430013 | 0.423188 | 0.625677562 |
| metabolite527 | 0.001690045 | 9.31E-06 | 0.306859612 | 0.01782 | 0.06211656 |  | 1.199669 | 0.017967 | 80.10135 | 0.93247 | 0.974816822 |  | 3.22302E-05 | 5.55463E-14 | 18701.21315 | 0.317281 | 0.700308709 |  | 1.399526 | 0.769826 | 2.544303 | 0.272751 | 0.482308416 |
| metabolite528 | 221.2696116 | 0.623451 | 78531.06426 | 0.074214 | 0.179888192 |  | 47.06439 | 0.456401 | 4853.317 | 0.106281 | 0.353481876 |  | 0.3777648 | 5.60243E-11 | 2547220648 | 0.932964 | 0.97828249 |  | 0.988654 | 0.505391 | 1.934021 | 0.973471 | 0.985146606 |
| metabolite529 | 848.2016855 | 2.870142 | 250665.6513 | 0.021986 | 0.073242117 |  | 31.30186 | 0.333345 | 2939.318 | 0.140125 | 0.402478569 |  | 43240.04411 | 1.15573E-05 | 1.61777E+14 | 0.344601 | 0.715221003 |  | 0.736504 | 0.383067 | 1.416041 | 0.361131 | 0.574435497 |
| metabolite530 | 0.01650187 | 4.1E-05 | 6.63856725 | 0.182541 | 0.336930729 |  | 24.75032 | 0.219993 | 2784.539 | 0.185707 | 0.460976804 |  | 8619580.125 | 0.001107197 | 6.71038E+16 | 0.172117 | 0.571122218 |  | 1.467761 | 0.745661 | 2.889146 | 0.269139 | 0.47856902 |
| metabolite531 | 0.052512677 | 0.000181 | 15.23025775 | 0.310601 | 0.484183577 |  | 335.3839 | 4.314851 | 26068.65 | 0.010071 | 0.133001993 |  | 87220419.23 | 0.045549853 | 1.67013E+17 | 0.09641 | 0.492678215 |  | 1.395818 | 0.737007 | 2.643542 | 0.308315 | 0.520914323 |
| metabolite532 | 0.410539806 | 0.000953 | 176.8535903 | 0.774128 | 0.867912169 |  | 147.4096 | 1.358338 | 15997.19 | 0.039079 | 0.223736893 |  | 823857573.5 | 0.110252525 | 6.15624E+18 | 0.079489 | 0.460912725 |  | 1.096103 | 0.553491 | 2.170662 | 0.792868 | 0.892475506 |
| metabolite533 | 1816.705161 | 6.370941 | 518042.3889 | 0.010531 | 0.041269431 |  | 5.41005 | 0.055789 | 524.6334 | 0.470978 | 0.717917212 |  | 41066629.26 | 0.01290796 | 1.30653E+17 | 0.11918 | 0.520940742 |  | 0.536703 | 0.281421 | 1.023554 | 0.061458 | 0.190695632 |
| metabolite534 | 0.352176512 | 0.001099 | 112.8848787 | 0.723632 | 0.836572439 |  | 8.776285 | 0.094791 | 812.5594 | 0.349171 | 0.623385672 |  | 466352.0715 | 0.000159056 | 1.36734E+15 | 0.243067 | 0.635907497 |  | 1.004256 | 0.524111 | 1.924269 | 0.98981 | 0.993652937 |
| metabolite535 | 156.4457623 | 0.517025 | 47338.71479 | 0.085755 | 0.200870984 |  | 35.14324 | 0.386394 | 3196.344 | 0.124763 | 0.379815411 |  | 4499353.519 | 0.001519366 | 1.33241E+16 | 0.17135 | 0.569275441 |  | 0.760339 | 0.396892 | 1.456606 | 0.410541 | 0.618089269 |
| metabolite536 | 0.183827822 | 0.000554 | 61.01427752 | 0.568551 | 0.715372941 |  | 48.54773 | 0.528423 | 4460.219 | 0.095102 | 0.333421024 |  | 6806.893278 | 1.85149E-06 | 2.50252E+13 | 0.433899 | 0.779379804 |  | 1.359946 | 0.708309 | 2.611083 | 0.357611 | 0.570302477 |
| metabolite537 | 105.2152057 | 0.273598 | 40461.66219 | 0.128071 | 0.266194087 |  | 25.05259 | 0.228254 | 2749.706 | 0.181783 | 0.45686231 |  | 55852.63539 | 7.28047E-06 | 4.28477E+14 | 0.348618 | 0.717869012 |  | 0.756898 | 0.385178 | 1.487349 | 0.420741 | 0.625020256 |
| metabolite538 | 0.022667548 | 0.000104 | 4.929781618 | 0.170654 | 0.322781235 |  | 3.364874 | 0.047126 | 240.2558 | 0.578522 | 0.79199206 |  | 0.075097545 | 8.35549E-11 | 67496199.77 | 0.806035 | 0.9460629 |  | 1.712696 | 0.936979 | 3.130622 | 0.083147 | 0.234288739 |
| metabolite539 | 25.88401656 | 0.087781 | 7632.45043 | 0.264521 | 0.432439794 |  | 6.116936 | 0.0686 | 545.4322 | 0.430931 | 0.692833984 |  | 220.6991968 | 8.28404E-08 | 5.87976E+11 | 0.626949 | 0.879305472 |  | 0.837622 | 0.440214 | 1.593794 | 0.590381 | 0.756788709 |
| metabolite540 | 48006.31907 | 133.0941 | 17315622.77 | 0.000497 | 0.004009458 |  | 1.29344 | 0.009653 | 173.3121 | 0.918174 | 0.972080981 |  | 33.89429293 | 1.86645E-09 | 6.15511E+11 | 0.77058 | 0.940047404 |  | 0.411822 | 0.208444 | 0.813634 | 0.012016 | 0.064907605 |
| metabolite541 | 162.5955904 | 0.634884 | 41641.2022 | 0.074668 | 0.180515446 |  | 3.258827 | 0.039037 | 272.0517 | 0.601797 | 0.80827686 |  | 11610374.15 | 0.00751292 | 1.79425E+16 | 0.134673 | 0.534467195 |  | 0.543643 | 0.291436 | 1.014111 | 0.057944 | 0.184531753 |
| metabolite542 | 6.352105195 | 0.035724 | 1129.484139 | 0.485739 | 0.644698499 |  | 34.74805 | 0.61461 | 1964.543 | 0.087574 | 0.32245972 |  | 0.412757888 | 1.11973E-09 | 152152458.1 | 0.930093 | 0.977587293 |  | 0.911424 | 0.507969 | 1.635323 | 0.756413 | 0.86717613 |
| metabolite543 | 2561.588961 | 16.35873 | 401115.4437 | 0.002916 | 0.015218843 |  | 0.269581 | 0.004314 | 16.84651 | 0.535638 | 0.767471559 |  | 90999.67631 | 0.000213222 | 3.88371E+13 | 0.262491 | 0.648628965 |  | 0.452499 | 0.254913 | 0.803237 | 0.007833 | 0.049021219 |
| metabolite544 | 6973417.69 | 20431.83 | 2380039501 | 6.07E-07 | 3.09E-05 |  | 0.767195 | 0.004493 | 130.9993 | 0.919692 | 0.972357747 |  | 4.80265472 | 8.15112E-11 | 2.82973E+11 | 0.901526 | 0.969275008 |  | 0.269359 | 0.134613 | 0.538984 | 0.00033 | 0.005796663 |
| metabolite545 | 1301.616601 | 2.028557 | 835177.9215 | 0.031792 | 0.096311585 |  | 0.984887 | 0.005448 | 178.0625 | 0.995428 | 0.998927461 |  | 3312.745245 | 4.46287E-08 | 2.45902E+14 | 0.526929 | 0.824919067 |  | 0.526618 | 0.252814 | 1.096958 | 0.089536 | 0.247040069 |
| metabolite546 | 396.4333794 | 0.958962 | 163884.9328 | 0.054138 | 0.14248801 |  | 8.672967 | 0.07085 | 1061.69 | 0.380368 | 0.652587742 |  | 13581.56833 | 1.13218E-06 | 1.62924E+14 | 0.423285 | 0.77189627 |  | 0.701714 | 0.353049 | 1.394714 | 0.314344 | 0.527241165 |
| metabolite547 | 20800082.5 | 46846.39 | 9235363273 | 3.54E-07 | 2.10E-05 |  | 0.010273 | 4.98E-05 | 2.120813 | 0.09508 | 0.333421024 |  | 4.00026E-06 | 2.17164E-17 | 736865.4875 | 0.349688 | 0.717869012 |  | 0.210399 | 0.102865 | 0.430345 | 4.15E-05 | 0.001854351 |
| metabolite548 | 0.448382801 | 0.001352 | 148.7450684 | 0.787006 | 0.876086631 |  | 177.839 | 2.037055 | 15525.7 | 0.025012 | 0.186447244 |  | 1960681.556 | 0.000608166 | 6.32109E+15 | 0.197294 | 0.596598127 |  | 1.295075 | 0.674572 | 2.486347 | 0.438812 | 0.637923084 |
| metabolite549 | 37.74391837 | 0.077138 | 18468.12546 | 0.25298 | 0.419299 |  | 25.66494 | 0.197278 | 3338.889 | 0.194079 | 0.471662387 |  | 2198.842275 | 1.20829E-07 | 4.00144E+13 | 0.524484 | 0.824725981 |  | 0.834598 | 0.414073 | 1.682199 | 0.61414 | 0.772874325 |
| metabolite550 | 0.006387192 | 8.86E-06 | 4.605538121 | 0.135133 | 0.276297087 |  | 3.512304 | 0.018815 | 655.6505 | 0.638665 | 0.831317121 |  | 9520155.09 | 0.00012232 | 7.4095E+17 | 0.211791 | 0.603798949 |  | 1.601167 | 0.761163 | 3.368179 | 0.217332 | 0.414397131 |
| metabolite551 | 32.26056081 | 0.05262 | 19778.39014 | 0.291084 | 0.462217806 |  | 26.13127 | 0.168597 | 4050.143 | 0.207399 | 0.485794701 |  | 2987296.488 | 7.93902E-05 | 1.12406E+17 | 0.23266 | 0.625185612 |  | 0.776616 | 0.376092 | 1.603682 | 0.495808 | 0.68424797 |
| metabolite552 | 0.01094714 | 3.68E-05 | 3.25689214 | 0.123113 | 0.258705245 |  | 534.1883 | 6.689575 | 42656.99 | 0.005849 | 0.117975509 |  | 7197600354 | 3.442746152 | 1.50477E+19 | 0.040496 | 0.358402676 |  | 2.082746 | 1.104884 | 3.92605 | 0.025241 | 0.10766476 |
| metabolite553 | 0.072912129 | 0.000261 | 20.40116208 | 0.364307 | 0.535634488 |  | 57.74403 | 0.716998 | 4650.463 | 0.072779 | 0.297124077 |  | 68328.45875 | 3.52588E-05 | 1.32415E+14 | 0.309807 | 0.693841165 |  | 1.311896 | 0.695229 | 2.475543 | 0.403859 | 0.613476301 |
| metabolite554 | 395.8056181 | 0.958961 | 163366.4036 | 0.054139 | 0.14248801 |  | 6.778513 | 0.055244 | 831.7269 | 0.437129 | 0.695686251 |  | 109.0674533 | 8.69256E-09 | 1.36849E+12 | 0.69324 | 0.907533437 |  | 0.650201 | 0.327685 | 1.290143 | 0.220808 | 0.418284658 |
| metabolite555 | 0.989939399 | 0.002793 | 350.9225904 | 0.997313 | 0.998718172 |  | 4.876332 | 0.048362 | 491.6809 | 0.502268 | 0.742300501 |  | 7360.268389 | 1.61133E-06 | 3.36205E+13 | 0.434353 | 0.779380463 |  | 1.076637 | 0.555832 | 2.085425 | 0.827119 | 0.914067653 |
| metabolite556 | 0.080131411 | 0.000231 | 27.79748352 | 0.399475 | 0.568424042 |  | 9.050096 | 0.090751 | 902.5149 | 0.350239 | 0.624460523 |  | 29550500.02 | 0.007745264 | 1.12744E+17 | 0.129312 | 0.530263915 |  | 1.44455 | 0.748559 | 2.787658 | 0.275206 | 0.485506966 |
| metabolite557 | 48.709295 | 0.210109 | 11292.23388 | 0.164747 | 0.315433364 |  | 465.1895 | 7.171002 | 30177.27 | 0.004697 | 0.109577358 |  | 65527.20624 | 6.2552E-05 | 6.86439E+13 | 0.297575 | 0.681279837 |  | 0.935296 | 0.503795 | 1.736378 | 0.832565 | 0.91708144 |
| metabolite558 | 18031.4305 | 41.30254 | 7871973.635 | 0.002035 | 0.011857403 |  | 0.798831 | 0.005397 | 118.2386 | 0.929962 | 0.974037858 |  | 4.553149478 | 1.54015E-10 | 1.34605E+11 | 0.902149 | 0.969275008 |  | 0.519391 | 0.25679 | 1.050535 | 0.071022 | 0.211357129 |
| metabolite559 | 1.935118644 | 0.003326 | 1125.715125 | 0.839307 | 0.910721567 |  | 43.75159 | 0.30551 | 6265.583 | 0.138579 | 0.402103043 |  | 218823.9295 | 7.61166E-06 | 6.29086E+15 | 0.319118 | 0.700344699 |  | 1.105198 | 0.539596 | 2.263662 | 0.785019 | 0.887091134 |
| metabolite560 | 188458.3019 | 1011.45 | 35114463.41 | 1.36E-05 | 0.000264686 |  | 0.585898 | 0.006618 | 51.87229 | 0.815637 | 0.926554541 |  | 3355.983681 | 1.41959E-06 | 7.93374E+12 | 0.462534 | 0.792880411 |  | 0.418844 | 0.225153 | 0.779159 | 0.007 | 0.04589891 |
| metabolite561 | 181.9859715 | 0.758943 | 43638.21061 | 0.065342 | 0.16306866 |  | 0.03316 | 0.000434 | 2.532877 | 0.126435 | 0.381464141 |  | 3.953219889 | 2.60705E-09 | 5994505671 | 0.898821 | 0.968957237 |  | 0.409988 | 0.224007 | 0.750381 | 0.004618 | 0.034833562 |
| metabolite562 | 3.559825991 | 0.010379 | 1220.927959 | 0.67071 | 0.797705167 |  | 69.57608 | 0.748281 | 6469.272 | 0.069243 | 0.290456173 |  | 17480490346 | 6.207236175 | 4.92276E+19 | 0.035851 | 0.342359087 |  | 1.186389 | 0.61483 | 2.28928 | 0.611322 | 0.772163563 |
| metabolite563 | 577.4994753 | 2.109864 | 158069.7363 | 0.028398 | 0.088674013 |  | 1.49773 | 0.016379 | 136.9542 | 0.861139 | 0.947996663 |  | 12.82769717 | 4.43746E-09 | 37081950370 | 0.818848 | 0.947573924 |  | 0.648438 | 0.341578 | 1.230967 | 0.188026 | 0.383177109 |
| metabolite564 | 0.104830934 | 0.000212 | 51.92510314 | 0.477711 | 0.639302835 |  | 59.06795 | 0.467853 | 7457.513 | 0.101303 | 0.343190353 |  | 467211.2486 | 2.8977E-05 | 7.53309E+15 | 0.278671 | 0.662490254 |  | 1.884618 | 0.94475 | 3.759495 | 0.074786 | 0.218782437 |
| metabolite565 | 327.5013638 | 1.350855 | 79399.4761 | 0.041026 | 0.117114314 |  | 0.425536 | 0.005207 | 34.77347 | 0.704437 | 0.870776373 |  | 0.063384811 | 3.73516E-11 | 107562465.1 | 0.799651 | 0.9460629 |  | 0.504287 | 0.27202 | 0.934878 | 0.031843 | 0.127108339 |
| metabolite566 | 1596.165222 | 7.540892 | 337857.025 | 0.008032 | 0.033369328 |  | 11.59915 | 0.152876 | 880.0607 | 0.26954 | 0.556559039 |  | 82878320952 | 106.2749282 | 6.46325E+19 | 0.017749 | 0.269946994 |  | 0.59545 | 0.321873 | 1.101553 | 0.101396 | 0.26461242 |
| metabolite567 | 234.317525 | 0.842458 | 65172.06618 | 0.059988 | 0.153846843 |  | 0.145825 | 0.001638 | 12.98415 | 0.402359 | 0.670616907 |  | 0.000193212 | 7.50395E-14 | 497481.7317 | 0.44086 | 0.782677331 |  | 0.534149 | 0.283481 | 1.006468 | 0.05491 | 0.179218842 |
| metabolite568 | 23.49040771 | 0.067108 | 8222.513578 | 0.293197 | 0.464781165 |  | 0.132506 | 0.001305 | 13.45852 | 0.393121 | 0.664744335 |  | 2.54182E-05 | 5.35522E-15 | 120645.6971 | 0.354022 | 0.720810472 |  | 0.690726 | 0.357155 | 1.335843 | 0.273915 | 0.48358673 |
| metabolite569 | 147.6323532 | 0.651781 | 33439.64428 | 0.07374 | 0.178979956 |  | 1.676704 | 0.022049 | 127.5029 | 0.815512 | 0.926554541 |  | 260.0699488 | 2.23041E-07 | 3.03246E+11 | 0.602653 | 0.872650335 |  | 0.529184 | 0.288019 | 0.972281 | 0.042664 | 0.150785972 |
| metabolite570 | 0.017029285 | 6.28E-05 | 4.617151331 | 0.157013 | 0.305866474 |  | 228.9497 | 2.997772 | 17485.64 | 0.015587 | 0.154964502 |  | 113562580 | 0.070485757 | 1.82965E+17 | 0.089175 | 0.478913031 |  | 1.845536 | 0.986328 | 3.453213 | 0.057817 | 0.184487114 |
| metabolite571 | 0.022092003 | 7.69E-05 | 6.343922145 | 0.189473 | 0.34501122 |  | 60.88192 | 0.728057 | 5091.096 | 0.071536 | 0.294639472 |  | 3286484.371 | 0.001524724 | 7.08389E+15 | 0.173924 | 0.572700186 |  | 1.577827 | 0.834554 | 2.983078 | 0.16328 | 0.352285021 |
| metabolite572 | 0.84920145 | 0.001952 | 369.4209655 | 0.958039 | 0.980990323 |  | 34.52378 | 0.302138 | 3944.859 | 0.145765 | 0.411005424 |  | 5739.618749 | 5.74035E-07 | 5.73889E+13 | 0.462832 | 0.792880411 |  | 1.247068 | 0.629826 | 2.469221 | 0.527701 | 0.706966915 |
| metabolite573 | 9.29491357 | 0.036399 | 2373.568927 | 0.432153 | 0.60019495 |  | 70.43346 | 0.951293 | 5214.872 | 0.055254 | 0.259769151 |  | 396033224.4 | 0.369355198 | 4.24638E+17 | 0.064665 | 0.426996029 |  | 1.292952 | 0.692589 | 2.413733 | 0.421564 | 0.625020256 |
| metabolite574 | 18.81873248 | 0.083624 | 4234.985255 | 0.290522 | 0.462181116 |  | 51.20071 | 0.749838 | 3496.107 | 0.070478 | 0.292171646 |  | 116.1658337 | 1.23392E-07 | 1.09363E+11 | 0.652839 | 0.890878737 |  | 0.955158 | 0.517368 | 1.763401 | 0.883665 | 0.942861226 |
| metabolite575 | 2387.199952 | 8.471167 | 672720.006 | 0.007966 | 0.033180055 |  | 0.806309 | 0.008221 | 79.08194 | 0.926851 | 0.974037858 |  | 16694.59726 | 4.42168E-06 | 6.30325E+13 | 0.389352 | 0.748257989 |  | 0.426179 | 0.225505 | 0.80543 | 0.009851 | 0.056302052 |
| metabolite576 | 124.164057 | 0.40981 | 37619.13993 | 0.100955 | 0.225454887 |  | 5.265606 | 0.055988 | 495.2231 | 0.475146 | 0.721241882 |  | 136677221.8 | 0.051716192 | 3.61215E+17 | 0.093374 | 0.486671909 |  | 0.645364 | 0.33814 | 1.231724 | 0.186901 | 0.382031657 |
| metabolite577 | 0.028434066 | 0.000104 | 7.800340667 | 0.216532 | 0.379212305 |  | 86.71345 | 1.093151 | 6878.484 | 0.047952 | 0.246134378 |  | 502116.3512 | 0.000271366 | 9.29082E+14 | 0.230495 | 0.623899558 |  | 1.568578 | 0.834366 | 2.948872 | 0.164976 | 0.354913566 |
| metabolite578 | 0.196542786 | 0.000533 | 72.50652375 | 0.59063 | 0.732180031 |  | 48.43766 | 0.484899 | 4838.551 | 0.101388 | 0.343190353 |  | 559307.0075 | 0.000109987 | 2.8442E+15 | 0.248284 | 0.640761773 |  | 1.444899 | 0.744478 | 2.804291 | 0.279024 | 0.490505354 |
| metabolite579 | 1281.213122 | 3.966611 | 413831.1041 | 0.016814 | 0.059609832 |  | 0.817069 | 0.007668 | 87.06051 | 0.932559 | 0.974816822 |  | 266.863192 | 4.50797E-08 | 1.57978E+12 | 0.627478 | 0.879305472 |  | 0.848755 | 0.435556 | 1.653945 | 0.63092 | 0.784633083 |
| metabolite580 | 16.38855138 | 0.058994 | 4552.729961 | 0.332113 | 0.505399449 |  | 31.09185 | 0.380352 | 2541.599 | 0.128923 | 0.3859835 |  | 1143298543 | 0.772452296 | 1.69218E+18 | 0.055406 | 0.402327874 |  | 1.056542 | 0.559096 | 1.996581 | 0.865803 | 0.936459959 |
| metabolite581 | 4.759942164 | 0.01976 | 1146.594925 | 0.578239 | 0.722750681 |  | 0.467102 | 0.006198 | 35.20268 | 0.730615 | 0.883843941 |  | 1359.232487 | 1.23146E-06 | 1.50026E+12 | 0.498471 | 0.811683517 |  | 0.650171 | 0.352068 | 1.200685 | 0.171711 | 0.363501683 |
| metabolite582 | 0.041202326 | 0.000183 | 9.288364263 | 0.251088 | 0.417841793 |  | 10.356 | 0.144822 | 740.5441 | 0.285586 | 0.572365249 |  | 0.054111519 | 5.53788E-11 | 52873211.15 | 0.782931 | 0.941497897 |  | 1.659362 | 0.90463 | 3.043767 | 0.104634 | 0.270486405 |
| metabolite583 | 0.136366302 | 0.000425 | 43.71383062 | 0.499948 | 0.656977941 |  | 29.76624 | 0.327707 | 2703.727 | 0.14303 | 0.407407962 |  | 1056.678589 | 3.16557E-07 | 3.52723E+12 | 0.534989 | 0.829425866 |  | 1.318683 | 0.688933 | 2.524083 | 0.40544 | 0.614831664 |
| metabolite584 | 0.000706811 | 3.76E-06 | 0.132761765 | 0.007668 | 0.032260913 |  | 8.312699 | 0.119859 | 576.5184 | 0.329633 | 0.606177589 |  | 11.82291596 | 1.42572E-08 | 9804240977 | 0.814065 | 0.946906762 |  | 1.621428 | 0.88778 | 2.96135 | 0.11864 | 0.292540625 |
| metabolite585 | 9.286258136 | 0.035757 | 2411.708147 | 0.43374 | 0.601497505 |  | 100.673 | 1.358698 | 7459.38 | 0.038035 | 0.221931548 |  | 3845116.73 | 0.002939405 | 5.0299E+15 | 0.159664 | 0.563224478 |  | 1.006628 | 0.537213 | 1.886215 | 0.983586 | 0.991369918 |
| metabolite586 | 0.007985221 | 3.06E-05 | 2.083320503 | 0.091657 | 0.210562153 |  | 312.6795 | 4.21048 | 23220.27 | 0.010187 | 0.133418413 |  | 2.06461E+13 | 22448.48106 | 1.89884E+22 | 0.004349 | 0.142834324 |  | 2.064669 | 1.110264 | 3.839502 | 0.023883 | 0.103541421 |
| metabolite587 | 4.81805955 | 0.015899 | 1460.050339 | 0.590716 | 0.732180031 |  | 57.23845 | 0.67391 | 4861.542 | 0.076853 | 0.30475391 |  | 169849.8498 | 6.92289E-05 | 4.16719E+14 | 0.277324 | 0.660932141 |  | 1.303246 | 0.685431 | 2.47793 | 0.420882 | 0.625020256 |
| metabolite588 | 2.276791616 | 0.006688 | 775.081449 | 0.782603 | 0.873455477 |  | 11.00062 | 0.113849 | 1062.931 | 0.306065 | 0.586221996 |  | 10931194.02 | 0.003200256 | 3.7338E+16 | 0.150691 | 0.554224569 |  | 1.049261 | 0.544007 | 2.023776 | 0.886172 | 0.944724491 |
| metabolite589 | 0.035545247 | 0.000127 | 9.960924038 | 0.248315 | 0.414714631 |  | 57.83958 | 0.709896 | 4712.547 | 0.073411 | 0.297124077 |  | 4201700.02 | 0.00223906 | 7.88469E+15 | 0.164328 | 0.568636202 |  | 1.473723 | 0.781331 | 2.779692 | 0.23354 | 0.435291859 |
| metabolite590 | 0.368991183 | 0.000703 | 193.7061561 | 0.755634 | 0.857121608 |  | 2.284268 | 0.016478 | 316.6654 | 0.743313 | 0.89169117 |  | 4.820782305 | 2.21335E-10 | 1.04999E+11 | 0.897186 | 0.96870562 |  | 0.987237 | 0.487409 | 1.999629 | 0.97161 | 0.984805499 |
| metabolite591 | 0.532831778 | 0.001919 | 147.9102795 | 0.826805 | 0.9020159 |  | 70.27116 | 0.897891 | 5499.597 | 0.058508 | 0.264422304 |  | 83125836.38 | 0.056540604 | 1.22211E+17 | 0.093212 | 0.486671909 |  | 1.396566 | 0.743194 | 2.624343 | 0.30161 | 0.515582402 |
| metabolite592 | 339.9006299 | 0.857432 | 134742.4397 | 0.058765 | 0.151232578 |  | 0.255251 | 0.002141 | 30.42832 | 0.576735 | 0.791517982 |  | 3.079425878 | 2.86511E-10 | 33097761807 | 0.924137 | 0.976740842 |  | 0.518639 | 0.264368 | 1.017469 | 0.058762 | 0.185905838 |
| metabolite593 | 20.78509668 | 0.080751 | 5350.059875 | 0.286301 | 0.458352703 |  | 2.028471 | 0.02512 | 163.8047 | 0.752839 | 0.89553175 |  | 411.3610865 | 2.63911E-07 | 6.41193E+11 | 0.578389 | 0.85722005 |  | 0.719823 | 0.385115 | 1.345431 | 0.305159 | 0.51867642 |
| metabolite594 | 3372.599759 | 14.14511 | 804124.5704 | 0.004386 | 0.020786941 |  | 0.130297 | 0.001513 | 11.22055 | 0.371945 | 0.645910279 |  | 79.70985235 | 3.4534E-08 | 1.83983E+11 | 0.691364 | 0.907533437 |  | 0.522355 | 0.278716 | 0.978969 | 0.045129 | 0.156665374 |
| metabolite595 | 320.9508423 | 0.598836 | 172016.2009 | 0.074566 | 0.180505576 |  | 2.072967 | 0.013715 | 313.3154 | 0.776387 | 0.909748377 |  | 10124.71046 | 3.25865E-07 | 3.14577E+14 | 0.455912 | 0.791392334 |  | 0.510868 | 0.251885 | 1.036133 | 0.065303 | 0.199619432 |
| metabolite596 | 0.055919262 | 0.000207 | 15.08735459 | 0.314806 | 0.488032073 |  | 38.03693 | 0.478314 | 3024.811 | 0.106003 | 0.353481876 |  | 318813.7903 | 0.000191345 | 5.31199E+14 | 0.244615 | 0.637028366 |  | 1.457471 | 0.776605 | 2.735266 | 0.243369 | 0.447542434 |
| metabolite597 | 76.96638677 | 0.408727 | 14493.35824 | 0.106953 | 0.23359118 |  | 0.299094 | 0.004633 | 19.3066 | 0.571408 | 0.790542575 |  | 210.4905003 | 3.88158E-07 | 1.14145E+11 | 0.603166 | 0.872650335 |  | 0.701664 | 0.387652 | 1.270037 | 0.244363 | 0.448039436 |
| metabolite598 | 2.42152329 | 0.006774 | 865.6265063 | 0.768662 | 0.865969677 |  | 41.98358 | 0.431068 | 4088.963 | 0.112488 | 0.360174443 |  | 1037691.219 | 0.00023809 | 4.52268E+15 | 0.223819 | 0.616622693 |  | 1.111592 | 0.573274 | 2.155401 | 0.754765 | 0.866356782 |
| metabolite599 | 0.028323756 | 0.000115 | 7.003763837 | 0.207565 | 0.368376643 |  | 160.9001 | 2.256228 | 11474.4 | 0.021409 | 0.174688709 |  | 2.74032E+14 | 481709.0858 | 1.5589E+23 | 0.001617 | 0.078975047 |  | 1.323889 | 0.710047 | 2.468402 | 0.379302 | 0.593168909 |
| metabolite600 | 0.229322458 | 0.000764 | 68.83659419 | 0.613867 | 0.751339726 |  | 97.19613 | 1.174699 | 8042.133 | 0.044596 | 0.237074035 |  | 1.19014E+12 | 840.2593262 | 1.68572E+21 | 0.010994 | 0.213598176 |  | 1.029618 | 0.541144 | 1.959021 | 0.929294 | 0.966583064 |
| metabolite601 | 0.283644627 | 0.000584 | 137.701019 | 0.690446 | 0.811323344 |  | 0.07731 | 0.000605 | 9.881784 | 0.3032 | 0.583677462 |  | 135.1482203 | 8.43144E-09 | 2.1663E+12 | 0.683143 | 0.903557902 |  | 1.29801 | 0.647479 | 2.602139 | 0.463856 | 0.659108358 |
| metabolite602 | 0.002150665 | 1.02E-05 | 0.454567955 | 0.026509 | 0.084516043 |  | 385.5275 | 5.982189 | 24845.67 | 0.006001 | 0.118151077 |  | 207994774.2 | 0.262664054 | 1.64704E+17 | 0.069615 | 0.444648008 |  | 2.00539 | 1.097431 | 3.66455 | 0.025628 | 0.108319681 |
| metabolite603 | 0.1500757 | 0.000526 | 42.84714612 | 0.512255 | 0.667061668 |  | 1561.099 | 22.35326 | 109023.5 | 0.000955 | 0.07423496 |  | 6962345297 | 4.74616895 | 1.02133E+19 | 0.037581 | 0.346926806 |  | 1.602184 | 0.851537 | 3.014542 | 0.146662 | 0.330257184 |
| metabolite604 | 0.008385783 | 3.55E-05 | 1.979178972 | 0.089116 | 0.206434258 |  | 0.061641 | 0.000813 | 4.673003 | 0.209662 | 0.488116163 |  | 3077553.798 | 0.002718612 | 3.48389E+15 | 0.162938 | 0.566515099 |  | 1.639195 | 0.884675 | 3.037228 | 0.119126 | 0.293076362 |
| metabolite605 | 15544386.88 | 117687.3 | 2053136092 | 1.17E-09 | 3.61E-07 |  | 0.003894 | 4.65E-05 | 0.326144 | 0.015602 | 0.154964502 |  | 1.90113E-05 | 6.20596E-15 | 58239.35645 | 0.331468 | 0.705723693 |  | 0.204041 | 0.114326 | 0.364159 | 4.22E-07 | 8.65E-05 |
| metabolite606 | 322.7838779 | 1.413493 | 73710.58611 | 0.039372 | 0.113352167 |  | 38.13621 | 0.51408 | 2829.072 | 0.100308 | 0.343190353 |  | 256000339.5 | 0.256169431 | 2.55831E+17 | 0.069755 | 0.444776819 |  | 0.962698 | 0.516037 | 1.795971 | 0.905101 | 0.955842451 |
| metabolite607 | 0.003216752 | 1.31E-05 | 0.792330611 | 0.043436 | 0.12248517 |  | 240.4674 | 3.27083 | 17678.87 | 0.013864 | 0.149726748 |  | 116502154.7 | 0.085867004 | 1.58067E+17 | 0.086198 | 0.475306985 |  | 1.982121 | 1.067483 | 3.680435 | 0.032386 | 0.128357977 |
| metabolite608 | 2.942083341 | 0.012027 | 719.7223977 | 0.701287 | 0.820970791 |  | 194.3318 | 2.852694 | 13238.31 | 0.015985 | 0.155290634 |  | 15084174677 | 19.84584479 | 1.1465E+19 | 0.026658 | 0.301331155 |  | 1.100631 | 0.592287 | 2.045273 | 0.762238 | 0.870894265 |
| metabolite609 | 1.203579602 | 0.003482 | 416.0520029 | 0.95057 | 0.976911369 |  | 33.99981 | 0.357092 | 3237.227 | 0.132108 | 0.390685725 |  | 28247269.98 | 0.008053751 | 9.90729E+16 | 0.128859 | 0.530263915 |  | 1.123889 | 0.581994 | 2.170346 | 0.72861 | 0.851190927 |
| metabolite610 | 3.137291392 | 0.008472 | 1161.743194 | 0.70548 | 0.823799488 |  | 0.260338 | 0.002481 | 27.3226 | 0.571977 | 0.790542575 |  | 1934.233498 | 3.47453E-07 | 1.07677E+13 | 0.510001 | 0.818250856 |  | 0.724353 | 0.37292 | 1.40697 | 0.34316 | 0.555614442 |
| metabolite611 | 38.92832652 | 0.158325 | 9571.516906 | 0.195013 | 0.351818944 |  | 6.335608 | 0.081422 | 492.9851 | 0.407744 | 0.675507742 |  | 125303070.5 | 0.117682202 | 1.33417E+17 | 0.081464 | 0.465098286 |  | 0.736763 | 0.395465 | 1.37261 | 0.33798 | 0.550028652 |
| metabolite612 | 1.466379336 | 0.004729 | 454.7236882 | 0.896185 | 0.944276401 |  | 106.7491 | 1.26741 | 8991.06 | 0.041275 | 0.231728799 |  | 20120220.36 | 0.008613918 | 4.69964E+16 | 0.129353 | 0.530263915 |  | 1.323496 | 0.694982 | 2.520415 | 0.395594 | 0.607270747 |
| metabolite613 | 1.026336366 | 0.003226 | 326.567201 | 0.992961 | 0.996182633 |  | 0.272474 | 0.002934 | 25.30762 | 0.574977 | 0.791284873 |  | 0.065196045 | 2.03505E-11 | 208865549.7 | 0.807295 | 0.9460629 |  | 0.959436 | 0.501341 | 1.836109 | 0.900712 | 0.952834027 |
| metabolite614 | 0.781894901 | 0.001351 | 452.6135507 | 0.939707 | 0.971921334 |  | 33.86165 | 0.236028 | 4857.951 | 0.167261 | 0.437115775 |  | 14595.12071 | 4.97696E-07 | 4.28008E+14 | 0.437197 | 0.782651729 |  | 1.450778 | 0.711003 | 2.960264 | 0.308708 | 0.520970441 |
| metabolite615 | 0.597878649 | 0.002218 | 161.1734097 | 0.857379 | 0.921707218 |  | 1.349194 | 0.016441 | 110.7213 | 0.894285 | 0.959991298 |  | 0.040206526 | 2.35477E-11 | 68650720.22 | 0.767552 | 0.940047404 |  | 1.531506 | 0.819334 | 2.862703 | 0.184404 | 0.38014775 |
| metabolite616 | 1.457574139 | 0.004366 | 486.650759 | 0.899101 | 0.946124649 |  | 7.55047 | 0.078952 | 722.078 | 0.386811 | 0.658465144 |  | 359172.7668 | 0.000105401 | 1.22394E+15 | 0.25581 | 0.645470675 |  | 1.074525 | 0.558468 | 2.067448 | 0.829951 | 0.915768329 |
| metabolite617 | 0.385826244 | 0.001155 | 128.8563239 | 0.748646 | 0.852478187 |  | 124.0283 | 1.392604 | 11046.23 | 0.037584 | 0.220921127 |  | 342520.5307 | 9.9437E-05 | 1.17985E+15 | 0.257801 | 0.647052464 |  | 1.625365 | 0.849705 | 3.109092 | 0.144977 | 0.327654886 |
| metabolite618 | 0.389407483 | 0.001629 | 93.06324123 | 0.736344 | 0.844689876 |  | 92.51471 | 1.344179 | 6367.432 | 0.038264 | 0.222569499 |  | 910948.8357 | 0.000970888 | 8.5471E+14 | 0.195663 | 0.593709566 |  | 1.130596 | 0.610174 | 2.094891 | 0.69723 | 0.831848922 |
| metabolite619 | 0.002412842 | 1.13E-05 | 0.516668217 | 0.029793 | 0.092059481 |  | 222.7362 | 3.345488 | 14829.35 | 0.013025 | 0.146937782 |  | 40230161.25 | 0.046766145 | 3.46076E+16 | 0.09809 | 0.492678215 |  | 1.806019 | 0.983586 | 3.316137 | 0.059155 | 0.186496097 |
| metabolite620 | 0.010521137 | 3.51E-05 | 3.15094613 | 0.120313 | 0.254696718 |  | 147.5421 | 1.734702 | 12548.93 | 0.029662 | 0.200322487 |  | 60887.13559 | 2.06397E-05 | 1.79617E+14 | 0.324196 | 0.703974933 |  | 1.871985 | 0.988298 | 3.545822 | 0.05693 | 0.182827993 |
| metabolite621 | 2817.989976 | 3.398089 | 2336921.717 | 0.022353 | 0.074094381 |  | 0.571443 | 0.002536 | 128.7574 | 0.839933 | 0.938160513 |  | 2.981657598 | 1.32166E-11 | 6.7266E+11 | 0.934867 | 0.978919523 |  | 0.620246 | 0.287188 | 1.339561 | 0.226626 | 0.424538003 |
| metabolite622 | 0.004055852 | 2.08E-05 | 0.791091267 | 0.04301 | 0.121467573 |  | 211.9738 | 3.4745 | 12932.18 | 0.012012 | 0.14151295 |  | 114902624.9 | 0.211177802 | 6.25189E+16 | 0.07324 | 0.449246835 |  | 1.932998 | 1.0688 | 3.495957 | 0.031362 | 0.125958403 |
| metabolite623 | 0.034280342 | 0.000162 | 7.275658998 | 0.219808 | 0.383141446 |  | 210.8399 | 3.392753 | 13102.47 | 0.012474 | 0.143313692 |  | 4057228.465 | 0.006208965 | 2.65118E+15 | 0.144582 | 0.546460871 |  | 1.600436 | 0.877208 | 2.919941 | 0.128135 | 0.305518079 |
| metabolite624 | 0.054093468 | 0.000196 | 14.9135547 | 0.311149 | 0.484343428 |  | 130.157 | 1.67615 | 10107 | 0.030423 | 0.202218637 |  | 645270800.2 | 0.436724642 | 9.53403E+17 | 0.062305 | 0.420282514 |  | 1.707714 | 0.911278 | 3.200214 | 0.097724 | 0.258830584 |
| metabolite625 | 160.7237452 | 0.608061 | 42482.76119 | 0.076965 | 0.184150524 |  | 27.773 | 0.337187 | 2287.572 | 0.142516 | 0.407040102 |  | 203.3745655 | 9.657E-08 | 4.28303E+11 | 0.628451 | 0.879305472 |  | 0.984408 | 0.520547 | 1.861616 | 0.961531 | 0.981510624 |
| metabolite626 | 0.98964378 | 0.004427 | 221.2113928 | 0.996997 | 0.998718172 |  | 121.1379 | 1.880885 | 7801.86 | 0.025948 | 0.189924128 |  | 62253.67157 | 8.18953E-05 | 4.73229E+13 | 0.292324 | 0.675895889 |  | 1.204106 | 0.655333 | 2.212419 | 0.550782 | 0.722499747 |
| metabolite627 | 0.091828318 | 0.000206 | 40.84641594 | 0.444394 | 0.611385737 |  | 870.7282 | 8.353311 | 90762.53 | 0.005132 | 0.112026228 |  | 1363772.914 | 0.000129385 | 1.43748E+16 | 0.232823 | 0.625185612 |  | 1.58425 | 0.799948 | 3.137515 | 0.189631 | 0.384546167 |
| metabolite628 | 1.224035977 | 0.004792 | 312.6799066 | 0.943144 | 0.973441376 |  | 0.122874 | 0.00159 | 9.496071 | 0.346599 | 0.620752441 |  | 8.778396306 | 6.2957E-09 | 12240136624 | 0.840122 | 0.954234322 |  | 0.671473 | 0.361226 | 1.248182 | 0.21062 | 0.408151791 |
| metabolite629 | 0.007365788 | 2.16E-05 | 2.507969875 | 0.101586 | 0.226416753 |  | 99.719 | 1.035387 | 9604.021 | 0.05076 | 0.253485011 |  | 2556696995 | 0.674359373 | 9.6932E+18 | 0.05679 | 0.407210839 |  | 1.673905 | 0.867062 | 3.231552 | 0.127636 | 0.305190594 |
| metabolite630 | 0.101534545 | 0.000516 | 19.98658644 | 0.397872 | 0.567171872 |  | 261.6163 | 4.592079 | 14904.6 | 0.008041 | 0.126652263 |  | 38829.12105 | 7.70646E-05 | 1.95641E+13 | 0.303567 | 0.687752235 |  | 1.543644 | 0.854444 | 2.788756 | 0.153046 | 0.337265424 |
| metabolite631 | 0.001137138 | 5.99E-06 | 0.215768388 | 0.012706 | 0.047870245 |  | 149.8546 | 2.374401 | 9457.72 | 0.019568 | 0.165250229 |  | 12098379.2 | 0.019006488 | 7.7011E+15 | 0.11768 | 0.519645256 |  | 1.902311 | 1.048384 | 3.451779 | 0.03663 | 0.138667711 |
| metabolite632 | 1.192857647 | 0.004344 | 327.5822332 | 0.951029 | 0.977075415 |  | 162.8417 | 2.167509 | 12234.05 | 0.022677 | 0.178026796 |  | 135683.3599 | 8.25013E-05 | 2.23148E+14 | 0.277397 | 0.660932141 |  | 1.259274 | 0.66996 | 2.366964 | 0.475493 | 0.668489373 |
| metabolite633 | 28.15426357 | 0.025753 | 30779.45055 | 0.351834 | 0.523308614 |  | 0.834398 | 0.003305 | 210.6485 | 0.948963 | 0.977627498 |  | 92.41979377 | 2.40692E-10 | 3.54869E+13 | 0.740066 | 0.929662886 |  | 1.019632 | 0.46219 | 2.249398 | 0.961674 | 0.981510624 |
| metabolite634 | 0.506548269 | 0.002097 | 122.3462914 | 0.808492 | 0.889803214 |  | 41.50873 | 0.582743 | 2956.663 | 0.08971 | 0.325746027 |  | 403.2185748 | 3.65366E-07 | 4.44993E+11 | 0.573389 | 0.854899521 |  | 1.28437 | 0.693368 | 2.379123 | 0.427899 | 0.630382477 |
| metabolite635 | 25.69744049 | 0.188205 | 3508.723006 | 0.198294 | 0.355663218 |  | 13.72893 | 0.286332 | 658.2698 | 0.187352 | 0.462941033 |  | 18.16583626 | 1.22844E-07 | 2686305872 | 0.76314 | 0.938107312 |  | 0.703314 | 0.404111 | 1.224049 | 0.215786 | 0.412136756 |
| metabolite636 | 0.009576384 | 2.81E-05 | 3.265219626 | 0.121065 | 0.255703772 |  | 159.3235 | 1.69108 | 15010.52 | 0.030876 | 0.203594474 |  | 48956549.81 | 0.011697115 | 2.049E+17 | 0.120088 | 0.522446889 |  | 1.575331 | 0.815295 | 3.04389 | 0.179013 | 0.372583873 |
| metabolite637 | 0.038407076 | 0.000214 | 6.890458292 | 0.220912 | 0.383929199 |  | 29.09476 | 0.498699 | 1697.427 | 0.107075 | 0.353835193 |  | 5376.547644 | 1.37228E-05 | 2.10651E+12 | 0.39666 | 0.756425822 |  | 1.465935 | 0.817317 | 2.629294 | 0.202087 | 0.399756766 |
| metabolite638 | 0.261644602 | 0.000687 | 99.63105922 | 0.659175 | 0.789096265 |  | 93.98626 | 0.938687 | 9410.393 | 0.055774 | 0.260348525 |  | 1307817599 | 0.282515886 | 6.05413E+18 | 0.067165 | 0.436515549 |  | 1.318377 | 0.676011 | 2.571138 | 0.419066 | 0.624560084 |
| metabolite639 | 0.210562674 | 0.000863 | 51.39337983 | 0.579699 | 0.72359954 |  | 79.91172 | 1.131158 | 5645.44 | 0.046137 | 0.241700557 |  | 2688750.195 | 0.002662904 | 2.71485E+15 | 0.164433 | 0.568636202 |  | 1.448234 | 0.781991 | 2.682105 | 0.241364 | 0.445396403 |
| metabolite640 | 0.036397523 | 0.000127 | 10.43351587 | 0.253562 | 0.419796913 |  | 216.9794 | 2.748422 | 17129.84 | 0.017433 | 0.161329273 |  | 131891629.4 | 0.071002768 | 2.44996E+17 | 0.088751 | 0.47802064 |  | 1.388476 | 0.733448 | 2.628495 | 0.315666 | 0.527891308 |
| metabolite641 | 2.587446609 | 0.010372 | 645.4585366 | 0.736302 | 0.844689876 |  | 36.90925 | 0.50269 | 2710.008 | 0.102546 | 0.345324977 |  | 0.318961012 | 2.47088E-10 | 411740247.7 | 0.915172 | 0.975040351 |  | 1.17197 | 0.629629 | 2.181466 | 0.617648 | 0.774675165 |
| metabolite642 | 0.095121158 | 0.00035 | 25.83819947 | 0.412411 | 0.580432576 |  | 98.88856 | 1.284839 | 7611.029 | 0.040478 | 0.228287106 |  | 8533787.255 | 0.005569896 | 1.30748E+16 | 0.141973 | 0.542758378 |  | 1.320466 | 0.702501 | 2.482032 | 0.38981 | 0.604322801 |
| metabolite643 | 0.170979034 | 0.000808 | 36.1997856 | 0.519338 | 0.672481345 |  | 46.48507 | 0.722543 | 2990.635 | 0.073465 | 0.297124077 |  | 0.398155953 | 5.59374E-10 | 283402951.4 | 0.929597 | 0.977539147 |  | 1.434928 | 0.787028 | 2.616194 | 0.241138 | 0.445204953 |
| metabolite644 | 0.005466009 | 3.2E-05 | 0.933408238 | 0.049467 | 0.134044972 |  | 114.873 | 2.055397 | 6420.083 | 0.022681 | 0.178026796 |  | 9223664.27 | 0.027037171 | 3.14663E+15 | 0.11248 | 0.513062936 |  | 1.576442 | 0.879927 | 2.82429 | 0.128857 | 0.305745899 |
| metabolite645 | 17.37564079 | 0.078014 | 3869.972346 | 0.30285 | 0.474409897 |  | 15.82477 | 0.226539 | 1105.431 | 0.205095 | 0.483637188 |  | 18.27452461 | 2.00598E-08 | 16648122953 | 0.783028 | 0.941497897 |  | 0.911086 | 0.494242 | 1.679494 | 0.765947 | 0.873631653 |
| metabolite646 | 676.4575108 | 2.036905 | 224651.9928 | 0.029865 | 0.092204728 |  | 1.05135 | 0.009853 | 112.1858 | 0.98327 | 0.992676704 |  | 0.04690158 | 7.74196E-12 | 284134498.5 | 0.790546 | 0.944051932 |  | 0.586099 | 0.302745 | 1.134656 | 0.115771 | 0.287697033 |
| metabolite647 | 268.19842 | 0.538874 | 133482.8156 | 0.080338 | 0.190500231 |  | 7.590852 | 0.054103 | 1065.032 | 0.42335 | 0.687249806 |  | 700211.1373 | 3.25984E-05 | 1.50405E+16 | 0.269893 | 0.655253129 |  | 0.742818 | 0.36627 | 1.506481 | 0.411641 | 0.618642876 |
| metabolite648 | 0.005677919 | 2.71E-05 | 1.187902714 | 0.060448 | 0.154320867 |  | 129.8417 | 1.989749 | 8472.867 | 0.024351 | 0.185877348 |  | 1206.79967 | 1.38627E-06 | 1.05056E+12 | 0.50068 | 0.812201491 |  | 1.52809 | 0.833247 | 2.802364 | 0.173324 | 0.36566613 |
| metabolite649 | 0.046791458 | 0.000169 | 12.9548743 | 0.288181 | 0.460370413 |  | 210.8574 | 2.753999 | 16144.11 | 0.017247 | 0.161214888 |  | 2178254515 | 1.496869592 | 3.16981E+18 | 0.04822 | 0.384827718 |  | 1.393033 | 0.739191 | 2.625222 | 0.307456 | 0.520515511 |
| metabolite650 | 0.005117969 | 1.53E-05 | 1.707507379 | 0.077893 | 0.185653313 |  | 1.95773 | 0.018941 | 202.3485 | 0.77703 | 0.909927034 |  | 65826.90103 | 1.3729E-05 | 3.15623E+14 | 0.331409 | 0.705723693 |  | 1.468854 | 0.759296 | 2.84149 | 0.255886 | 0.462088404 |
| metabolite651 | 0.406696811 | 0.001957 | 84.52391494 | 0.741702 | 0.84885233 |  | 20.88291 | 0.323896 | 1346.409 | 0.155626 | 0.421465998 |  | 3495.349154 | 5.73339E-06 | 2.13093E+12 | 0.430879 | 0.777110602 |  | 1.089041 | 0.596953 | 1.986774 | 0.781472 | 0.884857504 |
| metabolite652 | 262.1449325 | 0.671131 | 102394.2499 | 0.070081 | 0.171659066 |  | 0.895521 | 0.007598 | 105.5439 | 0.963912 | 0.982173547 |  | 1319714.887 | 0.000155508 | 1.11997E+16 | 0.229528 | 0.621736854 |  | 0.618833 | 0.314646 | 1.217096 | 0.167102 | 0.357827706 |
| metabolite653 | 0.123761021 | 0.0004 | 38.31531823 | 0.476698 | 0.638966051 |  | 205.971 | 2.486848 | 17059.36 | 0.019803 | 0.165250229 |  | 28409970.4 | 0.011766668 | 6.85943E+16 | 0.122323 | 0.526349291 |  | 1.331689 | 0.698475 | 2.538955 | 0.386158 | 0.600851948 |
| metabolite654 | 2.338769517 | 0.001893 | 2889.352896 | 0.815483 | 0.895294409 |  | 1706.017 | 7.449719 | 390685.2 | 0.00838 | 0.126652263 |  | 1.13924E+11 | 0.308137217 | 4.212E+22 | 0.063647 | 0.425574296 |  | 1.141925 | 0.512218 | 2.545776 | 0.746205 | 0.861215423 |
| metabolite655 | 0.020449737 | 0.000111 | 3.763376109 | 0.146593 | 0.291425245 |  | 23.09017 | 0.380904 | 1399.712 | 0.136686 | 0.399462449 |  | 1428.429363 | 3.07247E-06 | 6.64094E+11 | 0.47708 | 0.79982887 |  | 1.475606 | 0.819095 | 2.658315 | 0.197828 | 0.395043799 |
| metabolite656 | 0.055322372 | 0.000191 | 15.98643885 | 0.318886 | 0.492195492 |  | 10.80418 | 0.124909 | 934.5226 | 0.297895 | 0.579217354 |  | 1780.781523 | 7.56316E-07 | 4.19294E+12 | 0.498035 | 0.811328654 |  | 1.513354 | 0.800794 | 2.859961 | 0.204655 | 0.402051276 |
| metabolite657 | 0.009935541 | 1.06E-05 | 9.287753318 | 0.189086 | 0.344733488 |  | 60.02595 | 0.278008 | 12960.46 | 0.138222 | 0.401960631 |  | 17163247590 | 0.104783436 | 2.81129E+21 | 0.07638 | 0.457907084 |  | 1.643043 | 0.759977 | 3.552203 | 0.209495 | 0.40703399 |
| metabolite658 | 0.05428227 | 0.000159 | 18.48553773 | 0.329501 | 0.502786682 |  | 32.50213 | 0.338232 | 3123.267 | 0.137855 | 0.401462844 |  | 35889711.5 | 0.009908772 | 1.29993E+17 | 0.124205 | 0.527043366 |  | 1.310995 | 0.679178 | 2.53057 | 0.421382 | 0.625020256 |
| metabolite659 | 0.000501718 | 1.73E-06 | 0.145511318 | 0.009851 | 0.039096971 |  | 1355.707 | 16.61996 | 110586.4 | 0.001727 | 0.084346324 |  | 30972.20746 | 7.68516E-06 | 1.24822E+14 | 0.361444 | 0.728978194 |  | 3.341507 | 1.799769 | 6.203945 | 0.000219 | 0.004532241 |
| metabolite660 | 0.02765159 | 0.000132 | 5.809496924 | 0.191184 | 0.347303451 |  | 68.45833 | 1.057698 | 4430.888 | 0.049457 | 0.249369471 |  | 1828645.806 | 0.002798737 | 1.19481E+15 | 0.1666 | 0.568636202 |  | 1.459965 | 0.798903 | 2.668032 | 0.221244 | 0.418896349 |
| metabolite661 | 0.00429087 | 1.81E-05 | 1.017003 | 0.053222 | 0.141480879 |  | 186.813 | 2.612435 | 13358.83 | 0.018022 | 0.16351629 |  | 1565997413 | 1.511897645 | 1.62203E+18 | 0.048049 | 0.384827718 |  | 1.702656 | 0.917255 | 3.16056 | 0.094542 | 0.254052857 |
| metabolite662 | 12.89708643 | 0.077966 | 2133.441385 | 0.328698 | 0.502076647 |  | 3.756212 | 0.066594 | 211.8686 | 0.521397 | 0.756687166 |  | 566.1604748 | 2.0096E-06 | 1.59503E+11 | 0.524422 | 0.824725981 |  | 0.810134 | 0.455146 | 1.441992 | 0.475648 | 0.668489373 |
| metabolite663 | 0.083715805 | 0.000192 | 36.48367068 | 0.425449 | 0.593617798 |  | 1.418721 | 0.01169 | 172.1818 | 0.886666 | 0.958345213 |  | 28864.58222 | 2.74532E-06 | 3.03485E+14 | 0.38491 | 0.743571898 |  | 1.109923 | 0.558813 | 2.204547 | 0.766357 | 0.873631653 |
| metabolite664 | 0.01083773 | 4.79E-05 | 2.45342088 | 0.10476 | 0.23072065 |  | 192.852 | 2.867474 | 12970.26 | 0.015823 | 0.155217644 |  | 136166048.4 | 0.161349227 | 1.14913E+17 | 0.076822 | 0.45805738 |  | 1.593268 | 0.864001 | 2.938079 | 0.138585 | 0.318910437 |
| metabolite665 | 0.002086411 | 1.17E-05 | 0.373534876 | 0.021501 | 0.071841748 |  | 19.54685 | 0.309042 | 1236.333 | 0.16281 | 0.43086535 |  | 16.06002498 | 2.76754E-08 | 9319628644 | 0.787918 | 0.942605472 |  | 2.034298 | 1.134786 | 3.646829 | 0.018794 | 0.086777083 |
| metabolite666 | 0.03941063 | 0.00013 | 11.90792547 | 0.269476 | 0.438799558 |  | 101.6377 | 1.199579 | 8611.542 | 0.04369 | 0.236415195 |  | 81680817.62 | 0.035656859 | 1.8711E+17 | 0.100381 | 0.493288222 |  | 1.524486 | 0.802193 | 2.897131 | 0.200711 | 0.398412495 |
| metabolite667 | 0.977477861 | 0.002827 | 337.9387072 | 0.99392 | 0.996605199 |  | 2.758808 | 0.027752 | 274.2547 | 0.666245 | 0.848467311 |  | 50.33667267 | 1.15325E-08 | 2.19708E+11 | 0.729977 | 0.925034149 |  | 1.12764 | 0.583946 | 2.177553 | 0.721183 | 0.846684943 |
| metabolite668 | 0.004553735 | 1.41E-05 | 1.475472777 | 0.070218 | 0.171659066 |  | 1.663242 | 0.016402 | 168.6585 | 0.829475 | 0.932500054 |  | 12.22642032 | 2.563E-09 | 58324410795 | 0.826129 | 0.950624405 |  | 1.143919 | 0.590997 | 2.21414 | 0.690615 | 0.828068199 |
| metabolite669 | 0.186718154 | 0.000108 | 323.6449209 | 0.660042 | 0.78987981 |  | 181.5188 | 0.550898 | 59809.77 | 0.081429 | 0.311528818 |  | 231934200.9 | 0.000140648 | 3.82468E+20 | 0.182319 | 0.580004487 |  | 1.432738 | 0.619694 | 3.312505 | 0.402199 | 0.61293609 |
| metabolite670 | 0.034348797 | 0.000156 | 7.570515326 | 0.2233 | 0.386504132 |  | 138.0821 | 2.117034 | 9006.309 | 0.02263 | 0.178026796 |  | 87.81077398 | 9.73998E-08 | 79165826222 | 0.671377 | 0.902331541 |  | 1.464093 | 0.797339 | 2.688401 | 0.221459 | 0.419088887 |
| metabolite671 | 0.094236122 | 0.000399 | 22.27793404 | 0.398807 | 0.56769704 |  | 47.40521 | 0.671067 | 3348.777 | 0.078411 | 0.307920012 |  | 2730392343 | 3.639708732 | 2.04825E+18 | 0.039464 | 0.355749234 |  | 1.532031 | 0.830347 | 2.826673 | 0.174986 | 0.367707393 |
| metabolite672 | 1.056815572 | 0.003638 | 307.0255385 | 0.984798 | 0.992378901 |  | 156.8125 | 1.991802 | 12345.68 | 0.025181 | 0.186815458 |  | 1743314.045 | 0.000902752 | 3.36653E+15 | 0.190418 | 0.590344766 |  | 1.211902 | 0.640398 | 2.293429 | 0.556013 | 0.726886063 |
| metabolite673 | 0.076863375 | 0.000248 | 23.80035319 | 0.382489 | 0.551443738 |  | 254.4524 | 3.082895 | 21001.7 | 0.015435 | 0.154964502 |  | 13022354.97 | 0.005144823 | 3.29616E+16 | 0.140918 | 0.542758378 |  | 1.509948 | 0.793225 | 2.874269 | 0.21223 | 0.409987638 |
| metabolite674 | 3040.461285 | 17.47328 | 529059.4028 | 0.00289 | 0.015149964 |  | 21.24562 | 0.321606 | 1403.508 | 0.155695 | 0.421465998 |  | 884.9320621 | 1.26684E-06 | 6.18156E+11 | 0.515057 | 0.819395994 |  | 0.551594 | 0.304324 | 0.999778 | 0.052416 | 0.173209634 |
| metabolite675 | 0.209275537 | 0.000664 | 65.94777019 | 0.595182 | 0.735749192 |  | 169.88 | 2.015542 | 14318.34 | 0.025151 | 0.186815458 |  | 177141055.1 | 0.073898066 | 4.24625E+17 | 0.087567 | 0.475490674 |  | 1.167828 | 0.610758 | 2.233001 | 0.639902 | 0.791558374 |
| metabolite676 | 417.7841001 | 1.643254 | 106218.2691 | 0.034896 | 0.102939476 |  | 3.264159 | 0.038336 | 277.9293 | 0.602909 | 0.808892614 |  | 2.005381418 | 9.51633E-10 | 4225949176 | 0.949461 | 0.986298878 |  | 0.716703 | 0.380377 | 1.350405 | 0.304983 | 0.51867642 |
| metabolite677 | 180.4705689 | 0.777078 | 41912.9413 | 0.064223 | 0.160846134 |  | 0.039827 | 0.000532 | 2.980688 | 0.146033 | 0.411005424 |  | 0.012391082 | 9.35256E-12 | 16416780.16 | 0.682804 | 0.903557902 |  | 0.616456 | 0.332719 | 1.142161 | 0.127007 | 0.303882925 |
| metabolite678 | 0.353299419 | 0.001335 | 93.48219021 | 0.715377 | 0.830392301 |  | 31.36251 | 0.405523 | 2425.525 | 0.123233 | 0.378275375 |  | 1068400827 | 0.941381138 | 1.21256E+18 | 0.053178 | 0.394365301 |  | 0.997079 | 0.531723 | 1.869709 | 0.992741 | 0.994616935 |
| metabolite679 | 0.133540123 | 0.00038 | 46.9625705 | 0.502284 | 0.658648933 |  | 0.079545 | 0.000798 | 7.928833 | 0.283301 | 0.570754969 |  | 0.000513612 | 1.08879E-13 | 2422844.652 | 0.506498 | 0.816726692 |  | 0.99291 | 0.512305 | 1.924381 | 0.983223 | 0.991369918 |
| metabolite680 | 3.644442395 | 0.011378 | 1167.378171 | 0.661271 | 0.790839672 |  | 0.063898 | 0.000697 | 5.856916 | 0.235341 | 0.515872016 |  | 0.004198284 | 1.27602E-12 | 13812996.3 | 0.625446 | 0.879305472 |  | 0.874664 | 0.45664 | 1.675358 | 0.687105 | 0.825459971 |
| metabolite681 | 3.563465381 | 0.014245 | 891.4487404 | 0.652848 | 0.784051457 |  | 0.77026 | 0.009921 | 59.80286 | 0.906632 | 0.965785701 |  | 6305369.831 | 0.005863857 | 6.78013E+15 | 0.142867 | 0.542758378 |  | 0.833682 | 0.44773 | 1.55233 | 0.567457 | 0.738111681 |
| metabolite682 | 10227.05059 | 29.53772 | 3540983.106 | 0.002492 | 0.013792041 |  | 0.070428 | 0.000595 | 8.332992 | 0.27833 | 0.565343163 |  | 0.00406803 | 3.66424E-13 | 45163120.55 | 0.641813 | 0.88541499 |  | 0.406118 | 0.208717 | 0.790218 | 0.009141 | 0.054101563 |
| metabolite683 | 0.004108945 | 1.51E-05 | 1.114980498 | 0.057178 | 0.148755441 |  | 3.601982 | 0.040869 | 317.4616 | 0.576071 | 0.791336134 |  | 14.318274 | 5.75803E-09 | 35604736306 | 0.809901 | 0.9460629 |  | 1.255539 | 0.661912 | 2.381553 | 0.487439 | 0.676219156 |
| metabolite684 | 18.47556957 | 0.0483 | 7067.1641 | 0.338523 | 0.512542019 |  | 0.008359 | 8.26E-05 | 0.846043 | 0.044648 | 0.237074035 |  | 0.000301146 | 4.44449E-14 | 2040482.87 | 0.484132 | 0.802417608 |  | 0.708252 | 0.362579 | 1.383481 | 0.314793 | 0.527640047 |
| metabolite685 | 5.902503278 | 0.022883 | 1522.535868 | 0.532163 | 0.684526049 |  | 56.53956 | 0.755416 | 4231.736 | 0.069541 | 0.290944915 |  | 11694243.44 | 0.00965067 | 1.41706E+16 | 0.130075 | 0.530263915 |  | 0.935073 | 0.499786 | 1.74947 | 0.834023 | 0.917870999 |
| metabolite686 | 0.178868491 | 0.000926 | 34.5567374 | 0.522927 | 0.67451521 |  | 14.89439 | 0.24146 | 918.7547 | 0.201711 | 0.480147062 |  | 1973429.328 | 0.004713777 | 8.26179E+14 | 0.155214 | 0.560993738 |  | 1.335891 | 0.739373 | 2.413675 | 0.339376 | 0.551170141 |
| metabolite687 | 0.013905572 | 4.51E-05 | 4.289081116 | 0.146547 | 0.291425245 |  | 1732.126 | 22.57621 | 132894.8 | 0.001043 | 0.075024912 |  | 504193635.2 | 0.196496662 | 1.29372E+18 | 0.072565 | 0.447917707 |  | 2.187146 | 1.158677 | 4.12851 | 0.017397 | 0.082746117 |
| metabolite688 | 13.3499786 | 0.054406 | 3275.782282 | 0.357982 | 0.530116391 |  | 64.33823 | 0.89042 | 4648.825 | 0.059125 | 0.265670403 |  | 2025.95637 | 1.63367E-06 | 2.51243E+12 | 0.477521 | 0.79982887 |  | 1.13893 | 0.611617 | 2.120873 | 0.682524 | 0.821818663 |
| metabolite689 | 0.088656351 | 0.000219 | 35.92607038 | 0.430676 | 0.598815341 |  | 26.09582 | 0.236757 | 2876.337 | 0.176741 | 0.451402 |  | 96239.78348 | 1.23493E-05 | 7.50007E+14 | 0.325579 | 0.704504181 |  | 1.559103 | 0.795262 | 3.056603 | 0.198687 | 0.396199711 |
| metabolite690 | 2.558149765 | 0.00915 | 715.2175738 | 0.74443 | 0.84980371 |  | 0.109651 | 0.001321 | 9.102176 | 0.329006 | 0.605724591 |  | 186.6762161 | 9.58128E-08 | 3.63709E+11 | 0.63276 | 0.881911308 |  | 0.852189 | 0.451993 | 1.606722 | 0.622028 | 0.777744638 |
| metabolite691 | 44.08793117 | 0.097587 | 19918.04021 | 0.227355 | 0.390371454 |  | 75.02147 | 0.63075 | 8923.056 | 0.079309 | 0.309228471 |  | 182076463 | 0.016744644 | 1.97985E+18 | 0.109553 | 0.512129781 |  | 0.805257 | 0.403101 | 1.608626 | 0.540804 | 0.713699433 |
| metabolite692 | 224.419844 | 0.612479 | 82230.20265 | 0.075016 | 0.180651705 |  | 0.147904 | 0.001342 | 16.30438 | 0.427405 | 0.689909047 |  | 2252.03585 | 3.09398E-07 | 1.6392E+13 | 0.506605 | 0.816726692 |  | 0.520753 | 0.268186 | 1.011177 | 0.056515 | 0.182211423 |
| metabolite693 | 338.5880077 | 0.968332 | 118391.0713 | 0.053791 | 0.142482468 |  | 0.190477 | 0.001767 | 20.531 | 0.488854 | 0.7318014 |  | 0.251630064 | 3.74591E-11 | 1690316450 | 0.905083 | 0.970355785 |  | 0.551107 | 0.284355 | 1.068098 | 0.080337 | 0.228628175 |
| metabolite694 | 0.031836827 | 0.000133 | 7.631104471 | 0.220159 | 0.383392509 |  | 7.547499 | 0.099596 | 571.9588 | 0.361982 | 0.635338624 |  | 165387.9608 | 0.000147201 | 1.85822E+14 | 0.260861 | 0.647529078 |  | 1.590251 | 0.859422 | 2.942555 | 0.142376 | 0.32414565 |
| metabolite695 | 195.8550961 | 1.052947 | 36430.3504 | 0.050252 | 0.135429596 |  | 5.084085 | 0.078085 | 331.0226 | 0.446962 | 0.699880306 |  | 32.19827936 | 5.48365E-08 | 18905808711 | 0.736729 | 0.92772069 |  | 0.752607 | 0.414449 | 1.366673 | 0.352469 | 0.563202842 |
| metabolite696 | 0.000604209 | 2.92E-06 | 0.125120884 | 0.007497 | 0.031794621 |  | 20.44811 | 0.277136 | 1508.736 | 0.171828 | 0.443346793 |  | 225.7640629 | 1.8853E-07 | 2.70352E+11 | 0.612354 | 0.875701065 |  | 2.456032 | 1.351228 | 4.464154 | 0.003907 | 0.031047908 |
| metabolite697 | 98.57135711 | 0.582461 | 16681.49514 | 0.082269 | 0.194457691 |  | 0.623564 | 0.010383 | 37.44752 | 0.821581 | 0.928972215 |  | 211730.7988 | 0.00063129 | 7.10133E+13 | 0.2234 | 0.616385807 |  | 0.620313 | 0.347603 | 1.106977 | 0.108921 | 0.277043621 |
| metabolite698 | 0.409793535 | 0.001907 | 88.08127942 | 0.74535 | 0.84980371 |  | 33.83867 | 0.517754 | 2211.581 | 0.101497 | 0.343190353 |  | 1528535.71 | 0.002477781 | 9.42949E+14 | 0.170685 | 0.568636202 |  | 1.318934 | 0.72167 | 2.410502 | 0.370188 | 0.585828607 |
| metabolite699 | 0.377412275 | 0.001521 | 93.62612476 | 0.729711 | 0.839623847 |  | 8.114914 | 0.107216 | 614.197 | 0.344951 | 0.619609394 |  | 17186736.47 | 0.017094073 | 1.72799E+16 | 0.118044 | 0.519645256 |  | 1.296228 | 0.69763 | 2.408449 | 0.413489 | 0.618733161 |
| metabolite700 | 0.185372124 | 0.000761 | 45.13934928 | 0.548971 | 0.700319896 |  | 41.45008 | 0.574683 | 2989.662 | 0.090761 | 0.327151227 |  | 811084.1306 | 0.000783893 | 8.39219E+14 | 0.201555 | 0.597241307 |  | 1.440621 | 0.777887 | 2.667984 | 0.248079 | 0.452839125 |
| metabolite701 | 0.106742824 | 0.000353 | 32.25611247 | 0.444212 | 0.611385737 |  | 52.5364 | 0.614514 | 4491.473 | 0.083672 | 0.315235932 |  | 869633.3891 | 0.000359842 | 2.10165E+15 | 0.217359 | 0.607850975 |  | 1.265416 | 0.664907 | 2.408274 | 0.47489 | 0.668303921 |
| metabolite702 | 0.000162931 | 4.93E-07 | 0.053818622 | 0.003906 | 0.019047359 |  | 553.0789 | 5.588698 | 54734.79 | 0.008156 | 0.126652263 |  | 12.59546809 | 1.46201E-09 | 1.08512E+11 | 0.82857 | 0.952506813 |  | 2.950771 | 1.543755 | 5.640173 | 0.001416 | 0.016109823 |
| metabolite703 | 0.004802481 | 1.57E-05 | 1.468231728 | 0.070166 | 0.171659066 |  | 63.01082 | 0.694239 | 5719.013 | 0.074364 | 0.299311934 |  | 4144276.036 | 0.001298479 | 1.3227E+16 | 0.175105 | 0.573796732 |  | 1.817441 | 0.953802 | 3.463081 | 0.072037 | 0.212842663 |
| metabolite704 | 0.000113785 | 5.32E-07 | 0.024334495 | 0.001229 | 0.008142837 |  | 427.1759 | 5.891529 | 30973.16 | 0.006545 | 0.121443014 |  | 1926569.947 | 0.001195539 | 3.1046E+15 | 0.183669 | 0.5811057 |  | 2.782177 | 1.520289 | 5.091471 | 0.001224 | 0.014375745 |
| metabolite705 | 0.000173121 | 7.25E-07 | 0.041336094 | 0.002449 | 0.013648009 |  | 206.443 | 2.576119 | 16543.76 | 0.018867 | 0.16512884 |  | 341681.9136 | 0.000148783 | 7.84679E+14 | 0.249099 | 0.640761773 |  | 2.962694 | 1.60924 | 5.454472 | 0.000699 | 0.010015017 |
| metabolite706 | 0.000464687 | 2E-06 | 0.107972135 | 0.006749 | 0.029293636 |  | 5.268054 | 0.063126 | 439.6321 | 0.463203 | 0.713133835 |  | 435.4037913 | 2.28492E-07 | 8.29685E+11 | 0.57841 | 0.85722005 |  | 2.61865 | 1.424824 | 4.812752 | 0.002451 | 0.02326622 |
| metabolite707 | 0.004079223 | 1.31E-05 | 1.272044321 | 0.063023 | 0.159125058 |  | 26.3475 | 0.27768 | 2499.967 | 0.161812 | 0.430111247 |  | 3.46692E+11 | 143.9405231 | 8.35035E+20 | 0.017556 | 0.269671483 |  | 1.718208 | 0.897643 | 3.288878 | 0.105087 | 0.270818784 |
| metabolite708 | 0.000422733 | 1.33E-06 | 0.134084956 | 0.009385 | 0.037810894 |  | 561.8826 | 6.087102 | 51865.73 | 0.007114 | 0.122988815 |  | 137896662.6 | 0.028816744 | 6.59876E+17 | 0.102159 | 0.49665678 |  | 2.646894 | 1.390278 | 5.039316 | 0.003728 | 0.030404702 |
| metabolite709 | 3.061959659 | 0.007403 | 1266.415314 | 0.716515 | 0.831193068 |  | 3.042118 | 0.026515 | 349.0267 | 0.646565 | 0.834865326 |  | 27165.38519 | 3.32229E-06 | 2.22123E+14 | 0.382526 | 0.741063249 |  | 0.801708 | 0.407052 | 1.579001 | 0.524077 | 0.703958754 |
| metabolite710 | 0.056414352 | 0.000202 | 15.79099025 | 0.319435 | 0.492285398 |  | 5.349988 | 0.06274 | 456.2078 | 0.461247 | 0.712136233 |  | 0.366679893 | 1.68314E-10 | 798828309.6 | 0.927298 | 0.977264574 |  | 1.37413 | 0.728366 | 2.592423 | 0.328559 | 0.541661886 |
| metabolite711 | 7.829510471 | 0.021127 | 2901.619458 | 0.496727 | 0.654599796 |  | 225.0808 | 2.360648 | 21460.78 | 0.021647 | 0.174688709 |  | 1.3463E+17 | 76317661.29 | 2.37499E+26 | 0.000429 | 0.047140629 |  | 1.091735 | 0.560063 | 2.128126 | 0.797096 | 0.895014774 |
| metabolite712 | 50.29338095 | 0.155756 | 16239.67096 | 0.186519 | 0.342180649 |  | 5.007442 | 0.051577 | 486.1542 | 0.4916 | 0.733951068 |  | 2923.707674 | 7.57844E-07 | 1.12794E+13 | 0.480036 | 0.799916815 |  | 0.913972 | 0.474421 | 1.760766 | 0.788513 | 0.889683784 |
| metabolite713 | 2000.793579 | 6.556159 | 610597.6236 | 0.010468 | 0.041063999 |  | 0.072501 | 0.000718 | 7.319725 | 0.267451 | 0.555007395 |  | 0.218739926 | 4.14275E-11 | 1154962424 | 0.894383 | 0.96870562 |  | 0.420939 | 0.221076 | 0.80149 | 0.009657 | 0.055732714 |
| metabolite714 | 16.92784982 | 0.053633 | 5342.830875 | 0.337369 | 0.511428437 |  | 218.4195 | 2.580064 | 18490.65 | 0.01909 | 0.165250229 |  | 1.07871E+13 | 6484.193967 | 1.79454E+22 | 0.006568 | 0.1721236 |  | 1.000261 | 0.521713 | 1.917762 | 0.999375 | 0.999603104 |
| metabolite715 | 0.000465661 | 9.22E-07 | 0.23524475 | 0.017339 | 0.06081899 |  | 13.30839 | 0.089145 | 1986.792 | 0.313045 | 0.591374838 |  | 0.471462327 | 1.36819E-11 | 16246024875 | 0.951675 | 0.986747222 |  | 2.781149 | 1.389742 | 5.56563 | 0.004637 | 0.034902214 |
| metabolite716 | 569.6245996 | 1.667867 | 194543.1803 | 0.035222 | 0.103738178 |  | 5.47986 | 0.051056 | 588.1573 | 0.477317 | 0.722694377 |  | 42930.77851 | 7.089E-06 | 2.59987E+14 | 0.355297 | 0.720888677 |  | 0.640726 | 0.329422 | 1.246209 | 0.192389 | 0.389075739 |
| metabolite717 | 39.51630558 | 0.129682 | 12041.29296 | 0.210316 | 0.372012863 |  | 87.84237 | 1.016959 | 7587.602 | 0.051633 | 0.254388663 |  | 7.54263E+11 | 430.4068657 | 1.3218E+21 | 0.013212 | 0.240490866 |  | 0.90968 | 0.475568 | 1.740062 | 0.77536 | 0.880465881 |
| metabolite718 | 0.597146745 | 0.002034 | 175.2965515 | 0.859164 | 0.923091692 |  | 293.8349 | 3.799001 | 22726.76 | 0.011759 | 0.141133863 |  | 1.69973E+12 | 1361.508344 | 2.12197E+21 | 0.009609 | 0.200325146 |  | 1.274356 | 0.672947 | 2.413239 | 0.458337 | 0.655195388 |
| metabolite719 | 0.014267681 | 5.15E-05 | 3.954731861 | 0.141469 | 0.28498181 |  | 269.2564 | 3.47581 | 20858.17 | 0.01311 | 0.14698155 |  | 609506.3041 | 0.000298669 | 1.24385E+15 | 0.225838 | 0.618474536 |  | 1.647392 | 0.874746 | 3.102502 | 0.125036 | 0.301398257 |
| metabolite720 | 0.277326129 | 0.001107 | 69.44913687 | 0.649896 | 0.78253229 |  | 4.310987 | 0.055934 | 332.2595 | 0.511158 | 0.749173391 |  | 4170309.104 | 0.003820638 | 4.55198E+15 | 0.153912 | 0.559966401 |  | 1.247485 | 0.670173 | 2.322114 | 0.486925 | 0.675758223 |
| metabolite721 | 9.425866444 | 0.030563 | 2907.044305 | 0.444589 | 0.611385737 |  | 31.45274 | 0.356667 | 2773.668 | 0.134165 | 0.394835088 |  | 21281.47434 | 7.59445E-06 | 5.96358E+13 | 0.371184 | 0.736141064 |  | 0.921831 | 0.482625 | 1.760729 | 0.805732 | 0.900624278 |
| metabolite722 | 1.007522877 | 0.002418 | 419.7471499 | 0.998061 | 0.9990055 |  | 39.05223 | 0.354902 | 4297.178 | 0.129338 | 0.386362383 |  | 15943694.38 | 0.002189267 | 1.16113E+17 | 0.15512 | 0.560993738 |  | 0.923962 | 0.468421 | 1.822517 | 0.819924 | 0.91004857 |
| metabolite723 | 252.4773246 | 1.055993 | 60364.79727 | 0.050236 | 0.135429596 |  | 88.21226 | 1.187003 | 6555.504 | 0.043928 | 0.236415195 |  | 19023.72362 | 1.31543E-05 | 2.75121E+13 | 0.361844 | 0.728991162 |  | 0.89782 | 0.479423 | 1.681355 | 0.736956 | 0.857586607 |
| metabolite724 | 12.15073385 | 0.041837 | 3528.931365 | 0.38995 | 0.559375655 |  | 34.06863 | 0.404892 | 2866.623 | 0.121559 | 0.375256628 |  | 250322.9687 | 0.000115726 | 5.41467E+14 | 0.259457 | 0.647529078 |  | 0.860528 | 0.45359 | 1.632551 | 0.646587 | 0.797274851 |
| metabolite725 | 8.90965E-05 | 3.89E-07 | 0.020387363 | 0.001054 | 0.007222029 |  | 15.42236 | 0.177952 | 1336.591 | 0.232025 | 0.513136671 |  | 14.38663447 | 5.6265E-09 | 36785783817 | 0.809807 | 0.9460629 |  | 2.864308 | 1.553438 | 5.281358 | 0.001031 | 0.012756973 |
| metabolite726 | 994.7506534 | 2.384375 | 415005.543 | 0.026931 | 0.085346461 |  | 0.93529 | 0.007267 | 120.373 | 0.978513 | 0.990088079 |  | 143.4865382 | 9.69513E-09 | 2.12358E+12 | 0.678463 | 0.903557902 |  | 0.413681 | 0.210595 | 0.812613 | 0.011736 | 0.063765457 |
| metabolite727 | 1.613574313 | 0.004881 | 533.3785694 | 0.871866 | 0.930364047 |  | 3.066667 | 0.031969 | 294.1742 | 0.631265 | 0.826559459 |  | 3891588661 | 1.533209223 | 9.87762E+18 | 0.048087 | 0.384827718 |  | 0.813528 | 0.423703 | 1.56201 | 0.53649 | 0.710787102 |
| metabolite728 | 11.95815488 | 0.069462 | 2058.650025 | 0.346875 | 0.519682799 |  | 63.28738 | 1.163167 | 3443.439 | 0.044323 | 0.237074035 |  | 7637.033024 | 2.42539E-05 | 2.40474E+12 | 0.37243 | 0.736141064 |  | 1.072501 | 0.599234 | 1.919547 | 0.814118 | 0.904548154 |
| metabolite729 | 0.009230951 | 3.42E-05 | 2.490046022 | 0.103721 | 0.229249448 |  | 514.5834 | 6.933589 | 38190.33 | 0.005349 | 0.113768772 |  | 1.51883E+11 | 117.1930678 | 1.96843E+20 | 0.017827 | 0.270019734 |  | 1.596373 | 0.848398 | 3.003786 | 0.149809 | 0.333698345 |
| metabolite730 | 0.107234443 | 0.000343 | 33.54407762 | 0.447881 | 0.614243311 |  | 159.8566 | 1.891617 | 13509.15 | 0.026977 | 0.193061777 |  | 2378327824 | 1.07864054 | 5.24405E+18 | 0.051691 | 0.392904029 |  | 1.369337 | 0.717554 | 2.613159 | 0.342489 | 0.555253967 |
| metabolite731 | 0.015404344 | 6.25E-05 | 3.793668996 | 0.140271 | 0.283831631 |  | 320.5318 | 4.593661 | 22365.75 | 0.008874 | 0.126652263 |  | 93971.97886 | 6.9892E-05 | 1.26348E+14 | 0.287948 | 0.67078217 |  | 1.655757 | 0.891316 | 3.075827 | 0.113361 | 0.284396398 |
| metabolite732 | 20.30080301 | 0.073491 | 5607.786963 | 0.296114 | 0.467608695 |  | 15.11753 | 0.182007 | 1255.664 | 0.230984 | 0.512158638 |  | 25665256.18 | 0.015523568 | 4.24326E+16 | 0.118017 | 0.519645256 |  | 0.656073 | 0.348908 | 1.233654 | 0.193494 | 0.390036726 |
| metabolite733 | 0.009802455 | 4.72E-05 | 2.037723216 | 0.0922 | 0.211206194 |  | 1495.216 | 26.47665 | 84439.36 | 0.000563 | 0.07423496 |  | 46014779404 | 92.62600023 | 2.28592E+19 | 0.017906 | 0.27012414 |  | 1.952596 | 1.075841 | 3.543859 | 0.029851 | 0.121867646 |
| metabolite734 | 0.041353802 | 0.000177 | 9.643218347 | 0.254566 | 0.420238522 |  | 541.8681 | 8.486914 | 34596.91 | 0.003668 | 0.104558924 |  | 3301036.511 | 0.003563643 | 3.05778E+15 | 0.156997 | 0.561539131 |  | 1.900684 | 1.036826 | 3.484287 | 0.040113 | 0.14522998 |
| metabolite735 | 0.004103544 | 3.45E-05 | 0.488537686 | 0.026176 | 0.083898538 |  | 0.031672 | 0.000712 | 1.408505 | 0.077305 | 0.305191899 |  | 0.000460598 | 4.19443E-12 | 50579.09668 | 0.417757 | 0.770141719 |  | 1.341101 | 0.775424 | 2.319444 | 0.295987 | 0.510413269 |
| metabolite736 | 0.728094404 | 0.001903 | 278.6148992 | 0.916897 | 0.956132096 |  | 78.95602 | 0.784005 | 7951.544 | 0.066021 | 0.281937775 |  | 59662.58692 | 1.00664E-05 | 3.53615E+14 | 0.340247 | 0.713874057 |  | 0.979734 | 0.501383 | 1.91446 | 0.95234 | 0.976798096 |
| metabolite737 | 0.150261414 | 0.000331 | 68.29765535 | 0.545031 | 0.696730418 |  | 18.04383 | 0.14902 | 2184.81 | 0.239694 | 0.521092519 |  | 3483277.93 | 0.000317783 | 3.81808E+16 | 0.204218 | 0.597241307 |  | 1.300264 | 0.653039 | 2.588953 | 0.456483 | 0.654106496 |
| metabolite738 | 8.697189264 | 0.033526 | 2256.209182 | 0.447254 | 0.614043629 |  | 161.3291 | 2.221378 | 11716.63 | 0.021888 | 0.175432524 |  | 6515.80011 | 4.42694E-06 | 9.5903E+12 | 0.416596 | 0.770009271 |  | 1.173251 | 0.626718 | 2.196392 | 0.61846 | 0.7751112 |
| metabolite739 | 0.052912785 | 0.000225 | 12.44483568 | 0.293723 | 0.465019882 |  | 23.95753 | 0.331405 | 1731.908 | 0.148679 | 0.413913431 |  | 4432065.424 | 0.004751557 | 4.13406E+15 | 0.149222 | 0.549913638 |  | 1.616098 | 0.876658 | 2.979238 | 0.126852 | 0.303882925 |
| metabolite740 | 0.004195549 | 1.3E-05 | 1.353633278 | 0.065925 | 0.16419265 |  | 170.3052 | 1.856558 | 15622.38 | 0.027872 | 0.194420599 |  | 5.6591E+11 | 211.2934693 | 1.51569E+21 | 0.016128 | 0.260225096 |  | 1.327991 | 0.687343 | 2.565766 | 0.400367 | 0.612180857 |
| metabolite741 | 0.547129877 | 0.000606 | 494.1404678 | 0.862437 | 0.925000983 |  | 97.5611 | 0.49098 | 19386.06 | 0.092589 | 0.329911989 |  | 1.64958E+11 | 1.520171311 | 1.79001E+22 | 0.048792 | 0.385659329 |  | 0.728149 | 0.339021 | 1.56392 | 0.417724 | 0.623059642 |
| metabolite742 | 16.32000052 | 0.065715 | 4052.992297 | 0.323145 | 0.496148559 |  | 182.1295 | 2.5901 | 12806.89 | 0.018125 | 0.163741265 |  | 13958.67512 | 1.09105E-05 | 1.78584E+13 | 0.374296 | 0.736141064 |  | 1.141211 | 0.611791 | 2.128772 | 0.678753 | 0.818110718 |
| metabolite743 | 0.023283957 | 9.8E-05 | 5.533538871 | 0.180701 | 0.33529062 |  | 97.75348 | 1.3825 | 6911.93 | 0.037187 | 0.220921127 |  | 25916172.86 | 0.026212124 | 2.56236E+16 | 0.109064 | 0.511030336 |  | 1.47159 | 0.79395 | 2.727598 | 0.222372 | 0.420601966 |
| metabolite744 | 2.18575886 | 0.009339 | 511.5521853 | 0.779282 | 0.87149173 |  | 153.1176 | 2.309297 | 10152.43 | 0.020482 | 0.168909139 |  | 10590401.16 | 0.013051126 | 8.59363E+15 | 0.125084 | 0.527043366 |  | 1.066368 | 0.576748 | 1.971641 | 0.83801 | 0.919530925 |
| metabolite745 | 0.049587407 | 0.000175 | 14.07009349 | 0.299464 | 0.471294638 |  | 21.3553 | 0.253717 | 1797.471 | 0.178624 | 0.453992551 |  | 236275901.7 | 0.137790002 | 4.05155E+17 | 0.078259 | 0.45805738 |  | 1.436093 | 0.760466 | 2.711972 | 0.266914 | 0.475296031 |
| metabolite746 | 0.056111125 | 0.0002 | 15.7280426 | 0.318652 | 0.492195492 |  | 52.80246 | 0.650573 | 4285.606 | 0.079751 | 0.309899962 |  | 9051.386522 | 4.41364E-06 | 1.85624E+13 | 0.406734 | 0.762519149 |  | 1.505807 | 0.799479 | 2.836166 | 0.207742 | 0.405325803 |
| metabolite747 | 0.038517493 | 0.000143 | 10.40841966 | 0.25675 | 0.423090534 |  | 43.2771 | 0.542543 | 3452.09 | 0.094545 | 0.33333026 |  | 79491.13745 | 4.49361E-05 | 1.40618E+14 | 0.301251 | 0.686275285 |  | 1.526396 | 0.813365 | 2.864499 | 0.190619 | 0.386129212 |
| metabolite748 | 0.965706414 | 0.003586 | 260.041128 | 0.99027 | 0.995366067 |  | 30.30661 | 0.387114 | 2372.662 | 0.128021 | 0.38468378 |  | 4301949.825 | 0.003045754 | 6.07625E+15 | 0.158124 | 0.563073112 |  | 1.143728 | 0.609257 | 2.147065 | 0.676808 | 0.817653672 |
| metabolite749 | 5.225105717 | 0.01723 | 1584.557089 | 0.571783 | 0.717381921 |  | 3.343802 | 0.037115 | 301.2537 | 0.60017 | 0.807553091 |  | 132754128.7 | 0.063415138 | 2.77909E+17 | 0.090409 | 0.479980149 |  | 1.043577 | 0.547759 | 1.988196 | 0.897038 | 0.951660568 |
| metabolite750 | 0.043858926 | 0.000215 | 8.953100363 | 0.2517 | 0.418484923 |  | 997.0456 | 18.03227 | 55128.94 | 0.001025 | 0.075024912 |  | 270228358.8 | 0.554673541 | 1.31651E+17 | 0.05973 | 0.415087442 |  | 1.716697 | 0.94759 | 3.110046 | 0.077411 | 0.223038637 |
| metabolite751 | 0.01079939 | 4.79E-05 | 2.435237058 | 0.104245 | 0.230036676 |  | 717.8384 | 11.41925 | 45124.86 | 0.002358 | 0.088837289 |  | 1072348389 | 1.381021514 | 8.32667E+17 | 0.048948 | 0.385659329 |  | 1.855925 | 1.011607 | 3.404935 | 0.048242 | 0.163644381 |
| metabolite752 | 2530.402381 | 7.973162 | 803061.0733 | 0.008811 | 0.035796367 |  | 0.173025 | 0.001626 | 18.41241 | 0.462855 | 0.713015197 |  | 0.037898107 | 6.00618E-12 | 239131373 | 0.776729 | 0.940047404 |  | 0.473798 | 0.246188 | 0.911839 | 0.027331 | 0.114058064 |
| metabolite753 | 0.080834665 | 0.000282 | 23.1949647 | 0.385553 | 0.554783119 |  | 3.905505 | 0.044959 | 339.2675 | 0.550974 | 0.780026154 |  | 3.457241524 | 1.4782E-09 | 8085883411 | 0.91047 | 0.972863198 |  | 0.951833 | 0.502109 | 1.804359 | 0.880026 | 0.940701625 |
| metabolite754 | 2.619792865 | 0.009682 | 708.8912329 | 0.736718 | 0.844858078 |  | 100.7754 | 1.329385 | 7639.388 | 0.039 | 0.223736893 |  | 54.07715172 | 3.11213E-08 | 93965668727 | 0.713863 | 0.920092212 |  | 1.150633 | 0.612428 | 2.161813 | 0.663621 | 0.808502548 |
| metabolite755 | 48.04501416 | 0.231669 | 9963.885906 | 0.157634 | 0.306432626 |  | 84.12475 | 1.315807 | 5378.428 | 0.038959 | 0.223736893 |  | 1564.459693 | 2.15011E-06 | 1.13833E+12 | 0.481356 | 0.800476896 |  | 0.949723 | 0.517964 | 1.741384 | 0.867855 | 0.937004424 |
| metabolite756 | 6472.948899 | 17.74294 | 2361450.207 | 0.004297 | 0.020515633 |  | 0.117354 | 0.000962 | 14.31035 | 0.383879 | 0.655278308 |  | 2.480382236 | 1.97291E-10 | 31183817064 | 0.939108 | 0.981248617 |  | 0.397891 | 0.204047 | 0.775886 | 0.007914 | 0.049357439 |
| metabolite757 | 121.8902829 | 0.438564 | 33877.0486 | 0.09716 | 0.218919995 |  | 98.44068 | 1.202823 | 8056.519 | 0.043504 | 0.236415195 |  | 40033268494 | 25.72310996 | 6.23044E+19 | 0.025727 | 0.298662816 |  | 0.97391 | 0.512617 | 1.85031 | 0.935798 | 0.967936596 |
| metabolite758 | 0.018430647 | 3.49E-05 | 9.730181995 | 0.214422 | 0.37640408 |  | 25.29752 | 0.181981 | 3516.659 | 0.202084 | 0.480506361 |  | 2.6173E+13 | 2020.008756 | 3.39121E+23 | 0.010572 | 0.212061921 |  | 1.449239 | 0.714136 | 2.94103 | 0.306384 | 0.519886204 |
| metabolite759 | 1.055372575 | 0.00352 | 316.3800903 | 0.985256 | 0.992378901 |  | 46.08912 | 0.546963 | 3883.642 | 0.093206 | 0.330362211 |  | 397401880.9 | 0.21199137 | 7.44975E+17 | 0.071822 | 0.447917707 |  | 1.244734 | 0.655617 | 2.363213 | 0.504696 | 0.691459815 |
| metabolite760 | 0.00297731 | 9.43E-06 | 0.939865402 | 0.05005 | 0.135081626 |  | 173.3718 | 1.906634 | 15764.84 | 0.027053 | 0.193061777 |  | 842479.0736 | 0.000212381 | 3.34197E+15 | 0.22885 | 0.621260608 |  | 1.846024 | 0.964058 | 3.534851 | 0.067037 | 0.20307985 |
| metabolite761 | 0.029057564 | 0.00014 | 6.02378105 | 0.196232 | 0.353104799 |  | 2.70685 | 0.039462 | 185.6722 | 0.645271 | 0.833774355 |  | 4155.166383 | 5.97224E-06 | 2.89094E+12 | 0.424215 | 0.772928355 |  | 1.374508 | 0.752461 | 2.510792 | 0.303012 | 0.516526976 |
| metabolite762 | 4.410190427 | 0.014818 | 1312.582122 | 0.610622 | 0.749097317 |  | 471.4791 | 6.13454 | 36236.22 | 0.006408 | 0.120104745 |  | 7.46818E+13 | 66850.44732 | 8.34305E+22 | 0.003282 | 0.120572584 |  | 1.23605 | 0.651029 | 2.346774 | 0.518406 | 0.699310535 |
| metabolite763 | 0.001285175 | 4.36E-06 | 0.379164807 | 0.023659 | 0.077371719 |  | 15.81824 | 0.166431 | 1503.425 | 0.237255 | 0.518127226 |  | 3865.121576 | 1.01614E-06 | 1.47018E+13 | 0.464562 | 0.792880411 |  | 1.877973 | 0.985266 | 3.579525 | 0.05808 | 0.184531753 |
| metabolite764 | 0.156659961 | 0.000605 | 40.53610092 | 0.514503 | 0.668139337 |  | 1001.21 | 15.15411 | 66148.45 | 0.001622 | 0.082989557 |  | 50387268.42 | 0.042668165 | 5.95028E+16 | 0.098925 | 0.492678215 |  | 1.919713 | 1.037702 | 3.551405 | 0.040024 | 0.145046173 |
| metabolite765 | 0.010214556 | 1.52E-05 | 6.856904931 | 0.170273 | 0.322719513 |  | 23.49205 | 0.138163 | 3994.372 | 0.230901 | 0.512158638 |  | 429.2258871 | 6.48613E-09 | 2.84044E+13 | 0.634392 | 0.882069794 |  | 1.541825 | 0.739268 | 3.215648 | 0.250782 | 0.4555323 |
| metabolite766 | 0.339006689 | 0.001304 | 88.13827339 | 0.703719 | 0.822779245 |  | 759.0727 | 11.33419 | 50836.58 | 0.002516 | 0.089719453 |  | 478.8514652 | 3.25712E-07 | 7.03992E+11 | 0.567782 | 0.852017603 |  | 1.595712 | 0.857802 | 2.968397 | 0.142866 | 0.324662759 |
| metabolite767 | 0.074450679 | 0.000236 | 23.44462229 | 0.378015 | 0.547616871 |  | 18.32653 | 0.200912 | 1671.682 | 0.209224 | 0.488014005 |  | 340.6711399 | 1.04589E-07 | 1.10965E+12 | 0.60288 | 0.872650335 |  | 2.025916 | 1.07166 | 3.829887 | 0.031905 | 0.127108339 |
| metabolite768 | 9.77212126 | 0.03351 | 2849.69097 | 0.432823 | 0.60067596 |  | 53.65612 | 0.645782 | 4458.126 | 0.080131 | 0.31022869 |  | 19915.75547 | 8.76806E-06 | 4.52366E+13 | 0.369742 | 0.734930036 |  | 1.228111 | 0.647624 | 2.328909 | 0.530412 | 0.708331897 |
| metabolite769 | 0.771413791 | 0.001247 | 477.3153452 | 0.937065 | 0.96945857 |  | 0.009342 | 6.38E-05 | 1.36776 | 0.0689 | 0.290456173 |  | 5.13916E-06 | 1.41511E-16 | 186635.3553 | 0.328392 | 0.705196155 |  | 0.575784 | 0.281185 | 1.179032 | 0.133984 | 0.310760289 |
| metabolite770 | 1.22301417 | 0.00188 | 795.4386689 | 0.951536 | 0.977075415 |  | 0.022241 | 0.000142 | 3.475089 | 0.14259 | 0.407040102 |  | 0.000138229 | 2.99735E-15 | 6374689.15 | 0.479594 | 0.79982887 |  | 0.668149 | 0.323349 | 1.380625 | 0.278522 | 0.489856343 |
| metabolite771 | 4113.829079 | 7.205554 | 2348686.792 | 0.0115 | 0.044225833 |  | 0.070873 | 0.000423 | 11.87198 | 0.313242 | 0.591374838 |  | 0.01043978 | 1.75853E-13 | 619773959.9 | 0.719193 | 0.920542269 |  | 0.424915 | 0.207142 | 0.871638 | 0.021354 | 0.094595828 |
| metabolite772 | 970.8495415 | 2.727828 | 345530.8295 | 0.023624 | 0.077371719 |  | 0.484788 | 0.004268 | 55.06342 | 0.76484 | 0.902319648 |  | 58.39317771 | 7.10392E-09 | 4.79983E+11 | 0.727616 | 0.924067425 |  | 0.611906 | 0.312791 | 1.19706 | 0.154202 | 0.338576282 |
| metabolite773 | 12.33820051 | 0.032062 | 4748.041004 | 0.409828 | 0.578277789 |  | 51.69375 | 0.497105 | 5375.61 | 0.098728 | 0.341771805 |  | 599022492.9 | 0.115220689 | 3.11427E+18 | 0.079359 | 0.460912725 |  | 1.138487 | 0.58132 | 2.22967 | 0.706 | 0.837317042 |
| metabolite774 | 1.441212726 | 0.00653 | 318.0628828 | 0.894642 | 0.943611806 |  | 357.4076 | 5.88266 | 21714.7 | 0.00593 | 0.118151077 |  | 450855385.8 | 0.786236909 | 2.58536E+17 | 0.055333 | 0.402327874 |  | 1.301831 | 0.710217 | 2.386261 | 0.395395 | 0.607270747 |
| metabolite775 | 0.021964252 | 8.25E-05 | 5.848825083 | 0.182947 | 0.336930729 |  | 230.4625 | 3.077663 | 17257.56 | 0.015012 | 0.152336933 |  | 731238.5419 | 0.00043621 | 1.22581E+15 | 0.215388 | 0.605810552 |  | 1.632266 | 0.871481 | 3.0572 | 0.128775 | 0.305745899 |
| metabolite776 | 0.146635776 | 0.00058 | 37.05593863 | 0.497818 | 0.655572208 |  | 1792.349 | 28.62591 | 112224 | 0.000568 | 0.07423496 |  | 2472368115 | 2.597432161 | 2.35333E+18 | 0.042672 | 0.369987647 |  | 1.621278 | 0.873953 | 3.007649 | 0.128203 | 0.305518079 |
| metabolite777 | 0.022159421 | 6.46E-05 | 7.599462331 | 0.203543 | 0.362973998 |  | 85.9816 | 0.903349 | 8183.814 | 0.057903 | 0.264422304 |  | 372620131.6 | 0.100146639 | 1.38642E+18 | 0.08196 | 0.46533994 |  | 1.662 | 0.862799 | 3.20149 | 0.131654 | 0.308440007 |
| metabolite778 | 0.38741205 | 0.001466 | 102.3907741 | 0.739568 | 0.847340831 |  | 163.4917 | 2.239953 | 11933.08 | 0.021701 | 0.174688709 |  | 1.08727E+11 | 115.2343199 | 1.02586E+20 | 0.017586 | 0.269671483 |  | 1.445001 | 0.773629 | 2.699004 | 0.250647 | 0.455510458 |
| metabolite779 | 295.7459024 | 0.778782 | 112310.8149 | 0.063075 | 0.159125058 |  | 1.80727 | 0.015644 | 208.7801 | 0.807503 | 0.924634528 |  | 97011.78156 | 1.19042E-05 | 7.90584E+14 | 0.326188 | 0.704745603 |  | 0.877283 | 0.444833 | 1.730146 | 0.706259 | 0.837356907 |
| metabolite780 | 180.0919315 | 0.214965 | 150876.0173 | 0.133289 | 0.27311593 |  | 1.286683 | 0.006084 | 272.1327 | 0.926647 | 0.974037858 |  | 0.298787892 | 1.8047E-12 | 49467707873 | 0.927136 | 0.977264574 |  | 0.648812 | 0.302918 | 1.389676 | 0.268019 | 0.476807127 |
| metabolite781 | 96.5974819 | 0.21964 | 42483.40417 | 0.143885 | 0.287382453 |  | 0.305361 | 0.002429 | 38.39378 | 0.631487 | 0.826559459 |  | 2031794.295 | 0.000172278 | 2.39624E+16 | 0.222213 | 0.615668421 |  | 0.766015 | 0.384045 | 1.527892 | 0.450844 | 0.647979801 |
| metabolite782 | 0.005815147 | 2.24E-05 | 1.509618207 | 0.072257 | 0.176178744 |  | 127.0144 | 1.639981 | 9837.093 | 0.031148 | 0.204052343 |  | 2717132404 | 1.965882273 | 3.75547E+18 | 0.045482 | 0.380219234 |  | 1.681021 | 0.897077 | 3.15004 | 0.10785 | 0.275553346 |
| metabolite783 | 0.007548913 | 1.45E-05 | 3.939881196 | 0.128732 | 0.267184523 |  | 2.481254 | 0.01712 | 359.625 | 0.721074 | 0.878058466 |  | 4378809.018 | 0.000191287 | 1.00237E+17 | 0.211568 | 0.603798949 |  | 1.336759 | 0.657082 | 2.719483 | 0.424827 | 0.627214456 |
| metabolite784 | 1.613724637 | 0.006204 | 419.7414445 | 0.866369 | 0.927609805 |  | 456.5403 | 6.651308 | 31336.55 | 0.005396 | 0.113768772 |  | 63858275521 | 70.99855089 | 5.74361E+19 | 0.019754 | 0.279023995 |  | 1.28377 | 0.68732 | 2.397813 | 0.434896 | 0.636975815 |
| metabolite785 | 7.215368068 | 0.01793 | 2903.526637 | 0.519719 | 0.672481345 |  | 11.59401 | 0.10445 | 1286.943 | 0.310023 | 0.587997129 |  | 2.01811E+11 | 41.33752588 | 9.8525E+20 | 0.024093 | 0.294657193 |  | 0.972531 | 0.494278 | 1.913529 | 0.935855 | 0.967936596 |
| metabolite786 | 0.196043992 | 0.000384 | 100.1414365 | 0.609576 | 0.748308512 |  | 285.2262 | 2.341471 | 34744.83 | 0.022897 | 0.178511926 |  | 1.7319E+12 | 155.5602727 | 1.92818E+22 | 0.018647 | 0.273520342 |  | 1.249234 | 0.619091 | 2.520768 | 0.535689 | 0.710147095 |
| metabolite787 | 0.342269256 | 0.000852 | 137.5081788 | 0.726642 | 0.838538656 |  | 98.04086 | 0.940849 | 10216.31 | 0.055624 | 0.260304827 |  | 4856687.162 | 0.000735122 | 3.20864E+16 | 0.184756 | 0.58220839 |  | 1.495549 | 0.764074 | 2.92729 | 0.242642 | 0.44664975 |
| metabolite788 | 0.141814824 | 0.000538 | 37.38749782 | 0.493672 | 0.651500018 |  | 249.0054 | 3.455317 | 17944.43 | 0.012873 | 0.146510756 |  | 13166.84633 | 8.58987E-06 | 2.01826E+13 | 0.381292 | 0.741054231 |  | 1.647754 | 0.884314 | 3.070279 | 0.118608 | 0.292540625 |
| metabolite789 | 0.009908044 | 3.16E-05 | 3.104883734 | 0.11842 | 0.251548767 |  | 155.7983 | 1.76831 | 13726.73 | 0.029195 | 0.199529376 |  | 393566.2348 | 0.000115733 | 1.33838E+15 | 0.252403 | 0.642874148 |  | 1.896242 | 0.99623 | 3.609344 | 0.05388 | 0.176944762 |
| metabolite790 | 0.009753747 | 3.67E-05 | 2.592582846 | 0.106883 | 0.23359118 |  | 111.6824 | 1.426019 | 8746.691 | 0.036276 | 0.218561613 |  | 329649603.2 | 0.215454575 | 5.0437E+17 | 0.071801 | 0.447917707 |  | 1.68245 | 0.897064 | 3.155447 | 0.107759 | 0.275553346 |
| metabolite791 | 0.205050025 | 0.000488 | 86.21933248 | 0.608234 | 0.747402693 |  | 131.0061 | 1.220744 | 14059.13 | 0.04336 | 0.236415195 |  | 1.38639E+14 | 32726.69818 | 5.87314E+23 | 0.004784 | 0.149202066 |  | 1.338279 | 0.678569 | 2.639361 | 0.402201 | 0.61293609 |
| metabolite792 | 3.269999628 | 0.013393 | 798.3920827 | 0.673562 | 0.798239357 |  | 11.7947 | 0.158711 | 876.528 | 0.264022 | 0.551213494 |  | 0.073746204 | 6.18953E-11 | 87866131.09 | 0.807284 | 0.9460629 |  | 1.205143 | 0.64908 | 2.237584 | 0.555697 | 0.726886063 |
| metabolite793 | 2.268047601 | 0.009029 | 569.7074921 | 0.772014 | 0.867104274 |  | 4.967621 | 0.064566 | 382.2049 | 0.470952 | 0.717917212 |  | 179895.38 | 0.000153465 | 2.10878E+14 | 0.258521 | 0.64746381 |  | 0.768797 | 0.413246 | 1.430259 | 0.40824 | 0.616474863 |
| metabolite794 | 2.880831926 | 0.003039 | 2730.734788 | 0.76279 | 0.862496413 |  | 19.68812 | 0.091536 | 4234.651 | 0.279186 | 0.566772209 |  | 44210.00714 | 2.31521E-07 | 8.4421E+15 | 0.421316 | 0.77189627 |  | 1.413541 | 0.654689 | 3.05198 | 0.380045 | 0.593649097 |
| metabolite795 | 16.14208619 | 0.069502 | 3749.053878 | 0.319152 | 0.492257977 |  | 0.057284 | 0.000796 | 4.122574 | 0.192645 | 0.470023308 |  | 3742.428899 | 3.70194E-06 | 3.78336E+12 | 0.438374 | 0.782677331 |  | 0.575945 | 0.31363 | 1.057657 | 0.077938 | 0.224208887 |
| metabolite796 | 0.489267456 | 0.001994 | 120.0641025 | 0.799494 | 0.885119735 |  | 6.314754 | 0.08391 | 475.2256 | 0.404978 | 0.673560412 |  | 14495.44224 | 1.29432E-05 | 1.62338E+13 | 0.369382 | 0.734917241 |  | 1.346908 | 0.726343 | 2.497663 | 0.346604 | 0.558994739 |
| metabolite797 | 0.026968826 | 7.75E-05 | 9.384249318 | 0.228812 | 0.391480244 |  | 144.289 | 1.531817 | 13591.26 | 0.034224 | 0.212415725 |  | 2710847.793 | 0.000613435 | 1.19796E+16 | 0.193829 | 0.592990803 |  | 1.375802 | 0.710477 | 2.664169 | 0.346094 | 0.558414501 |
| metabolite798 | 1190.057247 | 4.046041 | 350030.1327 | 0.016186 | 0.057965884 |  | 6.109346 | 0.062515 | 597.0379 | 0.440492 | 0.697085867 |  | 4.5305566 | 1.07109E-09 | 19163676242 | 0.893962 | 0.96870562 |  | 0.636535 | 0.331694 | 1.221538 | 0.177129 | 0.369945101 |
| metabolite799 | 0.540675936 | 0.00084 | 347.9357529 | 0.852492 | 0.918048785 |  | 8.519202 | 0.053118 | 1366.325 | 0.410032 | 0.676881422 |  | 974.1638275 | 2.14366E-08 | 4.42698E+13 | 0.583674 | 0.862610339 |  | 1.348837 | 0.652347 | 2.788949 | 0.42116 | 0.625020256 |
| metabolite800 | 0.207178256 | 0.000259 | 165.4367956 | 0.645205 | 0.779412908 |  | 0.18343 | 0.000955 | 35.23313 | 0.528561 | 0.763431516 |  | 604.5202183 | 5.70929E-09 | 6.40088E+13 | 0.621944 | 0.879305472 |  | 1.307485 | 0.616491 | 2.77298 | 0.486041 | 0.67528941 |
| metabolite801 | 0.003665933 | 1.44E-05 | 0.936295791 | 0.049806 | 0.134519997 |  | 73.38134 | 0.929417 | 5793.765 | 0.056516 | 0.262335019 |  | 218642505.2 | 0.145811829 | 3.27851E+17 | 0.077585 | 0.45805738 |  | 1.71605 | 0.916408 | 3.213447 | 0.094362 | 0.253809654 |
| metabolite802 | 5.864023502 | 0.022377 | 1536.678363 | 0.534831 | 0.68724298 |  | 24.655 | 0.317606 | 1913.909 | 0.151712 | 0.419211984 |  | 8803.657208 | 5.88832E-06 | 1.31624E+13 | 0.401206 | 0.76001764 |  | 0.919635 | 0.490701 | 1.723511 | 0.794257 | 0.89317816 |
| metabolite803 | 0.180383555 | 0.000502 | 64.76351477 | 0.569452 | 0.715865895 |  | 3.710999 | 0.036083 | 381.6648 | 0.580202 | 0.793342471 |  | 1905.577799 | 3.78006E-07 | 9.60626E+12 | 0.508963 | 0.817874416 |  | 1.416567 | 0.731781 | 2.742158 | 0.303683 | 0.51739825 |
| metabolite804 | 0.129193945 | 0.000153 | 109.1313421 | 0.552924 | 0.704245329 |  | 2.886873 | 0.014247 | 584.975 | 0.696382 | 0.867408066 |  | 264155198.3 | 0.002487317 | 2.80535E+19 | 0.137214 | 0.536225468 |  | 1.345149 | 0.63016 | 2.871377 | 0.445067 | 0.643162861 |
| metabolite805 | 0.191442099 | 0.000912 | 40.19069102 | 0.545746 | 0.697164598 |  | 50.26384 | 0.789344 | 3200.7 | 0.067209 | 0.285199729 |  | 4106.238652 | 6.34429E-06 | 2.6577E+12 | 0.423229 | 0.77189627 |  | 1.138317 | 0.622988 | 2.079918 | 0.674389 | 0.817060686 |
| metabolite806 | 0.038129944 | 8.39E-05 | 17.33628224 | 0.2977 | 0.469712129 |  | 332.6274 | 2.96399 | 37328.39 | 0.017544 | 0.161552712 |  | 2.15261E+12 | 281.1858635 | 1.64793E+22 | 0.016031 | 0.259782535 |  | 1.580771 | 0.79491 | 3.143549 | 0.194394 | 0.391101537 |
| metabolite807 | 0.043225298 | 0.000174 | 10.73399798 | 0.266635 | 0.434937794 |  | 473.0577 | 6.996048 | 31987.14 | 0.004991 | 0.110248598 |  | 944632.634 | 0.00078146 | 1.14188E+15 | 0.199912 | 0.597241307 |  | 2.418427 | 1.323725 | 4.418429 | 0.004886 | 0.036335819 |
| metabolite808 | 0.003770337 | 1.61E-05 | 0.880261783 | 0.047306 | 0.130037969 |  | 35.348 | 0.469888 | 2659.105 | 0.108636 | 0.355281522 |  | 926032.1436 | 0.00075012 | 1.1432E+15 | 0.201007 | 0.597241307 |  | 2.090609 | 1.135902 | 3.847733 | 0.019543 | 0.088429298 |
| metabolite809 | 0.09287753 | 0.000365 | 23.63809317 | 0.402226 | 0.571462149 |  | 49.47373 | 0.661171 | 3701.99 | 0.079126 | 0.309091227 |  | 1006874.643 | 0.000798778 | 1.26919E+15 | 0.19874 | 0.597241307 |  | 1.93107 | 1.045221 | 3.567696 | 0.037885 | 0.139751867 |
| metabolite810 | 0.005467948 | 1.26E-05 | 2.380631197 | 0.095728 | 0.216481439 |  | 299.5255 | 2.650094 | 33853.72 | 0.019814 | 0.165250229 |  | 16252711145 | 1.729563406 | 1.52727E+20 | 0.047206 | 0.384050582 |  | 1.948868 | 0.985487 | 3.854018 | 0.057683 | 0.18437727 |
| metabolite811 | 0.12822129 | 0.000508 | 32.3463045 | 0.468187 | 0.63110852 |  | 117.238 | 1.631951 | 8422.286 | 0.031022 | 0.203758819 |  | 958986.2131 | 0.000799524 | 1.15025E+15 | 0.199257 | 0.597241307 |  | 1.540847 | 0.829521 | 2.862145 | 0.173948 | 0.366234906 |
| metabolite812 | 0.000267374 | 8.4E-07 | 0.085116755 | 0.006066 | 0.026733255 |  | 94.20904 | 0.929446 | 9549.069 | 0.05629 | 0.261771456 |  | 168076.3343 | 2.72295E-05 | 1.03746E+15 | 0.297779 | 0.681292933 |  | 2.185303 | 1.134315 | 4.210076 | 0.021249 | 0.094322328 |
| metabolite813 | 0.010992214 | 2.05E-05 | 5.883019499 | 0.162172 | 0.31263432 |  | 35.1929 | 0.250031 | 4953.552 | 0.1611 | 0.429483589 |  | 333690.9916 | 1.3053E-05 | 8.53061E+15 | 0.300519 | 0.685450026 |  | 1.63964 | 0.807726 | 3.328383 | 0.173803 | 0.366234906 |
| metabolite814 | 1877.232989 | 5.681459 | 620263.8084 | 0.012234 | 0.046517222 |  | 0.041198 | 0.000389 | 4.358228 | 0.182646 | 0.45686231 |  | 0.702097929 | 1.00075E-10 | 4925705502 | 0.975662 | 0.991698663 |  | 0.459876 | 0.238532 | 0.886616 | 0.022208 | 0.09776192 |
| metabolite815 | 11863.90508 | 36.53708 | 3852312.753 | 0.001912 | 0.011297434 |  | 0.050471 | 0.000448 | 5.684167 | 0.217945 | 0.498948472 |  | 0.001300018 | 1.45108E-13 | 11646799.83 | 0.570925 | 0.853748966 |  | 0.423478 | 0.218577 | 0.820459 | 0.012252 | 0.065706493 |
| metabolite816 | 39961.8646 | 147.5013 | 10826690.66 | 0.000329 | 0.002903885 |  | 0.085791 | 0.000817 | 9.00749 | 0.303251 | 0.583677462 |  | 0.060764752 | 9.72405E-12 | 379713530.5 | 0.808165 | 0.9460629 |  | 0.380078 | 0.199562 | 0.723883 | 0.003958 | 0.031258922 |
| metabolite817 | 7.03000149 | 0.027272 | 1812.153657 | 0.492602 | 0.650747709 |  | 1.240095 | 0.015515 | 99.11666 | 0.92348 | 0.973312578 |  | 83297.82361 | 6.11026E-05 | 1.13555E+14 | 0.293344 | 0.676990344 |  | 0.825098 | 0.441334 | 1.542565 | 0.548248 | 0.720194646 |
| metabolite818 | 1.317914741 | 0.004635 | 374.7754623 | 0.923885 | 0.960374991 |  | 0.103515 | 0.001234 | 8.683145 | 0.317756 | 0.596153365 |  | 1.6519E-05 | 8.65638E-15 | 31523.3526 | 0.314728 | 0.697572488 |  | 0.769438 | 0.407914 | 1.45137 | 0.419964 | 0.624902667 |
| metabolite819 | 0.005606132 | 2.08E-05 | 1.508354706 | 0.072068 | 0.175833594 |  | 74.37108 | 0.914719 | 6046.726 | 0.057389 | 0.264422304 |  | 14604159.55 | 0.007801725 | 2.73377E+16 | 0.132755 | 0.532024368 |  | 1.651574 | 0.877281 | 3.109261 | 0.122941 | 0.298777281 |
| metabolite820 | 264.3983975 | 1.280416 | 54596.72104 | 0.042632 | 0.120769415 |  | 65.11476 | 0.971642 | 4363.675 | 0.054111 | 0.257328813 |  | 268079.9162 | 0.000335325 | 2.1432E+14 | 0.23461 | 0.626248163 |  | 0.770642 | 0.418834 | 1.417957 | 0.404132 | 0.613639087 |
| metabolite821 | 0.008334619 | 3.3E-05 | 2.104017519 | 0.09261 | 0.212015226 |  | 75.83827 | 0.991148 | 5802.807 | 0.05298 | 0.255757554 |  | 5072905.891 | 0.003515219 | 7.32085E+15 | 0.154139 | 0.560245064 |  | 1.680949 | 0.900992 | 3.136088 | 0.105446 | 0.271004899 |
| metabolite822 | 0.019798661 | 5.63E-05 | 6.956692329 | 0.192419 | 0.348835679 |  | 147.2493 | 1.543838 | 14044.44 | 0.03399 | 0.212352099 |  | 33586.45284 | 6.54752E-06 | 1.72287E+14 | 0.362899 | 0.72913894 |  | 1.758272 | 0.91146 | 3.391835 | 0.095099 | 0.254995174 |
| metabolite823 | 0.422406395 | 0.001482 | 120.3958369 | 0.765636 | 0.864305448 |  | 53.26978 | 0.659896 | 4300.179 | 0.078729 | 0.30884013 |  | 99830258.47 | 0.061426974 | 1.62243E+17 | 0.091522 | 0.484505039 |  | 1.225138 | 0.648699 | 2.313808 | 0.532653 | 0.708485456 |
| metabolite824 | 3.415900095 | 0.010955 | 1065.130527 | 0.675813 | 0.799982026 |  | 86.8595 | 1.015858 | 7426.799 | 0.051687 | 0.254388663 |  | 1980961765 | 0.941058389 | 4.17E+18 | 0.053163 | 0.394365301 |  | 1.135894 | 0.594828 | 2.169123 | 0.700192 | 0.833091879 |
| metabolite825 | 6.775495795 | 0.02496 | 1839.208497 | 0.504752 | 0.660019784 |  | 1.770698 | 0.021303 | 147.1809 | 0.800464 | 0.921663861 |  | 1767.488497 | 1.00544E-06 | 3.1071E+12 | 0.492599 | 0.807152595 |  | 0.672352 | 0.35875 | 1.260088 | 0.218086 | 0.415246994 |
| metabolite826 | 4132.554533 | 14.81334 | 1152880.589 | 0.004523 | 0.02127422 |  | 16.94218 | 0.175716 | 1633.529 | 0.227322 | 0.509702039 |  | 13.70292936 | 3.18271E-09 | 58996981241 | 0.817523 | 0.946906762 |  | 0.546154 | 0.285643 | 1.044254 | 0.070076 | 0.209381208 |
| metabolite827 | 5.846417764 | 0.011668 | 2929.546355 | 0.578837 | 0.722935677 |  | 3.674509 | 0.027466 | 491.5886 | 0.603426 | 0.809000429 |  | 1097724.013 | 6.76592E-05 | 1.78098E+16 | 0.248714 | 0.640761773 |  | 0.888772 | 0.440958 | 1.791363 | 0.742218 | 0.86020302 |
| metabolite828 | 113.3142323 | 0.173924 | 73826.07051 | 0.155273 | 0.303912638 |  | 0.317926 | 0.001854 | 54.52133 | 0.663259 | 0.84717161 |  | 1.564387691 | 2.54439E-11 | 96184364284 | 0.971899 | 0.990751288 |  | 0.68336 | 0.328293 | 1.422451 | 0.310939 | 0.523783032 |
| metabolite829 | 0.025295379 | 5.81E-05 | 11.00752046 | 0.238065 | 0.402598494 |  | 117.9938 | 1.039369 | 13395.19 | 0.050637 | 0.253251612 |  | 59497951.91 | 0.006174714 | 5.73307E+17 | 0.129789 | 0.530263915 |  | 1.957032 | 0.994176 | 3.852412 | 0.054537 | 0.178546467 |
| metabolite830 | 297.3107885 | 0.578743 | 152734.0478 | 0.076462 | 0.183211436 |  | 0.514534 | 0.003524 | 75.13261 | 0.794322 | 0.920287179 |  | 0.065972928 | 2.37564E-12 | 1832103923 | 0.825051 | 0.950563748 |  | 0.534751 | 0.264609 | 1.080681 | 0.083956 | 0.235494365 |
| metabolite831 | 0.163488242 | 0.000482 | 55.44610458 | 0.543622 | 0.695648833 |  | 17.30742 | 0.180177 | 1662.511 | 0.223484 | 0.505543581 |  | 575957716.1 | 0.186122945 | 1.7823E+18 | 0.073127 | 0.449246835 |  | 1.339952 | 0.695939 | 2.579925 | 0.383194 | 0.597744458 |
| metabolite832 | 35987.95996 | 135.0369 | 9590958.423 | 0.00036 | 0.003112115 |  | 0.033983 | 0.000336 | 3.436295 | 0.153844 | 0.421028033 |  | 0.001269704 | 2.27207E-13 | 7095509.376 | 0.561483 | 0.848397879 |  | 0.387844 | 0.203951 | 0.737547 | 0.004657 | 0.034984988 |
| metabolite833 | 260.0157961 | 1.174032 | 57586.34252 | 0.045979 | 0.127333202 |  | 15.93075 | 0.216367 | 1172.954 | 0.209561 | 0.488116163 |  | 10107.48804 | 9.18169E-06 | 1.11266E+13 | 0.387214 | 0.745305825 |  | 0.609507 | 0.330362 | 1.124522 | 0.115941 | 0.287796785 |
| metabolite834 | 934.864893 | 3.674301 | 237860.8559 | 0.017122 | 0.060364804 |  | 2.167115 | 0.024733 | 189.883 | 0.735333 | 0.887270319 |  | 0.009685503 | 4.13287E-12 | 22698257.53 | 0.674376 | 0.902621002 |  | 0.613032 | 0.325301 | 1.155263 | 0.132975 | 0.310359474 |
| metabolite835 | 12.9142561 | 0.05059 | 3296.651414 | 0.367566 | 0.538719752 |  | 5.758379 | 0.07298 | 454.3561 | 0.433828 | 0.694237021 |  | 2020457.795 | 0.001593549 | 2.56173E+15 | 0.177333 | 0.575285157 |  | 0.57502 | 0.309926 | 1.066861 | 0.08206 | 0.232460492 |
| metabolite836 | 0.019163358 | 8.36E-05 | 4.394721116 | 0.156633 | 0.305447068 |  | 156.6334 | 2.313034 | 10606.87 | 0.020545 | 0.169054354 |  | 551584.103 | 0.000569135 | 5.34574E+14 | 0.213097 | 0.6041275 |  | 1.704549 | 0.92637 | 3.13642 | 0.089289 | 0.246724304 |
| metabolite837 | 0.125573945 | 0.000311 | 50.75804243 | 0.499454 | 0.656794916 |  | 561.3649 | 5.715426 | 55136.83 | 0.007912 | 0.126652263 |  | 8233889.137 | 0.001190895 | 5.69294E+16 | 0.171118 | 0.569012497 |  | 1.424186 | 0.725675 | 2.79506 | 0.306237 | 0.519874318 |
| metabolite838 | 4712.976464 | 15.07884 | 1473066.981 | 0.004693 | 0.021963878 |  | 0.280673 | 0.002593 | 30.38053 | 0.596051 | 0.804929177 |  | 19.2091429 | 2.87441E-09 | 1.28371E+11 | 0.798387 | 0.9460629 |  | 0.56071 | 0.289156 | 1.087288 | 0.089631 | 0.247117054 |
| metabolite839 | 0.726510731 | 0.001433 | 368.2523077 | 0.920093 | 0.958311557 |  | 9.41883 | 0.071065 | 1248.358 | 0.370337 | 0.644917822 |  | 70211706.4 | 0.004716905 | 1.04511E+18 | 0.133432 | 0.532435499 |  | 1.206637 | 0.598771 | 2.431603 | 0.600347 | 0.763407527 |
| metabolite840 | 0.136735759 | 0.00033 | 56.60569809 | 0.518848 | 0.672481345 |  | 16.25933 | 0.144152 | 1833.944 | 0.249901 | 0.533591943 |  | 3250788.869 | 0.000421461 | 2.50738E+16 | 0.199418 | 0.597241307 |  | 1.336498 | 0.678527 | 2.632504 | 0.403471 | 0.613137464 |
| metabolite841 | 0.01290148 | 2.64E-05 | 6.300073724 | 0.171191 | 0.323214787 |  | 26.30392 | 0.200078 | 3458.131 | 0.191696 | 0.468324651 |  | 1746080236 | 0.12153395 | 2.5086E+19 | 0.077257 | 0.45805738 |  | 1.78255 | 0.889612 | 3.571765 | 0.105907 | 0.271612079 |
| metabolite842 | 0.003888838 | 1.34E-05 | 1.128530966 | 0.057654 | 0.149691724 |  | 0.617803 | 0.006609 | 57.75433 | 0.835602 | 0.93545489 |  | 1.1535E-06 | 4.13343E-16 | 3219.051774 | 0.2205 | 0.612908807 |  | 1.696476 | 0.892981 | 3.222947 | 0.109302 | 0.277575493 |
| metabolite843 | 0.244231661 | 0.000819 | 72.81947914 | 0.628689 | 0.765189835 |  | 2.63062 | 0.029585 | 233.9095 | 0.673527 | 0.851700797 |  | 237017.4403 | 0.000104034 | 5.3999E+14 | 0.262688 | 0.648628965 |  | 0.958117 | 0.503991 | 1.821435 | 0.896376 | 0.951229519 |
| metabolite844 | 0.002622379 | 8.48E-06 | 0.811144614 | 0.044589 | 0.124693862 |  | 97.6147 | 1.063964 | 8955.782 | 0.049398 | 0.249369471 |  | 26637.83614 | 6.68183E-06 | 1.06195E+14 | 0.368226 | 0.734276331 |  | 1.933747 | 1.013271 | 3.690403 | 0.047944 | 0.162782704 |
| metabolite845 | 0.000328979 | 1.49E-06 | 0.07263419 | 0.004338 | 0.020613139 |  | 2023.589 | 31.08932 | 131714.4 | 0.000523 | 0.07423496 |  | 96573417146 | 94.29863791 | 9.89031E+19 | 0.018557 | 0.273266792 |  | 3.687514 | 2.060204 | 6.600203 | 2.56E-05 | 0.00128452 |
| metabolite846 | 0.000117365 | 6.01E-07 | 0.022902074 | 0.001057 | 0.007222029 |  | 4.289298 | 0.055316 | 332.6021 | 0.513202 | 0.751575887 |  | 0.073573668 | 5.43271E-11 | 99638707.56 | 0.808266 | 0.9460629 |  | 2.366806 | 1.295535 | 4.323904 | 0.005992 | 0.041099553 |
| metabolite847 | 4.0853E-05 | 1.77E-07 | 0.009437752 | 0.000417 | 0.003505501 |  | 442.4305 | 5.485292 | 35685.39 | 0.007582 | 0.125141666 |  | 18339.29634 | 6.25071E-06 | 5.38066E+13 | 0.379345 | 0.739364696 |  | 3.569321 | 1.951359 | 6.528811 | 7.06E-05 | 0.002358863 |
| metabolite848 | 2.21979E-06 | 1.42E-08 | 0.000346198 | 1.73E-06 | 6.16E-05 |  | 1607.86 | 24.33774 | 106222.5 | 0.000786 | 0.07423496 |  | 90836169.25 | 0.068746135 | 1.20024E+17 | 0.090034 | 0.479980149 |  | 5.276344 | 3.045032 | 9.1427 | 3.49E-08 | 2.59E-05 |
| metabolite849 | 0.000248433 | 8.55E-07 | 0.072195137 | 0.004941 | 0.022863297 |  | 1292.456 | 15.35453 | 108791.4 | 0.001985 | 0.088493313 |  | 500.5275861 | 1.03097E-07 | 2.43001E+12 | 0.586004 | 0.863715961 |  | 3.095181 | 1.651942 | 5.799324 | 0.000613 | 0.00902107 |
| metabolite850 | 3.44836E-05 | 1.42E-07 | 0.008345252 | 0.000376 | 0.003234914 |  | 1135.426 | 14.13989 | 91174.05 | 0.002139 | 0.088493313 |  | 520.6842641 | 1.37972E-07 | 1.96498E+12 | 0.579345 | 0.858262485 |  | 4.099094 | 2.250108 | 7.467452 | 1.08E-05 | 0.000669579 |
| metabolite851 | 0.002660761 | 8.93E-06 | 0.79292917 | 0.043742 | 0.123161789 |  | 996.4824 | 12.4159 | 79976.28 | 0.002563 | 0.089719453 |  | 12250393.65 | 0.004024647 | 3.72883E+16 | 0.14576 | 0.546460871 |  | 2.273079 | 1.203935 | 4.291666 | 0.012727 | 0.067051153 |
| metabolite852 | 3.9168E-05 | 2.02E-07 | 0.007602151 | 0.000258 | 0.002416274 |  | 162.8025 | 2.200652 | 12044 | 0.022214 | 0.17690003 |  | 6536.085803 | 4.06266E-06 | 1.05154E+13 | 0.418384 | 0.770151094 |  | 4.252986 | 2.405291 | 7.520041 | 2.37E-06 | 0.000231136 |
| metabolite853 | 0.00010248 | 3.88E-07 | 0.027059174 | 0.001635 | 0.009946885 |  | 2022.657 | 25.62311 | 159666.1 | 0.000891 | 0.07423496 |  | 1655140.099 | 0.000463265 | 5.91344E+15 | 0.204646 | 0.597241307 |  | 3.343189 | 1.803194 | 6.198396 | 0.000211 | 0.004461706 |
| metabolite854 | 0.022473561 | 4.82E-05 | 10.48087024 | 0.228625 | 0.391341601 |  | 696.4692 | 6.234192 | 77807.89 | 0.007567 | 0.125141666 |  | 125261375.2 | 0.010089547 | 1.55512E+18 | 0.118704 | 0.520210366 |  | 1.779808 | 0.894121 | 3.542827 | 0.103551 | 0.268350463 |
| metabolite855 | 1.356995354 | 0.005253 | 350.5225987 | 0.914406 | 0.955069484 |  | 19.07428 | 0.248837 | 1462.113 | 0.185679 | 0.460976804 |  | 70.87440129 | 4.92353E-08 | 1.02024E+11 | 0.692841 | 0.907533437 |  | 1.134248 | 0.60699 | 2.119507 | 0.69367 | 0.830657876 |
| metabolite856 | 107.9262238 | 0.392217 | 29698.00322 | 0.105211 | 0.231164969 |  | 0.569932 | 0.006492 | 50.03769 | 0.805941 | 0.924527511 |  | 83852.79533 | 3.8822E-05 | 1.81116E+14 | 0.303469 | 0.687752235 |  | 0.44181 | 0.237193 | 0.822942 | 0.011369 | 0.062410909 |
| metabolite857 | 0.129024513 | 0.000421 | 39.55383228 | 0.484761 | 0.644092149 |  | 275.0539 | 3.390684 | 22312.5 | 0.013719 | 0.149599761 |  | 293864867.7 | 0.135663959 | 6.36548E+17 | 0.078165 | 0.45805738 |  | 1.571304 | 0.827836 | 2.98247 | 0.169706 | 0.360490917 |
| metabolite858 | 0.034242699 | 0.000141 | 8.289045784 | 0.23083 | 0.393301393 |  | 316.156 | 4.662026 | 21440.18 | 0.00859 | 0.126652263 |  | 1757422728 | 1.961072692 | 1.57492E+18 | 0.045368 | 0.380219234 |  | 1.739781 | 0.941774 | 3.213977 | 0.079737 | 0.227444684 |
| metabolite859 | 3040.700892 | 11.12057 | 831419.7782 | 0.006004 | 0.026571453 |  | 0.800537 | 0.008278 | 77.42163 | 0.924186 | 0.97342373 |  | 4812.682953 | 1.34011E-06 | 1.72835E+13 | 0.451644 | 0.787987411 |  | 0.52318 | 0.2751 | 0.994971 | 0.050708 | 0.16968257 |
| metabolite860 | 0.109100136 | 0.000454 | 26.23390969 | 0.43003 | 0.598141918 |  | 34.16196 | 0.473242 | 2466.053 | 0.108645 | 0.355281522 |  | 72.19923488 | 6.21657E-08 | 83852132664 | 0.68857 | 0.905974211 |  | 1.4491 | 0.783099 | 2.681516 | 0.239989 | 0.44375831 |
| metabolite861 | 0.190844781 | 0.000682 | 53.37247122 | 0.565615 | 0.713876408 |  | 1.371555 | 0.01614 | 116.5518 | 0.88939 | 0.95849259 |  | 0.001582374 | 8.03038E-13 | 3118043.869 | 0.555991 | 0.844728846 |  | 1.101284 | 0.583456 | 2.078693 | 0.766517 | 0.873631653 |
| metabolite862 | 894808.2356 | 3364.986 | 237945088.1 | 4.76E-06 | 0.000121932 |  | 2.356578 | 0.018811 | 295.2198 | 0.728636 | 0.883101408 |  | 1064221700 | 0.10968176 | 1.03259E+19 | 0.079202 | 0.460912725 |  | 0.37842 | 0.194164 | 0.737529 | 0.005149 | 0.037538436 |
| metabolite863 | 336.6283092 | 1.221074 | 92802.42051 | 0.044782 | 0.124889502 |  | 0.689331 | 0.007614 | 62.40548 | 0.871729 | 0.952026216 |  | 0.189653119 | 6.86791E-11 | 523715103.4 | 0.881118 | 0.965877673 |  | 0.774397 | 0.407178 | 1.472796 | 0.437321 | 0.637923084 |
| metabolite864 | 790941.9853 | 2559.643 | 244404863.5 | 9.49E-06 | 0.000204698 |  | 2.818548 | 0.020371 | 389.978 | 0.68115 | 0.856573852 |  | 2190207897 | 0.141218836 | 3.39686E+19 | 0.075136 | 0.452993909 |  | 0.378303 | 0.191239 | 0.748344 | 0.006153 | 0.041708357 |
| metabolite865 | 0.105842298 | 0.000441 | 25.38269876 | 0.42354 | 0.591998283 |  | 92.20119 | 1.321768 | 6431.584 | 0.039011 | 0.223736893 |  | 299423.3095 | 0.00029229 | 3.06731E+14 | 0.236105 | 0.6276411 |  | 1.323498 | 0.714218 | 2.452537 | 0.375084 | 0.590086889 |
| metabolite866 | 962.0383048 | 3.36112 | 275359.8796 | 0.019016 | 0.065340901 |  | 8.774083 | 0.092834 | 829.2727 | 0.351406 | 0.625845113 |  | 46624785196 | 20.32303207 | 1.06966E+20 | 0.027495 | 0.304618594 |  | 0.772572 | 0.402725 | 1.482069 | 0.439219 | 0.637942526 |
| metabolite867 | 78342.33828 | 235.5365 | 26057624.93 | 0.000234 | 0.002224318 |  | 2.05842 | 0.015959 | 265.5023 | 0.771464 | 0.906842578 |  | 3625925574 | 0.338253695 | 3.88683E+19 | 0.064398 | 0.426996029 |  | 0.428593 | 0.217715 | 0.843726 | 0.015776 | 0.077337991 |
| metabolite868 | 15557494.16 | 47324.95 | 5114334276 | 1.57E-07 | 1.16E-05 |  | 0.416092 | 0.002376 | 72.87681 | 0.739985 | 0.890429375 |  | 3580144.668 | 6.21722E-05 | 2.0616E+17 | 0.235102 | 0.626248163 |  | 0.268805 | 0.13379 | 0.540071 | 0.000348 | 0.006039676 |
| metabolite869 | 18.34926683 | 0.075537 | 4457.337496 | 0.301412 | 0.473632948 |  | 69.38466 | 0.966245 | 4982.414 | 0.054396 | 0.25747688 |  | 23379.59408 | 1.98812E-05 | 2.74936E+13 | 0.347194 | 0.717791422 |  | 0.753931 | 0.405787 | 1.400765 | 0.373427 | 0.588944766 |
| metabolite870 | 8.33429911 | 0.034578 | 2008.824761 | 0.450234 | 0.616083905 |  | 8.918136 | 0.119675 | 664.5772 | 0.321997 | 0.599401439 |  | 7074.879883 | 6.37785E-06 | 7.84809E+12 | 0.405955 | 0.762519149 |  | 0.745516 | 0.40225 | 1.381715 | 0.35289 | 0.563501012 |
| metabolite871 | 1442652.992 | 2021.555 | 1029528227 | 4.81E-05 | 0.000700588 |  | 1.728228 | 0.006556 | 455.5584 | 0.84781 | 0.940012856 |  | 6.781985466 | 1.41278E-11 | 3.25567E+12 | 0.889313 | 0.967390372 |  | 0.372616 | 0.171478 | 0.809681 | 0.014136 | 0.072259677 |
| metabolite872 | 0.868914313 | 0.003706 | 203.7239548 | 0.959843 | 0.980990323 |  | 8.770028 | 0.121603 | 632.4961 | 0.322027 | 0.599401439 |  | 1354829.332 | 0.001584792 | 1.15824E+15 | 0.181184 | 0.579133757 |  | 0.788394 | 0.42704 | 1.455522 | 0.448835 | 0.646343772 |
| metabolite873 | 8.801477204 | 0.032573 | 2378.217549 | 0.448073 | 0.614258952 |  | 0.053185 | 0.000662 | 4.274859 | 0.192595 | 0.470023308 |  | 0.253231181 | 1.38543E-10 | 462860673.5 | 0.899781 | 0.968957237 |  | 0.47626 | 0.256947 | 0.882766 | 0.020225 | 0.090753708 |
| metabolite874 | 1154206.541 | 3715.392 | 358560510.7 | 5.70E-06 | 0.000140928 |  | 2.64963 | 0.018643 | 376.5874 | 0.700747 | 0.869142035 |  | 12868736363 | 0.772873891 | 2.14271E+20 | 0.055096 | 0.402327874 |  | 0.347073 | 0.175543 | 0.686209 | 0.002926 | 0.025978411 |
| metabolite875 | 8.63842E-05 | 1.08E-07 | 0.069173408 | 0.007098 | 0.030593889 |  | 21.53403 | 0.096294 | 4815.599 | 0.268495 | 0.555007395 |  | 0.001758161 | 7.20809E-15 | 428841379 | 0.636298 | 0.882069794 |  | 2.910912 | 1.371785 | 6.176923 | 0.006322 | 0.042349926 |
| metabolite876 | 462091.9257 | 561.0136 | 380612764.4 | 0.00023 | 0.002201148 |  | 4.014303 | 0.014616 | 1102.527 | 0.628554 | 0.825686883 |  | 541.4695179 | 9.24012E-10 | 3.173E+14 | 0.64979 | 0.890461807 |  | 0.406275 | 0.185039 | 0.892027 | 0.026766 | 0.112348828 |
| metabolite877 | 942222.0048 | 2346.365 | 378364999.2 | 1.70E-05 | 0.000312755 |  | 4.175165 | 0.024796 | 703.0243 | 0.585866 | 0.797266959 |  | 5.59768E+11 | 16.47078973 | 1.9024E+22 | 0.030882 | 0.323112034 |  | 0.365582 | 0.179736 | 0.743593 | 0.006426 | 0.042912683 |
| metabolite878 | 0.352786045 | 0.001655 | 75.22206222 | 0.704061 | 0.82291995 |  | 13.31684 | 0.20014 | 886.0719 | 0.229287 | 0.511407568 |  | 30779.07242 | 4.72307E-05 | 2.00579E+13 | 0.320418 | 0.700344699 |  | 0.957155 | 0.523016 | 1.75166 | 0.887325 | 0.945681144 |
| metabolite879 | 3311.951674 | 13.13937 | 834820.8607 | 0.004874 | 0.022636408 |  | 14.65492 | 0.165164 | 1300.321 | 0.243257 | 0.526615886 |  | 51258.52684 | 1.96557E-05 | 1.33673E+14 | 0.329051 | 0.705196155 |  | 0.609375 | 0.321642 | 1.154508 | 0.131529 | 0.308440007 |
| metabolite880 | 5.878897195 | 0.033045 | 1045.878388 | 0.504194 | 0.659863196 |  | 0.005815 | 0.000109 | 0.30948 | 0.012523 | 0.143429015 |  | 0.002738322 | 7.66609E-12 | 978126.3561 | 0.558241 | 0.846255759 |  | 0.555641 | 0.312823 | 0.986939 | 0.047412 | 0.16127195 |
| metabolite881 | 68821.04417 | 123.8768 | 38234252.19 | 0.000781 | 0.005708758 |  | 33.20985 | 0.183819 | 5999.881 | 0.189167 | 0.465515387 |  | 5.70362E+16 | 1714481.49 | 1.89744E+27 | 0.002296 | 0.098476966 |  | 0.486493 | 0.23279 | 1.016691 | 0.05794 | 0.184531753 |
| metabolite882 | 798500.8189 | 1963.222 | 324773972.1 | 2.19E-05 | 0.000377865 |  | 4.69147 | 0.0279 | 788.8792 | 0.555609 | 0.783086139 |  | 4.74896E+11 | 13.89896969 | 1.62261E+22 | 0.031931 | 0.324646228 |  | 0.361612 | 0.177885 | 0.7351 | 0.005853 | 0.040827272 |
| metabolite883 | 13803.65625 | 22.27576 | 8553733.197 | 0.004418 | 0.020887103 |  | 38.83722 | 0.212669 | 7092.395 | 0.171177 | 0.442862885 |  | 9.56945E+14 | 21652.16977 | 4.22934E+25 | 0.006798 | 0.173978439 |  | 0.547733 | 0.260591 | 1.151272 | 0.115059 | 0.28695269 |
| metabolite884 | 578621.7476 | 670.617 | 499246371 | 0.0002 | 0.002017622 |  | 0.374636 | 0.001299 | 108.0199 | 0.734694 | 0.887227321 |  | 0.000131699 | 1.84965E-16 | 93772700.15 | 0.522398 | 0.824510163 |  | 0.327441 | 0.149564 | 0.716868 | 0.006159 | 0.041708357 |
| metabolite885 | 0.118915072 | 0.000352 | 40.16887111 | 0.475004 | 0.637518519 |  | 5.85026 | 0.059773 | 572.5939 | 0.451636 | 0.703347616 |  | 2274871950 | 0.770070687 | 6.72022E+18 | 0.055347 | 0.402327874 |  | 1.717954 | 0.897208 | 3.2895 | 0.10536 | 0.271004899 |
| metabolite886 | 250.0211673 | 0.32236 | 193915.3312 | 0.106676 | 0.23341641 |  | 333.3979 | 1.857474 | 59841.55 | 0.030331 | 0.202081312 |  | 1.41403E+20 | 4940003350 | 4.04751E+30 | 0.000257 | 0.047140629 |  | 0.786389 | 0.368864 | 1.676523 | 0.535109 | 0.710007114 |
| metabolite887 | 0.136576602 | 0.000474 | 39.31691419 | 0.492192 | 0.650703098 |  | 131.7058 | 1.657527 | 10465.24 | 0.030888 | 0.203594474 |  | 4.32503E+14 | 476057.0938 | 3.92933E+23 | 0.00178 | 0.082547774 |  | 1.552327 | 0.823511 | 2.92615 | 0.1767 | 0.369635162 |
| metabolite888 | 0.072269012 | 0.000249 | 20.96607263 | 0.36575 | 0.536607347 |  | 2.960547 | 0.033653 | 260.4498 | 0.635603 | 0.828784839 |  | 27054.26014 | 1.19681E-05 | 6.11569E+13 | 0.355064 | 0.720858157 |  | 1.318274 | 0.695809 | 2.497591 | 0.398506 | 0.610341437 |
| metabolite889 | 0.47912807 | 0.001265 | 181.4605112 | 0.80852 | 0.889803214 |  | 12.40947 | 0.118341 | 1301.279 | 0.291019 | 0.574315439 |  | 130794.1918 | 2.31594E-05 | 7.38668E+14 | 0.306012 | 0.68936977 |  | 1.018933 | 0.521975 | 1.989034 | 0.956269 | 0.978889882 |
| metabolite890 | 3842.892869 | 5.742815 | 2571530.804 | 0.014386 | 0.052911213 |  | 99.61456 | 0.552927 | 17946.43 | 0.085268 | 0.318982096 |  | 3.42839E+16 | 877031.8472 | 1.34018E+27 | 0.002778 | 0.107866667 |  | 0.61203 | 0.289851 | 1.29232 | 0.200591 | 0.398412495 |
| metabolite891 | 77321.73132 | 150.1838 | 39808899.94 | 0.000599 | 0.004624615 |  | 17.08834 | 0.098226 | 2972.849 | 0.2832 | 0.570754969 |  | 2.20038E+13 | 580.335823 | 8.3429E+23 | 0.014952 | 0.253980523 |  | 0.460507 | 0.222446 | 0.953341 | 0.03902 | 0.142244383 |
| metabolite892 | 593.9325559 | 1.220453 | 289036.8201 | 0.045465 | 0.126465235 |  | 87.86589 | 0.660213 | 11693.83 | 0.075597 | 0.302959698 |  | 3.10231E+13 | 2531.774689 | 3.80142E+23 | 0.009989 | 0.205946835 |  | 0.833673 | 0.410325 | 1.693804 | 0.615981 | 0.774675165 |
| metabolite893 | 496.9003417 | 1.681014 | 146881.5324 | 0.034632 | 0.102487179 |  | 2.770507 | 0.028776 | 266.7367 | 0.662736 | 0.84717161 |  | 128535.2789 | 3.78547E-05 | 4.3644E+14 | 0.295699 | 0.67904734 |  | 0.838666 | 0.436484 | 1.611425 | 0.598521 | 0.762482023 |
| metabolite894 | 0.130972045 | 0.00049 | 34.98213606 | 0.477313 | 0.639086946 |  | 336.3488 | 4.678757 | 24179.6 | 0.008787 | 0.126652263 |  | 16309.43127 | 1.01212E-05 | 2.62811E+13 | 0.371804 | 0.736141064 |  | 1.221892 | 0.650948 | 2.293607 | 0.534079 | 0.709619184 |
| metabolite895 | 0.023011238 | 6.87E-05 | 7.706911746 | 0.20619 | 0.366652379 |  | 168.0322 | 1.844881 | 15304.42 | 0.028031 | 0.194800441 |  | 24063337.67 | 0.006562292 | 8.82381E+16 | 0.13321 | 0.532435499 |  | 1.621933 | 0.843795 | 3.117661 | 0.149721 | 0.333698345 |
| metabolite896 | 20.43221739 | 0.045039 | 9269.204163 | 0.335803 | 0.509678861 |  | 1.433681 | 0.011372 | 180.7458 | 0.884202 | 0.957198024 |  | 6.279317415 | 4.60421E-10 | 85638660988 | 0.877644 | 0.96528138 |  | 0.635123 | 0.31959 | 1.262183 | 0.197839 | 0.395043799 |
| metabolite897 | 448.3563935 | 0.597949 | 336188.3131 | 0.073355 | 0.178271164 |  | 359.3948 | 2.034009 | 63502.46 | 0.027832 | 0.194420599 |  | 2.05813E+20 | 7861887936 | 5.3879E+30 | 0.000219 | 0.047140629 |  | 0.74529 | 0.350443 | 1.585015 | 0.446717 | 0.644543633 |
| metabolite898 | 0.137189879 | 0.000565 | 33.31556942 | 0.479901 | 0.639626783 |  | 31.2188 | 0.429038 | 2271.625 | 0.118533 | 0.371517626 |  | 15636910940 | 20.48903672 | 1.19338E+19 | 0.02646 | 0.300748226 |  | 1.253292 | 0.675119 | 2.326614 | 0.475923 | 0.668489373 |
| metabolite899 | 45.0936911 | 0.115092 | 17667.94169 | 0.213826 | 0.375657426 |  | 0.266848 | 0.002359 | 30.1803 | 0.58505 | 0.7970792 |  | 7.129928389 | 8.58274E-10 | 59230330902 | 0.866448 | 0.962114722 |  | 0.715975 | 0.364759 | 1.405367 | 0.333656 | 0.546666032 |
| metabolite900 | 1343.087689 | 1.918549 | 940233.8402 | 0.033328 | 0.099580033 |  | 465.0941 | 2.724362 | 79399.35 | 0.020955 | 0.172041135 |  | 7.40696E+19 | 2980320050 | 1.84085E+30 | 0.000286 | 0.047140629 |  | 0.688867 | 0.325397 | 1.458335 | 0.332172 | 0.54519747 |
| metabolite901 | 9888.157048 | 18.30127 | 5342561.457 | 0.004981 | 0.022933756 |  | 37.5733 | 0.231333 | 6102.69 | 0.165404 | 0.434098523 |  | 8.22932E+14 | 33139.96478 | 2.0435E+25 | 0.005818 | 0.163554624 |  | 0.562651 | 0.272054 | 1.163651 | 0.123702 | 0.29925505 |
| metabolite902 | 309.8309294 | 0.868331 | 110551.3782 | 0.058335 | 0.150543962 |  | 0.286361 | 0.00261 | 31.41837 | 0.602905 | 0.808892614 |  | 85.11502667 | 1.20439E-08 | 6.01513E+11 | 0.70166 | 0.910122668 |  | 0.596157 | 0.306307 | 1.160283 | 0.130744 | 0.307862507 |
| metabolite903 | 0.003254317 | 3.94E-06 | 2.688570364 | 0.09746 | 0.219461414 |  | 377.0829 | 1.998055 | 71164.95 | 0.028529 | 0.196418701 |  | 527.0834209 | 3.25243E-09 | 8.54184E+13 | 0.635068 | 0.882069794 |  | 1.923533 | 0.902896 | 4.097901 | 0.092826 | 0.25162578 |
| metabolite904 | 0.013885956 | 5.59E-05 | 3.452284034 | 0.131423 | 0.270800208 |  | 267.9238 | 3.770819 | 19036.49 | 0.011489 | 0.139886855 |  | 586367602.2 | 0.523242284 | 6.57109E+17 | 0.060151 | 0.415543963 |  | 1.688804 | 0.908367 | 3.139765 | 0.100497 | 0.262933884 |
| metabolite905 | 0.008974753 | 2.24E-05 | 3.602986971 | 0.126175 | 0.263054301 |  | 47.9744 | 0.429245 | 5361.844 | 0.110558 | 0.357150654 |  | 168.5817663 | 1.73139E-08 | 1.64144E+12 | 0.662988 | 0.897937999 |  | 1.92729 | 0.984763 | 3.771921 | 0.058043 | 0.184531753 |
| metabolite906 | 182119.1808 | 885.6957 | 37447844.69 | 1.99E-05 | 0.000345507 |  | 1.914212 | 0.020182 | 181.5596 | 0.780335 | 0.911495653 |  | 33.12837016 | 9.60357E-09 | 1.14279E+11 | 0.755323 | 0.937628791 |  | 0.441892 | 0.234539 | 0.832562 | 0.012913 | 0.067586181 |
| metabolite907 | 3374.947626 | 5.565156 | 2046711.875 | 0.014445 | 0.05302088 |  | 94.93121 | 0.570475 | 15797.25 | 0.08377 | 0.315284692 |  | 3.77623E+16 | 1450503.228 | 9.831E+26 | 0.002309 | 0.098476966 |  | 0.605861 | 0.290317 | 1.264368 | 0.184592 | 0.38014775 |
| metabolite908 | 26.60288421 | 0.076053 | 9305.562155 | 0.274623 | 0.445024204 |  | 5.563047 | 0.054466 | 568.1955 | 0.468715 | 0.717917212 |  | 9762340.887 | 0.002291659 | 4.1587E+16 | 0.157634 | 0.562481493 |  | 0.850173 | 0.438247 | 1.649285 | 0.632105 | 0.784791292 |
| metabolite909 | 0.05631753 | 0.000213 | 14.8730367 | 0.314148 | 0.487783267 |  | 23.73619 | 0.299785 | 1879.367 | 0.158442 | 0.425238556 |  | 0.919457912 | 5.25429E-10 | 1608977124 | 0.993844 | 0.99760733 |  | 1.560217 | 0.834664 | 2.916474 | 0.16618 | 0.356676119 |
| metabolite910 | 25802.29123 | 63.61854 | 10464846.62 | 0.001237 | 0.008181818 |  | 6.108567 | 0.043441 | 858.9623 | 0.474794 | 0.721241882 |  | 25574936.83 | 0.001294198 | 5.05392E+17 | 0.161272 | 0.564842819 |  | 0.464594 | 0.231947 | 0.930588 | 0.032688 | 0.128983989 |
| metabolite911 | 228.5783651 | 0.4589 | 113854.9007 | 0.089285 | 0.206698173 |  | 2.229171 | 0.015747 | 315.5661 | 0.751659 | 0.895049124 |  | 2912.306744 | 1.2643E-07 | 6.70849E+13 | 0.513666 | 0.819395994 |  | 0.779449 | 0.38426 | 1.581064 | 0.491319 | 0.680583595 |
| metabolite912 | 67.80281366 | 0.093491 | 49173.12693 | 0.212198 | 0.373737849 |  | 470.2633 | 2.878042 | 76839.61 | 0.019687 | 0.165250229 |  | 1.81882E+20 | 9658499948 | 3.42509E+30 | 0.000188 | 0.047140629 |  | 0.866439 | 0.410642 | 1.828153 | 0.707391 | 0.837715271 |
| metabolite913 | 71590.58834 | 268.9255 | 19058115.52 | 0.000152 | 0.001643043 |  | 1.699011 | 0.015588 | 185.1867 | 0.825149 | 0.931343691 |  | 1208.716554 | 1.84905E-07 | 7.90132E+12 | 0.539485 | 0.832371017 |  | 0.415628 | 0.216751 | 0.796981 | 0.009401 | 0.054940412 |
| metabolite914 | 2199435.311 | 8237.173 | 587278660.5 | 1.28E-06 | 5.05E-05 |  | 1.364048 | 0.01024 | 181.7013 | 0.901233 | 0.963270517 |  | 11223317037 | 0.935645205 | 1.34627E+20 | 0.053171 | 0.394365301 |  | 0.311756 | 0.160268 | 0.606432 | 0.000839 | 0.011446015 |
| metabolite915 | 7907.65815 | 35.65805 | 1753630.887 | 0.001495 | 0.009369707 |  | 3.749902 | 0.044001 | 319.5791 | 0.561228 | 0.78648406 |  | 3400592.678 | 0.001916004 | 6.03549E+15 | 0.169102 | 0.568636202 |  | 0.603935 | 0.321684 | 1.133839 | 0.119459 | 0.293389811 |
| metabolite916 | 307.5179225 | 1.02918 | 91886.05293 | 0.051342 | 0.137765358 |  | 3.145386 | 0.032872 | 300.9668 | 0.623383 | 0.822537502 |  | 99.20154731 | 2.73078E-08 | 3.60371E+11 | 0.683095 | 0.903557902 |  | 0.744842 | 0.388513 | 1.427982 | 0.376928 | 0.591200447 |
| metabolite917 | 297.6367643 | 0.403785 | 219393.025 | 0.093681 | 0.213808145 |  | 281.2694 | 1.614527 | 49000.42 | 0.034383 | 0.212415725 |  | 2.04651E+20 | 8798788086 | 4.75998E+30 | 0.000205 | 0.047140629 |  | 0.725095 | 0.342212 | 1.536365 | 0.403223 | 0.613011721 |
| metabolite918 | 1161.018799 | 3.884561 | 347005.6662 | 0.01685 | 0.059609832 |  | 0.007676 | 8.39E-05 | 0.702132 | 0.0368 | 0.2199105 |  | 0.004551609 | 1.03955E-12 | 19928911.37 | 0.634956 | 0.882069794 |  | 0.44469 | 0.234158 | 0.844514 | 0.014779 | 0.074769919 |
| metabolite919 | 326.8481815 | 1.193825 | 89485.28434 | 0.045595 | 0.126649 |  | 2.929441 | 0.032686 | 262.545 | 0.640276 | 0.831955879 |  | 1772078.336 | 0.000778454 | 4.03397E+15 | 0.193296 | 0.592990803 |  | 0.759473 | 0.399796 | 1.442735 | 0.402495 | 0.61293609 |
| metabolite920 | 98.95069368 | 0.341574 | 28665.08637 | 0.114996 | 0.246391333 |  | 4.635454 | 0.051222 | 419.4956 | 0.506005 | 0.744266611 |  | 10933.63923 | 4.06943E-06 | 2.93763E+13 | 0.402985 | 0.761444693 |  | 0.879877 | 0.461494 | 1.677562 | 0.698251 | 0.831848922 |
| metabolite921 | 0.7443954 | 0.001592 | 348.011705 | 0.925188 | 0.960689096 |  | 246.7547 | 2.175252 | 27991.17 | 0.024399 | 0.185877348 |  | 4.74381E+13 | 7192.96681 | 3.12857E+23 | 0.00737 | 0.178734331 |  | 1.543988 | 0.776178 | 3.07133 | 0.218361 | 0.415345611 |
| metabolite922 | 75.29639202 | 0.123213 | 46014.36137 | 0.189455 | 0.34501122 |  | 349.5264 | 2.418939 | 50505.08 | 0.022845 | 0.178478733 |  | 7.48683E+18 | 632262709.1 | 8.86541E+28 | 0.000371 | 0.047140629 |  | 0.785068 | 0.379515 | 1.623998 | 0.515424 | 0.697316729 |
| metabolite923 | 0.432904831 | 0.001551 | 120.8611003 | 0.771309 | 0.867104274 |  | 18.24963 | 0.223279 | 1491.627 | 0.198819 | 0.477435637 |  | 173.4819804 | 8.96583E-08 | 3.35674E+11 | 0.637422 | 0.882670671 |  | 1.277037 | 0.678112 | 2.404946 | 0.450527 | 0.647979801 |
| metabolite924 | 0.041291978 | 0.000166 | 10.26724876 | 0.259882 | 0.426735931 |  | 51.20625 | 0.690079 | 3799.684 | 0.075989 | 0.303547572 |  | 7841.045332 | 5.87943E-06 | 1.04571E+13 | 0.404678 | 0.762519149 |  | 1.775034 | 0.958964 | 3.285571 | 0.070445 | 0.210145556 |
| metabolite925 | 242643.4884 | 385.4543 | 152744082.1 | 0.000263 | 0.002443459 |  | 69.54494 | 0.336134 | 14388.59 | 0.121784 | 0.375256628 |  | 2.66125E+17 | 4034023.834 | 1.75563E+28 | 0.002055 | 0.090782442 |  | 0.400257 | 0.18869 | 0.849041 | 0.0187 | 0.086636785 |
| metabolite926 | 0.338117669 | 0.001254 | 91.14052182 | 0.70486 | 0.823593842 |  | 116.194 | 1.544792 | 8739.72 | 0.033139 | 0.210580718 |  | 1190.074856 | 7.12523E-07 | 1.9877E+12 | 0.514713 | 0.819395994 |  | 1.600831 | 0.857097 | 2.989927 | 0.14272 | 0.324660504 |
| metabolite927 | 34.67075875 | 0.140552 | 8552.425327 | 0.209674 | 0.371054419 |  | 10.55129 | 0.136694 | 814.4438 | 0.290279 | 0.573923383 |  | 179513.3701 | 0.000142337 | 2.26399E+14 | 0.26026 | 0.647529078 |  | 0.711553 | 0.382146 | 1.324907 | 0.285619 | 0.498088693 |
| metabolite928 | 289.9972603 | 1.477325 | 56926.13509 | 0.037559 | 0.109147853 |  | 19.73697 | 0.295044 | 1320.306 | 0.167069 | 0.436923648 |  | 1850113.561 | 0.00288822 | 1.18513E+15 | 0.165851 | 0.568636202 |  | 0.965115 | 0.52628 | 1.769872 | 0.908839 | 0.957528306 |
| metabolite929 | 0.042156444 | 0.000118 | 15.09232322 | 0.293554 | 0.464950396 |  | 129.6092 | 1.349275 | 12450.05 | 0.039028 | 0.223736893 |  | 60925.13957 | 1.1863E-05 | 3.12894E+14 | 0.336259 | 0.711435121 |  | 1.71409 | 0.887607 | 3.310142 | 0.111354 | 0.281406417 |
| metabolite930 | 0.085030875 | 0.000242 | 29.88633198 | 0.41166 | 0.579883308 |  | 265.6766 | 2.915444 | 24210.39 | 0.016931 | 0.16038797 |  | 3520791.427 | 0.000834577 | 1.4853E+16 | 0.185225 | 0.58220839 |  | 1.600886 | 0.830306 | 3.086617 | 0.162866 | 0.351962213 |
| metabolite931 | 0.015253528 | 4.74E-05 | 4.903969706 | 0.158364 | 0.30752882 |  | 662.107 | 7.928149 | 55294.83 | 0.004815 | 0.109577358 |  | 33518320.19 | 0.01038392 | 1.08194E+17 | 0.123718 | 0.527043366 |  | 1.676927 | 0.876252 | 3.209217 | 0.12135 | 0.296463574 |
| metabolite932 | 0.052202393 | 0.000196 | 13.92550727 | 0.30248 | 0.474409897 |  | 8.940475 | 0.109619 | 729.1839 | 0.331428 | 0.607338753 |  | 336.6273554 | 1.89371E-07 | 5.98392E+11 | 0.593381 | 0.867253231 |  | 1.356352 | 0.722574 | 2.546022 | 0.344852 | 0.557381312 |
| metabolite933 | 0.001653873 | 7.33E-06 | 0.373127286 | 0.022362 | 0.074094381 |  | 47.54346 | 0.639012 | 3537.305 | 0.081792 | 0.31227223 |  | 2807.169194 | 2.07165E-06 | 3.80383E+12 | 0.460799 | 0.792880411 |  | 1.66315 | 0.8967 | 3.08472 | 0.109355 | 0.277575493 |
| metabolite934 | 25.6475669 | 0.047315 | 13902.62498 | 0.314633 | 0.488032073 |  | 370.4302 | 2.879845 | 47647.88 | 0.018685 | 0.164700188 |  | 8.55443E+18 | 1287379606 | 5.68428E+28 | 0.000256 | 0.047140629 |  | 0.892917 | 0.438097 | 1.819917 | 0.755804 | 0.86674608 |
| metabolite935 | 383.9512801 | 0.53413 | 275997.6435 | 0.07895 | 0.187902892 |  | 187.5513 | 1.075436 | 32708.15 | 0.049316 | 0.249369471 |  | 1.49421E+19 | 579415058.7 | 3.85331E+29 | 0.000461 | 0.047571942 |  | 0.653031 | 0.309357 | 1.378505 | 0.266024 | 0.474394334 |
| metabolite936 | 0.14010828 | 0.00064 | 30.69022242 | 0.476255 | 0.638736568 |  | 63.88121 | 0.974718 | 4186.656 | 0.05394 | 0.257328813 |  | 0.000593795 | 7.60775E-13 | 463464.2629 | 0.478493 | 0.79982887 |  | 1.574224 | 0.861743 | 2.875776 | 0.142777 | 0.324660504 |
| metabolite937 | 31065.08789 | 141.0367 | 6842472.503 | 0.000275 | 0.00253041 |  | 6.10938 | 0.068025 | 548.6895 | 0.43198 | 0.693372042 |  | 173163.6983 | 6.88987E-05 | 4.35214E+14 | 0.27709 | 0.660932141 |  | 0.538217 | 0.285278 | 1.01542 | 0.058357 | 0.185095191 |
| metabolite938 | 13.12439123 | 0.025033 | 6880.892345 | 0.422079 | 0.590847096 |  | 10.99034 | 0.079798 | 1513.659 | 0.342213 | 0.617782379 |  | 492352180.5 | 0.028633039 | 8.46612E+18 | 0.098833 | 0.492678215 |  | 0.890347 | 0.439011 | 1.805687 | 0.748099 | 0.862227934 |
| metabolite939 | 0.004556406 | 2.6E-05 | 0.798508369 | 0.043183 | 0.121865546 |  | 36.6337 | 0.613489 | 2187.535 | 0.08716 | 0.321695362 |  | 3594.187368 | 7.89902E-06 | 1.63542E+12 | 0.422589 | 0.77189627 |  | 2.05791 | 1.15524 | 3.665899 | 0.015853 | 0.0775115 |
| metabolite940 | 0.094841588 | 0.000342 | 26.28920606 | 0.413508 | 0.581480889 |  | 0.121839 | 0.001459 | 10.17794 | 0.353179 | 0.627092503 |  | 18.27454636 | 9.03796E-09 | 36950691678 | 0.790907 | 0.944051932 |  | 0.995037 | 0.527049 | 1.87857 | 0.987784 | 0.993405964 |
| metabolite941 | 3.805185171 | 0.013135 | 1102.363544 | 0.644952 | 0.779360559 |  | 41.79603 | 0.506055 | 3452.008 | 0.100231 | 0.343190353 |  | 0.014317236 | 6.30538E-12 | 32509276.97 | 0.699995 | 0.909170666 |  | 1.196379 | 0.631926 | 2.265014 | 0.583027 | 0.750916404 |
| metabolite942 | 943.2187538 | 3.865708 | 230141.9503 | 0.01618 | 0.057965884 |  | 0.181391 | 0.002157 | 15.25667 | 0.451893 | 0.703347616 |  | 41.81279532 | 2.06807E-08 | 84538259524 | 0.733385 | 0.926341113 |  | 0.554003 | 0.296226 | 1.036099 | 0.067123 | 0.20317441 |
| metabolite943 | 0.06287955 | 0.000183 | 21.61439369 | 0.355157 | 0.527561507 |  | 3.168974 | 0.031492 | 318.8895 | 0.624937 | 0.823083204 |  | 17725367.14 | 0.00467774 | 6.71668E+16 | 0.140846 | 0.542758378 |  | 1.325952 | 0.686509 | 2.560999 | 0.402686 | 0.61293609 |
| metabolite944 | 0.000165269 | 6.42E-07 | 0.042553032 | 0.002653 | 0.014322114 |  | 224.9834 | 2.652502 | 19082.93 | 0.018505 | 0.164700188 |  | 25.95876648 | 7.53289E-09 | 89455401185 | 0.771863 | 0.940047404 |  | 2.395751 | 1.275022 | 4.501589 | 0.007687 | 0.048598438 |
| metabolite945 | 3473.076337 | 9.152001 | 1317991.52 | 0.008233 | 0.033968221 |  | 32.80152 | 0.274652 | 3917.459 | 0.155404 | 0.421465998 |  | 1.4439E+14 | 24842.99964 | 8.39209E+23 | 0.005332 | 0.15839396 |  | 0.539713 | 0.273203 | 1.066201 | 0.078563 | 0.225348567 |
| metabolite946 | 0.018498066 | 6.32E-05 | 5.412181545 | 0.171232 | 0.323214787 |  | 8.88929 | 0.099605 | 793.3285 | 0.342436 | 0.617782379 |  | 1155.740571 | 4.27146E-07 | 3.12712E+12 | 0.525791 | 0.824919067 |  | 1.153159 | 0.605291 | 2.196917 | 0.665608 | 0.810232906 |
| metabolite947 | 0.011811123 | 2.18E-05 | 6.409010246 | 0.169833 | 0.322287716 |  | 298.0594 | 2.248721 | 39506.64 | 0.024212 | 0.185877348 |  | 5809027.872 | 0.000230175 | 1.46605E+17 | 0.20514 | 0.597241307 |  | 1.769683 | 0.87234 | 3.590087 | 0.116592 | 0.288640321 |
| metabolite948 | 236.7535522 | 1.052432 | 53259.73418 | 0.05035 | 0.135594699 |  | 9.521586 | 0.126764 | 715.1922 | 0.308679 | 0.587722023 |  | 37053719.29 | 0.038565996 | 3.56007E+16 | 0.101461 | 0.49665678 |  | 0.812668 | 0.437384 | 1.509954 | 0.513008 | 0.697052269 |
| metabolite949 | 4562.154459 | 6.824709 | 3049690.856 | 0.012513 | 0.047290732 |  | 138.0777 | 0.770462 | 24745.47 | 0.06532 | 0.280557454 |  | 2.78657E+17 | 7808750.412 | 9.94394E+27 | 0.001577 | 0.078006156 |  | 0.549538 | 0.260775 | 1.158057 | 0.118298 | 0.292277533 |
| metabolite950 | 17.83551793 | 0.049636 | 6408.829381 | 0.339289 | 0.513081896 |  | 5.312601 | 0.051189 | 551.3683 | 0.482217 | 0.726556062 |  | 835260.901 | 0.000171883 | 4.05893E+15 | 0.233379 | 0.625237811 |  | 0.93515 | 0.480717 | 1.819169 | 0.843806 | 0.923161255 |
| metabolite951 | 1471.262353 | 2.11085 | 1025469.57 | 0.031091 | 0.094729093 |  | 91.44626 | 0.505807 | 16532.81 | 0.091374 | 0.328579908 |  | 9.68852E+17 | 29809861.87 | 3.14887E+28 | 0.001092 | 0.066418156 |  | 0.58497 | 0.277298 | 1.234014 | 0.161959 | 0.350249737 |
| metabolite952 | 0.015881987 | 4.59E-05 | 5.499294484 | 0.167733 | 0.318881879 |  | 18.35333 | 0.182191 | 1848.855 | 0.218894 | 0.500194123 |  | 88.98606783 | 1.67857E-08 | 4.71742E+11 | 0.69515 | 0.907533437 |  | 1.580274 | 0.817709 | 3.053977 | 0.176174 | 0.369159628 |
| metabolite953 | 118.0684644 | 0.091282 | 152715.4692 | 0.194532 | 0.351464148 |  | 2.067016 | 0.00703 | 607.7327 | 0.802743 | 0.922563913 |  | 2227958924 | 0.003655845 | 1.35777E+21 | 0.122867 | 0.526740039 |  | 1.081042 | 0.479415 | 2.437661 | 0.851347 | 0.926494842 |
| metabolite954 | 11.54223645 | 0.031348 | 4249.867673 | 0.41888 | 0.587476735 |  | 18.5811 | 0.180398 | 1913.862 | 0.219158 | 0.500219786 |  | 573994470.4 | 0.13112533 | 2.51263E+18 | 0.077708 | 0.45805738 |  | 0.963358 | 0.494201 | 1.877898 | 0.912912 | 0.958092754 |
| metabolite955 | 7500.587999 | 9.389445 | 5991708.835 | 0.010112 | 0.039962816 |  | 31.43474 | 0.144203 | 6852.463 | 0.212086 | 0.490285108 |  | 1.11142E+17 | 1365572.247 | 9.04574E+27 | 0.002757 | 0.107866667 |  | 0.491852 | 0.229025 | 1.056299 | 0.071523 | 0.211936939 |
| metabolite956 | 2686.665389 | 4.846171 | 1489458.491 | 0.015864 | 0.057102849 |  | 18.15046 | 0.112992 | 2915.602 | 0.265732 | 0.553074754 |  | 1.43123E+14 | 6058.993853 | 3.38081E+24 | 0.008611 | 0.188291096 |  | 0.531537 | 0.258441 | 1.093214 | 0.088629 | 0.245448351 |
| metabolite957 | 0.457783665 | 0.001469 | 142.6902726 | 0.790187 | 0.877696133 |  | 121.7711 | 1.445212 | 10260.23 | 0.035994 | 0.217903576 |  | 70.91496655 | 2.39748E-08 | 2.09759E+11 | 0.702448 | 0.910825601 |  | 1.303468 | 0.683806 | 2.484663 | 0.422414 | 0.625280207 |
| metabolite958 | 115.3968375 | 0.325643 | 40892.70321 | 0.11572 | 0.247800665 |  | 28.74808 | 0.279683 | 2954.958 | 0.158134 | 0.424934899 |  | 307866200.4 | 0.066306558 | 1.42945E+18 | 0.088025 | 0.475490674 |  | 0.859813 | 0.440816 | 1.677069 | 0.658546 | 0.804696336 |
| metabolite959 | 141.9566945 | 0.172554 | 116784.937 | 0.150726 | 0.298476153 |  | 276.1969 | 1.47579 | 51690.78 | 0.037476 | 0.220921127 |  | 4.44315E+18 | 104723255.3 | 1.88512E+29 | 0.000823 | 0.057911888 |  | 0.835504 | 0.389774 | 1.790954 | 0.644994 | 0.79600051 |
| metabolite960 | 28064.95191 | 35.34851 | 22282168.62 | 0.003268 | 0.016545856 |  | 4.728066 | 0.02011 | 1111.61 | 0.578194 | 0.79199206 |  | 2.31785E+14 | 1676.552344 | 3.20445E+25 | 0.012903 | 0.237046184 |  | 0.470204 | 0.217836 | 1.014945 | 0.057142 | 0.182964091 |
| metabolite961 | 2.349929167 | 0.008996 | 613.8581578 | 0.764057 | 0.863296259 |  | 9.916623 | 0.126288 | 778.6891 | 0.304997 | 0.585108179 |  | 0.00484437 | 3.2275E-12 | 7271236.087 | 0.62199 | 0.879305472 |  | 1.047911 | 0.559741 | 1.96183 | 0.883972 | 0.942861226 |
| metabolite962 | 792798.9227 | 1159.715 | 541969424.8 | 8.55E-05 | 0.001064853 |  | 4.301406 | 0.017491 | 1057.81 | 0.604488 | 0.809056761 |  | 1.22881E+13 | 63.12448288 | 2.39204E+24 | 0.024979 | 0.298066703 |  | 0.328802 | 0.15365 | 0.703618 | 0.00498 | 0.036665306 |
| metabolite963 | 0.31088507 | 0.000582 | 165.9576744 | 0.716078 | 0.830945733 |  | 0.942678 | 0.00669 | 132.8382 | 0.981387 | 0.992080789 |  | 5078.570914 | 2.29374E-07 | 1.12445E+14 | 0.484093 | 0.802417608 |  | 1.258837 | 0.621062 | 2.551553 | 0.524408 | 0.704079982 |
| metabolite964 | 0.007946081 | 3.21E-05 | 1.96872301 | 0.088375 | 0.20510182 |  | 110.5596 | 1.484944 | 8231.57 | 0.034562 | 0.212415725 |  | 1481.341223 | 9.41556E-07 | 2.33058E+12 | 0.500623 | 0.812201491 |  | 2.130124 | 1.1537 | 3.932939 | 0.017279 | 0.082525007 |
| metabolite965 | 1455.105985 | 6.003253 | 352697.6624 | 0.010596 | 0.041477676 |  | 13.05398 | 0.155926 | 1092.866 | 0.257856 | 0.542461848 |  | 10.21275451 | 4.78694E-09 | 21788505575 | 0.832483 | 0.952506813 |  | 0.825578 | 0.437063 | 1.559451 | 0.555934 | 0.726886063 |
| metabolite966 | 7401.873201 | 15.45291 | 3545464.415 | 0.005535 | 0.024927373 |  | 9.315035 | 0.061834 | 1403.267 | 0.384985 | 0.656863779 |  | 1.42858E+12 | 71.82373298 | 2.84146E+22 | 0.02255 | 0.28756568 |  | 0.462505 | 0.228442 | 0.936389 | 0.034328 | 0.132422671 |
| metabolite967 | 2458.493161 | 6.675857 | 905380.2056 | 0.010892 | 0.042236232 |  | 3.244391 | 0.027067 | 388.8894 | 0.630792 | 0.826559459 |  | 97107.91073 | 9.78655E-06 | 9.63562E+14 | 0.330284 | 0.705226226 |  | 0.607603 | 0.308104 | 1.198236 | 0.153241 | 0.337494888 |
| metabolite968 | 1610181.562 | 2454.044 | 1056494784 | 3.43E-05 | 0.000539276 |  | 2.439272 | 0.009795 | 607.4777 | 0.752025 | 0.895049124 |  | 4.71121E+11 | 2.052472167 | 1.0814E+23 | 0.046442 | 0.381293312 |  | 0.264518 | 0.124966 | 0.559909 | 0.000727 | 0.010343378 |
| metabolite969 | 0.194498641 | 0.000599 | 63.1957297 | 0.580095 | 0.723706447 |  | 25.97226 | 0.282776 | 2385.486 | 0.160659 | 0.428617074 |  | 0.000939211 | 2.7128E-13 | 3251688.449 | 0.535223 | 0.829425866 |  | 1.459024 | 0.762769 | 2.79082 | 0.256064 | 0.462185832 |
| metabolite970 | 1053675.296 | 3671.85 | 302363004.1 | 4.93E-06 | 0.000125243 |  | 0.681062 | 0.005086 | 91.19647 | 0.878101 | 0.954279989 |  | 65584371.82 | 0.004550949 | 9.45146E+17 | 0.134355 | 0.533823619 |  | 0.322933 | 0.165532 | 0.630003 | 0.001238 | 0.014449187 |
| metabolite971 | 7699.359705 | 25.20179 | 2352219.336 | 0.00273 | 0.014591755 |  | 3.167315 | 0.029165 | 343.974 | 0.630726 | 0.826559459 |  | 460157135.7 | 0.091893449 | 2.30424E+18 | 0.082793 | 0.465905693 |  | 0.566699 | 0.292056 | 1.09961 | 0.095919 | 0.256329689 |
| metabolite972 | 0.22651506 | 0.000949 | 54.05791672 | 0.596068 | 0.736354143 |  | 31.71124 | 0.444007 | 2264.835 | 0.115313 | 0.36576254 |  | 730103.1766 | 0.00076613 | 6.9577E+14 | 0.203252 | 0.597241307 |  | 1.361255 | 0.736085 | 2.517395 | 0.327656 | 0.540583797 |
| metabolite973 | 0.009837517 | 2.96E-05 | 3.266451827 | 0.121523 | 0.256487055 |  | 465.8413 | 5.292193 | 41005.33 | 0.008263 | 0.126652263 |  | 6400669.321 | 0.001608324 | 2.54728E+16 | 0.167424 | 0.568636202 |  | 2.025668 | 1.059756 | 3.87196 | 0.034911 | 0.133563091 |
| metabolite974 | 19233.91869 | 46.01269 | 8040035.29 | 0.001773 | 0.010593717 |  | 3.429081 | 0.023995 | 490.0347 | 0.627402 | 0.825011301 |  | 1.53835E+11 | 9.742171107 | 2.42916E+21 | 0.033725 | 0.333743961 |  | 0.430315 | 0.215181 | 0.860537 | 0.018785 | 0.086777083 |
| metabolite975 | 161086.2192 | 592.1602 | 43820525.43 | 5.58E-05 | 0.000772507 |  | 0.61112 | 0.005287 | 70.64042 | 0.839349 | 0.938160513 |  | 113380930.6 | 0.016333294 | 7.87057E+17 | 0.111529 | 0.512820569 |  | 0.364205 | 0.189542 | 0.699819 | 0.003032 | 0.026664124 |
| metabolite976 | 0.009427103 | 2.35E-05 | 3.774524222 | 0.129966 | 0.269143522 |  | 412.4929 | 4.005936 | 42474.55 | 0.012237 | 0.142361591 |  | 6.96082E+11 | 125.3520026 | 3.86536E+21 | 0.018919 | 0.274905275 |  | 1.956489 | 1.00065 | 3.825363 | 0.052278 | 0.172908107 |
| metabolite977 | 108.0831233 | 0.281086 | 41560.15672 | 0.125899 | 0.262921446 |  | 0.19609 | 0.001736 | 22.15199 | 0.500758 | 0.741149898 |  | 114.5173257 | 1.38354E-08 | 9.47877E+11 | 0.68488 | 0.903557902 |  | 0.673013 | 0.343163 | 1.319917 | 0.251674 | 0.456704962 |
| metabolite978 | 10.40746649 | 0.038994 | 2777.757693 | 0.412947 | 0.580912483 |  | 22.66158 | 0.285447 | 1799.101 | 0.164822 | 0.433105382 |  | 59563333.05 | 0.043915511 | 8.07867E+16 | 0.098 | 0.492678215 |  | 0.901798 | 0.479846 | 1.694791 | 0.748732 | 0.862366684 |
| metabolite979 | 218403.3128 | 464.5066 | 102689619.3 | 0.000156 | 0.001656261 |  | 0.430498 | 0.002455 | 75.48648 | 0.749782 | 0.894781821 |  | 864259.6543 | 1.45193E-05 | 5.14449E+16 | 0.282518 | 0.665728265 |  | 0.324544 | 0.159694 | 0.659567 | 0.002376 | 0.022844341 |
| metabolite980 | 7184.499126 | 17.19327 | 3002164.166 | 0.00472 | 0.0220611 |  | 4.101846 | 0.029935 | 562.0629 | 0.575073 | 0.791284873 |  | 3783068603 | 0.256222622 | 5.58561E+19 | 0.067554 | 0.436748477 |  | 0.515389 | 0.257491 | 1.031591 | 0.063817 | 0.196210763 |
| metabolite981 | 679713.8021 | 1388.41 | 332762571.4 | 4.47E-05 | 0.00066642 |  | 0.128058 | 0.000675 | 24.28129 | 0.444096 | 0.699260511 |  | 35.81410162 | 3.45645E-10 | 3.71088E+12 | 0.782666 | 0.941497897 |  | 0.312294 | 0.151945 | 0.64186 | 0.001994 | 0.020326379 |
| metabolite982 | 42.00384061 | 0.086928 | 20296.25592 | 0.238408 | 0.402700282 |  | 1.906314 | 0.014253 | 254.9743 | 0.796669 | 0.920823742 |  | 17.84652863 | 9.82454E-10 | 3.24187E+11 | 0.811464 | 0.946368417 |  | 0.93182 | 0.462528 | 1.877268 | 0.843719 | 0.923161255 |
| metabolite983 | 9.60696919 | 0.023787 | 3880.001032 | 0.461501 | 0.625960962 |  | 1.532149 | 0.013436 | 174.7158 | 0.860175 | 0.94777649 |  | 66.81976587 | 8.03029E-09 | 5.56005E+11 | 0.719114 | 0.920542269 |  | 0.779556 | 0.396504 | 1.532666 | 0.471813 | 0.666501547 |
| metabolite984 | 3347083.349 | 14386.48 | 778714693 | 3.77E-07 | 2.15E-05 |  | 0.460302 | 0.003711 | 57.09191 | 0.753001 | 0.89553175 |  | 12509197.42 | 0.001198496 | 1.30564E+17 | 0.167772 | 0.568636202 |  | 0.291276 | 0.151972 | 0.558273 | 0.000319 | 0.005739144 |
| metabolite985 | 19667.48097 | 55.10569 | 7019417.341 | 0.001313 | 0.008504085 |  | 0.020627 | 0.000171 | 2.49503 | 0.115514 | 0.36576254 |  | 0.091325748 | 6.3243E-12 | 1318784235 | 0.841438 | 0.954296849 |  | 0.419888 | 0.213863 | 0.824388 | 0.013121 | 0.068581972 |
| metabolite986 | 31511.27812 | 60.94029 | 16293992.78 | 0.001532 | 0.009444014 |  | 0.088696 | 0.000525 | 14.97668 | 0.356587 | 0.629539526 |  | 0.538707056 | 8.76908E-12 | 33094159016 | 0.961161 | 0.988599765 |  | 0.406495 | 0.198414 | 0.832796 | 0.015435 | 0.076070371 |
| metabolite987 | 271.7112229 | 0.365422 | 202032.3739 | 0.099423 | 0.222667813 |  | 1.287229 | 0.006619 | 250.3311 | 0.925357 | 0.973632359 |  | 50.42680358 | 4.60972E-10 | 5.51631E+12 | 0.76298 | 0.938107312 |  | 0.814502 | 0.38361 | 1.729396 | 0.594341 | 0.759449929 |
| metabolite988 | 46.70510173 | 0.142057 | 15355.51824 | 0.196296 | 0.353104799 |  | 19.36011 | 0.201538 | 1859.768 | 0.205936 | 0.484609395 |  | 2414670.418 | 0.000663097 | 8.79303E+15 | 0.193426 | 0.592990803 |  | 0.790207 | 0.409941 | 1.523213 | 0.4834 | 0.674143893 |
| metabolite989 | 111880.0789 | 222.1768 | 56338704.99 | 0.000385 | 0.003281679 |  | 0.085702 | 0.000489 | 15.02558 | 0.353342 | 0.627092503 |  | 0.50190845 | 6.80148E-12 | 37037844292 | 0.957039 | 0.9871034 |  | 0.366133 | 0.178571 | 0.750704 | 0.007105 | 0.046188927 |
| metabolite990 | 0.000535576 | 2.38E-06 | 0.12069057 | 0.007473 | 0.031766058 |  | 8.892275 | 0.110525 | 715.4267 | 0.331123 | 0.607338753 |  | 14.12478432 | 8.30499E-09 | 24022848105 | 0.80754 | 0.9460629 |  | 1.733297 | 0.930588 | 3.22841 | 0.085819 | 0.239498277 |
| metabolite991 | 0.768080331 | 0.003758 | 156.984185 | 0.922733 | 0.959644668 |  | 1.56677 | 0.023765 | 103.2941 | 0.833965 | 0.935280435 |  | 1.166273301 | 1.94343E-09 | 699893893.1 | 0.988127 | 0.994828538 |  | 1.21953 | 0.670543 | 2.217983 | 0.516814 | 0.698432273 |
| metabolite992 | 0.010938772 | 4.07E-05 | 2.943350468 | 0.116536 | 0.248757465 |  | 0.083677 | 0.000996 | 7.031322 | 0.274882 | 0.563632158 |  | 7.95692E-08 | 4.56657E-17 | 138.6434126 | 0.134981 | 0.534467195 |  | 1.469282 | 0.779988 | 2.767722 | 0.236222 | 0.438309063 |
| metabolite993 | 26283.84607 | 52.06153 | 13269693.38 | 0.001766 | 0.010576258 |  | 1.111374 | 0.006622 | 186.5242 | 0.967847 | 0.984606206 |  | 8290775.12 | 0.00018263 | 3.76374E+17 | 0.205875 | 0.597241307 |  | 0.46332 | 0.225814 | 0.95063 | 0.038168 | 0.140515707 |
| metabolite994 | 742290.0123 | 1167.398 | 471985200.6 | 7.76E-05 | 0.000999015 |  | 0.030819 | 0.000137 | 6.936939 | 0.210624 | 0.489127291 |  | 0.000523347 | 2.01788E-15 | 135732637.1 | 0.574264 | 0.855522386 |  | 0.298078 | 0.141208 | 0.629217 | 0.001937 | 0.019971069 |
| metabolite995 | 104412.4915 | 104.4818 | 104343192.3 | 0.001391 | 0.008913424 |  | 0.033804 | 0.000118 | 9.720095 | 0.243443 | 0.526615886 |  | 0.007649641 | 9.0022E-15 | 6500298188 | 0.728708 | 0.924067425 |  | 0.337571 | 0.153299 | 0.743348 | 0.008101 | 0.050189115 |
| metabolite996 | 63710.92955 | 99.34352 | 40859058.74 | 0.001089 | 0.007372939 |  | 64.8533 | 0.328699 | 12795.77 | 0.124624 | 0.379704697 |  | 1.04228E+18 | 21855646.91 | 4.97057E+28 | 0.00127 | 0.072851874 |  | 0.470236 | 0.221889 | 0.996546 | 0.051442 | 0.171058581 |
| metabolite997 | 810511.8449 | 1485.58 | 442203927.7 | 4.80E-05 | 0.000700588 |  | 0.05626 | 0.000275 | 11.50792 | 0.291414 | 0.574315439 |  | 0.003395297 | 2.16306E-14 | 532951377.1 | 0.666392 | 0.899920753 |  | 0.306633 | 0.147376 | 0.637985 | 0.002019 | 0.020468032 |
| metabolite998 | 5246.670092 | 5.62498 | 4893803.284 | 0.015638 | 0.056396437 |  | 0.005614 | 2.43E-05 | 1.299147 | 0.064712 | 0.279282325 |  | 5.94392E-10 | 2.07446E-21 | 170.3102399 | 0.117343 | 0.519645256 |  | 0.426 | 0.196301 | 0.924476 | 0.033031 | 0.129848069 |
| metabolite999 | 12237.6196 | 49.28827 | 3038437.316 | 0.001122 | 0.007555993 |  | 0.385978 | 0.004068 | 36.62271 | 0.68271 | 0.857663676 |  | 10.72697603 | 3.06068E-09 | 37595609428 | 0.8328 | 0.952506813 |  | 0.559881 | 0.294416 | 1.064705 | 0.079673 | 0.227436831 |
| metabolite1000 | 2965.578095 | 13.46726 | 653039.6855 | 0.004436 | 0.020946226 |  | 20.02623 | 0.253101 | 1584.543 | 0.181721 | 0.45686231 |  | 2914551.391 | 0.002042872 | 4.15817E+15 | 0.169103 | 0.568636202 |  | 0.571035 | 0.306682 | 1.063252 | 0.08004 | 0.227958367 |
| metabolite1001 | 100447.9032 | 175.3033 | 57556153.54 | 0.000558 | 0.004365827 |  | 0.005778 | 3.22E-05 | 1.03717 | 0.054156 | 0.257328813 |  | 2.2954E-06 | 2.24728E-17 | 234455.1999 | 0.317585 | 0.700308709 |  | 0.327791 | 0.158614 | 0.677413 | 0.003218 | 0.027794187 |
| metabolite1002 | 0.408134662 | 0.001499 | 111.1244137 | 0.75466 | 0.856697287 |  | 0.385853 | 0.004674 | 31.85384 | 0.673177 | 0.851700797 |  | 769032.616 | 0.000496336 | 1.19155E+15 | 0.212005 | 0.603798949 |  | 0.878669 | 0.46733 | 1.652063 | 0.688796 | 0.827138021 |
| metabolite1003 | 216300.3666 | 757.1307 | 61793619.75 | 4.34E-05 | 0.000653505 |  | 0.141784 | 0.00118 | 17.04039 | 0.425716 | 0.688981122 |  | 6257920.601 | 0.000649565 | 6.02889E+16 | 0.184849 | 0.58220839 |  | 0.319718 | 0.166334 | 0.614546 | 0.000876 | 0.01182043 |
| metabolite1004 | 0.044874147 | 1E-04 | 20.14299249 | 0.321313 | 0.494168131 |  | 10.15887 | 0.082699 | 1247.928 | 0.346958 | 0.620752441 |  | 31671411.91 | 0.002995047 | 3.34912E+17 | 0.14532 | 0.546460871 |  | 1.448922 | 0.728578 | 2.881468 | 0.292714 | 0.506092088 |
| metabolite1005 | 0.012225422 | 4.74E-05 | 3.150578653 | 0.122826 | 0.258394593 |  | 95.09064 | 1.243234 | 7273.152 | 0.041894 | 0.234848291 |  | 2239.10024 | 1.29268E-06 | 3.87843E+12 | 0.478742 | 0.79982887 |  | 2.130234 | 1.150222 | 3.945236 | 0.017821 | 0.083858367 |
| metabolite1006 | 32.48403309 | 0.124787 | 8456.112248 | 0.222565 | 0.386090472 |  | 16.8041 | 0.210916 | 1338.818 | 0.209148 | 0.488014005 |  | 181056913.7 | 0.140432367 | 2.33433E+17 | 0.078376 | 0.45805738 |  | 0.731317 | 0.390261 | 1.370429 | 0.330919 | 0.543788165 |
| metabolite1007 | 10.19442228 | 0.020498 | 5070.150042 | 0.465162 | 0.629087665 |  | 2.87269 | 0.021448 | 384.7563 | 0.673603 | 0.851700797 |  | 82639.46799 | 4.87637E-06 | 1.40048E+15 | 0.348144 | 0.717869012 |  | 0.835901 | 0.414967 | 1.683821 | 0.616898 | 0.774675165 |
| metabolite1008 | 0.362739489 | 0.001072 | 122.7161113 | 0.73354 | 0.843037451 |  | 231.0036 | 2.630268 | 20287.91 | 0.018845 | 0.16512884 |  | 235091.081 | 6.44039E-05 | 8.58144E+14 | 0.273301 | 0.657303854 |  | 1.570866 | 0.819282 | 3.011929 | 0.176638 | 0.369635162 |
| metabolite1009 | 1.650119837 | 0.001791 | 1520.346515 | 0.885907 | 0.93770703 |  | 3.1109 | 0.014459 | 669.3126 | 0.679579 | 0.855467047 |  | 53596606.6 | 0.000361229 | 7.95228E+18 | 0.17783 | 0.575285157 |  | 1.078173 | 0.499804 | 2.325827 | 0.84818 | 0.925219154 |
| metabolite1010 | 0.38220795 | 0.00144 | 101.4640677 | 0.736195 | 0.844689876 |  | 15.63075 | 0.198235 | 1232.476 | 0.219897 | 0.500900006 |  | 68.43862354 | 4.24211E-08 | 1.10413E+11 | 0.696789 | 0.907533437 |  | 1.153826 | 0.615466 | 2.1631 | 0.656294 | 0.803831309 |
| metabolite1011 | 109.5892387 | 0.431037 | 27862.5726 | 0.0993 | 0.222667813 |  | 2.325415 | 0.028202 | 191.744 | 0.70847 | 0.871746821 |  | 2542.497387 | 1.49935E-06 | 4.31138E+12 | 0.471102 | 0.798982501 |  | 0.686354 | 0.366404 | 1.285689 | 0.2424 | 0.446531184 |
| metabolite1012 | 28.84904732 | 0.138545 | 6007.181107 | 0.219684 | 0.383141446 |  | 3.601693 | 0.052627 | 246.4911 | 0.553507 | 0.782501074 |  | 0.086488924 | 1.17575E-10 | 63621701.29 | 0.814651 | 0.946906762 |  | 0.618762 | 0.33998 | 1.126145 | 0.118991 | 0.293015754 |
| metabolite1013 | 243280.5159 | 394.0551 | 150195767.4 | 0.000251 | 0.002368385 |  | 0.010177 | 5.04E-05 | 2.054894 | 0.093061 | 0.330362211 |  | 9.30938E-06 | 5.53681E-17 | 1565244.213 | 0.38161 | 0.741054231 |  | 0.324137 | 0.154666 | 0.679302 | 0.003496 | 0.029086888 |
| metabolite1014 | 1.512319128 | 0.005955 | 384.0374201 | 0.883855 | 0.936742232 |  | 14.41776 | 0.189422 | 1097.398 | 0.229894 | 0.511535674 |  | 2562.015299 | 1.9664E-06 | 3.33805E+12 | 0.465131 | 0.792880411 |  | 0.997771 | 0.534732 | 1.86177 | 0.994418 | 0.996028815 |
| metabolite1015 | 0.97702486 | 0.003062 | 311.7602771 | 0.99371 | 0.996605199 |  | 1.532834 | 0.016372 | 143.5133 | 0.854018 | 0.944356957 |  | 79850.47226 | 2.71149E-05 | 2.35151E+14 | 0.312446 | 0.695897083 |  | 0.993826 | 0.519122 | 1.902615 | 0.985121 | 0.991802164 |
| metabolite1016 | 0.07628145 | 0.000268 | 21.72245268 | 0.374093 | 0.543989349 |  | 44.13295 | 0.536227 | 3632.262 | 0.095176 | 0.333421024 |  | 0.020081282 | 8.90468E-12 | 45286075.52 | 0.722772 | 0.922880405 |  | 1.669189 | 0.887456 | 3.139528 | 0.114777 | 0.286441796 |
| metabolite1017 | 0.104972754 | 0.000331 | 33.29268522 | 0.444659 | 0.611385737 |  | 34.68208 | 0.385628 | 3119.189 | 0.125221 | 0.380272847 |  | 1226346.401 | 0.000424799 | 3.54032E+15 | 0.209798 | 0.602134961 |  | 1.265005 | 0.661062 | 2.420707 | 0.47922 | 0.671596275 |
| metabolite1018 | 0.080331718 | 0.000232 | 27.76200503 | 0.399636 | 0.568436212 |  | 16.05526 | 0.163226 | 1579.229 | 0.23825 | 0.518915778 |  | 6534.993211 | 1.46482E-06 | 2.91545E+13 | 0.440015 | 0.782677331 |  | 1.507021 | 0.781945 | 2.904439 | 0.223091 | 0.42124391 |
| metabolite1019 | 0.002308096 | 1.04E-05 | 0.510983612 | 0.02961 | 0.09160278 |  | 500.6475 | 7.602479 | 32969.24 | 0.004375 | 0.105752273 |  | 12312.29609 | 1.04634E-05 | 1.44879E+13 | 0.37869 | 0.738475807 |  | 2.439218 | 1.340199 | 4.43948 | 0.004261 | 0.03294026 |
| metabolite1020 | 0.019673138 | 7.87E-05 | 4.920917105 | 0.165982 | 0.31714854 |  | 374.795 | 5.370981 | 26153.75 | 0.007241 | 0.122988815 |  | 2316.67187 | 1.56895E-06 | 3.42073E+12 | 0.473488 | 0.798982501 |  | 1.878506 | 1.014216 | 3.479324 | 0.047409 | 0.16127195 |
| metabolite1021 | 0.011539269 | 5.65E-05 | 2.357349423 | 0.102998 | 0.228603611 |  | 117.1474 | 1.85436 | 7400.67 | 0.026293 | 0.191394412 |  | 2570.848564 | 3.54361E-06 | 1.86512E+12 | 0.452254 | 0.788682526 |  | 1.998192 | 1.104762 | 3.614145 | 0.02394 | 0.103667638 |
| metabolite1022 | 0.003795508 | 1.79E-05 | 0.806438205 | 0.043858 | 0.12339379 |  | 168.712 | 2.553235 | 11148.1 | 0.018135 | 0.163741265 |  | 5724493.245 | 0.006918479 | 4.73656E+15 | 0.140309 | 0.542758378 |  | 1.899781 | 1.039384 | 3.472412 | 0.039313 | 0.143170416 |
| metabolite1023 | 5.499377171 | 0.031227 | 968.4790388 | 0.519543 | 0.672481345 |  | 4.790003 | 0.081867 | 280.2593 | 0.452117 | 0.703347616 |  | 139788.3791 | 0.000447759 | 4.36413E+13 | 0.237658 | 0.629451823 |  | 0.740849 | 0.41443 | 1.324366 | 0.313694 | 0.526749645 |
| metabolite1024 | 0.02492394 | 0.000115 | 5.410760254 | 0.181388 | 0.335870084 |  | 29.68181 | 0.434428 | 2027.975 | 0.118527 | 0.371517626 |  | 30848.10606 | 3.79196E-05 | 2.50953E+13 | 0.325551 | 0.704504181 |  | 1.856412 | 1.018801 | 3.382668 | 0.045703 | 0.157595802 |
| metabolite1025 | 48.01845709 | 0.144669 | 15938.28863 | 0.193837 | 0.350551082 |  | 1.404314 | 0.014036 | 140.5002 | 0.885368 | 0.957845085 |  | 821031.107 | 0.000211796 | 3.18274E+15 | 0.229243 | 0.621546086 |  | 0.52608 | 0.275201 | 1.005666 | 0.05456 | 0.178546467 |
| metabolite1026 | 8.275563193 | 0.022513 | 3041.951125 | 0.484632 | 0.644092149 |  | 0.227484 | 0.002167 | 23.87596 | 0.534145 | 0.766366251 |  | 0.006357783 | 1.1066E-12 | 36527615.35 | 0.659948 | 0.895497 |  | 0.962429 | 0.494065 | 1.874791 | 0.910577 | 0.957809547 |
| metabolite1027 | 26494.98147 | 91.09582 | 7705996.311 | 0.000629 | 0.004795643 |  | 68.63685 | 0.660607 | 7131.349 | 0.076993 | 0.304869417 |  | 27710081839 | 5.854725008 | 1.3115E+20 | 0.036625 | 0.342359087 |  | 0.503213 | 0.259707 | 0.975033 | 0.044242 | 0.154160119 |
| metabolite1028 | 0.001450162 | 4.91E-06 | 0.428048528 | 0.026263 | 0.084019381 |  | 18.69181 | 0.198045 | 1764.161 | 0.209571 | 0.488116163 |  | 9.56108E+13 | 55372.1336 | 1.65091E+23 | 0.00369 | 0.127024611 |  | 2.411511 | 1.278805 | 4.547514 | 0.007582 | 0.048261146 |
| metabolite1029 | 57457.55625 | 61.92233 | 53314701.41 | 0.002142 | 0.012303139 |  | 0.909219 | 0.003312 | 249.5732 | 0.973558 | 0.987934029 |  | 18.65491352 | 3.22034E-11 | 1.08065E+13 | 0.832693 | 0.952506813 |  | 0.335386 | 0.154197 | 0.729481 | 0.006849 | 0.045146546 |
| metabolite1030 | 1.06153808 | 0.003662 | 307.7244469 | 0.983566 | 0.991851034 |  | 4.721281 | 0.054859 | 406.3264 | 0.496135 | 0.736708633 |  | 3575795.524 | 0.001899507 | 6.73139E+15 | 0.168859 | 0.568636202 |  | 1.058014 | 0.558706 | 2.003548 | 0.862885 | 0.934393468 |
| metabolite1031 | 0.004060774 | 1.46E-05 | 1.133280016 | 0.057879 | 0.149887211 |  | 2.343634 | 0.025919 | 211.9152 | 0.711646 | 0.873381216 |  | 0.084757763 | 3.0619E-11 | 234621795.8 | 0.824343 | 0.950338031 |  | 1.4184 | 0.746825 | 2.693883 | 0.287834 | 0.501008785 |
| metabolite1032 | 386273.5336 | 548.2583 | 272147695.4 | 0.000201 | 0.002022948 |  | 1.656328 | 0.006796 | 403.6952 | 0.857519 | 0.946535588 |  | 762.9180157 | 2.3831E-09 | 2.44238E+14 | 0.624363 | 0.879305472 |  | 0.349818 | 0.163331 | 0.749227 | 0.007953 | 0.049434852 |
| metabolite1033 | 190118582.8 | 1735201 | 20830479157 | 1.65E-12 | 3.05E-09 |  | 0.04155 | 0.000419 | 4.118164 | 0.177717 | 0.452679722 |  | 0.708056414 | 1.37991E-10 | 3633153903 | 0.975911 | 0.991698663 |  | 0.178085 | 0.09971 | 0.318064 | 5.51E-08 | 3.40E-05 |
| metabolite1034 | 17.60440693 | 0.033021 | 9385.312867 | 0.372547 | 0.542803568 |  | 0.646123 | 0.004525 | 92.25626 | 0.863322 | 0.948212877 |  | 46.76567237 | 1.89433E-09 | 1.15451E+12 | 0.753395 | 0.936460506 |  | 0.774588 | 0.38151 | 1.572666 | 0.481104 | 0.673219063 |
| metabolite1035 | 81426.27444 | 186.5298 | 35545196.23 | 0.000407 | 0.003458099 |  | 0.154791 | 0.000989 | 24.23263 | 0.470821 | 0.717917212 |  | 4.652366318 | 1.13469E-10 | 1.90753E+11 | 0.902086 | 0.969275008 |  | 0.388726 | 0.192474 | 0.785085 | 0.009625 | 0.055732714 |
| metabolite1036 | 13911.90188 | 12.98344 | 14906754.49 | 0.008478 | 0.034765856 |  | 0.011278 | 4.14E-05 | 3.071725 | 0.119794 | 0.372764958 |  | 4.10428E-06 | 5.98903E-18 | 2812668.03 | 0.374302 | 0.736141064 |  | 0.392122 | 0.177611 | 0.865711 | 0.022345 | 0.098133487 |
| metabolite1037 | 22.7516088 | 0.073655 | 7027.867505 | 0.287725 | 0.459879553 |  | 0.42902 | 0.004603 | 39.98856 | 0.715242 | 0.874551186 |  | 12.719054 | 3.97077E-09 | 40741295522 | 0.820273 | 0.947841909 |  | 1.12962 | 0.590492 | 2.160979 | 0.713376 | 0.841761483 |
| metabolite1038 | 5516.267498 | 19.89833 | 1529233.84 | 0.003313 | 0.016680248 |  | 7.404186 | 0.075139 | 729.611 | 0.394494 | 0.665096037 |  | 958.8584896 | 2.22579E-07 | 4.13072E+12 | 0.545349 | 0.837314745 |  | 0.585087 | 0.305073 | 1.122116 | 0.109535 | 0.277843212 |
| metabolite1039 | 0.938379161 | 0.002413 | 364.8689529 | 0.983359 | 0.991851034 |  | 4.012423 | 0.036915 | 436.1285 | 0.562537 | 0.786576663 |  | 12698017.04 | 0.002258671 | 7.13869E+16 | 0.156085 | 0.560993738 |  | 0.885624 | 0.452588 | 1.732987 | 0.723539 | 0.84862569 |
| metabolite1040 | 8328.818932 | 39.5773 | 1752752.888 | 0.001268 | 0.008306779 |  | 0.892561 | 0.010796 | 73.79067 | 0.959847 | 0.981849859 |  | 0.128906387 | 7.25966E-11 | 228893042.1 | 0.850799 | 0.955623187 |  | 0.452146 | 0.244686 | 0.835502 | 0.012683 | 0.066948993 |
| metabolite1041 | 11587.84932 | 16.86729 | 7960866.661 | 0.005895 | 0.026200648 |  | 0.004234 | 2.28E-05 | 0.787359 | 0.042753 | 0.23574656 |  | 1.01843E-09 | 9.54391E-21 | 108.6762912 | 0.112858 | 0.513062936 |  | 0.403188 | 0.191887 | 0.847167 | 0.018162 | 0.08499062 |
| metabolite1042 | 108517.6179 | 263.8501 | 44631677.92 | 0.000258 | 0.002416274 |  | 0.209138 | 0.001372 | 31.87514 | 0.543015 | 0.771783989 |  | 51.66550692 | 1.47205E-09 | 1.81334E+12 | 0.750761 | 0.935191746 |  | 0.38605 | 0.192066 | 0.775954 | 0.008672 | 0.052411165 |
| metabolite1043 | 31941.39307 | 110.0886 | 9267558.541 | 0.000502 | 0.004034789 |  | 4.488973 | 0.040493 | 497.6422 | 0.533181 | 0.766318985 |  | 100141143 | 0.017004047 | 5.89757E+17 | 0.11133 | 0.512820569 |  | 0.452616 | 0.234319 | 0.874283 | 0.020022 | 0.090120173 |
| metabolite1044 | 0.005544231 | 0.000115 | 0.266846322 | 0.009794 | 0.038995961 |  | 8.722073 | 0.385886 | 197.1426 | 0.17613 | 0.450616586 |  | 18.3201121 | 4.77921E-06 | 70226413.25 | 0.707644 | 0.915324846 |  | 1.818576 | 1.176214 | 2.811748 | 0.008248 | 0.050737505 |
| metabolite1045 | 1634.161515 | 6.135732 | 435234.7868 | 0.010687 | 0.041659863 |  | 16.11417 | 0.17907 | 1450.085 | 0.228544 | 0.510919397 |  | 37553.86057 | 1.32569E-05 | 1.06381E+14 | 0.344887 | 0.715414351 |  | 0.565737 | 0.298557 | 1.072015 | 0.083446 | 0.234774606 |
| metabolite1046 | 1595.265974 | 1.273542 | 1998264.935 | 0.045117 | 0.125603099 |  | 0.192529 | 0.000637 | 58.21114 | 0.572968 | 0.791219919 |  | 0.036204391 | 3.77386E-14 | 34732507454 | 0.814056 | 0.946906762 |  | 0.62636 | 0.277655 | 1.413001 | 0.262131 | 0.469482559 |
| metabolite1047 | 231.8009361 | 0.474784 | 113170.8874 | 0.087462 | 0.203744871 |  | 16.58976 | 0.122059 | 2254.81 | 0.264808 | 0.5520808 |  | 0.234863229 | 1.04951E-11 | 5255858609 | 0.90537 | 0.970355785 |  | 0.639923 | 0.317234 | 1.29085 | 0.21506 | 0.411597102 |
| metabolite1048 | 17605.94409 | 75.48407 | 4106419.738 | 0.000639 | 0.004848862 |  | 18.71892 | 0.209443 | 1672.997 | 0.203912 | 0.48289214 |  | 27582789.87 | 0.011458963 | 6.63943E+16 | 0.122909 | 0.526740039 |  | 0.447748 | 0.238493 | 0.840607 | 0.013871 | 0.071295718 |
| metabolite1049 | 63.26399709 | 0.174729 | 22905.91421 | 0.170464 | 0.322756829 |  | 28.38995 | 0.274861 | 2932.347 | 0.160112 | 0.427962837 |  | 1.53795E+11 | 40.36497873 | 5.85976E+20 | 0.023999 | 0.294657193 |  | 0.92689 | 0.47469 | 1.809867 | 0.824436 | 0.91246088 |
| metabolite1050 | 27.83186842 | 0.095976 | 8070.911627 | 0.252688 | 0.419001144 |  | 13.66964 | 0.1573 | 1187.912 | 0.25342 | 0.538009389 |  | 46.53087306 | 1.82905E-08 | 1.18374E+11 | 0.728845 | 0.924067425 |  | 0.9957 | 0.523744 | 1.892944 | 0.989534 | 0.993652937 |
| metabolite1051 | 55.26827311 | 0.175136 | 17441.2471 | 0.174515 | 0.327911394 |  | 0.368848 | 0.003838 | 35.44904 | 0.669349 | 0.850377708 |  | 0.000747608 | 2.07597E-13 | 2692328.118 | 0.522714 | 0.824510163 |  | 0.459867 | 0.243109 | 0.869889 | 0.0186 | 0.086388888 |
| metabolite1052 | 1944.227119 | 8.484233 | 445534.5444 | 0.007343 | 0.031393493 |  | 17.4325 | 0.216466 | 1403.879 | 0.204426 | 0.48289214 |  | 3087595.823 | 0.002021239 | 4.71654E+15 | 0.168836 | 0.568636202 |  | 0.651272 | 0.347795 | 1.219554 | 0.183032 | 0.377980424 |
| metabolite1053 | 2134.638519 | 5.364192 | 849462.7672 | 0.013516 | 0.050512345 |  | 38.14269 | 0.314458 | 4626.573 | 0.139738 | 0.402308456 |  | 3.05514E+13 | 4427.399694 | 2.10821E+23 | 0.008334 | 0.188291096 |  | 0.654069 | 0.328507 | 1.302275 | 0.229499 | 0.428837762 |
| metabolite1054 | 538.3389081 | 1.436922 | 201687.2629 | 0.039838 | 0.114071947 |  | 52.17683 | 0.474935 | 5732.195 | 0.101884 | 0.343719589 |  | 1.5279E+13 | 3343.337086 | 6.98244E+22 | 0.008602 | 0.188291096 |  | 0.733142 | 0.372191 | 1.444141 | 0.371416 | 0.587204488 |
| metabolite1055 | 133.3001807 | 0.161249 | 110195.7234 | 0.156226 | 0.305133943 |  | 4.421677 | 0.021402 | 913.5031 | 0.585779 | 0.797266959 |  | 0.00017391 | 1.19505E-15 | 25308273.47 | 0.510539 | 0.818406864 |  | 0.49773 | 0.234535 | 1.05628 | 0.071857 | 0.212478785 |
| metabolite1056 | 301.7533906 | 0.775586 | 117401.6622 | 0.063216 | 0.159249077 |  | 54.46727 | 0.489631 | 6059.016 | 0.099145 | 0.342576214 |  | 8.32436E+12 | 1655.01002 | 4.18699E+22 | 0.010295 | 0.209346746 |  | 0.790859 | 0.40029 | 1.562512 | 0.500839 | 0.688375799 |
| metabolite1057 | 20.14947667 | 0.074159 | 5474.747776 | 0.295895 | 0.467461337 |  | 1.660189 | 0.019696 | 139.9409 | 0.823116 | 0.930140685 |  | 1465.54077 | 7.78405E-07 | 2.75924E+12 | 0.504849 | 0.816435137 |  | 0.770922 | 0.409522 | 1.451254 | 0.421914 | 0.625122608 |
| metabolite1058 | 1.415997211 | 0.003146 | 637.4180837 | 0.911352 | 0.953758328 |  | 1249.593 | 12.25356 | 127431.1 | 0.003118 | 0.096298132 |  | 8.76727E+17 | 269338663 | 2.85384E+27 | 0.000341 | 0.047140629 |  | 1.309846 | 0.659364 | 2.602047 | 0.442502 | 0.640205342 |
| metabolite1059 | 0.255101198 | 0.000674 | 96.57172552 | 0.652841 | 0.784051457 |  | 621.069 | 6.740581 | 57224.55 | 0.006262 | 0.118571609 |  | 3.883E+15 | 1675316.298 | 8.99989E+24 | 0.001467 | 0.076862474 |  | 1.495093 | 0.768817 | 2.90746 | 0.238456 | 0.441571081 |
| metabolite1060 | 0.30353537 | 0.000735 | 125.3162589 | 0.698776 | 0.818547297 |  | 1482.233 | 15.70131 | 139925.5 | 0.002119 | 0.088493313 |  | 5.45373E+17 | 226660850.3 | 1.31223E+27 | 0.000331 | 0.047140629 |  | 1.529579 | 0.779354 | 3.001989 | 0.219303 | 0.4167085 |
| metabolite1061 | 0.335570212 | 0.000764 | 147.4815777 | 0.725748 | 0.838235227 |  | 2742.744 | 28.60947 | 262942.5 | 0.000935 | 0.07423496 |  | 1.13971E+19 | 4532953106 | 2.86557E+28 | 0.000126 | 0.047140629 |  | 1.464446 | 0.740294 | 2.896963 | 0.275438 | 0.485582398 |
| metabolite1062 | 23430.9954 | 56.00711 | 9802532.621 | 0.001446 | 0.009206128 |  | 0.645297 | 0.004454 | 93.49164 | 0.863324 | 0.948212877 |  | 25473832.08 | 0.001174485 | 5.52511E+17 | 0.163 | 0.566515099 |  | 0.504733 | 0.250538 | 1.016833 | 0.058287 | 0.185032976 |
| metabolite1063 | 73864.71472 | 189.6669 | 28766201.96 | 0.000357 | 0.003095972 |  | 1.004385 | 0.006934 | 145.4855 | 0.998628 | 0.999753507 |  | 290554173 | 0.014354105 | 5.88136E+18 | 0.110347 | 0.512129781 |  | 0.494333 | 0.245583 | 0.995042 | 0.050875 | 0.170086796 |
| metabolite1064 | 0.006252282 | 3.11E-05 | 1.255484118 | 0.063297 | 0.159249077 |  | 0.049634 | 0.000741 | 3.323173 | 0.164271 | 0.432346784 |  | 4.99343E-05 | 7.05028E-14 | 35366.42405 | 0.342837 | 0.714142474 |  | 1.240661 | 0.677276 | 2.27269 | 0.486484 | 0.675651729 |
| metabolite1065 | 550.3423367 | 1.687403 | 179492.7823 | 0.034778 | 0.10281162 |  | 13.83085 | 0.135689 | 1409.789 | 0.267938 | 0.555007395 |  | 52572439478 | 15.26194112 | 1.81095E+20 | 0.029643 | 0.316108398 |  | 0.586086 | 0.303618 | 1.131346 | 0.114177 | 0.285710915 |
| metabolite1066 | 1789.976328 | 3.839094 | 834575.8937 | 0.018576 | 0.064112952 |  | 126.8386 | 0.964563 | 16679.09 | 0.054243 | 0.257410714 |  | 1.57182E+15 | 155649.7609 | 1.5873E+25 | 0.003572 | 0.127024611 |  | 0.793385 | 0.390685 | 1.611169 | 0.523258 | 0.703390434 |
| metabolite1067 | 40340.24785 | 98.64073 | 16497602.35 | 0.000777 | 0.005683897 |  | 4.699425 | 0.032453 | 680.5178 | 0.543377 | 0.771834939 |  | 1.89636E+12 | 122.4984216 | 2.93569E+22 | 0.019937 | 0.279023995 |  | 0.508981 | 0.252313 | 1.026745 | 0.06187 | 0.191813782 |
| metabolite1068 | 19566.73595 | 49.3314 | 7760922.311 | 0.001592 | 0.009765729 |  | 6.000235 | 0.043911 | 819.9132 | 0.476613 | 0.721922701 |  | 53042738367 | 3.907308603 | 7.20069E+20 | 0.040346 | 0.358402676 |  | 0.589241 | 0.293158 | 1.184359 | 0.140388 | 0.32159339 |
| metabolite1069 | 4.407408276 | 0.009938 | 1954.568677 | 0.634288 | 0.76948146 |  | 281.2864 | 2.588839 | 30562.75 | 0.020141 | 0.16720854 |  | 5.49697E+16 | 14712285.05 | 2.05384E+26 | 0.000855 | 0.057911888 |  | 1.220675 | 0.614592 | 2.424453 | 0.570127 | 0.739669372 |
| metabolite1070 | 3461.99165 | 9.868217 | 1214544.247 | 0.007458 | 0.031761014 |  | 6.486908 | 0.055906 | 752.6967 | 0.442402 | 0.69832133 |  | 6016257123 | 0.909431849 | 3.98E+19 | 0.053485 | 0.394594431 |  | 0.646032 | 0.328283 | 1.271334 | 0.208539 | 0.406240048 |
| metabolite1071 | 8748.892298 | 20.57218 | 3720709.748 | 0.004004 | 0.019346172 |  | 9.064251 | 0.065425 | 1255.795 | 0.382836 | 0.654400502 |  | 2.18239E+11 | 15.40183138 | 3.09238E+21 | 0.030669 | 0.32241539 |  | 0.660273 | 0.326707 | 1.334408 | 0.250018 | 0.454714498 |
| metabolite1072 | 90896.04716 | 290.1941 | 28470916.22 | 0.000169 | 0.001764867 |  | 6.533308 | 0.053129 | 803.3998 | 0.446189 | 0.699833598 |  | 16194406043 | 1.906313118 | 1.37574E+20 | 0.046289 | 0.381128688 |  | 0.497103 | 0.252402 | 0.979036 | 0.045658 | 0.157595802 |
| metabolite1073 | 0.045866605 | 0.000116 | 18.18672167 | 0.314834 | 0.488032073 |  | 896.5751 | 9.375188 | 85741.94 | 0.004216 | 0.105014469 |  | 5.82311E+15 | 1959950.551 | 1.73007E+25 | 0.001471 | 0.076862474 |  | 1.659249 | 0.848717 | 3.243845 | 0.14159 | 0.323347373 |
| metabolite1074 | 0.143506619 | 0.00039 | 52.8134287 | 0.520876 | 0.673274971 |  | 1752.769 | 20.48825 | 149949.4 | 0.001342 | 0.082976723 |  | 1.73838E+17 | 103591327.5 | 2.9172E+26 | 0.000384 | 0.047140629 |  | 1.672034 | 0.86433 | 3.234526 | 0.129627 | 0.30669642 |
| metabolite1075 | 35289.98255 | 106.1377 | 11733657.53 | 0.000597 | 0.004613812 |  | 3.8073 | 0.030822 | 470.2932 | 0.587501 | 0.798613737 |  | 10174106836 | 1.188282637 | 8.7111E+19 | 0.050776 | 0.389316104 |  | 0.586033 | 0.296099 | 1.159866 | 0.127823 | 0.305440663 |
| metabolite1076 | 22.22603268 | 0.047117 | 10484.4587 | 0.325621 | 0.499123723 |  | 301.1635 | 2.600695 | 34875.08 | 0.020319 | 0.167939347 |  | 2.19093E+16 | 3996393.052 | 1.20113E+26 | 0.001349 | 0.075321073 |  | 1.172934 | 0.584877 | 2.352244 | 0.654106 | 0.802707389 |
| metabolite1077 | 299.6752579 | 0.690452 | 130067.2581 | 0.068372 | 0.168367766 |  | 51.3287 | 0.422819 | 6231.123 | 0.110581 | 0.357150654 |  | 1.01426E+13 | 1336.28667 | 7.69841E+22 | 0.011183 | 0.216142524 |  | 0.951882 | 0.475358 | 1.9061 | 0.889545 | 0.947503591 |
| metabolite1078 | 0.028825303 | 8.12E-05 | 10.23599235 | 0.239063 | 0.403255237 |  | 1153.793 | 13.27932 | 100249 | 0.002488 | 0.089719453 |  | 5.15902E+16 | 29599197.19 | 8.99195E+25 | 0.000577 | 0.053508115 |  | 1.791872 | 0.929168 | 3.455571 | 0.0845 | 0.236663355 |
| metabolite1079 | 515.9983643 | 1.361347 | 195581.4768 | 0.041559 | 0.118545151 |  | 106.2764 | 0.981704 | 11505.18 | 0.053424 | 0.25621721 |  | 2.38083E+14 | 57400.34789 | 9.87508E+23 | 0.004118 | 0.13777711 |  | 0.802321 | 0.40636 | 1.584109 | 0.527009 | 0.70655032 |
| metabolite1080 | 3.357407926 | 0.012865 | 876.157876 | 0.67048 | 0.797705167 |  | 403.5469 | 5.809567 | 28031.37 | 0.006513 | 0.121443014 |  | 1066829265 | 0.986961013 | 1.15316E+18 | 0.052649 | 0.394365301 |  | 1.234336 | 0.659992 | 2.308492 | 0.511188 | 0.695870474 |
| metabolite1081 | 0.000751973 | 2.22E-06 | 0.255181416 | 0.017171 | 0.060398478 |  | 93.59387 | 0.911565 | 9609.643 | 0.05732 | 0.264422304 |  | 7.38094E+11 | 179.3400275 | 3.03771E+21 | 0.017169 | 0.267707456 |  | 2.064775 | 1.0674 | 3.994093 | 0.033422 | 0.131109253 |
| metabolite1082 | 6313.015972 | 17.4275 | 2286855.736 | 0.004359 | 0.020685361 |  | 14.20251 | 0.118256 | 1705.718 | 0.279778 | 0.56735959 |  | 3.58377E+11 | 50.08076143 | 2.56453E+21 | 0.023433 | 0.292802915 |  | 0.699204 | 0.352339 | 1.387545 | 0.308394 | 0.520914323 |
| metabolite1083 | 8406.943976 | 26.20289 | 2697286.803 | 0.002698 | 0.014490012 |  | 1.192226 | 0.010491 | 135.4925 | 0.942089 | 0.97690349 |  | 9041.739693 | 1.16383E-06 | 7.02451E+13 | 0.434698 | 0.779380463 |  | 0.4639 | 0.239334 | 0.899176 | 0.024842 | 0.107198242 |
| metabolite1084 | 0.018356485 | 5.76E-05 | 5.852999421 | 0.176826 | 0.33024805 |  | 3016.608 | 39.81882 | 228533.3 | 0.000432 | 0.07423496 |  | 6.14384E+16 | 52574075.83 | 7.17973E+25 | 0.000432 | 0.047140629 |  | 1.941466 | 1.020672 | 3.692949 | 0.045542 | 0.157509111 |
| metabolite1085 | 0.842347852 | 0.00175 | 405.4366583 | 0.956681 | 0.980554024 |  | 1067.424 | 9.830001 | 115909.8 | 0.004296 | 0.105642828 |  | 3.38785E+18 | 869768702.7 | 1.31961E+28 | 0.000248 | 0.047140629 |  | 1.621458 | 0.813367 | 3.232399 | 0.17248 | 0.364598211 |
| metabolite1086 | 0.020816 | 7.37E-05 | 5.882538083 | 0.181482 | 0.335870084 |  | 1521.972 | 21.32268 | 108635.4 | 0.001051 | 0.075024912 |  | 6.03899E+14 | 637791.5022 | 5.71807E+23 | 0.001642 | 0.079139598 |  | 1.945697 | 1.037482 | 3.648965 | 0.040327 | 0.145628527 |
| metabolite1087 | 0.011470214 | 3.65E-05 | 3.605346933 | 0.130629 | 0.269724284 |  | 1478.56 | 18.72297 | 116762.5 | 0.001413 | 0.082989557 |  | 1.89825E+11 | 83.97114456 | 4.29116E+20 | 0.019859 | 0.279023995 |  | 2.462076 | 1.307837 | 4.634994 | 0.006179 | 0.041768163 |
| metabolite1088 | 2.201083147 | 0.00523 | 926.3066739 | 0.798485 | 0.884267152 |  | 600.8102 | 5.990491 | 60257.65 | 0.007549 | 0.125141666 |  | 6.31441E+16 | 21392268.25 | 1.86384E+26 | 0.000725 | 0.056021378 |  | 1.386171 | 0.703604 | 2.730896 | 0.347283 | 0.559382136 |
| metabolite1089 | 135.2525111 | 0.373711 | 48950.21917 | 0.105398 | 0.231418475 |  | 114.5406 | 1.138812 | 11520.38 | 0.046295 | 0.241970689 |  | 3.8815E+13 | 12098.01142 | 1.24533E+23 | 0.005998 | 0.165405637 |  | 0.961403 | 0.491226 | 1.88161 | 0.908739 | 0.957528306 |
| metabolite1090 | 761.2520519 | 2.486299 | 233079.2622 | 0.025022 | 0.080822448 |  | 0.720448 | 0.007165 | 72.44264 | 0.8894 | 0.95849259 |  | 1137.111725 | 2.5747E-07 | 5.02203E+12 | 0.535888 | 0.82979146 |  | 0.765813 | 0.396715 | 1.478316 | 0.428245 | 0.630641913 |
| metabolite1091 | 26.63997484 | 0.072615 | 9773.356179 | 0.278291 | 0.449211692 |  | 1.129843 | 0.01054 | 121.1091 | 0.959269 | 0.981849859 |  | 0.059875529 | 9.65692E-12 | 371244505.1 | 0.80711 | 0.9460629 |  | 0.803171 | 0.412042 | 1.565576 | 0.521121 | 0.701451728 |
| metabolite1092 | 0.001393661 | 6.56E-06 | 0.29619843 | 0.017826 | 0.06211656 |  | 969.6674 | 15.51465 | 60604.31 | 0.001482 | 0.082989557 |  | 6.33525E+11 | 1003.91017 | 3.9979E+20 | 0.009789 | 0.202953467 |  | 1.890419 | 1.029487 | 3.471325 | 0.042352 | 0.149970475 |
| metabolite1093 | 22866.15384 | 81.27818 | 6432981.227 | 0.000697 | 0.005218501 |  | 0.423903 | 0.003952 | 45.46577 | 0.719676 | 0.877659088 |  | 84222996.68 | 0.017181413 | 4.1286E+17 | 0.111772 | 0.512820569 |  | 0.441726 | 0.230218 | 0.847551 | 0.015538 | 0.076374969 |
| metabolite1094 | 1163.917026 | 5.159809 | 262549.0302 | 0.01202 | 0.045939258 |  | 1.259279 | 0.015613 | 101.5713 | 0.918209 | 0.972080981 |  | 8770.307125 | 5.92693E-06 | 1.29778E+13 | 0.401172 | 0.76001764 |  | 0.596979 | 0.320932 | 1.110465 | 0.106124 | 0.271979928 |
| metabolite1095 | 233.1758997 | 0.640477 | 84891.47667 | 0.072702 | 0.176915659 |  | 0.140104 | 0.001277 | 15.37025 | 0.413982 | 0.679773848 |  | 8.65301E-06 | 1.28498E-15 | 58268.82924 | 0.314857 | 0.697572488 |  | 0.375911 | 0.196441 | 0.719343 | 0.00382 | 0.03063876 |
| metabolite1096 | 7428.354648 | 19.98383 | 2761255.326 | 0.003855 | 0.018847154 |  | 7.124997 | 0.05714 | 888.4485 | 0.426858 | 0.689909047 |  | 3.26819E+11 | 40.0248036 | 2.6686E+21 | 0.024712 | 0.297752183 |  | 0.631548 | 0.317724 | 1.255342 | 0.192496 | 0.389081744 |
| metabolite1097 | 5.275796454 | 0.01149 | 2422.414638 | 0.595913 | 0.736354143 |  | 596.7597 | 5.514408 | 64580.31 | 0.008617 | 0.126652263 |  | 1.00292E+17 | 24080887.31 | 4.17693E+26 | 0.000757 | 0.056768812 |  | 1.427174 | 0.717184 | 2.840033 | 0.313194 | 0.52662634 |
| metabolite1098 | 0.004312565 | 1.46E-05 | 1.271793161 | 0.063125 | 0.159142938 |  | 2722.121 | 36.79198 | 201401 | 0.000474 | 0.07423496 |  | 5.14349E+13 | 33756.93126 | 7.83706E+22 | 0.004158 | 0.13777711 |  | 2.030327 | 1.073628 | 3.839529 | 0.03148 | 0.126279015 |
| metabolite1099 | 0.025002848 | 7.6E-05 | 8.228482483 | 0.214902 | 0.37671285 |  | 654.7917 | 7.762 | 55237.34 | 0.00498 | 0.110248598 |  | 1.92111E+16 | 13859573.01 | 2.6629E+25 | 0.000691 | 0.055755268 |  | 2.106875 | 1.107966 | 4.006372 | 0.024971 | 0.107350324 |
| metabolite1100 | 748.9148062 | 3.131559 | 179103.5506 | 0.019589 | 0.066999978 |  | 0.608034 | 0.00732 | 50.50465 | 0.825776 | 0.931343691 |  | 27.84199166 | 1.5346E-08 | 50513170102 | 0.760305 | 0.938107312 |  | 0.69272 | 0.36944 | 1.298888 | 0.254814 | 0.460911308 |
| metabolite1101 | 352340.4868 | 1412.006 | 87920170.43 | 1.46E-05 | 0.000282406 |  | 0.823236 | 0.007253 | 93.443 | 0.935929 | 0.975459713 |  | 1838845124 | 0.316450589 | 1.06852E+19 | 0.065576 | 0.430715508 |  | 0.39055 | 0.203092 | 0.751037 | 0.00572 | 0.040331076 |
| metabolite1102 | 2536.402799 | 4.342157 | 1481599.713 | 0.017514 | 0.061315916 |  | 95.56501 | 0.597093 | 15295.21 | 0.081016 | 0.310772257 |  | 2.11301E+18 | 124620190.8 | 3.58274E+28 | 0.000646 | 0.054960347 |  | 0.620979 | 0.299035 | 1.28953 | 0.203926 | 0.401257255 |
| metabolite1103 | 6.74244814 | 0.022568 | 2014.364624 | 0.513008 | 0.667755528 |  | 66.63787 | 0.795165 | 5584.507 | 0.065739 | 0.281707409 |  | 30092779078 | 18.19030079 | 4.97834E+19 | 0.027906 | 0.305488117 |  | 1.103153 | 0.579946 | 2.098378 | 0.765306 | 0.873324216 |
| metabolite1104 | 0.073055006 | 0.000231 | 23.12642294 | 0.374998 | 0.545091025 |  | 95.6717 | 1.09508 | 8358.36 | 0.047962 | 0.246134378 |  | 4.63374E+12 | 2679.584422 | 8.01303E+21 | 0.008311 | 0.188291096 |  | 1.94341 | 1.025776 | 3.681936 | 0.043921 | 0.154055957 |
| metabolite1105 | 772.5940897 | 2.182152 | 273538.1134 | 0.028411 | 0.088674013 |  | 31.60647 | 0.293418 | 3404.593 | 0.150875 | 0.418146878 |  | 2.83995E+11 | 61.40410086 | 1.31348E+21 | 0.022022 | 0.284438275 |  | 0.705985 | 0.360339 | 1.383182 | 0.312483 | 0.525667529 |
| metabolite1106 | 0.189054925 | 0.000502 | 71.20403401 | 0.583124 | 0.726408842 |  | 1664.612 | 19.07959 | 145230.3 | 0.001512 | 0.082989557 |  | 4.33508E+17 | 252823725.6 | 7.4332E+26 | 0.000289 | 0.047140629 |  | 1.760932 | 0.909525 | 3.409341 | 0.096031 | 0.25638074 |
| metabolite1107 | 0.205269526 | 0.000565 | 74.60975099 | 0.599657 | 0.74004934 |  | 1242.014 | 14.42719 | 106923.1 | 0.002205 | 0.088493313 |  | 4.24275E+16 | 24661822.43 | 7.29911E+25 | 0.000609 | 0.054960347 |  | 1.766171 | 0.916098 | 3.405052 | 0.092243 | 0.250890714 |
| metabolite1108 | 205.0459381 | 0.346664 | 121281.173 | 0.10495 | 0.230919108 |  | 395.8314 | 2.767851 | 56607.99 | 0.019915 | 0.16570848 |  | 1.85825E+20 | 20013065440 | 1.72542E+30 | 0.000121 | 0.047140629 |  | 0.803544 | 0.38863 | 1.661433 | 0.55628 | 0.726886063 |
| metabolite1109 | 1483.363841 | 4.075335 | 539923.2342 | 0.016835 | 0.059609832 |  | 32.46083 | 0.289078 | 3645.05 | 0.151341 | 0.418499549 |  | 1.56388E+13 | 3278.73121 | 7.45935E+22 | 0.008676 | 0.188291096 |  | 0.732802 | 0.371551 | 1.445289 | 0.371589 | 0.587204488 |
| metabolite1110 | 4.861847962 | 0.016253 | 1454.32657 | 0.587737 | 0.730439149 |  | 326.388 | 4.153089 | 25650.58 | 0.010606 | 0.135235336 |  | 1.25776E+14 | 113081.1273 | 1.39895E+23 | 0.002819 | 0.107866667 |  | 1.248379 | 0.657159 | 2.371499 | 0.499417 | 0.686929342 |
| metabolite1111 | 1.844572021 | 0.006376 | 533.6220777 | 0.832701 | 0.905937469 |  | 160.3893 | 2.044171 | 12584.43 | 0.024443 | 0.185877348 |  | 40157759769 | 28.91705377 | 5.5768E+19 | 0.024936 | 0.298066703 |  | 1.23034 | 0.650474 | 2.327126 | 0.52513 | 0.704795194 |
| metabolite1112 | 0.001498072 | 7.35E-06 | 0.305265108 | 0.018181 | 0.06293841 |  | 885.9924 | 14.6237 | 53678.81 | 0.001572 | 0.082989557 |  | 29589859059 | 48.68377543 | 1.79846E+19 | 0.021259 | 0.283005659 |  | 2.33097 | 1.287352 | 4.220616 | 0.006136 | 0.041706949 |
| metabolite1113 | 0.008438623 | 1.94E-05 | 3.663117413 | 0.126163 | 0.263054301 |  | 3.172599 | 0.025377 | 396.6362 | 0.64023 | 0.831955879 |  | 280022.5649 | 2.348E-05 | 3.33955E+15 | 0.291652 | 0.674763106 |  | 1.566697 | 0.788726 | 3.112032 | 0.202444 | 0.400037958 |
| metabolite1114 | 15.71691482 | 0.039942 | 6184.508313 | 0.368145 | 0.539142472 |  | 0.037356 | 0.000346 | 4.036151 | 0.171602 | 0.443346793 |  | 3.22587E-05 | 4.45754E-15 | 233452.9517 | 0.373872 | 0.736141064 |  | 0.851313 | 0.433521 | 1.671736 | 0.641032 | 0.792692073 |
| metabolite1115 | 606023.2331 | 2928.639 | 125404393.2 | 3.37E-06 | 9.48E-05 |  | 0.017035 | 0.000177 | 1.64042 | 0.083298 | 0.315235932 |  | 0.976236631 | 1.9425E-10 | 4906249655 | 0.99832 | 0.999851958 |  | 0.281932 | 0.151818 | 0.523557 | 0.000111 | 0.003022452 |
| metabolite1116 | 20445.85066 | 73.7622 | 5667304.008 | 0.000771 | 0.005653319 |  | 4.52062 | 0.043084 | 474.325 | 0.526436 | 0.761936106 |  | 3.52456E+11 | 104.3197141 | 1.19081E+21 | 0.019256 | 0.277589364 |  | 0.494721 | 0.257274 | 0.951316 | 0.03714 | 0.138701319 |
| metabolite1117 | 1.075010348 | 0.002694 | 428.998911 | 0.981158 | 0.991308526 |  | 941.6018 | 10.04187 | 88291.74 | 0.003811 | 0.104558924 |  | 1.16777E+16 | 4449921.19 | 3.06452E+25 | 0.001129 | 0.066489481 |  | 1.49527 | 0.764796 | 2.923434 | 0.24207 | 0.446260049 |
| metabolite1118 | 0.57427662 | 0.001572 | 209.8545879 | 0.854176 | 0.919062416 |  | 1252.07 | 14.56361 | 107643.7 | 0.002174 | 0.088493313 |  | 1.4767E+16 | 8080709.784 | 2.69859E+25 | 0.000872 | 0.057911888 |  | 1.629141 | 0.843231 | 3.147538 | 0.149178 | 0.333698345 |
| metabolite1119 | 0.999569049 | 0.002617 | 381.7535649 | 0.999887 | 0.999886871 |  | 2642.19 | 30.97672 | 225368.2 | 0.000734 | 0.07423496 |  | 1.33324E+17 | 70343620.29 | 2.52692E+26 | 0.000449 | 0.047571942 |  | 1.356539 | 0.696056 | 2.643747 | 0.372344 | 0.587736382 |
| metabolite1120 | 28.54042913 | 0.046905 | 17365.97401 | 0.307784 | 0.480718964 |  | 816.308 | 5.980252 | 111426.5 | 0.008651 | 0.126652263 |  | 3.89102E+20 | 47485974207 | 3.18832E+30 | 8.81E-05 | 0.047140629 |  | 0.960633 | 0.465027 | 1.984435 | 0.913791 | 0.958407831 |
| metabolite1121 | 0.012310419 | 5.59E-05 | 2.712191563 | 0.112993 | 0.243505413 |  | 736.4195 | 11.99123 | 45225.86 | 0.002149 | 0.088493313 |  | 14977269360 | 23.61660737 | 9.49834E+18 | 0.025405 | 0.298662816 |  | 1.885401 | 1.031405 | 3.446501 | 0.041691 | 0.148490198 |
| metabolite1122 | 0.001485161 | 7.5E-06 | 0.294085537 | 0.017432 | 0.061085904 |  | 730.1398 | 12.19389 | 43718.96 | 0.002048 | 0.088493313 |  | 106420136.5 | 0.15877661 | 7.13282E+16 | 0.077398 | 0.45805738 |  | 2.358095 | 1.307281 | 4.25357 | 0.005209 | 0.037752632 |
| metabolite1123 | 153.6625054 | 0.333655 | 70768.20221 | 0.110418 | 0.23906754 |  | 348.5074 | 2.979177 | 40768.78 | 0.017628 | 0.161922002 |  | 6.14704E+18 | 1473407071 | 2.56454E+28 | 0.000214 | 0.047140629 |  | 0.817954 | 0.407093 | 1.643478 | 0.573579 | 0.742950349 |
| metabolite1124 | 4680.00261 | 20.46681 | 1070143.278 | 0.002869 | 0.015081172 |  | 2.352724 | 0.027468 | 201.5188 | 0.707033 | 0.871405269 |  | 45986585.29 | 0.027622412 | 7.65598E+16 | 0.106214 | 0.504684321 |  | 0.497343 | 0.26658 | 0.927865 | 0.030223 | 0.12244119 |
| metabolite1125 | 0.000347298 | 1.78E-06 | 0.067573727 | 0.003739 | 0.018453346 |  | 173.5833 | 2.594438 | 11613.76 | 0.017847 | 0.162567222 |  | 12021960.65 | 0.013889617 | 1.04054E+16 | 0.123346 | 0.526740039 |  | 2.051774 | 1.12373 | 3.746252 | 0.021083 | 0.093825933 |
| metabolite1126 | 0.004736669 | 2.41E-05 | 0.932048287 | 0.049486 | 0.134044972 |  | 307.3147 | 5.112103 | 18474.26 | 0.007147 | 0.122988815 |  | 2863147140 | 5.812259755 | 1.4104E+18 | 0.035183 | 0.338247665 |  | 1.934123 | 1.069051 | 3.499208 | 0.031313 | 0.125896895 |
| metabolite1127 | 57682.08417 | 106.9804 | 31101246.31 | 0.000889 | 0.006330137 |  | 0.010153 | 5.97E-05 | 1.727093 | 0.082624 | 0.313545344 |  | 4.84254E-11 | 8.80706E-22 | 2.662658006 | 0.062402 | 0.420282514 |  | 0.343541 | 0.167586 | 0.70424 | 0.004273 | 0.032963835 |
| metabolite1128 | 0.274953051 | 0.000738 | 102.4954412 | 0.669911 | 0.797318838 |  | 1583.478 | 18.30038 | 137013.8 | 0.001591 | 0.082989557 |  | 2.16176E+17 | 127192079 | 3.67413E+26 | 0.00036 | 0.047140629 |  | 1.769721 | 0.915611 | 3.420572 | 0.092352 | 0.250890714 |
| metabolite1129 | 0.36695649 | 0.001046 | 128.7929151 | 0.738054 | 0.846128795 |  | 1371.432 | 16.54625 | 113670.9 | 0.001763 | 0.084988806 |  | 4.37292E+16 | 29820138.28 | 6.41258E+25 | 0.000551 | 0.052825881 |  | 1.6237 | 0.844007 | 3.12367 | 0.149331 | 0.333698345 |
| metabolite1130 | 1.188903358 | 0.003713 | 380.650062 | 0.953226 | 0.97799917 |  | 277.4973 | 3.337721 | 23071.06 | 0.01409 | 0.149726748 |  | 5.93509E+14 | 457346.7003 | 7.70209E+23 | 0.001925 | 0.087127805 |  | 1.56747 | 0.822877 | 2.985819 | 0.17437 | 0.36662055 |
| metabolite1131 | 16477.85346 | 45.61581 | 5952314.3 | 0.001622 | 0.009882607 |  | 0.065504 | 0.000526 | 8.155444 | 0.270533 | 0.55743827 |  | 0.022523429 | 1.55926E-12 | 325349387 | 0.751228 | 0.935191746 |  | 0.39417 | 0.20131 | 0.771793 | 0.007674 | 0.048598438 |
| metabolite1132 | 2.03826647 | 0.004618 | 899.6446242 | 0.819147 | 0.896976489 |  | 966.6505 | 9.495667 | 98404.16 | 0.004313 | 0.105642828 |  | 3.79637E+19 | 16295950536 | 8.84419E+28 | 7.99E-05 | 0.047140629 |  | 1.185235 | 0.597226 | 2.352176 | 0.627946 | 0.783033822 |
| metabolite1133 | 4.140353783 | 0.008114 | 2112.70853 | 0.65601 | 0.786322462 |  | 1554.59 | 13.8583 | 174389.9 | 0.002848 | 0.09238113 |  | 1.05334E+20 | 26497380076 | 4.18729E+29 | 8.25E-05 | 0.047140629 |  | 1.132849 | 0.561122 | 2.287106 | 0.728506 | 0.851190927 |
| metabolite1134 | 0.000974242 | 5.69E-06 | 0.166788804 | 0.009416 | 0.037818243 |  | 106.5773 | 1.795125 | 6327.539 | 0.027029 | 0.193061777 |  | 242.7224207 | 4.4463E-07 | 1.32502E+11 | 0.593684 | 0.867253231 |  | 2.084453 | 1.165461 | 3.728093 | 0.014789 | 0.074769919 |
| metabolite1135 | 0.018816677 | 8.8E-05 | 4.02446534 | 0.149502 | 0.296553282 |  | 993.6376 | 17.01928 | 58011.61 | 0.001195 | 0.081136316 |  | 7063950190 | 12.66273252 | 3.94065E+18 | 0.029368 | 0.315894154 |  | 1.789161 | 0.981529 | 3.261337 | 0.060142 | 0.188334379 |
| metabolite1136 | 1.063661078 | 0.004385 | 258.0217363 | 0.982465 | 0.991819145 |  | 0.376146 | 0.005001 | 28.29054 | 0.658202 | 0.84458436 |  | 2.88044E-09 | 3.46172E-18 | 2.396761734 | 0.063201 | 0.424121099 |  | 1.05947 | 0.57082 | 1.966431 | 0.855068 | 0.928909963 |
| metabolite1137 | 0.275164932 | 0.000745 | 101.5978244 | 0.669594 | 0.797293772 |  | 2332.692 | 27.79974 | 195737.5 | 0.000845 | 0.07423496 |  | 5.023E+17 | 324683019.9 | 7.77082E+26 | 0.000258 | 0.047140629 |  | 1.710003 | 0.884796 | 3.304842 | 0.113354 | 0.284396398 |
| metabolite1138 | 0.33381826 | 0.000864 | 128.9319307 | 0.718766 | 0.832763488 |  | 1764.024 | 19.94539 | 156015 | 0.001439 | 0.082989557 |  | 2.54138E+17 | 131734517.4 | 4.90276E+26 | 0.000369 | 0.047140629 |  | 1.673507 | 0.861107 | 3.252355 | 0.131631 | 0.308440007 |
| metabolite1139 | 0.558590518 | 0.001505 | 207.343745 | 0.847383 | 0.91600335 |  | 1.356062 | 0.012843 | 143.1822 | 0.898285 | 0.962093744 |  | 125.3636062 | 2.19744E-08 | 7.15198E+11 | 0.674194 | 0.902621002 |  | 1.568778 | 0.809783 | 3.039164 | 0.184724 | 0.38014775 |
| metabolite1140 | 0.201442287 | 0.000657 | 61.79078205 | 0.584486 | 0.727617419 |  | 1468.661 | 19.80033 | 108935.8 | 0.001223 | 0.081136316 |  | 1.67758E+15 | 1577823.564 | 1.78364E+24 | 0.001276 | 0.072851874 |  | 1.831376 | 0.969574 | 3.459187 | 0.064855 | 0.198740751 |
| metabolite1141 | 232978.3994 | 437.5383 | 124055280.1 | 0.000192 | 0.00194459 |  | 8.006189 | 0.042009 | 1525.847 | 0.439046 | 0.696282563 |  | 1.9102E+14 | 3760.122674 | 9.70407E+24 | 0.010175 | 0.208625442 |  | 0.374124 | 0.180206 | 0.776714 | 0.009537 | 0.055561365 |
| metabolite1142 | 0.067820675 | 0.000209 | 21.97636953 | 0.363564 | 0.534840548 |  | 1.474473 | 0.015294 | 142.1495 | 0.867997 | 0.950093633 |  | 9637.743299 | 2.74633E-06 | 3.38219E+13 | 0.415074 | 0.769732538 |  | 1.016498 | 0.528759 | 1.954138 | 0.96095 | 0.981510624 |
| metabolite1143 | 18.5429442 | 0.049982 | 6879.332627 | 0.335443 | 0.509340154 |  | 205.5545 | 2.124528 | 19888.02 | 0.024333 | 0.185877348 |  | 2.56376E+16 | 12407624.11 | 5.29746E+25 | 0.000787 | 0.057248886 |  | 0.97039 | 0.496964 | 1.894817 | 0.930007 | 0.966583064 |
| metabolite1144 | 9.874442937 | 0.020395 | 4780.709585 | 0.469378 | 0.632255151 |  | 1752.871 | 16.30005 | 188499.8 | 0.002238 | 0.088493313 |  | 1.04627E+20 | 31410622393 | 3.48506E+29 | 7.31E-05 | 0.047140629 |  | 1.09136 | 0.543122 | 2.193003 | 0.806488 | 0.900926262 |
| metabolite1145 | 0.009592452 | 4.53E-05 | 2.029176779 | 0.091749 | 0.210562153 |  | 636.9028 | 10.57082 | 38374.05 | 0.002548 | 0.089719453 |  | 70780765372 | 135.3595813 | 3.70119E+19 | 0.016305 | 0.261934914 |  | 1.990581 | 1.095347 | 3.617498 | 0.025849 | 0.109029127 |
| metabolite1146 | 0.25402023 | 0.000672 | 95.9606886 | 0.651718 | 0.783455532 |  | 1086.958 | 12.16262 | 97140.08 | 0.002863 | 0.09238113 |  | 1.06936E+17 | 56865314.59 | 2.01094E+26 | 0.000479 | 0.048037771 |  | 1.695983 | 0.874997 | 3.287277 | 0.120542 | 0.295362154 |
| metabolite1147 | 0.174621746 | 0.000503 | 60.6219439 | 0.559926 | 0.709417892 |  | 900.8391 | 10.66372 | 76100.16 | 0.003277 | 0.09887434 |  | 1.11708E+16 | 7141752.35 | 1.74727E+25 | 0.000874 | 0.057911888 |  | 1.726166 | 0.899271 | 3.313404 | 0.103655 | 0.268431682 |
| metabolite1148 | 0.004126333 | 2.41E-05 | 0.707586007 | 0.038739 | 0.111874695 |  | 21.00368 | 0.35122 | 1256.064 | 0.147471 | 0.412408021 |  | 0.008241881 | 1.86549E-11 | 3641320.434 | 0.637521 | 0.882670671 |  | 1.636146 | 0.912746 | 2.932878 | 0.101075 | 0.264146231 |
| metabolite1149 | 3293.181023 | 3.667347 | 2957190.003 | 0.021366 | 0.071624534 |  | 94.58622 | 0.419195 | 21342.23 | 0.102688 | 0.345490788 |  | 2.52421E+20 | 3587442928 | 1.7761E+31 | 0.000354 | 0.047140629 |  | 0.608421 | 0.27916 | 1.326035 | 0.213905 | 0.410835897 |
| metabolite1150 | 6.82663139 | 0.020265 | 2299.720711 | 0.519026 | 0.672481345 |  | 256.2299 | 2.921697 | 22471.1 | 0.016714 | 0.159452505 |  | 2.50138E+15 | 1637220.96 | 3.82165E+24 | 0.00136 | 0.075321073 |  | 1.29867 | 0.674607 | 2.500037 | 0.435839 | 0.637273283 |
| metabolite1151 | 2.749840763 | 0.009536 | 792.9703201 | 0.726984 | 0.838538656 |  | 282.0235 | 3.685584 | 21580.64 | 0.012155 | 0.142290974 |  | 3.12565E+12 | 2738.151077 | 3.568E+21 | 0.007933 | 0.185159455 |  | 1.286259 | 0.680509 | 2.431212 | 0.439987 | 0.638204408 |
| metabolite1152 | 1.705607065 | 0.004504 | 645.8208304 | 0.860398 | 0.923881857 |  | 1016.85 | 11.35915 | 91026.58 | 0.00314 | 0.096298132 |  | 3.52654E+18 | 2390564949 | 5.20233E+27 | 0.00013 | 0.047140629 |  | 1.24953 | 0.640991 | 2.435798 | 0.514393 | 0.697316729 |
| metabolite1153 | 2.925284777 | 0.006826 | 1253.568168 | 0.729137 | 0.839623847 |  | 1306.225 | 13.33595 | 127941.7 | 0.002712 | 0.09238113 |  | 3.46661E+18 | 1407232537 | 8.53973E+27 | 0.000185 | 0.047140629 |  | 1.208684 | 0.611056 | 2.390806 | 0.58711 | 0.754152931 |
| metabolite1154 | 1.281398708 | 0.003732 | 439.9450792 | 0.933814 | 0.966904243 |  | 475.9781 | 5.545177 | 40856.25 | 0.007708 | 0.126571062 |  | 3.03146E+14 | 170615.3167 | 5.38623E+23 | 0.002702 | 0.107838143 |  | 1.370238 | 0.711705 | 2.638104 | 0.348014 | 0.559809336 |
| metabolite1155 | 0.473470064 | 0.001275 | 175.7885829 | 0.80485 | 0.888073767 |  | 905.4483 | 10.20909 | 80304.58 | 0.003593 | 0.104558924 |  | 1.82346E+16 | 9457049.439 | 3.51592E+25 | 0.000841 | 0.057911888 |  | 1.545842 | 0.797592 | 2.996053 | 0.199686 | 0.397977255 |
| metabolite1156 | 1.947686819 | 0.007645 | 496.1835359 | 0.813992 | 0.894499648 |  | 9.101175 | 0.118127 | 701.2081 | 0.321255 | 0.599401439 |  | 556563038.1 | 0.559479345 | 5.53662E+17 | 0.05936 | 0.414299218 |  | 1.14666 | 0.614563 | 2.139453 | 0.66798 | 0.812207104 |
| metabolite1157 | 0.001911607 | 1.07E-05 | 0.341039489 | 0.019679 | 0.067182619 |  | 64.84849 | 1.062665 | 3957.34 | 0.049164 | 0.249369471 |  | 3.678568737 | 6.30374E-09 | 2146640476 | 0.899581 | 0.968957237 |  | 1.858522 | 1.033043 | 3.34362 | 0.040916 | 0.146990433 |
| metabolite1158 | 293.0641202 | 0.348285 | 246598.427 | 0.101144 | 0.225704499 |  | 613.7119 | 3.272695 | 115086.3 | 0.017873 | 0.162567222 |  | 6.41618E+21 | 2.06821E+11 | 1.99048E+32 | 8.71E-05 | 0.047140629 |  | 0.771793 | 0.358634 | 1.660923 | 0.509051 | 0.694175389 |
| metabolite1159 | 0.034184435 | 7.98E-05 | 14.64266157 | 0.277234 | 0.448090667 |  | 0.306586 | 0.002542 | 36.97058 | 0.629677 | 0.826559459 |  | 0.001738546 | 1.58869E-13 | 19025411.64 | 0.591094 | 0.866084937 |  | 0.942271 | 0.47444 | 1.871414 | 0.865433 | 0.936333291 |
| metabolite1160 | 18.60661407 | 0.047862 | 7233.381523 | 0.338666 | 0.512542019 |  | 288.104 | 2.90825 | 28540.85 | 0.017356 | 0.161329273 |  | 4.68657E+16 | 19531031.22 | 1.12457E+26 | 0.000709 | 0.056016459 |  | 0.968306 | 0.493302 | 1.900697 | 0.925597 | 0.964585566 |
| metabolite1161 | 0.120772628 | 0.000517 | 28.19202955 | 0.448981 | 0.6148223 |  | 259.619 | 3.976022 | 16952.13 | 0.010378 | 0.133723351 |  | 55189795257 | 88.21002233 | 3.45302E+19 | 0.018366 | 0.272623674 |  | 1.639603 | 0.891861 | 3.014258 | 0.114316 | 0.285867949 |
| metabolite1162 | 1.552724378 | 0.003889 | 619.9660715 | 0.885773 | 0.93770703 |  | 565.3036 | 5.871473 | 54427.25 | 0.007587 | 0.125141666 |  | 1.18165E+16 | 4486727.073 | 3.11209E+25 | 0.001127 | 0.066489481 |  | 1.337044 | 0.68242 | 2.619626 | 0.399118 | 0.610773749 |
| metabolite1163 | 1087.788704 | 1.305654 | 906277.0129 | 0.043955 | 0.123479874 |  | 336.8815 | 1.708528 | 66425.13 | 0.033029 | 0.210580718 |  | 6.21724E+20 | 14934047298 | 2.58832E+31 | 0.000207 | 0.047140629 |  | 0.698221 | 0.323717 | 1.505984 | 0.361672 | 0.574570902 |
| metabolite1164 | 9.642807659 | 0.020933 | 4442.041364 | 0.470416 | 0.632963875 |  | 736.8634 | 6.822981 | 79579.25 | 0.006689 | 0.122236677 |  | 1.57339E+17 | 37325287.36 | 6.63237E+26 | 0.000666 | 0.054960347 |  | 1.075079 | 0.538005 | 2.148298 | 0.837977 | 0.919530925 |
| metabolite1165 | 1.374446903 | 0.003673 | 514.3671685 | 0.916396 | 0.956071204 |  | 855.3644 | 9.563988 | 76500.33 | 0.003936 | 0.104558924 |  | 3.56212E+15 | 1633870.644 | 7.76605E+24 | 0.00146 | 0.076862474 |  | 1.389513 | 0.714875 | 2.700818 | 0.334091 | 0.547137043 |
| metabolite1166 | 4.762746229 | 0.013861 | 1636.509937 | 0.601404 | 0.741219007 |  | 382.3066 | 4.380322 | 33367.02 | 0.010365 | 0.133723351 |  | 8.43107E+16 | 64169490.61 | 1.10774E+26 | 0.000418 | 0.047140629 |  | 1.248003 | 0.646787 | 2.408075 | 0.510211 | 0.694823294 |
| metabolite1167 | 7.525545944 | 0.01796 | 3153.36397 | 0.513713 | 0.667995766 |  | 547.9137 | 5.414662 | 55443.8 | 0.008554 | 0.126652263 |  | 8.42918E+16 | 28431431.93 | 2.49903E+26 | 0.000666 | 0.054960347 |  | 1.081986 | 0.547458 | 2.138417 | 0.821073 | 0.910367889 |
| metabolite1168 | 1.949791058 | 0.008156 | 466.1110567 | 0.811574 | 0.892370706 |  | 8.473039 | 0.115502 | 621.5708 | 0.331643 | 0.607338753 |  | 65.03585236 | 6.03551E-08 | 70079583954 | 0.694745 | 0.907533437 |  | 1.125264 | 0.607353 | 2.084818 | 0.708296 | 0.837715271 |
| metabolite1169 | 130.9536189 | 0.195579 | 87682.40378 | 0.144809 | 0.2886072 |  | 458.1536 | 2.948207 | 71197.4 | 0.019019 | 0.165250229 |  | 1.02771E+20 | 6787451023 | 1.5561E+30 | 0.000196 | 0.047140629 |  | 0.83957 | 0.40086 | 1.758412 | 0.643835 | 0.794833959 |
| metabolite1170 | 4.248653524 | 0.012226 | 1476.418377 | 0.628908 | 0.765206071 |  | 242.6879 | 2.702884 | 21790.58 | 0.018374 | 0.164423236 |  | 4.71166E+13 | 22393.91621 | 9.91328E+22 | 0.004851 | 0.150009525 |  | 1.212321 | 0.62737 | 2.34267 | 0.567901 | 0.738171789 |
| metabolite1171 | 15.03049194 | 0.040669 | 5555.010839 | 0.37091 | 0.54105579 |  | 393.7372 | 4.209235 | 36830.68 | 0.011164 | 0.138431279 |  | 2.33824E+17 | 133904259.8 | 4.08303E+26 | 0.000357 | 0.047140629 |  | 1.042353 | 0.534266 | 2.033631 | 0.903399 | 0.954366418 |
| metabolite1172 | 2.176016349 | 0.005695 | 831.4856808 | 0.798193 | 0.88420729 |  | 248.8915 | 2.579372 | 24016.29 | 0.019693 | 0.165250229 |  | 1.75993E+15 | 709759.4127 | 4.36395E+24 | 0.001905 | 0.087127805 |  | 1.253267 | 0.642202 | 2.445769 | 0.509478 | 0.694221893 |
| metabolite1173 | 1.911840613 | 0.007612 | 480.1944546 | 0.818627 | 0.896672114 |  | 533.823 | 8.062569 | 35344.44 | 0.004046 | 0.104558924 |  | 45576688395 | 57.11975389 | 3.63663E+19 | 0.020709 | 0.280473407 |  | 1.315354 | 0.70722 | 2.446418 | 0.388466 | 0.603430908 |
| metabolite1174 | 36805.52867 | 66.86295 | 20260053.76 | 0.001455 | 0.009244163 |  | 0.506217 | 0.00279 | 91.85791 | 0.797998 | 0.920823742 |  | 2355.759659 | 3.08317E-08 | 1.79997E+14 | 0.544889 | 0.836955166 |  | 0.481544 | 0.231671 | 1.000921 | 0.052795 | 0.174308006 |
| metabolite1175 | 1622469.709 | 6375.798 | 412875059.3 | 1.68E-06 | 6.04E-05 |  | 0.137563 | 0.001105 | 17.13262 | 0.422047 | 0.68573328 |  | 0.055398361 | 4.04885E-12 | 757987141.3 | 0.80848 | 0.9460629 |  | 0.291629 | 0.151774 | 0.560357 | 0.000339 | 0.005939106 |
| metabolite1176 | 7.910639344 | 0.018549 | 3373.758352 | 0.504621 | 0.660019784 |  | 372.733 | 3.566213 | 38957.26 | 0.014029 | 0.149726748 |  | 2.68761E+16 | 7863904.059 | 9.18534E+25 | 0.001009 | 0.063459657 |  | 1.07784 | 0.544235 | 2.134628 | 0.830158 | 0.915768329 |
| metabolite1177 | 55472.06269 | 155.183 | 19829163.8 | 0.000413 | 0.003490871 |  | 0.611544 | 0.004566 | 81.90563 | 0.844333 | 0.938792681 |  | 0.476005464 | 2.59645E-11 | 8726591951 | 0.951018 | 0.986747222 |  | 0.489327 | 0.245944 | 0.97356 | 0.044096 | 0.15415799 |
| metabolite1178 | 40.81852237 | 0.084895 | 19626.03149 | 0.241625 | 0.405916864 |  | 512.371 | 4.417547 | 59427.56 | 0.011415 | 0.139809157 |  | 9.53861E+16 | 16804602.91 | 5.4143E+26 | 0.000901 | 0.058655721 |  | 0.899993 | 0.447075 | 1.811748 | 0.768413 | 0.875204326 |
| metabolite1179 | 13.56305038 | 0.030605 | 6010.643143 | 0.403494 | 0.572824945 |  | 114.1059 | 1.004667 | 12959.67 | 0.052274 | 0.255419086 |  | 4.60672E+14 | 89667.86262 | 2.36672E+24 | 0.003766 | 0.128217975 |  | 1.042262 | 0.523533 | 2.074961 | 0.906416 | 0.95668693 |
| metabolite1180 | 226.2825771 | 0.605236 | 84601.41434 | 0.075558 | 0.181602866 |  | 307.2781 | 3.056852 | 30887.93 | 0.016483 | 0.15805892 |  | 4.30092E+16 | 16361690.15 | 1.13056E+26 | 0.000765 | 0.056768812 |  | 0.78972 | 0.401911 | 1.551731 | 0.494745 | 0.68364345 |
| metabolite1181 | 15.27676094 | 0.036972 | 6312.246952 | 0.376961 | 0.546613589 |  | 373.1228 | 3.635881 | 38290.75 | 0.013649 | 0.149599761 |  | 6.23022E+15 | 1830852.357 | 2.12008E+25 | 0.001539 | 0.077588829 |  | 1.078491 | 0.545951 | 2.130491 | 0.828182 | 0.914697612 |
| metabolite1182 | 831.5740203 | 2.585783 | 267429.7387 | 0.024363 | 0.079101192 |  | 87.40309 | 0.899298 | 8494.736 | 0.058125 | 0.264422304 |  | 2.34884E+13 | 8847.880423 | 6.23547E+22 | 0.006371 | 0.170088592 |  | 0.780387 | 0.401818 | 1.515618 | 0.465602 | 0.660493071 |
| metabolite1183 | 3.288212712 | 0.008952 | 1207.840884 | 0.693588 | 0.813753783 |  | 338.7899 | 3.66634 | 31306.04 | 0.013066 | 0.146937782 |  | 2.93127E+13 | 11024.86086 | 7.79359E+22 | 0.006017 | 0.165405637 |  | 1.273325 | 0.655343 | 2.474056 | 0.477339 | 0.669683116 |
| metabolite1184 | 615.1629872 | 1.01681 | 372169.4202 | 0.051901 | 0.139064695 |  | 27.06026 | 0.165984 | 4411.62 | 0.207095 | 0.485504903 |  | 1.313E+13 | 444.8617078 | 3.87528E+23 | 0.01561 | 0.258606599 |  | 0.730305 | 0.35138 | 1.51786 | 0.40159 | 0.61293609 |
| metabolite1185 | 334.3749455 | 0.801764 | 139450.7672 | 0.061607 | 0.156805671 |  | 30.74916 | 0.257073 | 3677.98 | 0.16326 | 0.431469419 |  | 14532843712 | 1.68808925 | 1.25114E+20 | 0.047412 | 0.384160502 |  | 0.779521 | 0.391439 | 1.55236 | 0.479998 | 0.672178204 |
| metabolite1186 | 56.08280383 | 0.153023 | 20554.33501 | 0.184015 | 0.338561502 |  | 113.3951 | 1.136447 | 11314.6 | 0.046379 | 0.242069301 |  | 2.17422E+14 | 77080.9056 | 6.13284E+23 | 0.003612 | 0.127024611 |  | 0.867774 | 0.444119 | 1.695564 | 0.678953 | 0.818110718 |
| metabolite1187 | 65.83230174 | 0.17102 | 25341.43146 | 0.1708 | 0.322892634 |  | 135.4487 | 1.30987 | 14006.24 | 0.040387 | 0.228124785 |  | 1.29841E+12 | 293.0754299 | 5.75234E+21 | 0.015385 | 0.257536123 |  | 0.908698 | 0.46219 | 1.786565 | 0.781851 | 0.884857504 |
| metabolite1188 | 21.64187567 | 0.056675 | 8264.103244 | 0.312948 | 0.486327375 |  | 141.0979 | 1.401367 | 14206.57 | 0.037689 | 0.220921127 |  | 5.27763E+13 | 16009.70758 | 1.73978E+23 | 0.005595 | 0.160941223 |  | 1.01129 | 0.51607 | 1.981721 | 0.973967 | 0.985146606 |
| metabolite1189 | 3528.348861 | 15.25687 | 815976.6419 | 0.00398 | 0.019283204 |  | 0.020627 | 0.000255 | 1.665746 | 0.086006 | 0.320129285 |  | 0.002193777 | 1.0695E-12 | 4499906.199 | 0.576861 | 0.857178478 |  | 0.471791 | 0.253524 | 0.877971 | 0.019478 | 0.088429298 |
| metabolite1190 | 0.005878094 | 1.5E-05 | 2.306818292 | 0.094665 | 0.214732748 |  | 0.224131 | 0.00193 | 26.026 | 0.538826 | 0.768471369 |  | 5.99325E-05 | 6.74532E-15 | 532502.6508 | 0.40728 | 0.762519149 |  | 1.276809 | 0.647003 | 2.519682 | 0.482545 | 0.673964238 |
| metabolite1191 | 557.6722536 | 0.908031 | 342497.5347 | 0.05609 | 0.146584879 |  | 126.4385 | 0.802083 | 19931.47 | 0.06348 | 0.276819381 |  | 1.97791E+16 | 916450.985 | 4.26877E+26 | 0.002524 | 0.104068846 |  | 0.711843 | 0.342189 | 1.480819 | 0.365059 | 0.57919357 |
| metabolite1192 | 14.90629138 | 0.02248 | 9884.273325 | 0.416773 | 0.585628346 |  | 0.811542 | 0.004796 | 137.3173 | 0.936568 | 0.975459713 |  | 156771.1255 | 3.0692E-06 | 8.0077E+15 | 0.343709 | 0.714568205 |  | 1.208741 | 0.58067 | 2.516153 | 0.613287 | 0.772805844 |
| metabolite1193 | 0.086589509 | 0.000142 | 52.78521253 | 0.456182 | 0.620333428 |  | 1.277468 | 0.008088 | 201.7682 | 0.924635 | 0.97342373 |  | 2022063.52 | 5.79807E-05 | 7.0519E+16 | 0.243572 | 0.636099374 |  | 1.533227 | 0.746467 | 3.149214 | 0.247014 | 0.451560206 |
| metabolite1194 | 16.36311255 | 0.044766 | 5981.110913 | 0.355262 | 0.527561507 |  | 187.7294 | 1.95973 | 17983.26 | 0.026483 | 0.191574909 |  | 2.51886E+12 | 799.09672 | 7.93977E+21 | 0.011846 | 0.223140748 |  | 0.945734 | 0.485287 | 1.843059 | 0.870109 | 0.938371361 |
| metabolite1195 | 422.9909785 | 0.890731 | 200870.1936 | 0.057017 | 0.148606228 |  | 39.09834 | 0.295282 | 5177.011 | 0.144211 | 0.409150286 |  | 1.75403E+11 | 13.21219036 | 2.32863E+21 | 0.031595 | 0.323868345 |  | 0.747822 | 0.370021 | 1.511368 | 0.41997 | 0.624902667 |
| metabolite1196 | 56774.29416 | 155.0316 | 20791382.09 | 0.000423 | 0.003538924 |  | 32.0271 | 0.244573 | 4193.986 | 0.166164 | 0.435784136 |  | 1.16642E+14 | 12626.47997 | 1.07753E+24 | 0.006637 | 0.1721236 |  | 0.519898 | 0.260043 | 1.039422 | 0.066885 | 0.203026447 |
| metabolite1197 | 204.206675 | 0.476992 | 87423.61811 | 0.088118 | 0.204891876 |  | 45.48009 | 0.381008 | 5428.858 | 0.120542 | 0.373711282 |  | 2.36212E+11 | 29.27224513 | 1.90611E+21 | 0.026412 | 0.300748226 |  | 0.802042 | 0.402143 | 1.599607 | 0.532406 | 0.708485456 |
| metabolite1198 | 537.4835835 | 1.553394 | 185972.4692 | 0.037312 | 0.108599557 |  | 29.47093 | 0.281057 | 3090.253 | 0.156869 | 0.422760735 |  | 2.28045E+12 | 620.5873562 | 8.37992E+21 | 0.012731 | 0.235046438 |  | 0.680029 | 0.34876 | 1.325951 | 0.260123 | 0.467358546 |
| metabolite1199 | 1432.442668 | 3.418907 | 600160.3127 | 0.020069 | 0.068140094 |  | 16.03703 | 0.126204 | 2037.867 | 0.264023 | 0.551213494 |  | 7.0759E+11 | 76.15865303 | 6.57422E+21 | 0.021614 | 0.283005659 |  | 0.62332 | 0.312214 | 1.244427 | 0.182961 | 0.377980424 |
| metabolite1200 | 3837.293685 | 9.757896 | 1509016.187 | 0.007855 | 0.032827437 |  | 11.54535 | 0.091652 | 1454.356 | 0.32362 | 0.599929612 |  | 145904346.7 | 0.012591706 | 1.69064E+18 | 0.114683 | 0.516670981 |  | 0.600734 | 0.301818 | 1.195692 | 0.149577 | 0.333698345 |
| metabolite1201 | 2139.630234 | 5.570636 | 821812.4261 | 0.012958 | 0.048673049 |  | 13.92882 | 0.115345 | 1682.015 | 0.283848 | 0.571235911 |  | 63051075883 | 8.02808971 | 4.95191E+20 | 0.034612 | 0.336462533 |  | 0.578445 | 0.292542 | 1.143763 | 0.118376 | 0.292277533 |
| metabolite1202 | 463.0290131 | 1.198034 | 178956.4413 | 0.04585 | 0.127167249 |  | 17.4748 | 0.151708 | 2012.865 | 0.24001 | 0.521473087 |  | 27325624455 | 4.156320263 | 1.79652E+20 | 0.0395 | 0.355749234 |  | 0.73213 | 0.370629 | 1.44623 | 0.371284 | 0.587204488 |
| metabolite1203 | 24477.15109 | 81.24639 | 7374247.072 | 0.000742 | 0.005497379 |  | 7.835934 | 0.070087 | 876.0808 | 0.394126 | 0.665096037 |  | 9900175052 | 1.80233719 | 5.43813E+19 | 0.046697 | 0.381704294 |  | 0.479506 | 0.247098 | 0.930506 | 0.031911 | 0.127108339 |
| metabolite1204 | 0.014528895 | 3.28E-05 | 6.44276801 | 0.176309 | 0.329613726 |  | 2.921862 | 0.023221 | 367.6487 | 0.664651 | 0.848184475 |  | 16.16071966 | 1.17904E-09 | 2.2151E+11 | 0.81568 | 0.946906762 |  | 1.62402 | 0.817595 | 3.225853 | 0.168875 | 0.359963674 |
| metabolite1205 | 3646.988154 | 7.406714 | 1795738.534 | 0.010793 | 0.04198557 |  | 8.360234 | 0.055674 | 1255.409 | 0.408062 | 0.675733682 |  | 4643656423 | 0.193919115 | 1.11199E+20 | 0.070618 | 0.446444805 |  | 0.588126 | 0.288481 | 1.199013 | 0.146953 | 0.330511705 |
| metabolite1206 | 1437.513254 | 2.84492 | 726362.8779 | 0.02396 | 0.078133285 |  | 4.088938 | 0.027291 | 612.6394 | 0.58274 | 0.795225682 |  | 56057612.05 | 0.002172623 | 1.44639E+18 | 0.147476 | 0.548173861 |  | 0.611102 | 0.299938 | 1.245074 | 0.177746 | 0.370694311 |
| metabolite1207 | 5051.757893 | 11.42133 | 2234438.086 | 0.007088 | 0.030587572 |  | 7.754589 | 0.055345 | 1086.517 | 0.418371 | 0.683953793 |  | 1161369554 | 0.06545011 | 2.06077E+19 | 0.085773 | 0.474373206 |  | 0.546924 | 0.271555 | 1.10153 | 0.09397 | 0.253615473 |
| metabolite1208 | 111084.6424 | 365.8213 | 33731765.26 | 0.000122 | 0.00142306 |  | 7.94599 | 0.065621 | 962.1737 | 0.398844 | 0.667920235 |  | 11842419679 | 1.466603145 | 9.56243E+19 | 0.048726 | 0.385659329 |  | 0.409798 | 0.210112 | 0.799262 | 0.010095 | 0.057282501 |
| metabolite1209 | 1457325.151 | 5738.156 | 370118298.8 | 1.95E-06 | 6.78E-05 |  | 0.814537 | 0.006503 | 102.0306 | 0.933814 | 0.974816822 |  | 49408035.24 | 0.004718607 | 5.17346E+17 | 0.13517 | 0.534467195 |  | 0.333866 | 0.172532 | 0.646065 | 0.001494 | 0.016548374 |
| metabolite1210 | 5068.423677 | 11.71829 | 2192206.999 | 0.006866 | 0.029765998 |  | 2.30324 | 0.016516 | 321.2008 | 0.741136 | 0.890859072 |  | 68476905.27 | 0.0038662 | 1.21284E+18 | 0.136826 | 0.535611443 |  | 0.508486 | 0.253677 | 1.019243 | 0.0592 | 0.186496097 |
| metabolite1211 | 8106.998084 | 17.78826 | 3694763.381 | 0.004751 | 0.022177011 |  | 3.009816 | 0.020411 | 443.8269 | 0.666222 | 0.848467311 |  | 123236802.2 | 0.005338631 | 2.84479E+18 | 0.128814 | 0.530263915 |  | 0.482071 | 0.238935 | 0.972616 | 0.043975 | 0.154098261 |
| metabolite1212 | 82270.7048 | 233.8083 | 28948793.56 | 0.000251 | 0.002368385 |  | 1.235433 | 0.009149 | 166.8237 | 0.932832 | 0.974816822 |  | 11308096.34 | 0.000721769 | 1.77166E+17 | 0.177844 | 0.575285157 |  | 0.392935 | 0.199093 | 0.775507 | 0.008181 | 0.050597785 |
| metabolite1213 | 6346.504762 | 14.48837 | 2780030.65 | 0.005665 | 0.025451839 |  | 1.931653 | 0.013591 | 274.5494 | 0.795088 | 0.920287179 |  | 8964213.51 | 0.000440602 | 1.8238E+17 | 0.188914 | 0.589125935 |  | 0.536256 | 0.266372 | 1.07958 | 0.083663 | 0.234874892 |
| metabolite1214 | 835.971661 | 3.378668 | 206841.4583 | 0.01839 | 0.063602752 |  | 0.23007 | 0.002709 | 19.53997 | 0.518082 | 0.753654888 |  | 0.159549856 | 7.57323E-11 | 336133494.1 | 0.867229 | 0.962305681 |  | 0.715094 | 0.379516 | 1.347402 | 0.301764 | 0.515582402 |
| metabolite1215 | 727260.3527 | 2435.554 | 217161130.4 | 9.52E-06 | 0.000204698 |  | 0.287599 | 0.002145 | 38.56432 | 0.619029 | 0.82043448 |  | 48050.94817 | 2.77394E-06 | 8.32351E+14 | 0.37207 | 0.736141064 |  | 0.36943 | 0.187799 | 0.726726 | 0.004708 | 0.03529235 |
| metabolite1216 | 40288.85584 | 107.5482 | 15092691 | 0.000655 | 0.004938059 |  | 4.103583 | 0.030221 | 557.2005 | 0.57425 | 0.791246778 |  | 12597506086 | 0.928855667 | 1.70852E+20 | 0.053241 | 0.394365301 |  | 0.432333 | 0.217719 | 0.858501 | 0.018252 | 0.085304125 |
| metabolite1217 | 25754.9405 | 55.85825 | 11875003.6 | 0.00155 | 0.009539308 |  | 1.406893 | 0.008986 | 220.2597 | 0.894902 | 0.960221533 |  | 1394332.358 | 4.13741E-05 | 4.69899E+16 | 0.255111 | 0.645470675 |  | 0.449386 | 0.221504 | 0.911711 | 0.028726 | 0.118182739 |
| metabolite1218 | 17500.77925 | 35.65879 | 8589110.522 | 0.002526 | 0.01390598 |  | 2.209484 | 0.013709 | 356.1068 | 0.760391 | 0.900309113 |  | 14049307.16 | 0.000377516 | 5.22846E+17 | 0.187792 | 0.586870392 |  | 0.465204 | 0.227971 | 0.949308 | 0.03773 | 0.139457072 |
| metabolite1219 | 1104423.998 | 3903.258 | 312495971.5 | 4.38E-06 | 0.000112937 |  | 1.423989 | 0.010711 | 189.3209 | 0.887594 | 0.95849259 |  | 93563847.18 | 0.00678953 | 1.28937E+18 | 0.126193 | 0.530263915 |  | 0.335192 | 0.171634 | 0.654615 | 0.001788 | 0.018887126 |
| metabolite1220 | 24900.54056 | 34.02185 | 18224668.36 | 0.003253 | 0.016529707 |  | 0.006881 | 3.36E-05 | 1.408218 | 0.069346 | 0.290456173 |  | 0.009045895 | 4.41897E-14 | 1851748269 | 0.72393 | 0.922880405 |  | 0.325792 | 0.154832 | 0.68552 | 0.003821 | 0.03063876 |
| metabolite1221 | 0.059862386 | 0.000174 | 20.56024873 | 0.346636 | 0.519682799 |  | 11.00837 | 0.111231 | 1089.478 | 0.308441 | 0.587722023 |  | 291.4058333 | 6.34421E-08 | 1.3385E+12 | 0.618112 | 0.877846255 |  | 1.637869 | 0.851677 | 3.149801 | 0.142024 | 0.323741911 |
| metabolite1222 | 58050.62611 | 171.1516 | 19689420.26 | 0.000349 | 0.003042443 |  | 0.450321 | 0.003493 | 58.04771 | 0.748204 | 0.893942862 |  | 1313067.559 | 9.90636E-05 | 1.74044E+16 | 0.238672 | 0.629502794 |  | 0.409868 | 0.208624 | 0.805237 | 0.010923 | 0.060479946 |
| metabolite1223 | 16056.54608 | 31.44733 | 8198237.588 | 0.002916 | 0.015218843 |  | 2.390484 | 0.014452 | 395.4056 | 0.738727 | 0.889781026 |  | 2866934.712 | 6.54205E-05 | 1.25638E+17 | 0.236846 | 0.628260432 |  | 0.469306 | 0.229024 | 0.96168 | 0.041084 | 0.147450536 |
| metabolite1224 | 6495.859993 | 11.77049 | 3584914.447 | 0.007463 | 0.031761014 |  | 2.551856 | 0.015067 | 432.2118 | 0.721186 | 0.878058466 |  | 51999232.34 | 0.00112839 | 2.39626E+18 | 0.158925 | 0.563107638 |  | 0.517112 | 0.250645 | 1.066868 | 0.077021 | 0.22225954 |
| metabolite1225 | 0.938411312 | 0.002521 | 349.2960168 | 0.983245 | 0.991851034 |  | 29.30858 | 0.289137 | 2970.882 | 0.154545 | 0.421393324 |  | 0.002991442 | 5.25003E-13 | 17045100.63 | 0.61308 | 0.875727365 |  | 1.211633 | 0.622603 | 2.357931 | 0.573139 | 0.742791887 |
| metabolite1226 | 5045.310438 | 8.889197 | 2863605.825 | 0.009609 | 0.038382879 |  | 0.091388 | 0.000542 | 15.41608 | 0.362439 | 0.635338624 |  | 0.003078393 | 5.16415E-14 | 183505608.6 | 0.64866 | 0.889898952 |  | 0.481016 | 0.233294 | 0.991782 | 0.049911 | 0.168177103 |
| metabolite1227 | 70541.08007 | 153.7313 | 32368440.45 | 0.000528 | 0.004198456 |  | 0.363794 | 0.002235 | 59.20413 | 0.697871 | 0.868760796 |  | 2480.08949 | 5.44553E-08 | 1.12952E+14 | 0.533767 | 0.829370607 |  | 0.399541 | 0.196699 | 0.811558 | 0.012552 | 0.066545486 |
| metabolite1228 | 0.343955087 | 0.000457 | 258.8523028 | 0.752736 | 0.855338201 |  | 0.105804 | 0.000583 | 19.20299 | 0.399133 | 0.668102053 |  | 1.73885E-06 | 2.28762E-17 | 132171.5237 | 0.30175 | 0.68656942 |  | 0.831349 | 0.394427 | 1.752265 | 0.628252 | 0.78315231 |
| metabolite1229 | 0.17895648 | 0.000364 | 87.94630156 | 0.587417 | 0.730439149 |  | 0.230204 | 0.001751 | 30.2695 | 0.556354 | 0.783444637 |  | 1.00109E-06 | 6.64378E-17 | 15084.60786 | 0.250434 | 0.641381034 |  | 0.878764 | 0.436999 | 1.767111 | 0.717591 | 0.845171425 |
| metabolite1230 | 17375.14788 | 32.17617 | 9382589.706 | 0.002939 | 0.015274255 |  | 0.591162 | 0.003409 | 102.5118 | 0.841971 | 0.938160513 |  | 8446.233622 | 1.40145E-07 | 5.09035E+14 | 0.476767 | 0.79982887 |  | 0.488968 | 0.236698 | 1.010105 | 0.055804 | 0.180705406 |
| metabolite1231 | 0.007360239 | 1.97E-05 | 2.744350513 | 0.106822 | 0.23359118 |  | 0.955808 | 0.00854 | 106.9774 | 0.985052 | 0.993620374 |  | 0.222994559 | 2.90778E-11 | 1710121189 | 0.897414 | 0.96870562 |  | 0.965616 | 0.491734 | 1.896177 | 0.91924 | 0.960929034 |
| metabolite1232 | 0.624973308 | 0.00247 | 158.1643075 | 0.868076 | 0.928861972 |  | 2.049478 | 0.026295 | 159.742 | 0.747392 | 0.893259649 |  | 1.30707E-06 | 1.1235E-15 | 1520.63121 | 0.206014 | 0.597241307 |  | 1.242595 | 0.667058 | 2.314707 | 0.495186 | 0.68364345 |
| metabolite1233 | 118.1745447 | 0.467081 | 29898.9058 | 0.093766 | 0.213865932 |  | 14.24493 | 0.177492 | 1143.255 | 0.237648 | 0.518465878 |  | 805072592.8 | 0.643764394 | 1.0068E+18 | 0.057579 | 0.409076168 |  | 0.701079 | 0.374216 | 1.313445 | 0.269932 | 0.479401203 |
| metabolite1234 | 72833.93785 | 162.1403 | 32717230.95 | 0.000489 | 0.003951785 |  | 0.084454 | 0.000536 | 13.31505 | 0.340509 | 0.616004697 |  | 0.829619568 | 1.86998E-11 | 36806203488 | 0.988112 | 0.994828538 |  | 0.408109 | 0.201153 | 0.827992 | 0.014527 | 0.074051084 |
| metabolite1235 | 188480.4114 | 914.4813 | 38847009.59 | 1.91E-05 | 0.000334361 |  | 0.004139 | 4.87E-05 | 0.351534 | 0.017085 | 0.160917476 |  | 2.1537E-05 | 6.65416E-15 | 69707.15722 | 0.338233 | 0.713403042 |  | 0.310578 | 0.167984 | 0.574216 | 0.000304 | 0.005616989 |
| metabolite1236 | 143973.1185 | 290.9116 | 71252778.74 | 0.000281 | 0.002550684 |  | 0.049733 | 0.000287 | 8.627509 | 0.256399 | 0.540622845 |  | 0.085890383 | 1.17191E-12 | 6294973188 | 0.84785 | 0.955623187 |  | 0.373557 | 0.182033 | 0.766591 | 0.008372 | 0.051355574 |
| metabolite1237 | 682827.0573 | 1104.153 | 422271751 | 7.99E-05 | 0.001019159 |  | 1.196456 | 0.005244 | 272.9985 | 0.948496 | 0.977627498 |  | 1879538.558 | 9.03467E-06 | 3.91012E+17 | 0.279612 | 0.663453555 |  | 0.291957 | 0.138987 | 0.613287 | 0.001523 | 0.016710577 |
| metabolite1238 | 0.000989415 | 1.27E-06 | 0.772772483 | 0.044146 | 0.12374939 |  | 55.50783 | 0.280027 | 11002.92 | 0.139498 | 0.402308456 |  | 15097939.19 | 0.000116339 | 1.95934E+18 | 0.208119 | 0.600568725 |  | 2.217825 | 1.048018 | 4.693381 | 0.039583 | 0.143589072 |
| metabolite1239 | 19498.35566 | 39.60227 | 9600103.408 | 0.002282 | 0.012878426 |  | 0.055771 | 0.000353 | 8.816271 | 0.266233 | 0.553495851 |  | 0.003085098 | 6.77696E-14 | 140443858.2 | 0.645192 | 0.888249197 |  | 0.479726 | 0.234589 | 0.98102 | 0.046588 | 0.159491349 |
| metabolite1240 | 31845.12768 | 61.62002 | 16457509.88 | 0.001514 | 0.009412724 |  | 0.037884 | 0.000228 | 6.293229 | 0.212179 | 0.490285108 |  | 0.00145996 | 2.44709E-14 | 87102678.29 | 0.607034 | 0.874215598 |  | 0.443081 | 0.215503 | 0.910988 | 0.028908 | 0.118538643 |
| metabolite1241 | 4.389251009 | 0.016362 | 1177.469582 | 0.605177 | 0.744879168 |  | 30.87296 | 0.393608 | 2421.544 | 0.126147 | 0.381213231 |  | 16837451.32 | 0.012214143 | 2.32108E+16 | 0.124056 | 0.527043366 |  | 1.013072 | 0.539211 | 1.903366 | 0.967875 | 0.983912265 |
| metabolite1242 | 2.359926371 | 0.005574 | 999.197473 | 0.781342 | 0.872572921 |  | 964.5128 | 9.795663 | 94969.07 | 0.004061 | 0.104558924 |  | 2.89324E+13 | 6273.995253 | 1.33421E+23 | 0.007363 | 0.178734331 |  | 0.943721 | 0.477419 | 1.865465 | 0.867981 | 0.937004424 |
| metabolite1243 | 0.01095158 | 2.16E-05 | 5.562180574 | 0.158364 | 0.30752882 |  | 0.2293 | 0.001636 | 32.13856 | 0.560412 | 0.785873824 |  | 431821.7689 | 2.06864E-05 | 9.01415E+15 | 0.286801 | 0.669805045 |  | 1.159029 | 0.571204 | 2.351783 | 0.683476 | 0.82243127 |
| metabolite1244 | 0.017646855 | 3.85E-05 | 8.087864051 | 0.199261 | 0.35636428 |  | 0.113302 | 0.000892 | 14.39131 | 0.380172 | 0.652587742 |  | 172.968405 | 1.14948E-08 | 2.60274E+12 | 0.667309 | 0.900024909 |  | 1.111568 | 0.554702 | 2.227473 | 0.766069 | 0.873631653 |
| metabolite1245 | 3.744378664 | 0.009602 | 1460.092131 | 0.665318 | 0.794399997 |  | 156.9197 | 1.56656 | 15718.38 | 0.033646 | 0.210982668 |  | 3.419886655 | 4.81322E-10 | 24298952159 | 0.915581 | 0.975040351 |  | 1.090986 | 0.556937 | 2.137136 | 0.800086 | 0.896695486 |
| metabolite1246 | 460.6095662 | 1.969568 | 107719.6304 | 0.029621 | 0.09160278 |  | 28.26523 | 0.36701 | 2176.841 | 0.134463 | 0.395398415 |  | 0.000616436 | 4.11516E-13 | 923400.4878 | 0.49432 | 0.808827519 |  | 0.691817 | 0.370687 | 1.291145 | 0.249622 | 0.454315235 |
| metabolite1247 | 21107.6572 | 36.25698 | 12288203.06 | 0.00273 | 0.014591755 |  | 0.022983 | 0.00013 | 4.059252 | 0.155733 | 0.421465998 |  | 3.06489E-05 | 3.78526E-16 | 2481619.907 | 0.419102 | 0.77032628 |  | 0.462407 | 0.222176 | 0.962393 | 0.041494 | 0.148490198 |
| metabolite1248 | 1186.06263 | 0.830905 | 1693026.933 | 0.058712 | 0.151200553 |  | 29.06355 | 0.089854 | 9400.732 | 0.255589 | 0.540322215 |  | 1.33645E+13 | 15.59844187 | 1.14505E+25 | 0.033245 | 0.330758623 |  | 0.529767 | 0.232611 | 1.206534 | 0.133147 | 0.310369395 |
| metabolite1249 | 9271.124086 | 11.40895 | 7533889.848 | 0.008673 | 0.035327915 |  | 0.042706 | 0.000191 | 9.564103 | 0.255847 | 0.540322215 |  | 0.000440343 | 1.8014E-15 | 107639741.7 | 0.564683 | 0.851185759 |  | 0.47264 | 0.219709 | 1.016747 | 0.057744 | 0.184412482 |
| metabolite1250 | 0.038488512 | 9.35E-05 | 15.84265379 | 0.291206 | 0.462217806 |  | 0.090607 | 0.000789 | 10.40464 | 0.323266 | 0.599929612 |  | 1.2604E-07 | 1.58549E-17 | 1001.971878 | 0.174728 | 0.573796732 |  | 0.799338 | 0.404854 | 1.5782 | 0.520051 | 0.700765454 |
| metabolite1251 | 35.9277806 | 0.140302 | 9200.163452 | 0.208215 | 0.369176318 |  | 19.76635 | 0.251828 | 1551.487 | 0.182819 | 0.45686231 |  | 743153.9954 | 0.000525237 | 1.05148E+15 | 0.211201 | 0.603798949 |  | 0.892771 | 0.476063 | 1.674233 | 0.724338 | 0.848711841 |
| metabolite1252 | 9.415775617 | 0.029624 | 2992.753567 | 0.447184 | 0.614043629 |  | 0.222822 | 0.002378 | 20.88083 | 0.518231 | 0.753654888 |  | 532.2374644 | 1.61547E-07 | 1.75353E+12 | 0.575666 | 0.856574192 |  | 0.979566 | 0.511036 | 1.877657 | 0.950524 | 0.975765697 |
| metabolite1253 | 5.382722443 | 0.025089 | 1154.844001 | 0.54013 | 0.691896026 |  | 0.028533 | 0.000435 | 1.869936 | 0.098394 | 0.341151213 |  | 0.077666845 | 1.04631E-10 | 57651761.63 | 0.806748 | 0.9460629 |  | 0.72457 | 0.396554 | 1.32391 | 0.297092 | 0.511306667 |
| metabolite1254 | 2.11310998 | 0.009448 | 472.5992866 | 0.786857 | 0.876086631 |  | 17.36854 | 0.253173 | 1191.541 | 0.188473 | 0.464424653 |  | 586513.7148 | 0.000800831 | 4.29552E+14 | 0.204841 | 0.597241307 |  | 0.892955 | 0.485561 | 1.642157 | 0.716366 | 0.845020783 |
| metabolite1255 | 16.4079843 | 0.076018 | 3541.558819 | 0.309808 | 0.483471921 |  | 0.567919 | 0.008096 | 39.84082 | 0.794667 | 0.920287179 |  | 143768.5057 | 0.000199584 | 1.03562E+14 | 0.256205 | 0.645470675 |  | 0.622717 | 0.341138 | 1.136714 | 0.12576 | 0.3021945 |
| metabolite1256 | 2.44299E-05 | 1.37E-07 | 0.004350555 | 0.000108 | 0.001277598 |  | 826.669 | 12.58856 | 54285.91 | 0.002123 | 0.088493313 |  | 8298570.422 | 0.007229757 | 9.52539E+15 | 0.137271 | 0.536225468 |  | 3.532794 | 1.980168 | 6.302815 | 4.09E-05 | 0.001851283 |
| metabolite1257 | 1.40013E-05 | 8.87E-08 | 0.002209395 | 3.31E-05 | 0.000528986 |  | 6.961563 | 0.095069 | 509.7728 | 0.377646 | 0.650679785 |  | 36.7731168 | 3.48328E-08 | 38821551709 | 0.734463 | 0.926749811 |  | 2.798326 | 1.557632 | 5.027266 | 0.000813 | 0.011267123 |
| metabolite1258 | 142295.7348 | 615.7714 | 32882457.7 | 4.09E-05 | 0.00063014 |  | 2.027875 | 0.019918 | 206.4574 | 0.764941 | 0.902319648 |  | 10365086.35 | 0.002587802 | 4.15159E+16 | 0.154967 | 0.560993738 |  | 0.430896 | 0.226579 | 0.819454 | 0.011584 | 0.063124052 |
| metabolite1259 | 0.056397954 | 0.000282 | 11.29042906 | 0.289876 | 0.461885182 |  | 105.8446 | 1.748089 | 6408.761 | 0.027981 | 0.194800441 |  | 2395012546 | 5.836805406 | 9.82744E+17 | 0.035015 | 0.338247665 |  | 1.590726 | 0.878529 | 2.880279 | 0.128255 | 0.305518079 |
| metabolite1260 | 0.067986036 | 0.000269 | 17.15401533 | 0.342786 | 0.516056608 |  | 1.372249 | 0.017317 | 108.7382 | 0.887454 | 0.95849259 |  | 800468.3068 | 0.000640736 | 1.00002E+15 | 0.206047 | 0.597241307 |  | 1.486436 | 0.798695 | 2.766376 | 0.213658 | 0.410835897 |
| metabolite1261 | 0.390188113 | 0.00107 | 142.2654787 | 0.755089 | 0.856922543 |  | 3.792534 | 0.036611 | 392.8637 | 0.574543 | 0.791246778 |  | 1188764.819 | 0.000253352 | 5.57785E+15 | 0.220858 | 0.613017826 |  | 1.745531 | 0.905245 | 3.365804 | 0.099165 | 0.261157657 |
| metabolite1262 | 0.000921454 | 4.35E-06 | 0.195098451 | 0.011872 | 0.045418827 |  | 21.9778 | 0.29782 | 1621.865 | 0.161909 | 0.430111247 |  | 1746.706866 | 1.47706E-06 | 2.06558E+12 | 0.485133 | 0.802577618 |  | 2.138742 | 1.168586 | 3.914317 | 0.015225 | 0.075699583 |
| metabolite1263 | 1261957.58 | 7484.454 | 212779306.1 | 4.38E-07 | 2.42E-05 |  | 1.322493 | 0.014234 | 122.8704 | 0.903992 | 0.965107208 |  | 2762.355846 | 9.26744E-07 | 8.23378E+12 | 0.478011 | 0.79982887 |  | 0.38922 | 0.208533 | 0.726464 | 0.00372 | 0.030404702 |
| metabolite1264 | 1.03444E-05 | 8.27E-08 | 0.001294646 | 8.90E-06 | 0.000197709 |  | 1.800662 | 0.028195 | 114.9981 | 0.782045 | 0.912186158 |  | 1.33343E-05 | 2.88023E-14 | 6173.263226 | 0.272564 | 0.656510118 |  | 2.480989 | 1.402666 | 4.388291 | 0.002284 | 0.022244058 |
| metabolite1265 | 0.003004731 | 8.07E-06 | 1.118553145 | 0.057054 | 0.148606228 |  | 0.668213 | 0.005852 | 76.29669 | 0.867846 | 0.950093633 |  | 1422.275931 | 1.74499E-07 | 1.15924E+13 | 0.534221 | 0.829370607 |  | 2.052957 | 1.056353 | 3.989798 | 0.036087 | 0.136929681 |
| metabolite1266 | 0.016042228 | 5.8E-05 | 4.440308765 | 0.152568 | 0.301640335 |  | 4.878597 | 0.056438 | 421.7127 | 0.487529 | 0.730703363 |  | 681.4797894 | 3.04628E-07 | 1.52453E+12 | 0.553731 | 0.843207133 |  | 1.637013 | 0.869521 | 3.08194 | 0.129637 | 0.30669642 |
| metabolite1267 | 0.645744346 | 0.002484 | 167.8602614 | 0.877763 | 0.933078715 |  | 1.751602 | 0.021988 | 139.5383 | 0.802312 | 0.922563913 |  | 0.065542615 | 4.40084E-11 | 97614060.93 | 0.800833 | 0.9460629 |  | 1.182406 | 0.632502 | 2.210405 | 0.600687 | 0.763407527 |
| metabolite1268 | 4958.519344 | 30.14668 | 815576.2845 | 0.001441 | 0.009201683 |  | 0.456981 | 0.006822 | 30.61321 | 0.715763 | 0.874720395 |  | 663.0054523 | 1.0517E-06 | 4.17969E+11 | 0.530997 | 0.82747097 |  | 0.600466 | 0.331436 | 1.087871 | 0.095337 | 0.255448028 |
| metabolite1269 | 0.141802222 | 0.000556 | 36.14922727 | 0.491042 | 0.649751017 |  | 0.273246 | 0.003471 | 21.51063 | 0.561455 | 0.78648406 |  | 37195.07012 | 2.80492E-05 | 4.93231E+13 | 0.328247 | 0.705196155 |  | 1.227202 | 0.657323 | 2.291147 | 0.521707 | 0.70197797 |
| metabolite1270 | 72609.5555 | 384.4824 | 13712326.46 | 5.71E-05 | 0.000782649 |  | 0.834767 | 0.009841 | 70.81248 | 0.936608 | 0.975459713 |  | 19734.16883 | 1.0584E-05 | 3.6795E+13 | 0.365791 | 0.732569803 |  | 0.522361 | 0.279982 | 0.974567 | 0.043626 | 0.153312437 |
| metabolite1271 | 0.862068483 | 0.003011 | 246.8360619 | 0.959081 | 0.980990323 |  | 2.416623 | 0.028174 | 207.2853 | 0.698398 | 0.868760796 |  | 29.19721173 | 1.36482E-08 | 62460900586 | 0.758794 | 0.938107312 |  | 0.972821 | 0.514396 | 1.839793 | 0.932608 | 0.96754464 |
| metabolite1272 | 1.89773E-05 | 1.2E-07 | 0.003012157 | 5.30E-05 | 0.000745001 |  | 11.61298 | 0.162051 | 832.2125 | 0.262996 | 0.549846726 |  | 945394.5471 | 0.001102498 | 8.10678E+14 | 0.192536 | 0.592667461 |  | 3.263863 | 1.838788 | 5.79338 | 9.86E-05 | 0.002792026 |
| metabolite1273 | 0.000494892 | 2.05E-06 | 0.119760712 | 0.007626 | 0.032233335 |  | 277.3497 | 3.618163 | 21260.2 | 0.01244 | 0.143313692 |  | 2948019158 | 1.922617066 | 4.52031E+18 | 0.045731 | 0.3810281 |  | 3.080751 | 1.685307 | 5.631633 | 0.000393 | 0.006603083 |
| metabolite1274 | 4.29343E-05 | 2.12E-07 | 0.008688433 | 0.000324 | 0.002872661 |  | 0.568254 | 0.006757 | 47.79241 | 0.803093 | 0.922563913 |  | 1.207921142 | 6.20511E-10 | 2351406169 | 0.986221 | 0.994828538 |  | 2.145679 | 1.156508 | 3.980896 | 0.017094 | 0.081957568 |
| metabolite1275 | 9.688177362 | 0.058162 | 1613.793322 | 0.38612 | 0.555383895 |  | 14.16681 | 0.25647 | 782.5424 | 0.197949 | 0.476694979 |  | 7.486608491 | 2.5514E-08 | 2196802677 | 0.839995 | 0.954234322 |  | 1.038567 | 0.582585 | 1.85144 | 0.898148 | 0.951826138 |
| metabolite1276 | 0.000879792 | 4.77E-06 | 0.162305343 | 0.009404 | 0.037810894 |  | 1.443699 | 0.020898 | 99.73419 | 0.86537 | 0.948991721 |  | 2171.539099 | 3.04036E-06 | 1.551E+12 | 0.461667 | 0.792880411 |  | 1.52819 | 0.838053 | 2.786654 | 0.169259 | 0.360367878 |
| metabolite1277 | 0.001823958 | 9.25E-06 | 0.359756981 | 0.021115 | 0.070912031 |  | 3.098369 | 0.043873 | 218.8108 | 0.603658 | 0.809019269 |  | 14.24800255 | 1.67827E-08 | 12096136794 | 0.800533 | 0.9460629 |  | 1.399286 | 0.762953 | 2.566343 | 0.279974 | 0.491245231 |
| metabolite1278 | 3.47692E-06 | 2.73E-08 | 0.000442621 | 1.52E-06 | 5.69E-05 |  | 195.0712 | 3.161682 | 12035.61 | 0.013613 | 0.149599761 |  | 10373536.22 | 0.017123341 | 6.28442E+15 | 0.120246 | 0.522522999 |  | 3.889328 | 2.2418 | 6.747645 | 4.37E-06 | 0.000337742 |
| metabolite1279 | 0.010323618 | 4.48E-05 | 2.378930215 | 0.102232 | 0.227156714 |  | 0.090336 | 0.00121 | 6.742368 | 0.276906 | 0.563849167 |  | 0.019826309 | 1.65138E-11 | 23803277.82 | 0.713886 | 0.920092212 |  | 1.226807 | 0.660588 | 2.278359 | 0.518828 | 0.699626247 |
| metabolite1280 | 0.059629619 | 0.000289 | 12.31565951 | 0.302101 | 0.474036879 |  | 0.005636 | 9.28E-05 | 0.342221 | 0.014961 | 0.152336933 |  | 4.46238E-05 | 7.06451E-14 | 28187.10832 | 0.334698 | 0.710418618 |  | 0.736505 | 0.403946 | 1.342854 | 0.320442 | 0.533495657 |
| metabolite1281 | 3.181518475 | 0.010386 | 974.5730531 | 0.692677 | 0.812942489 |  | 0.670655 | 0.007373 | 61.0029 | 0.862494 | 0.948212877 |  | 2.914766182 | 1.02995E-09 | 8248796333 | 0.923421 | 0.976740842 |  | 0.566158 | 0.299565 | 1.07 | 0.082593 | 0.23308125 |
| metabolite1282 | 0.007612586 | 2.76E-05 | 2.098541365 | 0.091658 | 0.210562153 |  | 231.9965 | 2.946499 | 18266.55 | 0.016053 | 0.155545176 |  | 686.4499098 | 2.88271E-07 | 1.63462E+12 | 0.554437 | 0.843243794 |  | 2.394843 | 1.288008 | 4.452823 | 0.006767 | 0.044843921 |
| metabolite1283 | 0.585128691 | 0.002447 | 139.9056353 | 0.848258 | 0.916477001 |  | 0.304171 | 0.004095 | 22.59253 | 0.589239 | 0.799804083 |  | 1.38276E-10 | 1.9525E-19 | 0.097927618 | 0.031106 | 0.323868345 |  | 0.782128 | 0.422714 | 1.447136 | 0.435441 | 0.637192795 |
| metabolite1284 | 1.965480171 | 0.009399 | 411.0185177 | 0.804679 | 0.888073767 |  | 0.7133 | 0.010612 | 47.94671 | 0.875242 | 0.953687327 |  | 8.846154535 | 1.3503E-08 | 5795346734 | 0.833681 | 0.953010974 |  | 1.184282 | 0.649189 | 2.160425 | 0.582441 | 0.750760185 |
| metabolite1285 | 4.90365888 | 0.021431 | 1122.006551 | 0.567392 | 0.714728785 |  | 0.016687 | 0.000246 | 1.131042 | 0.059663 | 0.266303606 |  | 2.17721E-09 | 3.20856E-18 | 1.477368425 | 0.05712 | 0.407641201 |  | 0.596099 | 0.325429 | 1.091895 | 0.096688 | 0.256904122 |
| metabolite1286 | 4.1819E-05 | 1.99E-07 | 0.00878289 | 0.000342 | 0.002996814 |  | 247.1951 | 3.213485 | 19015.31 | 0.014376 | 0.149726748 |  | 14260804.47 | 0.007935504 | 2.56279E+16 | 0.132575 | 0.531877651 |  | 2.645523 | 1.434443 | 4.879098 | 0.002339 | 0.022665578 |
| metabolite1287 | 0.725019371 | 0.003266 | 160.9248959 | 0.907341 | 0.951169987 |  | 15.81738 | 0.231725 | 1079.682 | 0.202722 | 0.481010626 |  | 0.013003807 | 1.60864E-11 | 10511899.09 | 0.678962 | 0.903557902 |  | 1.404709 | 0.766859 | 2.573101 | 0.273528 | 0.483363481 |
| metabolite1288 | 1.709939833 | 0.007565 | 386.5153723 | 0.846554 | 0.915906801 |  | 0.946516 | 0.013244 | 67.64434 | 0.979912 | 0.991129599 |  | 0.191945226 | 2.18084E-10 | 168939252 | 0.875471 | 0.965088312 |  | 1.125318 | 0.611261 | 2.071688 | 0.705283 | 0.83693098 |
| metabolite1289 | 0.099965951 | 0.000394 | 25.39461782 | 0.416745 | 0.585628346 |  | 1.353158 | 0.017066 | 107.2932 | 0.892421 | 0.959378477 |  | 8.436620042 | 5.80522E-09 | 12260783832 | 0.84331 | 0.955230753 |  | 0.981157 | 0.524864 | 1.834132 | 0.952582 | 0.976798096 |
| metabolite1290 | 0.400230969 | 0.001598 | 100.2148215 | 0.745818 | 0.84980371 |  | 306.5144 | 4.510831 | 20827.88 | 0.008974 | 0.127105548 |  | 64818840.66 | 0.064726748 | 6.49111E+16 | 0.091724 | 0.484850596 |  | 1.336343 | 0.718829 | 2.484335 | 0.361406 | 0.574570902 |
| metabolite1291 | 0.233167553 | 0.000712 | 76.34921652 | 0.623152 | 0.760696652 |  | 5.328164 | 0.056039 | 506.5993 | 0.473079 | 0.71980137 |  | 227747760.1 | 0.082925769 | 6.25488E+17 | 0.085434 | 0.473204407 |  | 1.127501 | 0.58704 | 2.165538 | 0.719249 | 0.846356465 |
| metabolite1292 | 1.859786223 | 0.00838 | 412.729403 | 0.82231 | 0.899643576 |  | 8.13889 | 0.117602 | 563.2673 | 0.334219 | 0.610077714 |  | 2967.150237 | 3.80222E-06 | 2.31548E+12 | 0.445683 | 0.784097818 |  | 1.129586 | 0.614845 | 2.075259 | 0.695326 | 0.830938413 |
| metabolite1293 | 3.101064665 | 0.013394 | 717.9628112 | 0.684491 | 0.80702296 |  | 0.370965 | 0.005101 | 26.97677 | 0.651134 | 0.837849119 |  | 847688324 | 1.238008307 | 5.80429E+17 | 0.050113 | 0.387346738 |  | 0.683683 | 0.371607 | 1.257838 | 0.224103 | 0.421513294 |
| metabolite1294 | 2.865988752 | 0.013285 | 618.2883179 | 0.701702 | 0.821197452 |  | 0.072928 | 0.001087 | 4.894871 | 0.225056 | 0.506832586 |  | 1585794.972 | 0.002527135 | 9.95098E+14 | 0.169949 | 0.568636202 |  | 0.826046 | 0.451222 | 1.512229 | 0.536899 | 0.710884434 |
| metabolite1295 | 93.92330693 | 0.542334 | 16265.97283 | 0.08689 | 0.202543704 |  | 2.370351 | 0.038899 | 144.4403 | 0.681435 | 0.856641682 |  | 0.59344169 | 1.4302E-09 | 246240708.8 | 0.958987 | 0.987458698 |  | 0.772255 | 0.429639 | 1.38809 | 0.389525 | 0.604322801 |
| metabolite1296 | 1595705.132 | 8974.414 | 283726038 | 3.77E-07 | 2.15E-05 |  | 0.606893 | 0.0062 | 59.40901 | 0.831299 | 0.933701248 |  | 1.97503E-05 | 5.35546E-15 | 72836.79916 | 0.33723 | 0.712676798 |  | 0.284648 | 0.154238 | 0.525321 | 0.000107 | 0.002955697 |
| metabolite1297 | 38850.29995 | 123.893 | 12182658.8 | 0.000472 | 0.003830017 |  | 0.101749 | 0.000867 | 11.93552 | 0.349237 | 0.623385672 |  | 3.743222827 | 3.55073E-10 | 39461508862 | 0.910947 | 0.973093071 |  | 0.439533 | 0.225594 | 0.856356 | 0.017337 | 0.082620345 |
| metabolite1298 | 16.13551847 | 0.054788 | 4752.017144 | 0.339766 | 0.513368609 |  | 401.517 | 5.157431 | 31258.95 | 0.008058 | 0.126652263 |  | 1674466.083 | 0.000751546 | 3.73076E+15 | 0.1946 | 0.593481771 |  | 1.137116 | 0.598039 | 2.16212 | 0.695864 | 0.830938413 |
| metabolite1299 | 0.011101693 | 5.94E-05 | 2.073397103 | 0.094466 | 0.2144111 |  | 1227.219 | 23.50702 | 64068.77 | 0.000617 | 0.07423496 |  | 586.0776038 | 1.107E-06 | 3.10286E+11 | 0.535294 | 0.829425866 |  | 2.549621 | 1.440985 | 4.511196 | 0.001709 | 0.01817719 |
| metabolite1300 | 114.4063662 | 0.625198 | 20935.48592 | 0.077273 | 0.18464916 |  | 0.654442 | 0.010217 | 41.9209 | 0.842028 | 0.938160513 |  | 1.13391E-05 | 2.42973E-14 | 5291.703634 | 0.265929 | 0.650304541 |  | 0.566326 | 0.315294 | 1.017223 | 0.059651 | 0.187596725 |
| metabolite1301 | 2.45912E-05 | 1.23E-07 | 0.004898962 | 0.000149 | 0.001629 |  | 1.202244 | 0.014049 | 102.8809 | 0.935479 | 0.975459713 |  | 23.53997783 | 1.12726E-08 | 49157473564 | 0.773504 | 0.940047404 |  | 2.535766 | 1.374387 | 4.678527 | 0.003567 | 0.029548564 |
| metabolite1302 | 491831.4645 | 2718.98 | 88966522.26 | 2.76E-06 | 8.74E-05 |  | 1.153653 | 0.012551 | 106.0405 | 0.9507 | 0.978382603 |  | 32.47973128 | 1.10355E-08 | 95594079732 | 0.754946 | 0.937628791 |  | 0.464246 | 0.247069 | 0.872325 | 0.018801 | 0.086777083 |
| metabolite1303 | 8.063240581 | 0.036095 | 1801.24995 | 0.45103 | 0.616491367 |  | 1.281174 | 0.017914 | 91.62684 | 0.909653 | 0.96725598 |  | 3935.615399 | 4.70436E-06 | 3.2925E+12 | 0.431379 | 0.777110602 |  | 0.931726 | 0.505906 | 1.715957 | 0.820868 | 0.910367889 |
| metabolite1304 | 3.50862126 | 0.016298 | 755.3234485 | 0.647862 | 0.781350084 |  | 0.296907 | 0.004329 | 20.36318 | 0.574619 | 0.791246778 |  | 8.140733765 | 1.09744E-08 | 6038717025 | 0.840893 | 0.954234322 |  | 0.742683 | 0.406319 | 1.357501 | 0.335775 | 0.548683187 |
| metabolite1305 | 31.65393923 | 0.116613 | 8592.261036 | 0.229465 | 0.392093994 |  | 4.728297 | 0.056213 | 397.7155 | 0.493503 | 0.734908196 |  | 4665514.14 | 0.00278806 | 7.80723E+15 | 0.159245 | 0.563107638 |  | 0.956988 | 0.506993 | 1.806385 | 0.892352 | 0.948858612 |
| metabolite1306 | 0.48935553 | 0.000691 | 346.3633765 | 0.831361 | 0.904745285 |  | 1.006313 | 0.00573 | 176.7431 | 0.9981 | 0.999716475 |  | 2.82915E-05 | 4.52025E-16 | 1770722.098 | 0.410741 | 0.765574693 |  | 1.497731 | 0.717823 | 3.125003 | 0.28404 | 0.496409031 |
| metabolite1307 | 0.000407187 | 1.89E-06 | 0.087519981 | 0.005228 | 0.023834062 |  | 2935.261 | 47.62315 | 180915.3 | 0.000239 | 0.070911374 |  | 830900.7044 | 0.000640696 | 1.07757E+15 | 0.205616 | 0.597241307 |  | 4.031617 | 2.278958 | 7.132179 | 5.19E-06 | 0.000363486 |
| metabolite1308 | 0.00038742 | 2.16E-06 | 0.069619864 | 0.003694 | 0.01827683 |  | 456.6515 | 7.635513 | 27310.62 | 0.004068 | 0.104558924 |  | 107.2148556 | 1.37977E-07 | 83311460602 | 0.65532 | 0.892267662 |  | 2.980745 | 1.681491 | 5.283906 | 0.000294 | 0.0055438 |
| metabolite1309 | 24.94151349 | 0.144066 | 4318.001725 | 0.223844 | 0.386635571 |  | 1.099409 | 0.018482 | 65.3989 | 0.963819 | 0.982173547 |  | 0.051437243 | 1.42406E-10 | 18579253.31 | 0.768424 | 0.940047404 |  | 0.513473 | 0.290051 | 0.908994 | 0.024065 | 0.104086067 |
| metabolite1310 | 3.135771424 | 0.015603 | 630.1969304 | 0.673553 | 0.798239357 |  | 26.10044 | 0.41767 | 1631.03 | 0.12491 | 0.37984995 |  | 8.995768218 | 1.5803E-08 | 5120776476 | 0.83127 | 0.952506813 |  | 1.331981 | 0.734345 | 2.415996 | 0.347417 | 0.559382136 |
| metabolite1311 | 24.45357055 | 0.103537 | 5775.494176 | 0.254015 | 0.420043288 |  | 16.90459 | 0.230197 | 1241.396 | 0.199756 | 0.477707064 |  | 5801667.524 | 0.006047437 | 5.56589E+15 | 0.1428 | 0.542758378 |  | 0.857695 | 0.462081 | 1.592017 | 0.627607 | 0.782874049 |
| metabolite1312 | 1.208059317 | 0.006699 | 217.8610377 | 0.943275 | 0.973441376 |  | 181.7474 | 3.414437 | 9674.252 | 0.011634 | 0.140235238 |  | 891.0374471 | 2.48849E-06 | 3.19049E+11 | 0.500497 | 0.812201491 |  | 1.07486 | 0.598807 | 1.929376 | 0.80933 | 0.901935501 |
| metabolite1313 | 74702.8733 | 372.1803 | 14994128.49 | 6.58E-05 | 0.000863349 |  | 39.92804 | 0.473737 | 3365.259 | 0.105986 | 0.353481876 |  | 3794217.294 | 0.001809156 | 7.95735E+15 | 0.169334 | 0.568636202 |  | 0.483882 | 0.258367 | 0.90624 | 0.025294 | 0.107767194 |
| metabolite1314 | 0.000840109 | 3.21E-06 | 0.219534778 | 0.014107 | 0.052246315 |  | 16.18904 | 0.184591 | 1419.816 | 0.225128 | 0.506832586 |  | 0.000219662 | 8.49633E-14 | 567909.4888 | 0.447814 | 0.785367543 |  | 2.731919 | 1.474739 | 5.060818 | 0.001821 | 0.019102578 |
| metabolite1315 | 0.469525286 | 0.001933 | 114.0733609 | 0.787836 | 0.876134515 |  | 767.455 | 12.14077 | 48513.17 | 0.002165 | 0.088493313 |  | 5.94283E+12 | 10590.63947 | 3.33476E+21 | 0.005038 | 0.153233187 |  | 1.699073 | 0.922257 | 3.130199 | 0.091858 | 0.250465519 |
| metabolite1316 | 0.002913467 | 1.53E-05 | 0.55301992 | 0.031269 | 0.095115382 |  | 2.038159 | 0.030063 | 138.1803 | 0.741278 | 0.890859072 |  | 0.537910638 | 7.78817E-10 | 371522032.1 | 0.952493 | 0.987070684 |  | 1.661157 | 0.915302 | 3.014789 | 0.097943 | 0.258878913 |
| metabolite1317 | 0.01329687 | 6.29E-05 | 2.810525622 | 0.116569 | 0.248757465 |  | 15.31038 | 0.222228 | 1054.808 | 0.209052 | 0.488014005 |  | 24.39947235 | 2.87628E-08 | 20698097091 | 0.761273 | 0.938107312 |  | 1.784158 | 0.978971 | 3.251598 | 0.061286 | 0.190695632 |
| metabolite1318 | 92690.68628 | 407.3183 | 21092996.17 | 7.05E-05 | 0.000917651 |  | 0.859537 | 0.008723 | 84.69312 | 0.948586 | 0.977627498 |  | 8.87369E-05 | 2.28577E-14 | 344489.5751 | 0.409332 | 0.764099599 |  | 0.561239 | 0.29364 | 1.072705 | 0.08329 | 0.234514727 |
| metabolite1319 | 0.544445875 | 0.001034 | 286.7359692 | 0.84953 | 0.917261722 |  | 8.593188 | 0.062778 | 1176.26 | 0.393268 | 0.664744335 |  | 47154206099 | 3.346747929 | 6.64382E+20 | 0.041609 | 0.365902327 |  | 1.268891 | 0.627228 | 2.566981 | 0.509051 | 0.694175389 |
| metabolite1320 | 0.367059525 | 0.001107 | 121.6747228 | 0.735644 | 0.844670365 |  | 23.9157 | 0.256781 | 2227.427 | 0.172738 | 0.443346793 |  | 0.103583764 | 2.73116E-11 | 392858215.9 | 0.840687 | 0.954234322 |  | 1.351886 | 0.704587 | 2.593852 | 0.366452 | 0.581155915 |
| metabolite1321 | 9.376944022 | 0.013045 | 6740.20619 | 0.506183 | 0.661191548 |  | 12.68656 | 0.07224 | 2227.969 | 0.33741 | 0.613183543 |  | 4.49257E+12 | 108.5719211 | 1.85897E+23 | 0.021297 | 0.283005659 |  | 0.902215 | 0.42954 | 1.895028 | 0.786304 | 0.887732263 |
| metabolite1322 | 13.60015375 | 0.040011 | 4622.780307 | 0.382008 | 0.551179285 |  | 8.747434 | 0.088993 | 859.8155 | 0.356193 | 0.629200512 |  | 75101155851 | 27.52053658 | 2.04945E+20 | 0.025834 | 0.298662816 |  | 0.891174 | 0.461311 | 1.721596 | 0.732284 | 0.853756458 |
| metabolite1323 | 8.42631E-05 | 5.16E-07 | 0.013750092 | 0.000462 | 0.003765842 |  | 0.693007 | 0.009982 | 48.11192 | 0.865705 | 0.949078292 |  | 2.154754661 | 2.80337E-09 | 1656207905 | 0.941508 | 0.982270929 |  | 1.544312 | 0.84651 | 2.817332 | 0.159353 | 0.346431072 |
| metabolite1324 | 1.963522882 | 0.005259 | 733.0708237 | 0.823713 | 0.900487952 |  | 3.355014 | 0.031784 | 354.1486 | 0.611623 | 0.814985059 |  | 0.003826474 | 6.59151E-13 | 22213286.89 | 0.628468 | 0.879305472 |  | 1.21239 | 0.622692 | 2.360544 | 0.572171 | 0.741902754 |
| metabolite1325 | 35.38039776 | 0.164485 | 7610.24532 | 0.195833 | 0.353104799 |  | 0.190938 | 0.002722 | 13.39167 | 0.446761 | 0.699880306 |  | 0.050657293 | 6.00326E-11 | 42746135.94 | 0.77661 | 0.940047404 |  | 0.786433 | 0.428183 | 1.444422 | 0.44026 | 0.638204408 |
| metabolite1326 | 15857.4942 | 68.98966 | 3644895.539 | 0.000703 | 0.005237574 |  | 1.115163 | 0.012266 | 101.3889 | 0.962303 | 0.981849859 |  | 5.78404E-05 | 2.21381E-14 | 151119.804 | 0.379674 | 0.739616988 |  | 0.557278 | 0.295056 | 1.052539 | 0.074232 | 0.217939704 |
| metabolite1327 | 976.4742698 | 4.466006 | 213502.1752 | 0.013718 | 0.051008717 |  | 2.273363 | 0.029112 | 177.5275 | 0.712555 | 0.873627959 |  | 59.30277529 | 4.38714E-08 | 80162079872 | 0.704227 | 0.912813656 |  | 0.463666 | 0.252654 | 0.85091 | 0.014594 | 0.0741885 |
| metabolite1328 | 0.052911151 | 0.000233 | 12.01412251 | 0.290613 | 0.462181116 |  | 81.86022 | 1.209523 | 5540.277 | 0.042874 | 0.236060993 |  | 14458.45026 | 1.57271E-05 | 1.32921E+13 | 0.364965 | 0.731310396 |  | 1.904646 | 1.042803 | 3.478775 | 0.038321 | 0.140547174 |
| metabolite1329 | 0.070542854 | 0.000257 | 19.34568953 | 0.356599 | 0.528911944 |  | 103.7933 | 1.33575 | 8065.172 | 0.03887 | 0.223736893 |  | 15296332.16 | 0.00960515 | 2.43596E+16 | 0.128792 | 0.530263915 |  | 1.536244 | 0.818393 | 2.883756 | 0.184201 | 0.379971156 |
| metabolite1330 | 0.065796304 | 0.000312 | 13.88062714 | 0.321122 | 0.494168131 |  | 342.833 | 5.731227 | 20507.73 | 0.006092 | 0.118151077 |  | 2918148311 | 5.916914229 | 1.43919E+18 | 0.035037 | 0.338247665 |  | 2.197106 | 1.220927 | 3.953781 | 0.009862 | 0.056302052 |
| metabolite1331 | 9.474136772 | 0.046138 | 1945.461492 | 0.409622 | 0.578207909 |  | 22.47268 | 0.348722 | 1448.206 | 0.145928 | 0.411005424 |  | 497454.5332 | 0.000887251 | 2.78907E+14 | 0.204527 | 0.597241307 |  | 1.213239 | 0.665477 | 2.211872 | 0.529433 | 0.707540497 |
| metabolite1332 | 0.066889421 | 0.000259 | 17.24853678 | 0.341775 | 0.515160589 |  | 892.9947 | 13.33407 | 59804.64 | 0.001985 | 0.088493313 |  | 4185617620 | 3.968920642 | 4.41415E+18 | 0.038898 | 0.35379826 |  | 1.487872 | 0.797497 | 2.775889 | 0.214355 | 0.410835897 |
| metabolite1333 | 1.220216109 | 0.003224 | 461.8381865 | 0.947723 | 0.9750487 |  | 0.866488 | 0.008085 | 92.86399 | 0.952193 | 0.978833321 |  | 0.000120299 | 2.0545E-14 | 704399.0615 | 0.43322 | 0.779282769 |  | 0.897448 | 0.459985 | 1.750957 | 0.751612 | 0.864342098 |
| metabolite1334 | 1.461000622 | 0.007403 | 288.3203277 | 0.888439 | 0.939582748 |  | 7.94703 | 0.126024 | 501.1378 | 0.329041 | 0.605724591 |  | 1.6294E-06 | 3.61373E-15 | 734.6853901 | 0.192605 | 0.592667461 |  | 1.03499 | 0.570682 | 1.877062 | 0.910052 | 0.957528306 |
| metabolite1335 | 9.05345E-05 | 5.34E-07 | 0.015352611 | 0.000557 | 0.004365827 |  | 3.918273 | 0.055418 | 277.0384 | 0.530936 | 0.764725841 |  | 0.83953673 | 9.66362E-10 | 729356372.7 | 0.986741 | 0.994828538 |  | 2.207128 | 1.220783 | 3.990402 | 0.010016 | 0.056922431 |
| metabolite1336 | 0.228385848 | 0.001082 | 48.19720898 | 0.58973 | 0.731935183 |  | 13.97956 | 0.211452 | 924.218 | 0.220025 | 0.500900006 |  | 33306.47224 | 5.25209E-05 | 2.11215E+13 | 0.316106 | 0.69992255 |  | 1.156246 | 0.632634 | 2.113233 | 0.637955 | 0.789940178 |
| metabolite1337 | 396029120.4 | 3687778 | 42529422565 | 2.81E-13 | 1.04E-09 |  | 0.045991 | 0.000439 | 4.820558 | 0.197207 | 0.476003291 |  | 1.13305E-09 | 2.36953E-19 | 5.417993498 | 0.072782 | 0.447917707 |  | 0.165514 | 0.092576 | 0.29592 | 1.84E-08 | 2.59E-05 |
| metabolite1338 | 141779.7212 | 653.3813 | 30765327.4 | 3.39E-05 | 0.000539276 |  | 0.083493 | 0.000877 | 7.945031 | 0.287705 | 0.57368832 |  | 0.000203023 | 5.46742E-14 | 753886.2097 | 0.451096 | 0.787987411 |  | 0.439273 | 0.232354 | 0.830458 | 0.012749 | 0.067051153 |
| metabolite1339 | 2397.485251 | 14.92652 | 385082.0352 | 0.003302 | 0.016650375 |  | 0.01313 | 0.000222 | 0.776489 | 0.039685 | 0.225876579 |  | 6.02733E-08 | 1.47973E-16 | 24.5508867 | 0.103097 | 0.49665678 |  | 0.434801 | 0.244822 | 0.772203 | 0.005334 | 0.038212009 |
| metabolite1340 | 0.016494542 | 7.25E-05 | 3.754946677 | 0.141112 | 0.284601189 |  | 1.909197 | 0.025532 | 142.7646 | 0.769478 | 0.90505024 |  | 4.50595E-06 | 4.62085E-15 | 4393.910186 | 0.246233 | 0.639636914 |  | 1.572366 | 0.852946 | 2.898582 | 0.149796 | 0.333698345 |
| metabolite1341 | 0.054143059 | 0.000207 | 14.15741233 | 0.306742 | 0.479291958 |  | 67.9017 | 0.890969 | 5174.859 | 0.059003 | 0.265670403 |  | 614709.8685 | 0.000420587 | 8.98431E+14 | 0.218338 | 0.609212564 |  | 1.763758 | 0.9478 | 3.28217 | 0.07605 | 0.220605979 |
| metabolite1342 | 583.4160184 | 1.965085 | 173210.9223 | 0.030426 | 0.093392243 |  | 0.108759 | 0.001137 | 10.40212 | 0.342415 | 0.617782379 |  | 0.078966034 | 2.0159E-11 | 309322666.9 | 0.822183 | 0.948494237 |  | 0.415576 | 0.220373 | 0.783687 | 0.007728 | 0.048774613 |
| metabolite1343 | 1.490618689 | 0.010777 | 206.182949 | 0.874179 | 0.931621453 |  | 0.426446 | 0.008815 | 20.62915 | 0.667564 | 0.849855716 |  | 0.00417837 | 3.15116E-11 | 554043.2835 | 0.567088 | 0.851903545 |  | 0.924062 | 0.530406 | 1.60988 | 0.780887 | 0.884576365 |
| metabolite1344 | 1264.967764 | 8.992743 | 177937.1831 | 0.005521 | 0.024895134 |  | 0.057179 | 0.00105 | 3.115088 | 0.163428 | 0.431469419 |  | 6.43095E-06 | 2.58749E-14 | 1598.348191 | 0.228061 | 0.620933678 |  | 0.471307 | 0.2693 | 0.824844 | 0.009634 | 0.055732714 |
| metabolite1345 | 0.570215554 | 0.001637 | 198.5802192 | 0.851132 | 0.917673295 |  | 7.043565 | 0.071136 | 697.4229 | 0.406844 | 0.67470416 |  | 4.05272E-05 | 9.63051E-15 | 170547.2308 | 0.372987 | 0.736141064 |  | 0.89248 | 0.461722 | 1.725106 | 0.735776 | 0.856713706 |
| metabolite1346 | 0.026900285 | 9.06E-05 | 7.989941525 | 0.215893 | 0.378272222 |  | 3.81108 | 0.042005 | 345.7744 | 0.561937 | 0.78648406 |  | 108587.7002 | 4.19118E-05 | 2.81336E+14 | 0.296679 | 0.680236719 |  | 1.179924 | 0.619015 | 2.24909 | 0.616169 | 0.774675165 |
| metabolite1347 | 1.113239526 | 0.00388 | 319.406417 | 0.97043 | 0.984976957 |  | 125.8642 | 1.600949 | 9895.259 | 0.032035 | 0.206393448 |  | 821367.9043 | 0.000438554 | 1.53834E+15 | 0.213856 | 0.604329586 |  | 1.21143 | 0.641046 | 2.289325 | 0.555948 | 0.726886063 |
| metabolite1348 | 0.000669732 | 2.77E-06 | 0.161987492 | 0.010302 | 0.040585795 |  | 457.9535 | 6.188332 | 33889.82 | 0.006205 | 0.118151077 |  | 1758047461 | 1.189815374 | 2.59766E+18 | 0.050621 | 0.388929664 |  | 2.652799 | 1.440691 | 4.884702 | 0.002219 | 0.021821825 |
| metabolite1349 | 0.000216443 | 9.81E-07 | 0.047767131 | 0.002737 | 0.014591755 |  | 77.93287 | 1.004884 | 6044.013 | 0.052243 | 0.255419086 |  | 4.073246596 | 2.17302E-09 | 7635152189 | 0.897652 | 0.96870562 |  | 2.746113 | 1.500257 | 5.026563 | 0.001409 | 0.016109823 |
| metabolite1350 | 0.043593001 | 0.000209 | 9.075289293 | 0.252521 | 0.419001144 |  | 0.347747 | 0.00509 | 23.75623 | 0.62502 | 0.823083204 |  | 0.034692277 | 4.82856E-11 | 24925722.11 | 0.747258 | 0.933686356 |  | 1.050245 | 0.573613 | 1.922925 | 0.874062 | 0.939114565 |
| metabolite1351 | 237.7092547 | 2.135915 | 26455.02687 | 0.024786 | 0.080333391 |  | 0.030179 | 0.000717 | 1.270299 | 0.069241 | 0.290456173 |  | 0.002850703 | 3.27213E-11 | 248355.3689 | 0.531138 | 0.82747097 |  | 0.662027 | 0.386734 | 1.133284 | 0.135471 | 0.313424676 |
| metabolite1352 | 185.5424856 | 1.028752 | 33463.85731 | 0.051248 | 0.137614239 |  | 0.040195 | 0.000654 | 2.470688 | 0.128973 | 0.3859835 |  | 9.65138E-05 | 1.97914E-13 | 47065.37065 | 0.366973 | 0.733320142 |  | 0.438177 | 0.246521 | 0.778834 | 0.005827 | 0.040722674 |
| metabolite1353 | 2.679582521 | 0.01311 | 547.6912136 | 0.717195 | 0.831202343 |  | 13.03399 | 0.202528 | 838.8215 | 0.229451 | 0.511407568 |  | 48488.85406 | 8.82779E-05 | 2.66337E+13 | 0.295628 | 0.67904734 |  | 1.205596 | 0.662574 | 2.19366 | 0.541648 | 0.713960117 |
| metabolite1354 | 7.01753E-05 | 3.92E-07 | 0.012548997 | 0.000454 | 0.00373414 |  | 380.7562 | 5.86145 | 24733.69 | 0.006198 | 0.118151077 |  | 4520497.13 | 0.0049349 | 4.14089E+15 | 0.14835 | 0.548173861 |  | 3.151894 | 1.765588 | 5.626703 | 0.000176 | 0.004028754 |
| metabolite1355 | 0.000144024 | 5.5E-07 | 0.037744089 | 0.002353 | 0.013149818 |  | 96.17108 | 1.076051 | 8595.201 | 0.048829 | 0.248657497 |  | 314250.0702 | 9.35811E-05 | 1.05527E+15 | 0.260464 | 0.647529078 |  | 2.744057 | 1.466552 | 5.13439 | 0.002047 | 0.020570116 |
| metabolite1356 | 31.48664634 | 0.035101 | 28244.37709 | 0.322181 | 0.495283952 |  | 0.205487 | 0.000957 | 44.13592 | 0.564711 | 0.787805088 |  | 57.48032345 | 3.13405E-10 | 1.05422E+13 | 0.760039 | 0.938107312 |  | 0.540336 | 0.252525 | 1.156175 | 0.115565 | 0.287697033 |
| metabolite1357 | 0.00238039 | 9.87E-06 | 0.57415945 | 0.033063 | 0.099113234 |  | 114.9682 | 1.528908 | 8645.181 | 0.033514 | 0.210821857 |  | 0.042193752 | 2.45282E-11 | 72582388.78 | 0.77102 | 0.940047404 |  | 2.50692 | 1.366068 | 4.600541 | 0.003683 | 0.030171356 |
| metabolite1358 | 58.05119968 | 0.226585 | 14872.7251 | 0.154009 | 0.303517081 |  | 0.002724 | 3.82E-05 | 0.19452 | 0.007757 | 0.126652263 |  | 0.346401681 | 2.01729E-10 | 594829709.3 | 0.922331 | 0.976632536 |  | 0.607743 | 0.32575 | 1.133852 | 0.120386 | 0.295276714 |
| metabolite1359 | 0.255674185 | 0.000968 | 67.55232314 | 0.632641 | 0.767985687 |  | 0.027531 | 0.000357 | 2.122114 | 0.107945 | 0.354968411 |  | 0.000363054 | 2.35081E-13 | 560692.7766 | 0.464634 | 0.792880411 |  | 0.751099 | 0.401409 | 1.405423 | 0.372537 | 0.587791572 |
| metabolite1360 | 7.87739E-05 | 4.73E-07 | 0.013114343 | 0.000444 | 0.00366257 |  | 64.87106 | 0.984938 | 4272.608 | 0.053349 | 0.25621721 |  | 2208.765777 | 2.76673E-06 | 1.76332E+12 | 0.463111 | 0.792880411 |  | 2.892146 | 1.625328 | 5.14635 | 0.000458 | 0.007356291 |
| metabolite1361 | 82.57738029 | 0.255604 | 26678.088 | 0.137166 | 0.279528667 |  | 0.069888 | 0.000725 | 6.738702 | 0.256111 | 0.540322215 |  | 0.012025702 | 2.86836E-12 | 50418267.7 | 0.696501 | 0.907533437 |  | 0.552582 | 0.289042 | 1.056409 | 0.075532 | 0.219496962 |
| metabolite1362 | 0.000267008 | 1.24E-06 | 0.057317682 | 0.003296 | 0.016647984 |  | 44.44452 | 0.579807 | 3406.847 | 0.089356 | 0.324973836 |  | 1764.489878 | 1.13394E-06 | 2.74566E+12 | 0.490213 | 0.806374128 |  | 2.642973 | 1.447133 | 4.826996 | 0.002017 | 0.020468032 |
| metabolite1363 | 1.674569837 | 0.00502 | 558.5776639 | 0.862239 | 0.925000983 |  | 0.489049 | 0.005047 | 47.38613 | 0.759772 | 0.900227717 |  | 2.22643E-05 | 6.3005E-15 | 78676.34837 | 0.341647 | 0.713874057 |  | 0.758765 | 0.395124 | 1.457071 | 0.408738 | 0.61684686 |
| metabolite1364 | 92802.65162 | 485.8004 | 17728129.95 | 4.17E-05 | 0.000636226 |  | 29.16767 | 0.351643 | 2419.365 | 0.137399 | 0.400854765 |  | 5799.327993 | 2.74826E-06 | 1.22376E+13 | 0.430588 | 0.777110602 |  | 0.628808 | 0.334068 | 1.183589 | 0.153339 | 0.337510259 |
| metabolite1365 | 0.001239983 | 4.55E-06 | 0.337880685 | 0.021111 | 0.070912031 |  | 0.948453 | 0.010295 | 87.38121 | 0.981745 | 0.992172335 |  | 1.48141351 | 4.93205E-10 | 4449644096 | 0.971907 | 0.990751288 |  | 1.845506 | 0.976142 | 3.489136 | 0.061951 | 0.191903823 |
| metabolite1366 | 144.3333993 | 0.93981 | 22166.32508 | 0.055432 | 0.145375886 |  | 0.193957 | 0.003486 | 10.7924 | 0.425497 | 0.688925767 |  | 279.0465055 | 1.02942E-06 | 75641864290 | 0.570899 | 0.853748966 |  | 0.638479 | 0.360906 | 1.129534 | 0.126039 | 0.302542687 |
| metabolite1367 | 0.013063284 | 2.13E-05 | 8.004211676 | 0.187962 | 0.34377926 |  | 81.66835 | 0.535469 | 12455.84 | 0.088864 | 0.324630307 |  | 333304.3291 | 7.93653E-06 | 1.39975E+16 | 0.310431 | 0.694400821 |  | 1.68652 | 0.819203 | 3.472091 | 0.158789 | 0.345610269 |
| metabolite1368 | 839.8340305 | 2.197986 | 320894.25 | 0.028478 | 0.088808014 |  | 98.53278 | 0.889186 | 10918.64 | 0.058571 | 0.264422304 |  | 1255803.287 | 0.000137341 | 1.14826E+16 | 0.232673 | 0.625185612 |  | 0.618343 | 0.313717 | 1.218769 | 0.167754 | 0.358603931 |
| metabolite1369 | 0.358461465 | 0.001628 | 78.92986211 | 0.710041 | 0.826440128 |  | 271.1552 | 4.401452 | 16704.75 | 0.00885 | 0.126652263 |  | 13745827.07 | 0.021448063 | 8.80955E+15 | 0.114986 | 0.516670981 |  | 1.407784 | 0.769002 | 2.577176 | 0.269994 | 0.479401203 |
| metabolite1370 | 0.020122878 | 6.18E-05 | 6.556184787 | 0.188539 | 0.344324076 |  | 52.07838 | 0.559795 | 4844.908 | 0.090221 | 0.326006724 |  | 3284270.611 | 0.000935137 | 1.15346E+16 | 0.183622 | 0.5811057 |  | 1.508041 | 0.785357 | 2.895736 | 0.219755 | 0.416989176 |
| metabolite1371 | 0.013592157 | 5.57E-05 | 3.313946763 | 0.128183 | 0.266194087 |  | 26.77387 | 0.352295 | 2034.775 | 0.139632 | 0.402308456 |  | 1156.919872 | 8.2538E-07 | 1.62163E+12 | 0.51291 | 0.819395994 |  | 1.390489 | 0.746049 | 2.591598 | 0.301641 | 0.515582402 |
| metabolite1372 | 0.218610559 | 0.001188 | 40.21954978 | 0.568837 | 0.715372941 |  | 0.101167 | 0.001693 | 6.045385 | 0.274665 | 0.563632158 |  | 1.7260688 | 4.17076E-09 | 714333087.8 | 0.957095 | 0.9871034 |  | 0.846996 | 0.470712 | 1.524078 | 0.580659 | 0.749244321 |
| metabolite1373 | 0.000168316 | 8.28E-07 | 0.034230742 | 0.001767 | 0.010576258 |  | 23.94947 | 0.313931 | 1827.081 | 0.153784 | 0.421028033 |  | 0.121838842 | 8.33748E-11 | 178047887 | 0.845346 | 0.955623187 |  | 2.413344 | 1.31929 | 4.41467 | 0.005073 | 0.037278103 |
| metabolite1374 | 0.003012552 | 9.03E-06 | 1.005342143 | 0.052715 | 0.140435775 |  | 6.303931 | 0.060819 | 653.4092 | 0.43848 | 0.696199328 |  | 0.735873107 | 1.30562E-10 | 4147521596 | 0.978689 | 0.991698663 |  | 2.824557 | 1.493787 | 5.340869 | 0.001822 | 0.019102578 |
| metabolite1375 | 2.72121E-06 | 1.62E-08 | 0.000456271 | 3.24E-06 | 9.32E-05 |  | 20.38722 | 0.247002 | 1682.737 | 0.183317 | 0.457170747 |  | 1696.815015 | 8.45878E-07 | 3.40378E+12 | 0.497615 | 0.811271809 |  | 3.266248 | 1.79783 | 5.93403 | 0.000174 | 0.004011164 |
| metabolite1376 | 0.074828675 | 0.000307 | 18.26038482 | 0.35731 | 0.529755824 |  | 11.14267 | 0.147864 | 839.6821 | 0.276667 | 0.563849167 |  | 0.469242686 | 3.68453E-10 | 597602565.5 | 0.943734 | 0.983763595 |  | 1.720479 | 0.931705 | 3.177024 | 0.085704 | 0.239494596 |
| metabolite1377 | 0.008059946 | 2.74E-05 | 2.374632139 | 0.099361 | 0.222667813 |  | 45.29212 | 0.515023 | 3983.077 | 0.097839 | 0.340181253 |  | 4.670423806 | 1.48917E-09 | 14647647894 | 0.890371 | 0.967390372 |  | 1.824641 | 0.963439 | 3.455656 | 0.067602 | 0.203654718 |
| metabolite1378 | 12.18010568 | 0.056631 | 2619.685861 | 0.363622 | 0.534840548 |  | 105.115 | 1.647273 | 6707.544 | 0.030217 | 0.202044304 |  | 115364.1094 | 0.000164774 | 8.07706E+13 | 0.26441 | 0.650304541 |  | 1.070052 | 0.583089 | 1.9637 | 0.827377 | 0.914080058 |
| metabolite1379 | 114.9027623 | 0.455775 | 28967.48405 | 0.095478 | 0.216048496 |  | 1.002045 | 0.012185 | 82.40181 | 0.999277 | 0.999753507 |  | 0.001547305 | 9.22174E-13 | 2596204.498 | 0.551633 | 0.842085888 |  | 0.754947 | 0.402644 | 1.415503 | 0.382643 | 0.597135336 |
| metabolite1380 | 16.97471165 | 0.059662 | 4829.565956 | 0.328139 | 0.501740826 |  | 0.01946 | 0.000237 | 1.598547 | 0.082632 | 0.313545344 |  | 18.62242457 | 8.10852E-09 | 42769152404 | 0.790797 | 0.944051932 |  | 0.591105 | 0.314271 | 1.111796 | 0.105682 | 0.271220918 |
| metabolite1381 | 7.42235E-05 | 5.29E-07 | 0.010418116 | 0.000264 | 0.002449115 |  | 3.255781 | 0.052407 | 202.2646 | 0.57639 | 0.791336134 |  | 72.91233632 | 1.60515E-07 | 33119672999 | 0.674033 | 0.902621002 |  | 2.054966 | 1.155057 | 3.655999 | 0.015831 | 0.077503408 |
| metabolite1382 | 0.001528453 | 5.69E-06 | 0.410692891 | 0.025024 | 0.080822448 |  | 51.06245 | 0.598675 | 4355.242 | 0.085724 | 0.319399245 |  | 6.865057008 | 2.49186E-09 | 18913152271 | 0.862411 | 0.96050692 |  | 2.787331 | 1.505911 | 5.159144 | 0.001464 | 0.016315002 |
| metabolite1383 | 26.4653728 | 0.09497 | 7375.133125 | 0.256563 | 0.42297012 |  | 12.59443 | 0.149417 | 1061.593 | 0.265247 | 0.552487671 |  | 0.069232606 | 3.156E-11 | 151874257.8 | 0.808199 | 0.9460629 |  | 1.001856 | 0.529397 | 1.89596 | 0.995464 | 0.99680745 |
| metabolite1384 | 7.002350699 | 0.020674 | 2371.705979 | 0.513912 | 0.667995766 |  | 37.97363 | 0.402987 | 3578.276 | 0.119698 | 0.372764958 |  | 18522.14285 | 4.68422E-06 | 7.32394E+13 | 0.385315 | 0.743660185 |  | 0.984445 | 0.510136 | 1.899754 | 0.962804 | 0.98212409 |
| metabolite1385 | 1.826654988 | 0.007703 | 433.1670514 | 0.829434 | 0.903708325 |  | 22.61767 | 0.316898 | 1614.269 | 0.154882 | 0.421465998 |  | 8890797.35 | 0.010399398 | 7.60104E+15 | 0.130139 | 0.530263915 |  | 1.05185 | 0.568079 | 1.947595 | 0.872519 | 0.939114565 |
| metabolite1386 | 0.000460736 | 2.04E-06 | 0.104289672 | 0.006439 | 0.02818149 |  | 0.195354 | 0.002386 | 15.99144 | 0.469015 | 0.717917212 |  | 0.034174656 | 1.93114E-11 | 60477707.79 | 0.756561 | 0.937628791 |  | 1.59707 | 0.854359 | 2.985431 | 0.145248 | 0.327868785 |
| metabolite1387 | 25863.60605 | 160.0159 | 4180372.326 | 0.000156 | 0.001656261 |  | 38.52306 | 0.567511 | 2614.972 | 0.092547 | 0.329911989 |  | 35.4397069 | 4.00501E-08 | 31360053752 | 0.734914 | 0.926749811 |  | 0.501438 | 0.275896 | 0.911359 | 0.025486 | 0.108042815 |
| metabolite1388 | 4938.673113 | 19.79731 | 1232010.187 | 0.003135 | 0.016069559 |  | 0.385342 | 0.004206 | 35.30781 | 0.679873 | 0.855547102 |  | 7.56251E-05 | 2.73048E-14 | 209455.6572 | 0.394128 | 0.75353341 |  | 0.465348 | 0.247622 | 0.874515 | 0.019187 | 0.087794436 |
| metabolite1389 | 0.003040957 | 1.13E-05 | 0.815362545 | 0.044588 | 0.124693862 |  | 149.0583 | 1.855147 | 11976.62 | 0.027343 | 0.19379821 |  | 2.989268248 | 1.20302E-09 | 7427773311 | 0.92115 | 0.976632536 |  | 2.641109 | 1.427246 | 4.887353 | 0.002507 | 0.023675386 |
| metabolite1390 | 0.000154469 | 8.94E-07 | 0.026702002 | 0.001148 | 0.007678553 |  | 0.438952 | 0.00624 | 30.87617 | 0.705102 | 0.870776373 |  | 39.45306588 | 4.81951E-08 | 32296761920 | 0.726268 | 0.924067425 |  | 1.80671 | 0.992747 | 3.288047 | 0.055389 | 0.180128249 |
| metabolite1391 | 62.49713668 | 0.227955 | 17134.50464 | 0.151625 | 0.299936297 |  | 0.107398 | 0.001264 | 9.127232 | 0.32706 | 0.604742395 |  | 3.41825E-11 | 2.45324E-20 | 0.047628603 | 0.026856 | 0.301331155 |  | 0.458188 | 0.246109 | 0.853022 | 0.015382 | 0.075990842 |
| metabolite1392 | 27580796.35 | 148927.6 | 5107854100 | 3.29E-09 | 7.64E-07 |  | 1.271609 | 0.01029 | 157.1423 | 0.92229 | 0.972887284 |  | 1765.12005 | 1.48394E-07 | 2.09958E+13 | 0.528942 | 0.825510246 |  | 0.290296 | 0.151623 | 0.555798 | 0.000301 | 0.005616989 |
| metabolite1393 | 91979.20371 | 313.2982 | 27003579.88 | 0.000142 | 0.001577111 |  | 0.08252 | 0.000711 | 9.58391 | 0.306017 | 0.586221996 |  | 1.65749E-05 | 1.77307E-15 | 154943.8691 | 0.349392 | 0.717869012 |  | 0.365026 | 0.189201 | 0.704247 | 0.003276 | 0.028017373 |
| metabolite1394 | 0.068574903 | 0.000312 | 15.07073902 | 0.332166 | 0.505399449 |  | 0.279564 | 0.003956 | 19.75399 | 0.558601 | 0.784917999 |  | 24.21622597 | 2.83306E-08 | 20699345145 | 0.761904 | 0.938107312 |  | 1.100911 | 0.598331 | 2.025643 | 0.757877 | 0.867603587 |
| metabolite1395 | 0.346228821 | 0.001306 | 91.78595106 | 0.710192 | 0.826440128 |  | 20.15286 | 0.257227 | 1578.906 | 0.179835 | 0.455229213 |  | 254.4180217 | 1.59819E-07 | 4.0501E+11 | 0.609404 | 0.874215598 |  | 1.447998 | 0.774937 | 2.705637 | 0.248294 | 0.453008272 |
| metabolite1396 | 2410288.002 | 4192.728 | 1385610659 | 1.48E-05 | 0.000283824 |  | 12.07554 | 0.053074 | 2747.464 | 0.370245 | 0.644917822 |  | 2.69947E+12 | 17.97526566 | 4.05398E+23 | 0.031367 | 0.323868345 |  | 0.340732 | 0.160472 | 0.723483 | 0.005985 | 0.041099553 |
| metabolite1397 | 3.73983E-06 | 2.56E-08 | 0.000545774 | 3.09E-06 | 9.02E-05 |  | 0.864133 | 0.01138 | 65.6197 | 0.947415 | 0.977627498 |  | 125.1801129 | 1.07919E-07 | 1.45202E+11 | 0.651039 | 0.890878737 |  | 2.927995 | 1.629164 | 5.262303 | 0.000491 | 0.007817974 |
| metabolite1398 | 0.001905828 | 6.08E-06 | 0.597189115 | 0.034893 | 0.102939476 |  | 0.694953 | 0.006866 | 70.34098 | 0.877514 | 0.954279989 |  | 0.000117906 | 2.65071E-14 | 524456.0921 | 0.426542 | 0.774359849 |  | 1.447213 | 0.750273 | 2.791549 | 0.272499 | 0.482308416 |
| metabolite1399 | 0.004442299 | 1.78E-05 | 1.110387488 | 0.057064 | 0.148606228 |  | 4.040812 | 0.049024 | 333.0658 | 0.536282 | 0.767505568 |  | 263422.5547 | 0.000164158 | 4.22712E+14 | 0.250914 | 0.641493072 |  | 1.798111 | 0.964679 | 3.351586 | 0.067438 | 0.203465568 |
| metabolite1400 | 0.01507002 | 3.49E-05 | 6.516374872 | 0.178258 | 0.332316858 |  | 1.651494 | 0.013352 | 204.2662 | 0.83865 | 0.937817718 |  | 291.3002128 | 2.38789E-08 | 3.55359E+12 | 0.632968 | 0.881911308 |  | 1.72101 | 0.870288 | 3.403327 | 0.121457 | 0.296530577 |
| metabolite1401 | 0.042219298 | 0.000183 | 9.766638444 | 0.256956 | 0.423241516 |  | 3.488465 | 0.047075 | 258.5089 | 0.570642 | 0.790253764 |  | 2914.108615 | 2.8375E-06 | 2.99278E+12 | 0.452731 | 0.789142009 |  | 1.363822 | 0.738028 | 2.520243 | 0.324132 | 0.537708209 |
| metabolite1402 | 1534.825486 | 8.784489 | 268164.6327 | 0.006297 | 0.02762391 |  | 0.857283 | 0.012788 | 57.47122 | 0.942914 | 0.977416869 |  | 0.060380149 | 9.3779E-11 | 38876109.96 | 0.786697 | 0.942360455 |  | 0.51674 | 0.286763 | 0.931153 | 0.030068 | 0.122349635 |
| metabolite1403 | 20.4451815 | 0.068374 | 6113.474682 | 0.301716 | 0.473632948 |  | 13.83782 | 0.156205 | 1225.862 | 0.253245 | 0.537946712 |  | 19520.47018 | 7.46577E-06 | 5.10394E+13 | 0.373811 | 0.736141064 |  | 0.715002 | 0.376192 | 1.358955 | 0.308118 | 0.520914323 |
| metabolite1404 | 0.019292898 | 2.92E-05 | 12.76548831 | 0.236023 | 0.400310613 |  | 0.788004 | 0.004585 | 135.427 | 0.927868 | 0.974037858 |  | 20.90263381 | 3.45548E-10 | 1.26443E+12 | 0.810774 | 0.94615772 |  | 1.437012 | 0.69034 | 2.991286 | 0.334503 | 0.547569843 |
| metabolite1405 | 117636.9448 | 475.6164 | 29095823.98 | 6.48E-05 | 0.000854972 |  | 2.349298 | 0.022204 | 248.5703 | 0.720189 | 0.877877645 |  | 0.016055634 | 2.74138E-12 | 94034226.32 | 0.719484 | 0.920542269 |  | 0.423217 | 0.221437 | 0.808865 | 0.010536 | 0.059339755 |
| metabolite1406 | 642408.3136 | 4396.056 | 93876978.55 | 7.12E-07 | 3.43E-05 |  | 0.003286 | 4.66E-05 | 0.231751 | 0.00966 | 0.13042689 |  | 1.2369E-08 | 1.04474E-17 | 14.64405499 | 0.090395 | 0.479980149 |  | 0.302101 | 0.168023 | 0.543172 | 0.000115 | 0.00305052 |
| metabolite1407 | 78005.97938 | 416.7576 | 14600651.26 | 5.02E-05 | 0.00072148 |  | 0.12904 | 0.00155 | 10.7416 | 0.366035 | 0.639829067 |  | 1.08673E-05 | 6.06255E-15 | 19479.88378 | 0.295353 | 0.67904734 |  | 0.31216 | 0.171873 | 0.566955 | 0.000217 | 0.004532241 |
| metabolite1408 | 1.871526198 | 0.02535 | 138.1683536 | 0.77574 | 0.869051617 |  | 0.674335 | 0.02278 | 19.9613 | 0.820095 | 0.928972215 |  | 0.001861065 | 1.54037E-10 | 22485.28113 | 0.451489 | 0.787987411 |  | 0.872966 | 0.537986 | 1.416524 | 0.583359 | 0.750916404 |
| metabolite1409 | 2730.998723 | 13.44386 | 554777.8618 | 0.00426 | 0.020396351 |  | 0.238601 | 0.00313 | 18.18926 | 0.518277 | 0.753654888 |  | 0.006962426 | 5.67089E-12 | 8548107.242 | 0.642705 | 0.885987004 |  | 0.470803 | 0.257073 | 0.862229 | 0.016263 | 0.079201012 |
| metabolite1410 | 369.3840157 | 1.826449 | 74704.81512 | 0.03119 | 0.094950438 |  | 9.009877 | 0.128514 | 631.6662 | 0.312881 | 0.591374838 |  | 76.71237726 | 8.81653E-08 | 66747230592 | 0.680216 | 0.903557902 |  | 0.770026 | 0.41889 | 1.415503 | 0.401978 | 0.61293609 |
| metabolite1411 | 8.40215E-05 | 3.42E-07 | 0.020654168 | 0.001136 | 0.00761013 |  | 0.044526 | 0.00049 | 4.049899 | 0.179063 | 0.454515013 |  | 0.000281409 | 8.78949E-14 | 900977.0695 | 0.465624 | 0.793356931 |  | 2.188513 | 1.160993 | 4.125425 | 0.017076 | 0.081957568 |
| metabolite1412 | 5468590.08 | 36651.33 | 815945304 | 1.78E-08 | 2.55E-06 |  | 2.446023 | 0.025933 | 230.7134 | 0.700541 | 0.869142035 |  | 396.3777464 | 1.19413E-07 | 1.31573E+12 | 0.593827 | 0.867253231 |  | 0.319228 | 0.172557 | 0.590566 | 0.000418 | 0.006962957 |
| metabolite1413 | 0.022794094 | 0.000102 | 5.113283114 | 0.173721 | 0.32675075 |  | 17.28573 | 0.242766 | 1230.803 | 0.193066 | 0.470431387 |  | 465.0884996 | 4.72776E-07 | 4.57526E+11 | 0.562156 | 0.848472323 |  | 1.485534 | 0.806816 | 2.735209 | 0.20647 | 0.404118465 |
| metabolite1414 | 320.6952456 | 1.400748 | 73421.81363 | 0.039682 | 0.113712934 |  | 101.7183 | 1.414154 | 7316.463 | 0.036333 | 0.218561613 |  | 120094.0807 | 9.7747E-05 | 1.4755E+14 | 0.275745 | 0.660614131 |  | 0.769942 | 0.413375 | 1.434075 | 0.411773 | 0.618642876 |
| metabolite1415 | 0.00105095 | 5.36E-06 | 0.206253436 | 0.012265 | 0.046548535 |  | 0.493923 | 0.006872 | 35.50006 | 0.746995 | 0.893252345 |  | 3.524418771 | 3.85501E-09 | 3222175564 | 0.904968 | 0.970355785 |  | 1.461877 | 0.796142 | 2.684301 | 0.223279 | 0.42124391 |
| metabolite1416 | 83205159.56 | 700405.1 | 9884420412 | 1.84E-11 | 2.28E-08 |  | 0.313279 | 0.003121 | 31.44808 | 0.62258 | 0.822537502 |  | 0.229987471 | 4.94755E-11 | 1069100548 | 0.897266 | 0.96870562 |  | 0.248639 | 0.135489 | 0.456284 | 1.73E-05 | 0.000891368 |
| metabolite1417 | 6423.124791 | 26.0636 | 1582917.787 | 0.002302 | 0.012942315 |  | 2.426804 | 0.026432 | 222.8136 | 0.70137 | 0.869454446 |  | 37.41829783 | 1.26134E-08 | 1.11003E+11 | 0.745413 | 0.932960791 |  | 0.627745 | 0.33063 | 1.191855 | 0.157411 | 0.343821898 |
| metabolite1418 | 488411.6438 | 3857.283 | 61842997.98 | 5.86E-07 | 3.06E-05 |  | 0.265836 | 0.003751 | 18.83827 | 0.543467 | 0.771834939 |  | 0.018493455 | 2.13857E-11 | 15992389.38 | 0.70462 | 0.913004242 |  | 0.300588 | 0.170319 | 0.530496 | 6.61E-05 | 0.002249976 |
| metabolite1419 | 53212.47876 | 321.2524 | 8814154.325 | 5.97E-05 | 0.000805753 |  | 0.8781 | 0.011586 | 66.54845 | 0.953161 | 0.979285585 |  | 2.653594599 | 2.26742E-09 | 3105542326 | 0.927176 | 0.977264574 |  | 0.394855 | 0.217902 | 0.715505 | 0.002744 | 0.025387543 |
| metabolite1420 | 87.77093366 | 0.379033 | 20324.71187 | 0.110068 | 0.238587701 |  | 103.4656 | 1.474938 | 7258.016 | 0.034611 | 0.212415725 |  | 0.065239399 | 5.35695E-11 | 79451517.06 | 0.798624 | 0.9460629 |  | 0.840388 | 0.452266 | 1.561584 | 0.583367 | 0.750916404 |
| metabolite1421 | 0.04088874 | 0.000218 | 7.674675483 | 0.233869 | 0.397566227 |  | 0.616674 | 0.009744 | 39.02778 | 0.819726 | 0.928855549 |  | 186.9563494 | 3.88268E-07 | 90021962113 | 0.609097 | 0.874215598 |  | 1.137217 | 0.628526 | 2.057611 | 0.67164 | 0.814398943 |
| metabolite1422 | 22.51949946 | 0.068169 | 7439.31201 | 0.294893 | 0.466076933 |  | 8.870109 | 0.091689 | 858.1074 | 0.351458 | 0.625845113 |  | 271449.9067 | 7.40008E-05 | 9.95733E+14 | 0.267899 | 0.653630337 |  | 0.843557 | 0.437821 | 1.625296 | 0.612147 | 0.77219049 |
| metabolite1423 | 14.18923548 | 0.076823 | 2620.741262 | 0.321322 | 0.494168131 |  | 1.440826 | 0.023237 | 89.34053 | 0.862622 | 0.948212877 |  | 1.490488945 | 3.34364E-09 | 664412795.3 | 0.968739 | 0.990165411 |  | 0.699027 | 0.388774 | 1.256869 | 0.23416 | 0.435791513 |
| metabolite1424 | 4.372893382 | 0.033906 | 563.9776088 | 0.553003 | 0.704245329 |  | 0.685787 | 0.014857 | 31.65619 | 0.847375 | 0.940012856 |  | 0.164426776 | 1.53577E-09 | 17604355.67 | 0.848579 | 0.955623187 |  | 0.669435 | 0.388885 | 1.152379 | 0.15038 | 0.33409124 |
| metabolite1425 | 7311.128064 | 40.17712 | 1330423.719 | 0.001101 | 0.007442381 |  | 0.058367 | 0.000818 | 4.163843 | 0.194643 | 0.472106925 |  | 1.42014E-07 | 1.70907E-16 | 118.0055398 | 0.135237 | 0.534467195 |  | 0.345331 | 0.192882 | 0.618273 | 0.000514 | 0.008088903 |
| metabolite1426 | 424.3951664 | 1.808533 | 99589.69195 | 0.031924 | 0.096630356 |  | 6.063915 | 0.076286 | 482.0142 | 0.421197 | 0.685629403 |  | 30.79068386 | 1.98277E-08 | 47815275051 | 0.751534 | 0.935229984 |  | 0.940355 | 0.502009 | 1.761459 | 0.848058 | 0.925219154 |
| metabolite1427 | 132856.2224 | 910.2395 | 19391353.1 | 9.60E-06 | 0.000204698 |  | 2.856309 | 0.039407 | 207.0306 | 0.631994 | 0.826559459 |  | 1.234930267 | 1.28015E-09 | 1191303696 | 0.984085 | 0.993995836 |  | 0.412139 | 0.228286 | 0.744061 | 0.003984 | 0.03139277 |
| metabolite1428 | 48.68101256 | 0.231886 | 10219.87569 | 0.15718 | 0.306031234 |  | 0.361998 | 0.005193 | 25.23648 | 0.639828 | 0.831955879 |  | 0.415396247 | 5.20503E-10 | 331514108.3 | 0.933204 | 0.97828249 |  | 0.628096 | 0.344172 | 1.146244 | 0.132544 | 0.309920393 |
| metabolite1429 | 1705.964777 | 10.20755 | 285113.9866 | 0.00522 | 0.023827138 |  | 3.316801 | 0.051272 | 214.5649 | 0.574155 | 0.791246778 |  | 309682.9414 | 0.000633108 | 1.51481E+14 | 0.21813 | 0.609109294 |  | 0.520466 | 0.29001 | 0.934053 | 0.030717 | 0.12417364 |
| metabolite1430 | 15.23403643 | 0.052605 | 4411.670211 | 0.348381 | 0.520886479 |  | 1.048086 | 0.011862 | 92.60779 | 0.983649 | 0.992676704 |  | 0.003719951 | 1.55345E-12 | 8907910.965 | 0.612678 | 0.875727365 |  | 0.912616 | 0.480822 | 1.732173 | 0.780244 | 0.884118185 |
| metabolite1431 | 9.58937785 | 0.051276 | 1793.363568 | 0.398811 | 0.56769704 |  | 3.335229 | 0.053829 | 206.6517 | 0.568389 | 0.790127792 |  | 0.001051394 | 2.40068E-12 | 460465.2218 | 0.500759 | 0.812201491 |  | 1.041508 | 0.576705 | 1.880925 | 0.892971 | 0.94924513 |
| metabolite1432 | 29.25079208 | 0.149564 | 5720.672935 | 0.212425 | 0.373817901 |  | 0.497221 | 0.007593 | 32.56073 | 0.743917 | 0.89169117 |  | 1.51536E-05 | 2.88321E-14 | 7964.490362 | 0.281068 | 0.665728265 |  | 0.748938 | 0.41265 | 1.359282 | 0.343852 | 0.556007118 |
| metabolite1433 | 361.4711369 | 2.161127 | 60459.82613 | 0.026093 | 0.083762903 |  | 0.059907 | 0.001004 | 3.576275 | 0.180013 | 0.455371156 |  | 0.033940221 | 7.88119E-11 | 14616294.64 | 0.739358 | 0.929457427 |  | 0.588414 | 0.328986 | 1.052421 | 0.076543 | 0.221396397 |
| metabolite1434 | 39995.53493 | 196.9963 | 8120167.646 | 0.00016 | 0.001686035 |  | 0.522538 | 0.006036 | 45.23908 | 0.776045 | 0.909634389 |  | 6.48526E-05 | 3.1163E-14 | 134963.3684 | 0.380267 | 0.740383694 |  | 0.380324 | 0.206133 | 0.701713 | 0.002503 | 0.023675386 |
| metabolite1435 | 1767861.243 | 14168.58 | 220582020.5 | 5.24E-08 | 5.26E-06 |  | 0.026344 | 0.00036 | 1.927536 | 0.099662 | 0.343190353 |  | 3.38825E-06 | 3.02813E-15 | 3791.211021 | 0.238617 | 0.629502794 |  | 0.215133 | 0.123871 | 0.373634 | 3.00E-07 | 8.65E-05 |
| metabolite1436 | 23873059.08 | 213357.9 | 2671205718 | 1.54E-10 | 9.55E-08 |  | 0.582113 | 0.006662 | 50.86365 | 0.812905 | 0.926554541 |  | 9.29448017 | 3.98914E-09 | 21655609953 | 0.839827 | 0.954234322 |  | 0.230274 | 0.129146 | 0.410591 | 2.38E-06 | 0.000231136 |
| metabolite1437 | 35.18129294 | 0.158925 | 7788.080282 | 0.198911 | 0.356094071 |  | 114.2874 | 1.727115 | 7562.679 | 0.028768 | 0.19733666 |  | 546374.9124 | 0.000668055 | 4.46857E+14 | 0.209687 | 0.602134961 |  | 0.945734 | 0.512475 | 1.745279 | 0.858671 | 0.932005434 |
| metabolite1438 | 166.4797279 | 0.691491 | 40080.8036 | 0.070213 | 0.171659066 |  | 0.457042 | 0.005723 | 36.49979 | 0.726739 | 0.882213768 |  | 12336.43841 | 8.69942E-06 | 1.7494E+13 | 0.382813 | 0.741063249 |  | 0.643382 | 0.345574 | 1.197833 | 0.167084 | 0.357827706 |
| metabolite1439 | 4.672845303 | 0.020941 | 1042.704851 | 0.577426 | 0.722647953 |  | 128.5418 | 1.991147 | 8298.227 | 0.02428 | 0.185877348 |  | 3368.414388 | 4.13314E-06 | 2.74518E+12 | 0.439485 | 0.782677331 |  | 1.225078 | 0.666432 | 2.252016 | 0.51476 | 0.697316729 |
| metabolite1440 | 13092.81752 | 55.91266 | 3065886.582 | 0.00092 | 0.006504952 |  | 0.065012 | 0.000732 | 5.773573 | 0.235007 | 0.515872016 |  | 2.72676E-10 | 1.39177E-19 | 0.534227958 | 0.046063 | 0.381128688 |  | 0.409276 | 0.219261 | 0.76396 | 0.005933 | 0.041099553 |
| metabolite1441 | 3155243.195 | 22812.78 | 436402810.5 | 3.18E-08 | 3.81E-06 |  | 0.024095 | 0.000295 | 1.969939 | 0.100093 | 0.343190353 |  | 4.71948E-06 | 2.43002E-15 | 9165.975173 | 0.263477 | 0.64884445 |  | 0.281822 | 0.155821 | 0.509709 | 5.64E-05 | 0.00206643 |
| metabolite1442 | 235.5586858 | 1.095723 | 50640.42186 | 0.048675 | 0.13272035 |  | 0.031894 | 0.000452 | 2.24949 | 0.115436 | 0.36576254 |  | 4.33473E-05 | 4.51958E-14 | 41574.50148 | 0.343119 | 0.714142474 |  | 0.470263 | 0.258225 | 0.856411 | 0.015161 | 0.075518652 |
| metabolite1443 | 0.029983937 | 0.000101 | 8.896760976 | 0.229815 | 0.392111984 |  | 9.394925 | 0.105121 | 839.6507 | 0.330556 | 0.607102955 |  | 458531543.9 | 0.221445384 | 9.49449E+17 | 0.071109 | 0.447263726 |  | 1.567045 | 0.826291 | 2.971872 | 0.1717 | 0.363501683 |
| metabolite1444 | 0.096015604 | 0.000186 | 49.54633866 | 0.463711 | 0.628040944 |  | 14.33943 | 0.106192 | 1936.3 | 0.289635 | 0.573923383 |  | 1.66887E+11 | 12.72103088 | 2.18939E+21 | 0.031832 | 0.324524805 |  | 1.439405 | 0.713348 | 2.904455 | 0.311405 | 0.524330215 |
| metabolite1445 | 5326.99512 | 28.20592 | 1006061.18 | 0.001742 | 0.010461483 |  | 1.154016 | 0.015439 | 86.25934 | 0.948227 | 0.977627498 |  | 2.76504E-06 | 2.89272E-15 | 2643.002833 | 0.227661 | 0.620300106 |  | 0.489932 | 0.268161 | 0.89511 | 0.022149 | 0.097617539 |
| metabolite1446 | 3439.485824 | 18.86236 | 627178.4098 | 0.002726 | 0.014591755 |  | 71.61018 | 1.079417 | 4750.731 | 0.048415 | 0.24747673 |  | 4658.16336 | 5.60251E-06 | 3.873E+12 | 0.421946 | 0.77189627 |  | 0.569493 | 0.312023 | 1.039416 | 0.069323 | 0.207801866 |
| metabolite1447 | 86948.552 | 454.9876 | 16615948.21 | 4.58E-05 | 0.000674323 |  | 2.107188 | 0.024435 | 181.7134 | 0.743703 | 0.89169117 |  | 0.15860322 | 7.20868E-11 | 348953884.1 | 0.867069 | 0.962305681 |  | 0.398804 | 0.215714 | 0.737295 | 0.00409 | 0.032023101 |
| metabolite1448 | 118.8815697 | 0.603079 | 23434.46061 | 0.07907 | 0.187925621 |  | 0.041909 | 0.000643 | 2.733216 | 0.139514 | 0.402308456 |  | 0.022334635 | 3.26304E-11 | 15287444.93 | 0.714871 | 0.920092212 |  | 0.509148 | 0.282143 | 0.918796 | 0.027003 | 0.112974539 |
| metabolite1449 | 0.385232974 | 0.001934 | 76.74704612 | 0.724655 | 0.837494785 |  | 0.024842 | 0.000406 | 1.520703 | 0.081109 | 0.310772257 |  | 8.05450201 | 1.46928E-08 | 4415429918 | 0.839343 | 0.954234322 |  | 0.950551 | 0.523438 | 1.72618 | 0.867988 | 0.937004424 |
| metabolite1450 | 42.20779527 | 0.155906 | 11426.75389 | 0.193018 | 0.349695824 |  | 28.13991 | 0.345158 | 2294.182 | 0.140049 | 0.402478569 |  | 2.1123E+12 | 1988.902993 | 2.24336E+21 | 0.008572 | 0.188291096 |  | 0.615605 | 0.328087 | 1.155087 | 0.133637 | 0.310712625 |
| metabolite1451 | 1084724.426 | 7317.732 | 160791228.2 | 3.09E-07 | 1.91E-05 |  | 1.047493 | 0.012461 | 88.05327 | 0.983664 | 0.992676704 |  | 14.08918834 | 7.34022E-09 | 27043481749 | 0.808786 | 0.9460629 |  | 0.250945 | 0.14059 | 0.447923 | 8.26E-06 | 0.000528474 |
| metabolite1452 | 2313311.685 | 18874.7 | 283522965 | 2.86E-08 | 3.54E-06 |  | 0.118685 | 0.001556 | 9.055264 | 0.337287 | 0.613183543 |  | 0.000130918 | 1.0597E-13 | 161739.8061 | 0.40434 | 0.762519149 |  | 0.227596 | 0.130209 | 0.397822 | 9.37E-07 | 0.00012218 |
| metabolite1453 | 9074.965655 | 31.60188 | 2606015.961 | 0.002061 | 0.01196668 |  | 3.300847 | 0.031646 | 344.3 | 0.615511 | 0.817816838 |  | 0.000171232 | 3.23519E-14 | 906297.2937 | 0.449345 | 0.78638921 |  | 0.425923 | 0.223171 | 0.812874 | 0.010936 | 0.060479946 |
| metabolite1454 | 79075.68693 | 486.4358 | 12854653.55 | 3.13E-05 | 0.000508551 |  | 1.679706 | 0.022008 | 128.1985 | 0.815039 | 0.926554541 |  | 0.004419337 | 3.719E-12 | 5251555.197 | 0.612074 | 0.875638024 |  | 0.391398 | 0.215839 | 0.709753 | 0.002538 | 0.023905684 |
| metabolite1455 | 1.37519159 | 0.005645 | 335.0425623 | 0.909741 | 0.952493538 |  | 0.11725 | 0.001576 | 8.722767 | 0.331737 | 0.607338753 |  | 0.047019418 | 4.04963E-11 | 54593230.81 | 0.774587 | 0.940047404 |  | 0.856773 | 0.461635 | 1.590133 | 0.625141 | 0.780501468 |
| metabolite1456 | 372.0939087 | 2.097229 | 66017.54074 | 0.027062 | 0.085688195 |  | 24.01978 | 0.387534 | 1488.771 | 0.133941 | 0.394488505 |  | 3.76735249 | 6.93779E-09 | 2045744581 | 0.897389 | 0.96870562 |  | 0.553455 | 0.307991 | 0.994548 | 0.050374 | 0.168717728 |
| metabolite1457 | 519.6245546 | 2.901101 | 93071.43902 | 0.019899 | 0.067686162 |  | 5.251539 | 0.080716 | 341.6754 | 0.437903 | 0.695686251 |  | 199.9578825 | 3.45738E-07 | 1.15646E+11 | 0.607791 | 0.874215598 |  | 0.579782 | 0.321311 | 1.046171 | 0.072984 | 0.215093315 |
| metabolite1458 | 36.97406182 | 0.180209 | 7586.095938 | 0.186535 | 0.342180649 |  | 5.195942 | 0.076807 | 351.5038 | 0.44507 | 0.699260511 |  | 20251.83223 | 3.08278E-05 | 1.33041E+13 | 0.340516 | 0.713874057 |  | 0.88869 | 0.48576 | 1.625843 | 0.702514 | 0.834517832 |
| metabolite1459 | 334.4106267 | 1.670568 | 66941.59749 | 0.033742 | 0.100413437 |  | 2.382248 | 0.033786 | 167.9737 | 0.690087 | 0.864005738 |  | 0.002218029 | 2.72848E-12 | 1803077.054 | 0.560523 | 0.847673811 |  | 0.673572 | 0.367912 | 1.233173 | 0.202963 | 0.400209546 |
| metabolite1460 | 212236.1756 | 1897.802 | 23734924.76 | 1.43E-06 | 5.48E-05 |  | 0.957406 | 0.015457 | 59.30057 | 0.983541 | 0.992676704 |  | 0.661760068 | 1.49637E-09 | 292658414.6 | 0.96765 | 0.989787236 |  | 0.342469 | 0.196496 | 0.596884 | 0.000254 | 0.005033333 |
| metabolite1461 | 0.007923681 | 1.06E-05 | 5.9493813 | 0.154922 | 0.303559958 |  | 264.602 | 1.521657 | 46011.84 | 0.036276 | 0.218561613 |  | 93543029.35 | 0.001111587 | 7.8719E+18 | 0.155517 | 0.560993738 |  | 2.035345 | 0.970153 | 4.27008 | 0.062751 | 0.194218757 |
| metabolite1462 | 0.051282662 | 0.000229 | 11.49475035 | 0.284395 | 0.45628648 |  | 0.004765 | 7.39E-05 | 0.307441 | 0.013343 | 0.148274315 |  | 1.53835E-08 | 2.13278E-17 | 11.09593749 | 0.086636 | 0.475490674 |  | 1.114782 | 0.604219 | 2.056768 | 0.728708 | 0.851190927 |
| metabolite1463 | 0.000326621 | 1.56E-06 | 0.068382005 | 0.003948 | 0.019198815 |  | 0.122876 | 0.001583 | 9.53848 | 0.347093 | 0.620752441 |  | 1.229861386 | 8.60115E-10 | 1758554606 | 0.984687 | 0.994332699 |  | 1.628424 | 0.877409 | 3.02227 | 0.125075 | 0.301398257 |
| metabolite1464 | 0.000193647 | 7.76E-07 | 0.048294251 | 0.002986 | 0.01549611 |  | 162.9262 | 1.955914 | 13571.64 | 0.025945 | 0.189924128 |  | 1002567.302 | 0.00038927 | 2.58212E+15 | 0.213983 | 0.604329586 |  | 2.405375 | 1.286192 | 4.498415 | 0.007 | 0.04589891 |
| metabolite1465 | 2.194382942 | 0.012157 | 396.0875216 | 0.767427 | 0.865366827 |  | 70.59864 | 1.273003 | 3915.285 | 0.040033 | 0.227506275 |  | 0.034501631 | 9.24568E-11 | 12874793.54 | 0.738762 | 0.929022922 |  | 1.266008 | 0.706124 | 2.269821 | 0.430143 | 0.632093157 |
| metabolite1466 | 3.670289387 | 0.01431 | 941.3542246 | 0.646818 | 0.780540757 |  | 0.110412 | 0.001421 | 8.579363 | 0.32326 | 0.599929612 |  | 0.199829998 | 1.38167E-10 | 289012720.9 | 0.881323 | 0.965877673 |  | 0.88682 | 0.474674 | 1.656822 | 0.707141 | 0.837715271 |
| metabolite1467 | 0.037142186 | 0.000127 | 10.8230795 | 0.257828 | 0.42430101 |  | 2.13204 | 0.023869 | 190.4411 | 0.741782 | 0.890859072 |  | 1242.888732 | 4.94957E-07 | 3.12103E+12 | 0.520109 | 0.822379046 |  | 1.35952 | 0.716602 | 2.579249 | 0.349231 | 0.56079513 |
| metabolite1468 | 637.5797727 | 2.729056 | 148955.5128 | 0.022124 | 0.073634041 |  | 2.898131 | 0.035831 | 234.4123 | 0.6359 | 0.828881657 |  | 931802190.4 | 0.808043906 | 1.07452E+18 | 0.054919 | 0.402327874 |  | 0.642813 | 0.344541 | 1.1993 | 0.167666 | 0.358603931 |
| metabolite1469 | 1334740.252 | 10490.78 | 169818719.6 | 9.80E-08 | 8.08E-06 |  | 0.032388 | 0.000443 | 2.368562 | 0.120137 | 0.372764958 |  | 1.6091E-10 | 1.98786E-19 | 0.130250454 | 0.033341 | 0.330826853 |  | 0.236364 | 0.135049 | 0.413687 | 1.74E-06 | 0.000186553 |
| metabolite1470 | 98865.53854 | 678.8283 | 14398920.6 | 1.52E-05 | 0.000290638 |  | 0.016854 | 0.000253 | 1.124892 | 0.059356 | 0.265830436 |  | 5.7105E-13 | 1.27816E-21 | 0.000255131 | 0.006494 | 0.1721236 |  | 0.249997 | 0.143729 | 0.434834 | 3.17E-06 | 0.000261358 |
[truncated: 611,019 more chars]
